# Identification and extraction of cementation patterns in sand modified by MICP: New insights at the pore scale

**DOI:** 10.1371/journal.pone.0296437

**Published:** 2024-03-21

**Authors:** Baoquan Wang, Liang Guo, Xuanli Luo, Yuhong Jiang, Quanwei Li, Jiaheng Xie

**Affiliations:** School of Geoscience and Technology, Southwest Petroleum University, Xindu, Chengdu, 610500, Sichuan, China; Middle East Technical University, TURKEY

## Abstract

Microbially induced calcium carbonate precipitation (MICP) is an environmentally friendly technology that improves soil permeability resistance through biocementation. In this study, 2D microscopic analysis and 3D volume reconstruction were performed on river sand after 24 cycles of bio-treatment based on stacked images and computed tomography (CT) scanning data, respectively, to extract biocementation patterns between particles. Based on the mutual validation findings of the two techniques, three patterns in the biocemented sand were identified as G-C-G, G-C, and G-G. Specifically, 2D microscopic analysis showed that G-C-G featured multi-particle encapsulation and bridging, with a pore filling ratio of 81.2%; G-C was characterized by locally coated particle layers, with a pore filling ratio of 19.7%; and the G-G was marked by sporadic filling of interparticle pores, with a pore filling ratio of 11.7%. G-C-G had the best cementation effect and permeability resistance (effective sealing rate of 68.5%), whereas G-C (effective sealing rate of 2.4%) had a relatively minor contribution to pore-filling and flow sealing. 3D volume reconstruction showed that G-C-G had the highest pore filling rate, followed by G-G and G-C. The average filling ratios of area and volume for G-C-G were 83.979% and 77.257%, respectively; for G-G 20.360% and 23.600%; and for G-C 11.545% and 11.250%. The analysis of the representative element volume (REV) was conducted, and the feasibility and reliability of the micro-scale pattern extraction results were confirmed to guide the analysis of macro-scale characteristics. The exploration of the effectiveness of cementation patterns in fluid sealing provides valuable insights into effective biocementation at the pore scale of porous media, which may inspire future research.

## 1 Introduction

Microbially or biofilm induced calcium carbonate precipitation (MICP) is a promising technique for enhancing soil properties through the induction of mineralized precipitation facilitated by eco-friendly ureolytic bacteria [1]. Mineralized precipitation effectively fills the pore spaces among soil particles, resulting in notable alterations in the physicochemical properties of the system, such as solid phase volume, roundness, and ambient pH [2–4]. The filling of pore spaces among soil particles has been demonstrated by Whiffin et al. (2008) [2] and DeJong et al. (2010) [1], leading to an increased roughness of the particle surfaces [5–8]. Additionally, the formation of effective bridge bindings at interparticle contacts has been reported by Gomez et al. (2017) [9] and Montoya et al. (2015) [10], contributing to enhanced shear strength and stiffness [11–14], improving liquefaction resistance [4,15,16], retaining or reducing porosity and hydraulic conductivity [17–20].

Microbial biofilms have been demonstrated effectiveness in controlling water seepage from hydraulic barriers in soils by reducing fluid flow rate [21–25], pore throat size [25,26], free pore volumes [23,27,28], and flow channels [23,28]. Moreover, microbial biofilms have been shown to enhance the shear strength [25,29,30] and viscosity [23,27,28], thereby positively affecting seepage resistance. The role of the biofilm as a collector continuously attracts bacteria, further enhancing their deposition [23]. According to Stoke’s law [24,27,31], the settling velocity is proportional to the diameter squared and density of the microbial floc, which shows the importance of biofilm thickness and density in preventing washout [24,29,30]. Unfortunately, biofilms degrade over time without a nutrient supply [32]. However, when CaCO_3_ crystals are developed around biofilms, they can fill pores and bridge particles, and a stable mineral can remain even after the biofilm is dispersed or nutrient addition has ceased [33,34] effectively improving the engineering performance of the soil by continuously reducing the permeability of hydraulic barriers.

While increasing the calcium carbonate content (CCC) in permeable media, such as sandy soil, generally reduces permeability [35], the efficiency of MICP sealing and permeability reduction depends not only on the macroscopic magnitude of CCC, but also on the precipitation position of calcium carbonate around the particle (i.e., structural characteristics and cementation pattern) at the micro scale [36,37]. Recent studies have highlighted the role of multi-particle encapsulation and bridging in promoting the aggregation of CaCO_3_ in open pore throats, leading to the effective sealing of preferential flow channels [38–41]. In contrast, fragmented or point-like CaCO_3_ precipitates on particle surfaces exhibit limited sealing effects [42,43]. The effectiveness of MICP sealing is intricately linked to the accumulated content and distribution pattern of CaCO_3_ at the pore scale [44], which influences the macro-scale flow behavior of particle media with the accumulation of CaCO_3_ [25]. DeJong et al. (2010) [1] highlighted the significance of CaCO_3_ precipitates formed at particle-particle contacts, both in strength enhancement and permeability reduction, in contrast to those formed in solution or on exposed particle surfaces. Larger CaCO_3_ precipitation in open pore throats has been found to seal dominant flow channels more efficiently and achieve greater permeability reduction [25,36,45,46].

The microstructural characteristics and cementation patterns significantly influence the target application of MICP in the field [6,25,26,34], shaping preferential flow paths and affecting the porosity, flow path, and hydraulic conductivity of the fluid in biocemented materials [47–51]. Wang et al. (2019) [52] emphasized that the size, morphology, and microstructure characteristics of CaCO_3_ crystals may vary under different microenvironmental conditions, which, in turn, affect the macroscopic behavior of the permeability with the accumulation of CaCO_3_. Although researchers have realized the importance of the cementation patterns and microstructure of CaCO_3_ bonding materials, little research has been conducted on the efficiency of biological bonding from a microscopic perspective. Therefore, it is necessary to investigate the microstructural characteristics of the CaCO_3_ distribution that contribute to fluid sealing in biocemented materials.

In this study, 2D microscopic analysis and 3D volume reconstruction were performed on river sand after 24 cycles of bio-treatment based on stacked images and computed tomography (CT) scanning data, respectively, to extract biocementation patterns between particles. Furthermore, we investigated the effects of cementation pattern on pore filling and fluid sealing in biocemented sand, analyzed the role of microstructural characteristics in the macroscopic flow behavior. Exploring the effectiveness of cementation patterns in fluid sealing provides valuable insights into effective biocementation at the pore scale of porous media, which may inspire future research.

## 2 Materials and methods

### 2.1 Sand

**Fig 1:**
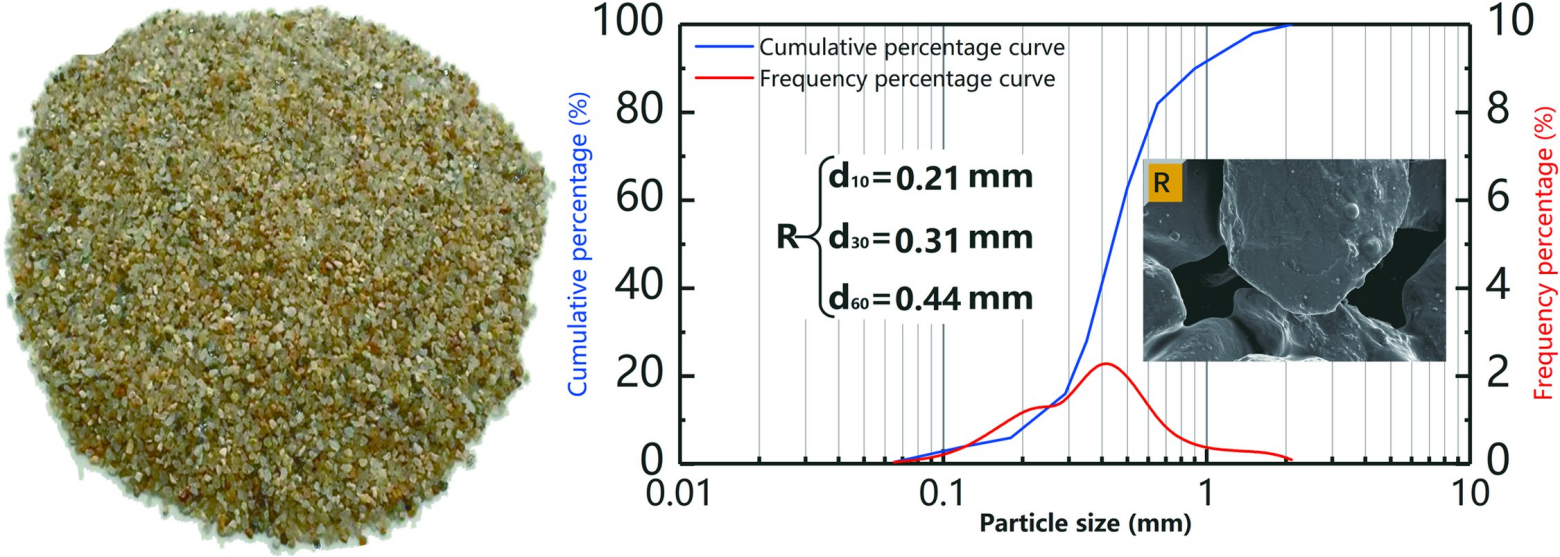
River sand particle gradation, SEM scanning image and size distribution.

River sand obtained from Chengdu city (Sichuan, China) was sieved to remove large and small amounts of detritus and impurities for the subsequent experiments. The sieved sand was washed with deionized water and dried in an oven at 60°C for 48 h. The particle size distribution of the experimental sand is presented in Fig 1, along with the SEM morphology before the MICP treatment. The SEM images reveal that the sand particles were discrete, with pores between them.

It can be observed from Fig 1 that the river sand particles have a size in the range 0.25–0.50 mm, the coefficient of uniformity *Cu*<5, and coefficient of curvature *Cc* is in the range 1–3, which is poorly graded sand (according to the Unified Soil Classification System (ASTM, 2017)) [53]. Poorly graded sands were selected for this experiment because the improvement of poorly graded sands using MICP is more reflective of the potential for biological action to enhance the soil performance than that of well-graded sands, and it is easier to observe the improvement and highlight its superiority [46,54].

### 2.2 Bacterial suspension and cementation solution

#### 2.2.1 Bacterial suspension

*Sporosarcina pasteurii* (*S*. *pasteurii*) is an oval-shaped (1.3–4.0 um in length, 0.5–1.2 um in diameter) alkalophilic heterotrophic gram-positive bacterium [1,55,56]. The microorganism was selected as the urea hydrolysis bacterium in this study owing to its alkalophilic activity (it typically occurs in an alkaline soil environment at 25–30°C) and lack of pathogenicity [57]. The *S*. *pasteurii* has been proven to have the capacity to induce CaCO_3_ precipitation on the premise of providing a calcium source.

**Fig 2:**
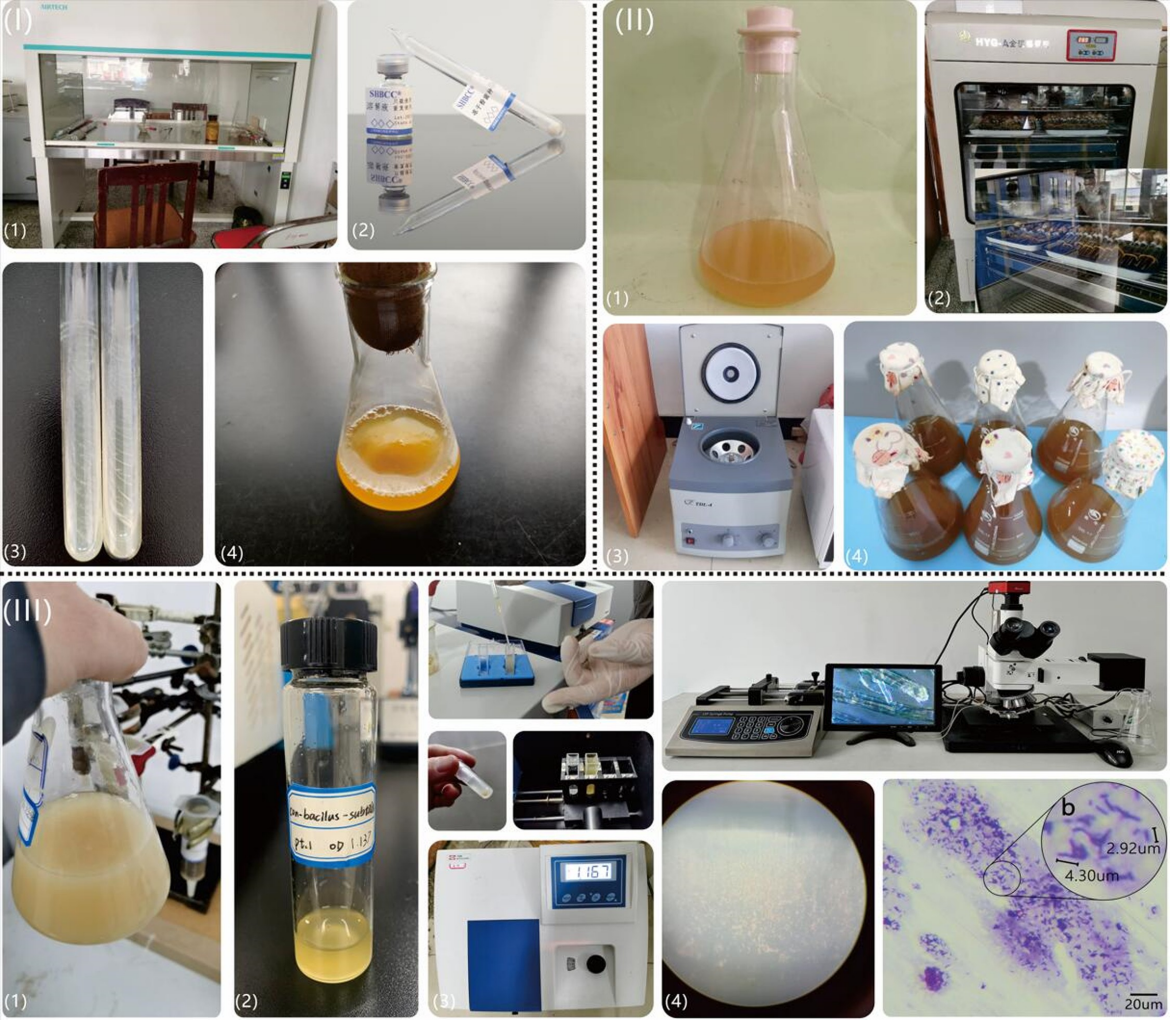
(I) Procedure for bacterial activation culture. (a) Ultraclean sterile operating table, (b) Freeze-dried powder of *Sporosarcina pasteurii* and dissolving solution, (c) Slant inoculation, and (d) Activated bacterial solution. (II) Procedure for bacterial amplification culture. (a) Activated bacterial solution in a 500-mL Erlenmeyer flask, (b) Incubation and shaking environment regulated by a cooling and heating system, (c) Low-speed centrifuge, and (d) Amplified bacterial suspension. (III) Detection of bacterial suspension. (a) Amplified bacterial suspension, (b) Bacterial suspension for detection, (c) Measurement of bacterial suspension concentration by visible spectrophotometer, and (d) Qualitative observation of the bacteria strains by the Gram-staining specimens combined with a micro fluidic device.

The purchased bacteria were in the form of freeze-dried powders. The detailed process (Fig 2A) of bacterial freeze-dried powder activation is as follows: (1) The bacterial freeze-dried powder was added to the bottom of a glass tube, and then the tip side was sterilized with alcohol and knocked open; (2) 0.2–0.3 ml of the dissolved solution was taken into a strain tube and flicked until the fungus powder was mixed evenly; (3) All the solutions were sucked out with a sterile suction head and inoculated on two inclined planes for culture; (4) The strain was added into the culture medium (see Table 1 for composition) at an ambient temperature of 28–30°C and cultivated for 1–2 days in an aerobic environment. pH of the medium was adjusted to 7.3 with 10g/L (NH_4_)_2_SO_4_ solution; (5) The utensils were sterilized at 121°C for 15 min and cooled to 60°C; (6) 100 ml of filtered and sterilized 0.5 mol/L urea solution (since urea cannot be sterilized under high pressure, it needs to be added to the culture medium after filtration and sterilization) were added to improve bacterial activity; (7) Yeast extract and agar were inoculated into the culture medium and cultured on a constant temperature shaking table at 30°C and 200 rpm.

**Table 1:** Compositions of the culture medium.

CASO Agar (g/L)	Casein (g/L)	Soybean protein (g/L)	NaCl (g/L)	ddH_2_O (L)	(NH_4_)_2_SO_4_ (g/L)	Urea (mol/L)	NiCl_2_·6H_2_O (mmol/L)
20	15	5	5	0.9	10	0.5	0.1

Note: (CaSO Agar) is soybean powder casein digestion agar medium, ddH_2_O is secondary distilled water. NiCl_2_·6H_2_O can improve urease activity.

The detailed procedure of bacterial amplification (Fig 2A) culture is as follows: (1) a certain amount (approximately 10 ml) of the solution was removed from the above-activated solution to a 500-mL Erlenmeyer flask with a sterilized growth medium (the detailed components see Table 1); (2) the Erlenmeyer flask was moved into the incubator (at a constant temperature of 30°C) for 24 h; (3) the amplified bacterial solution was centrifuged (4800 r/min) in the centrifuge for 8 min using the TDL-4 low-speed Bench Centrifuge at the State Key Laboratory of Southwest Petroleum University. The solid medium components in the bacterial solution were separated and removed under the action of centrifugal force; (4) the cells were washed twice with preheated 0.01M PBS (phosphate-buffered saline) to eliminate the growth medium. The bacterial suspension (BS) used in the experiments was prepared and the parameters obtained are listed in Table 2.

**Table 2:** The growth medium constituents and bacterial suspension parameters.

Growth medium			Bacterial suspension			
Yeast extract (g/L)	(NH_4_)_2_S0_4_ (g/L)	Tris buffer (mol/L)	OD_600_	pH	Urease activity (mmol/L/min)	Urease concentration (U/ml)
20	10	0.13	1.3	8	3.12	8.35

A certain amount of solution (Fig 2B) was extracted from the amplified bacterial suspension (Fig 2A) and placed on a visual spectrophotometer for concentration (OD_600_) detection (Fig 2C). Furthermore, to qualitatively detect the growth of bacterial strains (i.e., aggregation morphology and distribution range, reflecting bacterial activity to a certain extent), we used the Gram-staining specimens combined with a micro fluidic device for microscopic observation and resolution under unsterile conditions (Fig 2D). The rod-shaped, gram-positive purple cells of the bacteria ranged 2 to 5 um in size, with numbers in the range of approximately 7.1×10^8^–2.8×10^9^ cfu/ml.

The bacterial suspension (BS) used in the experiments was prepared and the parameters obtained are listed in Table 2.

#### 2.2.2 Cementation solution

**Fig 3:**
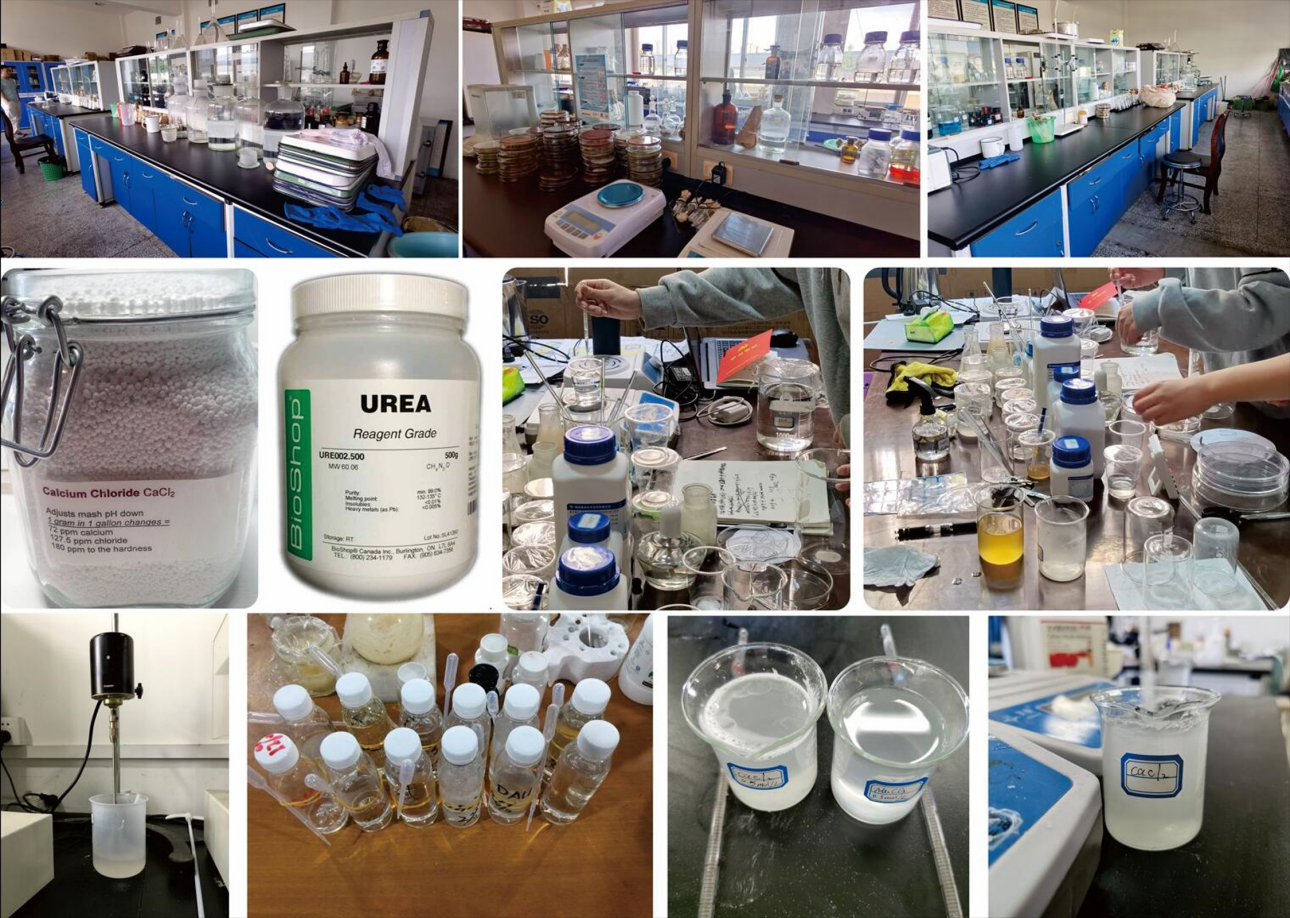
Cementation solution preparation process.

The constituents of cementation solution (CS) considered herein primarily consist of equimolar nitrogen sources and anhydrous calcium chloride (CaCl_2_) (See Fig 3 and Table 3 for operational details), in addition to part of the nutrient broth (BS can be continuously supplemented with nutrients to enable it to survive for a longer period and continue to work).

**Table 3:** Cementation solution recipes.

Molarity (mol/L)	Nitrogen source		CaCl_2_ (g/L)	Nutrient broth		
	Urea (g/L)	Ammonium chloride (g/L)		Peptone (g/L)	HM peptone B (g/L)	Sodium chloride (g/L)
0.5	30	0.6	55.5	0.6	0.6	0.3

### 2.3 Experimental apparatus and treatment protocols

#### 2.3.1 Experimental apparatus and setup

Several injection strategies for MICP treatment have been presented in biomineralization literature [2,58–61], with two predominant methods (referred to collectively as the longitudinal injection strategy in this study) emerging as the most commonly employed in practical applications [60,62,63]. Method A, designated as the mixed injection strategy, involves the initial mixing of a two-phase solution (BS and CS), followed by simultaneous injection into the specimen from the injection port [64]. Method B, termed the staged injection strategy, involves the injection of a one-phase solution initially, followed by another phase solution after a specified interval [61,65]. Both these strategies induce cementation during the infiltration process. Several scholars have observed that the simultaneous injection of two-phase solutions at low flow rates (i.e., Method A) is more prone to creating larger crystal clusters [60,63,66]. This often leads to the clogging of pore spaces in sand particles (channels for solution delivery and diffusion inside the sand specimen) near the injection port, hindering subsequent solution injection. For instance, Tobler et al. (2012) [60] observed that the implementation of a mixed injection strategy resulted in heterogeneous CaCO_3_ filling along a sand column, with a concentration of CaCO_3_ precipitation predominantly near the inlet area. In contrast, when adopting the staged injection strategy (Method B), a more homogeneous distribution emerged. Specifically, under the mixed injection strategy, the permeability of the sand column decreased by 95%. Backscattered electron imaging of the sand column revealed that this reduction in permeability was primarily due to extensive blockage within the first 1 cm of the column (total length 10 cm). CaCO_3_ filled 55 ± 20% of the pore space in this region, while the middle and lower parts of the column had a smaller amount of CaCO_3_ filling, approximately 18 ± 8%. To circumvent the heterogeneous cementation of MICP-treated sand resulting from shortcomings in the mixed injection strategy, a staged injection strategy was implemented for MICP treatment in subsequent experiments. This is crucial to ensure the consistent precipitation of CaCO_3_ throughout the sand column, thereby preventing preferential flow through high-porosity pathways.

**Fig 4:**
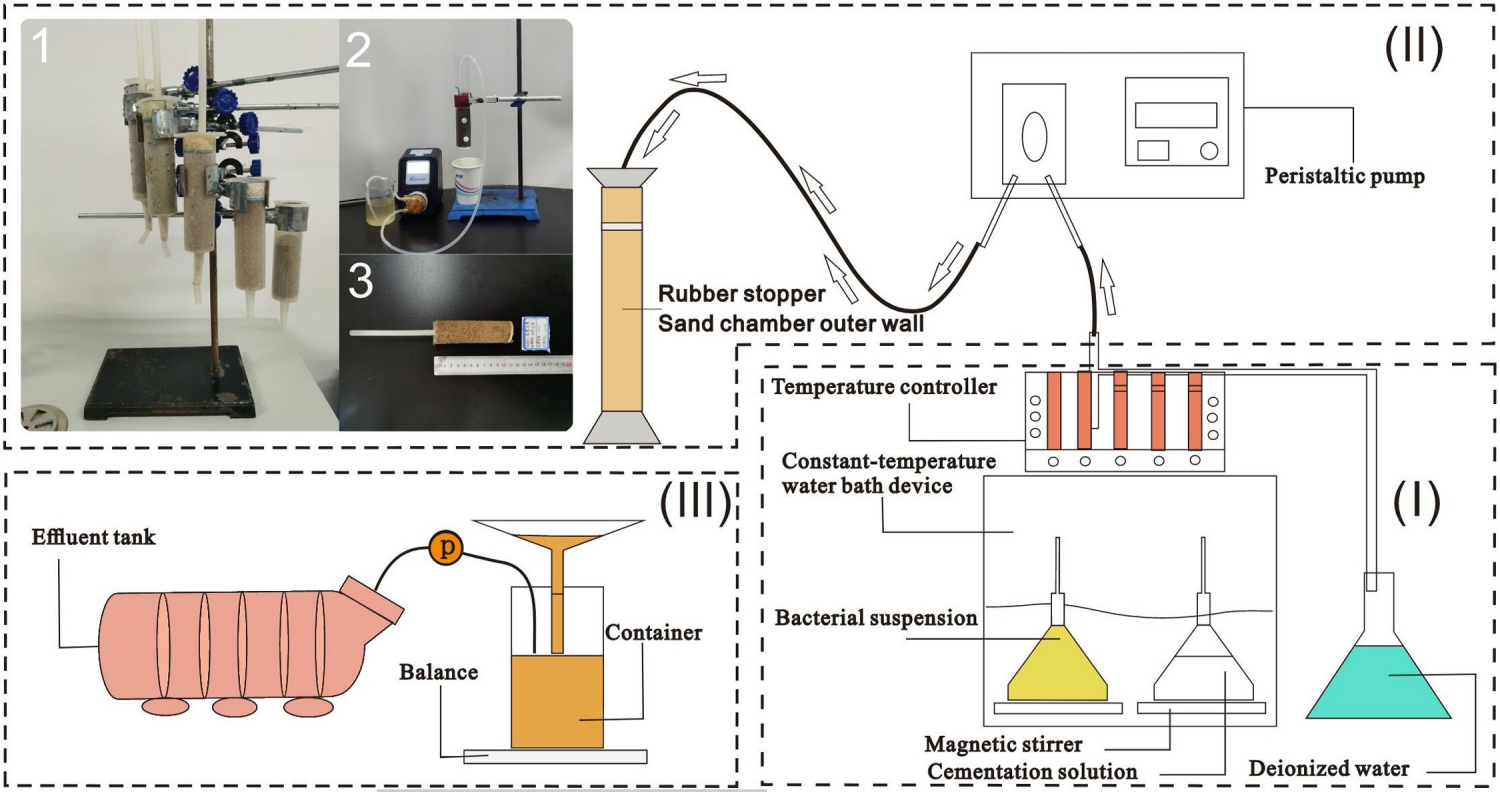
Schematic of setup for bio-treatment sand specimens with the alternate combined stop-flow injection method. (i) temperature control system, (ii) reaction system (flower tube center injection), and (iii) measurement system.

The experimental apparatus utilized for MICP treatment in this study comprised three primary components, as illustrated in Fig 4: (i) a temperature control system, (ii) a reaction system, and (iii) a measurement system.

(i) The temperature regulation apparatus comprised a constant-temperature water bath apparatus, arranged with three Erlenmeyer flasks (for BS, CS, and deionized water, positioned sequentially from left to right), and two magnetic stirrers. The magnetic stirrers were strategically positioned within the Erlenmeyer flasks containing BS and CS. Simultaneously, an Erlenmeyer flask housing deionized water (located on the right side of the incubator) was employed. Continuous stirring was applied to the BS and CS-containing flasks to avert precipitation and stratification, thereby ensuring a steadfast environment conducive to the proficient execution of the biochemical reaction within the sand chamber (throughout the injection phase). To uphold a consistent bacterial activity and CS solubility, the water bath temperature controller meticulously maintained the temperature within the 30°C range. (ii) The experimental configuration comprised a treated sand specimen situated within a cylindrical mold, facilitated by a peristaltic pump, iron frame, funnel, and beaker. Specifically, cylindrical molds fashioned from polyethylene (utilizing medical-grade PP raw material) with dimensions of 3.2 cm inner diameter and 11.3 cm height were employed, and these molds were densely filled with sand. To prevent the undesired displacement of sand particles with the effluent solution during treatment, filters crafted from metallic grids and filter paper (20–25 μm) were strategically positioned at both the upper and lower extremities of the sand specimen. The support structure for the mold and the funnel, along with a containment system for the spilled solution, were orchestrated through an iron frame table, ensuring precision in measurement and recording within the associated flask. (iii) The instrumentation for measurement encompassed a beaker container, balance, hydraulic pump, and an effluent tank. The drainage system connected to a beaker, and the cumulative liquid mass traversing the sand specimen was gauged using a balance positioned beneath the beaker. Ultimately, the percolated liquid collected in the beaker was systematically directed into the effluent tank through the utilization of a hydraulic pump.

#### 2.3.2 Treatment protocols

To prepare biocemented specimens for experimentation, river sand was introduced into a cylindrical mold, as depicted in Fig 2, forming a sand column with a 30 mm diameter and 100 mm height. Prior to the biocementation process, a uniform initial condition-setting procedure was conducted on the sand column. Vibrational consolidation was employed to establish a specific initial state. Precisely, the specimen underwent compaction and leveling to achieve a designated dry density of 1.68 Mg/m³, relative density of 37–41%, and initial porosity of 36.3–39.2%.

The biocementation treatment followed a phased injection strategy, as previously detailed by Wang et al. (2018) [67]. A peristaltic pump was utilized to inject bacterial spores (BS) and calcium source (CS) into sand specimens in batches (25 mL per batch). The injection rate was set at 5 mL/min, with an interval of 8 h between each injection. This ensured enhanced bacterial attachment to sand particle surfaces. Without this precaution, a significant number of bacteria might be washed away during subsequent injections, potentially compromising effective urea hydrolysis and impeding the precipitation of CaCO_3_.

### 2.4 Microscopic image acquisition and processing

#### 2.4.1 Images capture

Microscopic images of the tested specimens (bio-treated and control groups) were captured using a microscope (model: H-ICM-100, Ruihong, China, 10× objective (Fig 6A)): This microscope is located in the School of Geoscience and Technology, Southwest Petroleum University, and is characterized by high magnification, high resolution and high definition. With this microscope, the authors can clearly observe the microstructure and characteristics of the samples, which provides an important basis for geological analysis and research. (Licensed by School of Geoscience and Technology, Southwest Petroleum University).

In this investigation, a comprehensive examination of the microstructure of sand was undertaken employing microscopic techniques. The primary objective was to gain an in-depth understanding of crucial properties, encompassing morphology, size, distribution, and inter-particle interactions of soil particles. Microscopic observations were conducted utilizing a high-resolution microscope, and advanced optical techniques were applied to meticulously adjust lenses and light sources, ensuring the acquisition of clear and high-contrast microscopic images. This facilitated a detailed exploration of various characteristics of sand column particles before and after biocementation, encompassing the diversity of sand particle shapes, particle sizes, distribution of CaCO_3_ precipitation, and potential microstructures between particles (i.e., cementation patterns).

By conducting meticulous microscopic observation and image capture, we curated a comprehensive image database to record and preserve the identified microstructures. These image data not only offered researchers intuitive visual insights but also established the groundwork for subsequent quantitative analysis and data processing. In the data analysis stage, image processing software was utilized for more in-depth quantitative and semi-quantitative analysis of microscopic images, delving into crucial parameters like particle size distribution and particle shape. This approach facilitated a more profound comprehension of the microstructure of sand bodies, furnishing more objectively grounded data on the macro-mechanical properties of soils.

In summary, employing the microscopic observation method, we not only scrutinized the micro-characteristics of sand bodies but also furnished substantial theoretical and methodological underpinning to the domain of geotechnical engineering. This has bestowed a pivotal scientific foundation for the examination and application of soil’s mechanical properties.

#### 2.4.2 Stack processing

**Fig 5:**
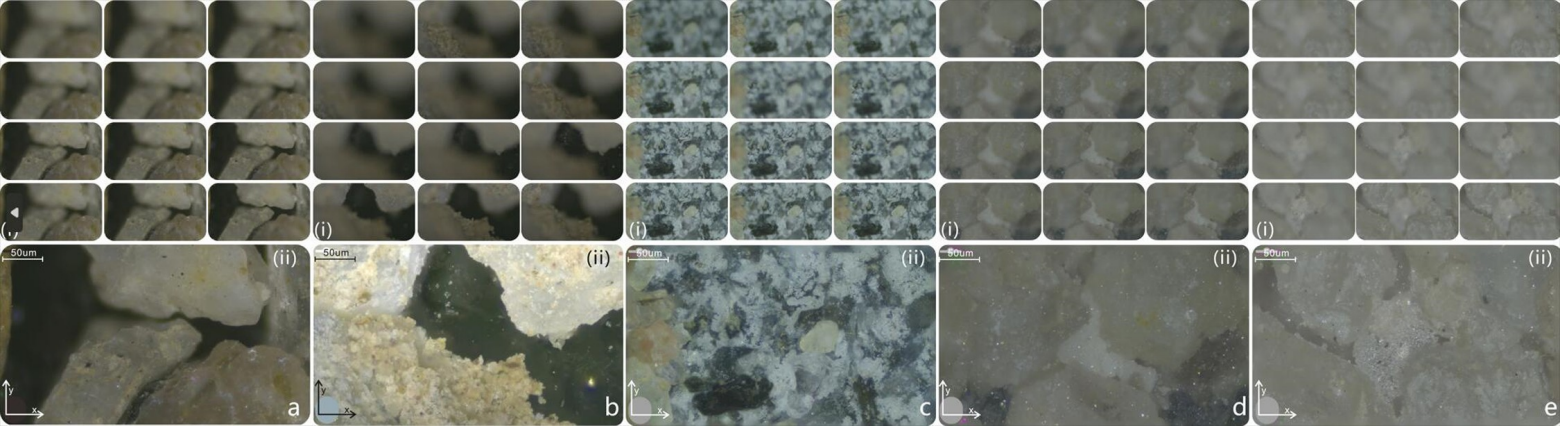
Stack processing of microscopic images. The different regions in the sands are a-e. The 12 subgraphs in (i) indicate that the processing process is excessive from ambiguity to clarity. (ii) is the clear diagram after stack.

Microscopic images frequently encounter challenges related to focal depth and uneven specimen surfaces, especially at high magnifications, resulting in blurred regions within the images. Image stacking techniques are commonly employed in the post-processing of microscopic images to improve clarity and recover information. To clearly analyze the microstructure of the sand specimens, we employed an image stacking method to overcome the challenge of blurred microscopic images caused by variations in height. Details of the operation are described below (Fig 5):

1. Construction of Image Stack: The process of creating a microscopic image stack initiates with capturing a sequence of images at various focal positions. These images are organized in a specific order, typically through incremental focusing steps, to encompass the entire region of interest. Stack construction ensures the collection of clear information at diverse specimen depths;
2. Image Alignment and Fusion: Given the incremental focusing steps in microscopic image stacks, precise image alignment is critical. It is imperative to align the images in the stack accurately, preventing the introduction of artifacts or distortions during subsequent fusion operations. Common image-alignment methods involve feature matching and fitting transformation models. In the fusion stage, techniques like weighted averaging or pyramid fusion are commonly employed to amalgamate information from multiple focus points, effectively minimizing blurriness and enhancing overall clarity;
3. Blur Restoration and Enhancement: Within a microscopic image stack, fusing a sequence of images proves effective in restoring local details that may be blurry or distorted. This procedure is designed to accentuate specimen structures, elevating both image detail and clarity. Commonly employed algorithms for blur restoration and image enhancement include blind deconvolution and non-local means filtering;
4. Application and Effects: The implementation of this method, aimed at reducing blurriness and enhancing image details, enhances the visual quality of microscopic images. This, in turn, establishes a clearer and more reliable foundation for subsequent analyses and results.

The image stack technique involves amalgamating a set of reference frames that exhibit similarities but may vary in quality or content, aiming to produce a clearer and higher-quality image. This method is particularly useful for creating composite images by combining multiple similar images. Initially, we carefully selected at least two images with comparable size and content. Subsequently, these images were merged into a multi-layered image using the "script load stack" option in Photoshop software. The layers were automatically aligned, each layer was converted into a smart object, and the desired stack mode was chosen. The editing process included the selection of the desired smart object layer. Following editing, the smart object automatically rendered in stack mode. Lastly, the smart object stack was converted into a regular layer stack image.

#### 2.4.3 Three-phase (particles, pores, cements) identification and cementation patterns extraction

We digitized the stacked images and removed any unwanted content or noise to generate a composite view, which allowed us to extract internal structural information for further analysis. Using these improved images, we employed the pores (particles) and cracks analysis system (PCAS) to capture the contact between the biocement and sand particles, which enabled us to quantify microstructural parameters (such as the pore filling ratio) and microstructural patterns (such as particle-cementation patterns) related to the biocement content and morphology.

The stacked microscope image (shown in the first column of Fig 10) was subjected to gray recognition, that is, according to the different gray values, the PCAS software was used to roughly identify the three-phase materials of particles, pores, and CaCO_3_ (second column of Fig 10). Although the solid phase (particles and CaCO_3_) and pore phase can be well separated in the grey segmentation step, the particles and the CaCO_3_ cannot be well identified and analyzed. Therefore, we must further process the stack and grey segmentation images. We applied the layer (same size) superposition method to identify and separate the three phases (third column of Fig 10), that is, when analyzing a single phase, the other two phases were ignored. When each phase material was identified and filled, the layer was discharged from bottom to top according to the order of analysis: pore (white), particle (orange), CaCO_3_ (blue). After the three-phase material is identified, PCAS was used to denoise each phase in the identified image (see the original image on the left side of the fourth column in Fig 10 and the display after noise reduction in the middle), re-formulate the color processing (see the right side of the fourth column in Fig 10, namely brown particles, green pores, and yellow CaCO_3_), and calculate the area of each phase (fifth column in Fig 10) to accurately calculate the pore filling rate and effective calcium carbonate sealing efficiency.

### 2.5 CT scan data acquisition and processing

#### 2.5.1 X-CT Theory and data acquisition

**Fig 6:**
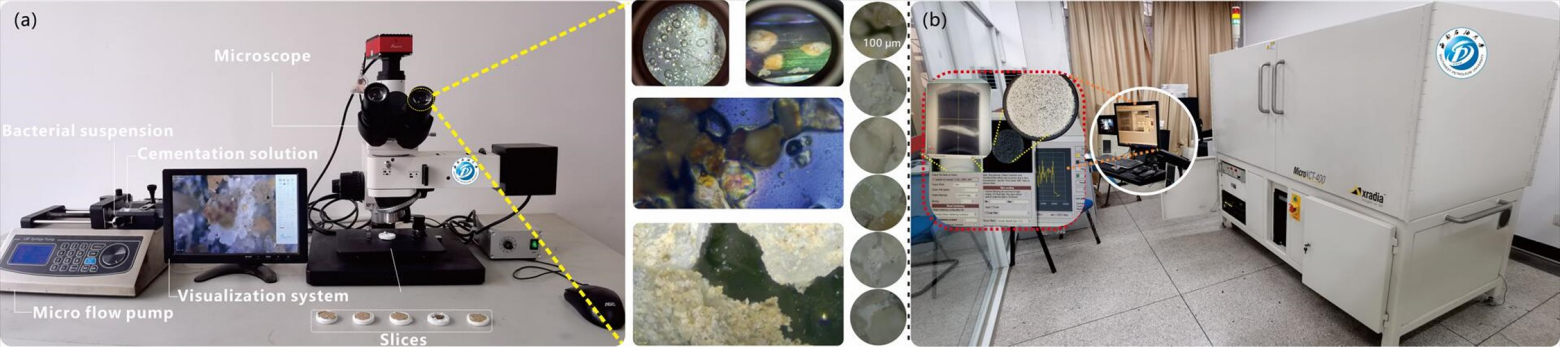
Procedures of image data acquisition. (a) acquisition of microscopic images and stack images based microstructure analysis, and (b) micro X-CT scanning.

Micro-CT scanning is regarded as a powerful approach for visualizing the microstructure of porous media [68,69]. A micro-X-CT (ZEISS XRADIA MICROXCT-400×) scan test (Fig 6B) was performed at the State Key Laboratory of Reservoir Geology and Development, Southwest Petroleum University, China. The 3D internal variations in the X-ray attenuation coefficient were used to obtain images of the biocemented sand specimens.

When photons interact with solids and complex biological materials, several processes occur, including absorption, scattering (elastic or inelastic), diffraction, refraction, or transmission through the material. The absorption of photons leads to the emission of electrons, visible light, and X-rays. At a fundamental level, when an X-ray photon is absorbed by an atom, it causes the ejection of electrons from the inner shell of the atom. The ionized atom eventually returns to its neutral state by filling an electron in the vacated spot in the inner shell, often accompanied by the emission of X-rays characteristic of the atom. As X-rays traverse through a solid object, their attenuation follows the Lambert–Beer law:

$$I=I_{0}\times e^{-\sum_{i}\mu_{i}x_{i}}$$ (1)

where *I* is the strength of X-ray after penetrating the core; *I*_*0*_ is the initial intensity of X-ray; *μ* is the attenuation coefficient of X-ray under the ith component; *x*_*i*_ is the propagation distance of ray through component *i*.

The Lambert–Beer law holds strictly for a pure monochromatic beam, where all photons share the same energy and wavelength. However, current X-ray sources, except for the free-electron laser, generate polychromatic beams analogous to white light, consisting of a spectrum of photon energies. Therefore, unless a monochromator selectively filters out all but a few wavelengths, the beam comprises multiple wavelengths. As the attenuation coefficient *l* varies with energy, the decay of beam intensity is not exponential. The attenuation coefficient is also influenced by the atomic electron density and the bulk density of the material. At low X-ray energies (50–100 keV), photoelectric absorption (strongly dependent on atomic number) predominantly governs X-ray interactions. Yet, for energies up to 5–10 MeV, photon attenuation is primarily due to Compton scattering, largely controlled by electron density. Finally, for photon energies exceeding 10 MeV, interactions are dominated by pair production [50]. A more comprehensive exploration of X-ray attenuation and its dependence on the mentioned variables concerning X-ray energy can be found in [44,50]. According to Eq (1), *I* is a decreasing function of distance x (the exponential argument is negative), signifying that incident intensity *I*_*0*_ is attenuated as X-rays traverse the object. Consequently, materials with a high attenuation coefficient enable X-rays to penetrate only a relatively short distance. Information regarding the absorption properties of elements and compounds across a broad spectrum of energies can be accessed through various online resources, such as the NIST XCOM Photon Cross-sections Database.

From Eq (1), the average linear attenuation coefficient of the (composite) material can be calculated using the measured values of *I*_*0*_ and *I(x)*. Due to the linear addition of attenuation coefficients for composite materials, and if the volume fractions *X*_*v*_ are known, it is straightforward to compute the linear attenuation coefficients for the composite parts. Achieving accurate identification and classification of objects or materials with different compositions and densities requires a sufficient variation in attenuation coefficients. To obtain data in three dimensions, the object is rotated in the beam, and a large number of 2D radiographic projections are collected at different angles. This process allows the full 3D distribution of attenuation coefficients to be mathematically back-calculated, i.e., reconstructed.

#### 2.5.2 3D volume reconstruction and bio-cementation patterns extraction

1. Micro-CT imaging

**Fig 7:**
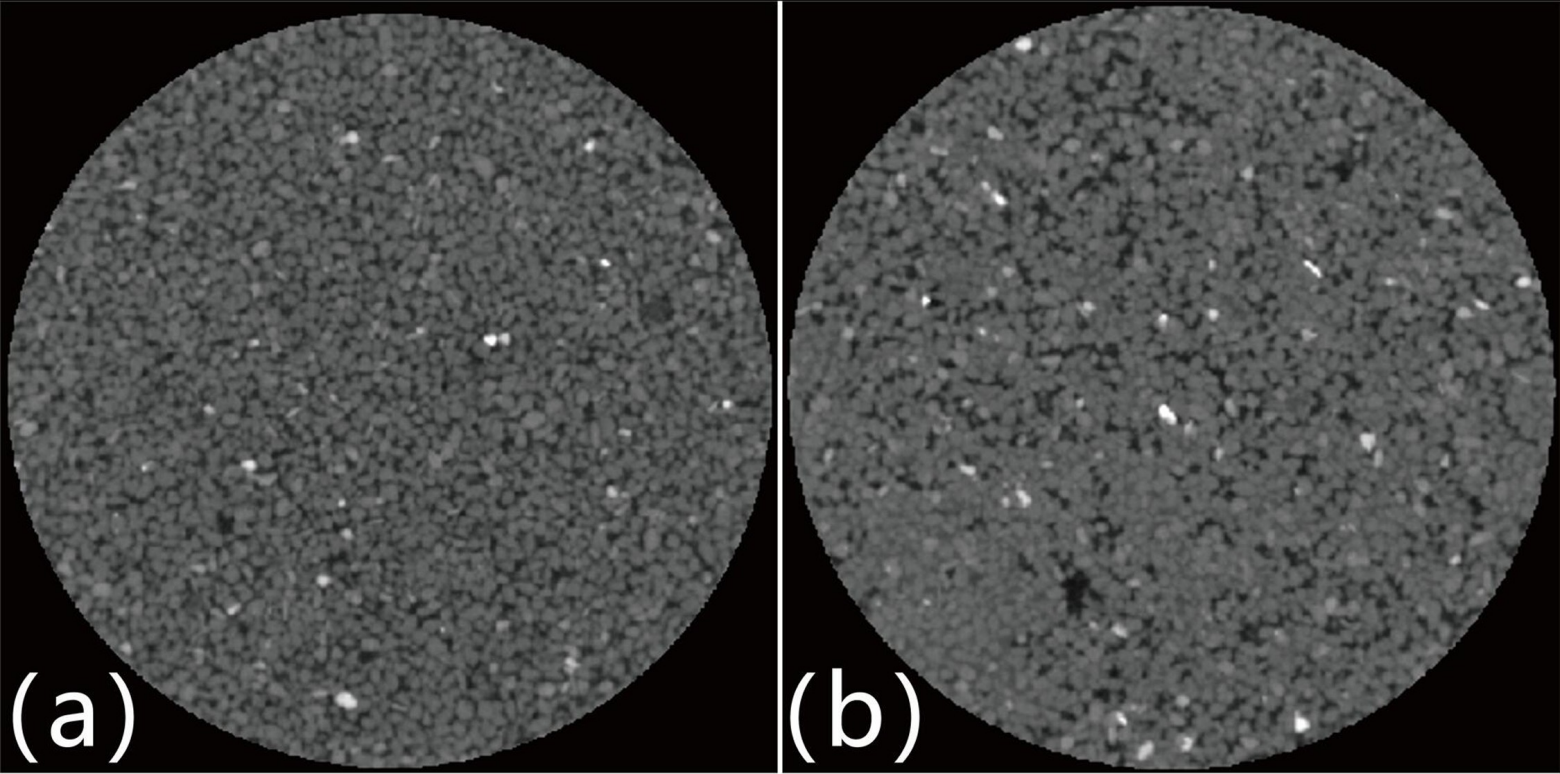
CT 2D sections of (a) unbiocemented sand, and (b) biocemented sand.

Micro-CT scanning, a non-destructive technique for examining the internal structure of objects, is currently the most direct and accurate method for constructing 3D digital rock cores. The technique is based on the principle of differential absorption coefficients of X-rays by components with different densities in rocks to distinguish between pores and the rock matrix. In this study, 3D images of rock cores were acquired using a Micro-CT 400 system, Xradia (Fig 6B). The system can achieve a maximum sampling resolution of 1 *μ*m. The experimental specimens consisted of cylindrical sandstone approximately 8 mm in diameter. Each specimen yielded 983 2D CT slice images with a resolution of 980 × 1005 pixels and spatial resolution of 2.1 *μ*m/voxel. These 2D slice images were sequentially stacked and combined to generate a 3D grayscale image of the rock specimen. Fig 7 shows illustrates one grayscale slice, where the rock matrix (high-density) appears in gray and white shades, whereas the pores (low-density) are clearly distinguishable in black.

A large number of 2D images of sand specimens were obtained using CT scanning technology. The micro CT data were reconstructed into a 3D model and imported into the Avizo software for visualization and quantitative analysis of the specimens. To ensure that the digital model truly reflected the actual pore structure characteristics of the sand specimens, a circular section was selected from the middle of the sand specimen scanning image for 3D reconstruction. The reconstructed 3D body provided microscale pore data, including throat size, coordination number, pore-filling rate, and flow channel, which were comprehensively analyzed using various methods. A total of 3612 consecutive images were selected from the scanned sand CT images. As the quality of the original data was excellent, it did not need to be filtered and could be directly reconstructed in 3D. In the 3D reconstruction, the center point of the sand specimens slice was used as a cylinder with a radius of 21.17 *μ*m and a height of 25.36 *μ*m to frame the research area.

2. Image processing and acquisition

The grayscale images obtained from the micro-CT scanning of rock cores exhibit various types of systematic noise, which not only reduces the image quality, but also hinders subsequent quantitative analysis. Therefore, the first step in image processing is to enhance the signal-to-noise ratio using filtering algorithms. Commonly used filtering algorithms include low-pass linear filtering, Gaussian smoothing, and median filtering. After a comprehensive comparison of the filtering effects of these algorithms, the median filter was selected.

After the grayscale rock core image was filtered with a median filter, the transition between the pores and rock matrix became more natural, and the boundaries became smoother (Fig 7). Important features of the image were preserved as much as possible. However, to differentiate and quantify the pores and rock matrix better, an image segmentation method is required to obtain a reasonable binary division of the grayscale image.

The key to image binarization is the selection of a segmentation threshold. Given that the actual porosity of the rock cores used in this study for Micro-CT scanning is known, the optimal segmentation threshold can be determined based on the measured porosity of the rock core for image segmentation.

1) Threshold Segmentation

While quantitative analysis can be directly applied to grayscale images obtained from tomography, Micro-CT is designed to capture geometric shapes with sufficient resolution (Fig 7). Therefore, most analyses commence with segmentation. The term "segmentation" can be ambiguous since, in image processing, it often refers to decomposing an object into several parts. However, in the context of tomography, it is interpreted as the identification of discrete materials in an image, typically achieved by binarizing the image to represent only black and white materials. In absorption-contrast tomography, the value associated with each voxel is proportional to its X-ray attenuation, which is influenced by the density, atomic number, and energy of the incident X-rays.

**Fig 8:**
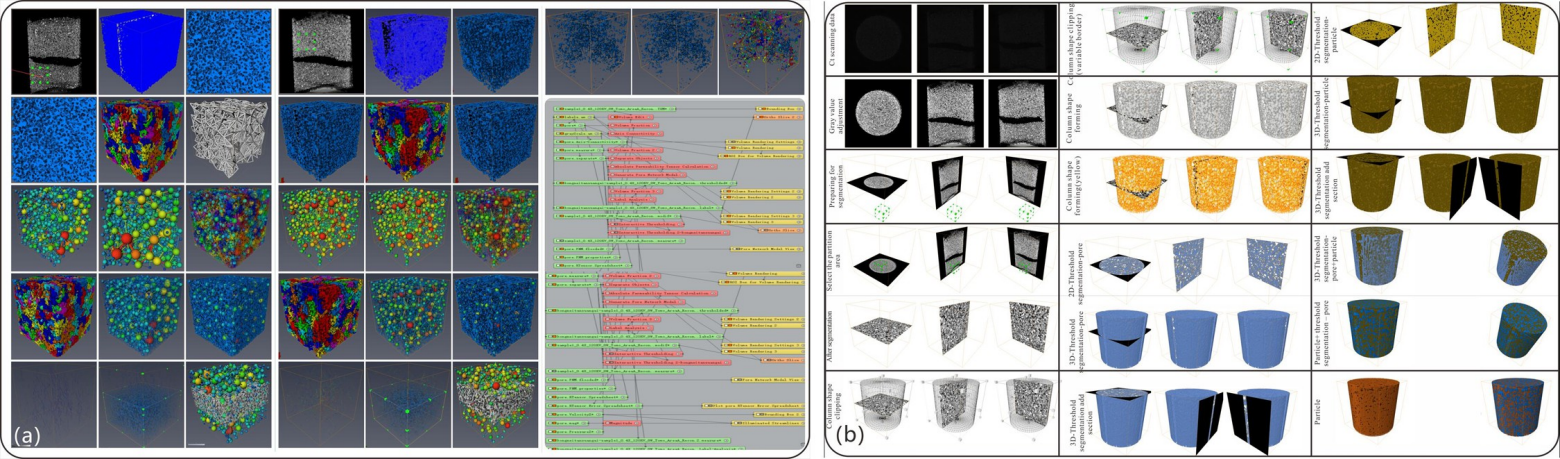
The overall operational steps and 3D reconstruction process views of the pore network give a very clear spatial structure. (a) Micro-CT raw data display and pore network display, segmented images, 2D view of extracted pore network, pore diameter, throat diameter, and coordination number distribution of specimen [68]. (b) Demonstration of 3D volume reconstruction process, including cropping, threshold segmentation, three-phase extraction. entire and sub volume 3D view of pore network to give a very clear spatial structure.

The following formula was used to determine the segmentation threshold *k* constrained by the measured porosity:

$$f(k*)=\min\left\{f(k)=|\phi -\frac{\sum_{j=I_{\min}}^{k}p(i)}{\sum_{i=I_{\min}}^{J_{\max}}p(i)}\right\}$$ (2)

where the gray scale threshold is *k*, the maximum and minimum gray scale values of the image are *I*_*max*_ and *I*_*min*_, the number of voxels with gray scale value *i* is *p(i)*, the voxels with gray scale values lower than the threshold characterize the pores, and the rest represent the skeleton. The final searched k* value was used as the segmentation threshold, and the segmented binary image (Fig 8A) was obtained, in which the black and white colors represent the pore space and skeleton, respectively. Based on the segmentation algorithm, we can use the mathematical morphology algorithm to further refine the image according to practical needs, that is, remove the isolated voxels by an open operation, fill the small holes and connect the neighboring voxels by a closed operation.

Macro-pores and micro-pores are used to obtain micron-scale pore data (original CT images, binary images, PNM (pore network model) and calculation parameters (such as pore size, throat size, coordination number, seepage path, etc.), skeleton structure visualization and other information. Before PNM extraction, it is necessary to process the selected area (denoising and segmentation (ROI) to improve image quality). The maximum sphere algorithm (MBA) extracts PNM from the binary images of the particle phase and pore phase [70]. And applies PNM to CT images to quantify the microscopic structure characteristics (such as pore throat radius and coordination number). The coordination number is a parameter that quantitatively characterizes the connectivity of porous media by counting the number of throats connecting a pore [71]. The spheres and cylinders are respectively represented as the orifice abdomen and the orifice throat. Based on voxel flow simulation [72], the binary CT images were calculated to calculate the absolute permeability of the specimen to characterize its migration behavior.

The pore is transformed into PNM to quickly understand and explore the pore space, realize the visualization of sphere or ellipsoid and rod with attribute mapping, and calculate the pore characteristics and statistical data, such as volume fraction, the maximum sphere fitting of the given pore, pore size distribution, pore throat size distribution, pore orientation, and shape factor. The pore throat size, connection mode, and porosity of the specimen are extracted, and the microstructure of the specimen is obtained, to reveal the pore throat obstruction and the dominant seepage mechanism of the liquid phase, so as to reveal the mechanism of the decrease of hydraulic characteristics of the specimen after cementation2) 3D Volume Reconstruction

Avizo software, a powerful tool for 3D image processing, provides superior functionality, making the 3D reconstruction of sand columns before and after biocementation more accurate and efficient. The theoretical basis for the 3D reconstruction of sand columns relies primarily on non-invasive imaging techniques such as X-ray computed tomography (XCT). This technology allows the acquisition of volumetric data inside the sand column at high resolution, providing robust support for subsequent three-dimensional reconstruction. Avizo, with its robust image processing and analysis capabilities, can effectively handle large volumes of data and generate high-quality 3D reconstruction models.

The first step in the 3D reconstruction of sand columns using Avizo, involves preprocessing the data obtained from the XCT scans. This includes steps such as denoising, filtering, and contrast enhancement to improve the image quality (Fig 8B). Subsequently, using Avizo’s segmentation tools, the sand column is separated from the surrounding soil, creating a clear interface (Fig 8B). Avizo’s volume rendering and surface reconstruction functions are used to create a 3D model of the sand column, accurately reproducing its internal structure (Fig 8). Avizo provides powerful visualization and analysis tools to further explore the physical properties of the sand column. Slicing (Fig 8), projection, and virtual cutting (Fig 8) of the 3D model allow for an in-depth understanding of the internal microstructure and particle distribution of the sand column (Fig 8), which is crucial for analyzing biocemented sand bodies.

In theory, a larger digital rock core offers more accurate characterization of the micro-pore structure and macroscopic properties of the rock. However, larger digital rock cores demand increased computer storage and processing power. Therefore, a compromise solution involves selecting a representative elementary volume (REV). Yin et al. (2017) [73] demonstrated through multiple experiments that when the size of a digital rock core was 200×200×200 voxels, its physical properties (such as porosity and elastic modulus) were almost unaffected by the size. In this study, considering computational storage and speed, 200×200×200 voxels were chosen as the REV. The Marching Cubes algorithm was used to extract a set of triangular facets representing the surface from the 3D data volume of the REV obtained from the image processing results. These facets were then rendered using a lighting model to form a 3D surface image of the rock core, thereby completing the 3D modeling of the digital rock core (Fig 8).

**Fig 9:**
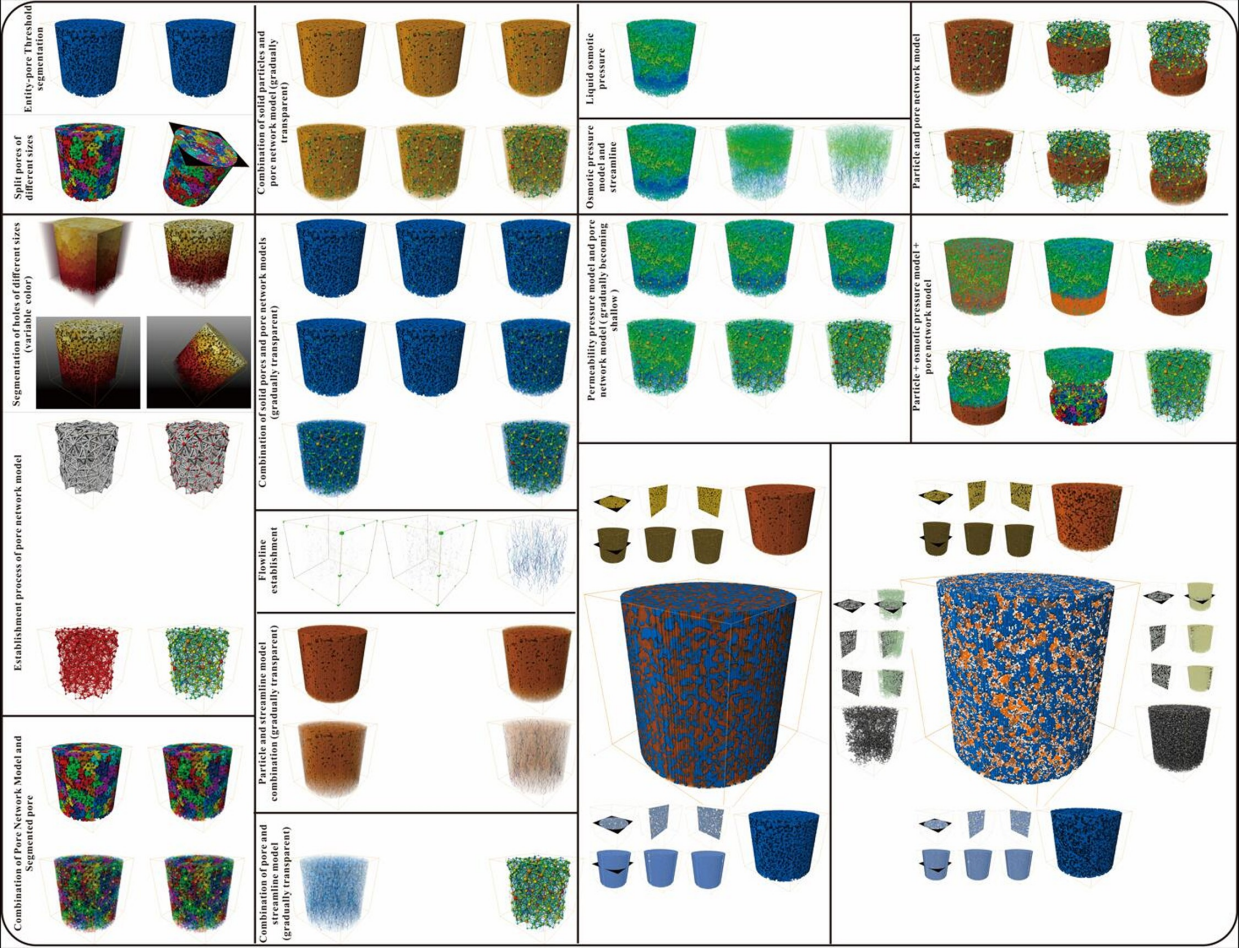
Comparison of three-dimensional reconstruction of samples before and after bio treatment.

In the integrated image analysis platform, we used the interactive threshold algorithm to segment the upper and lower region values based on their different gray values, whereas the volume fraction algorithm was used to quantify the filling ratio. Finally, the segmentation results were rendered using the volume rendering method to extract cementation patterns. Using this comprehensive approach, various types of pores and biocements were detected and classified, and the porous material was analyzed quantitatively, providing valuable information for further research (Fig 9) [80–81].

## 3 Results and analysis

### 3.1 Cementation patterns and area filling rate (2D)

According to the microscopic slices (Fig 6A), five ideal distribution types of CaCO_3_ at the pore-scale of sand were obtained: contact cementation (G-G), particle encapsulation (G-C), matrix support (G-C-G), pore abdomen, and pore throat plugging. The original image was partially blurred owing to the height difference between the sand particles. To avoid a misleading analysis in this area, the original image was stacked [75]. The results can intuitively distinguish three phases (particles, pores, and CaCO_3_) but cannot obtain quantitative data.

**Fig 10:**
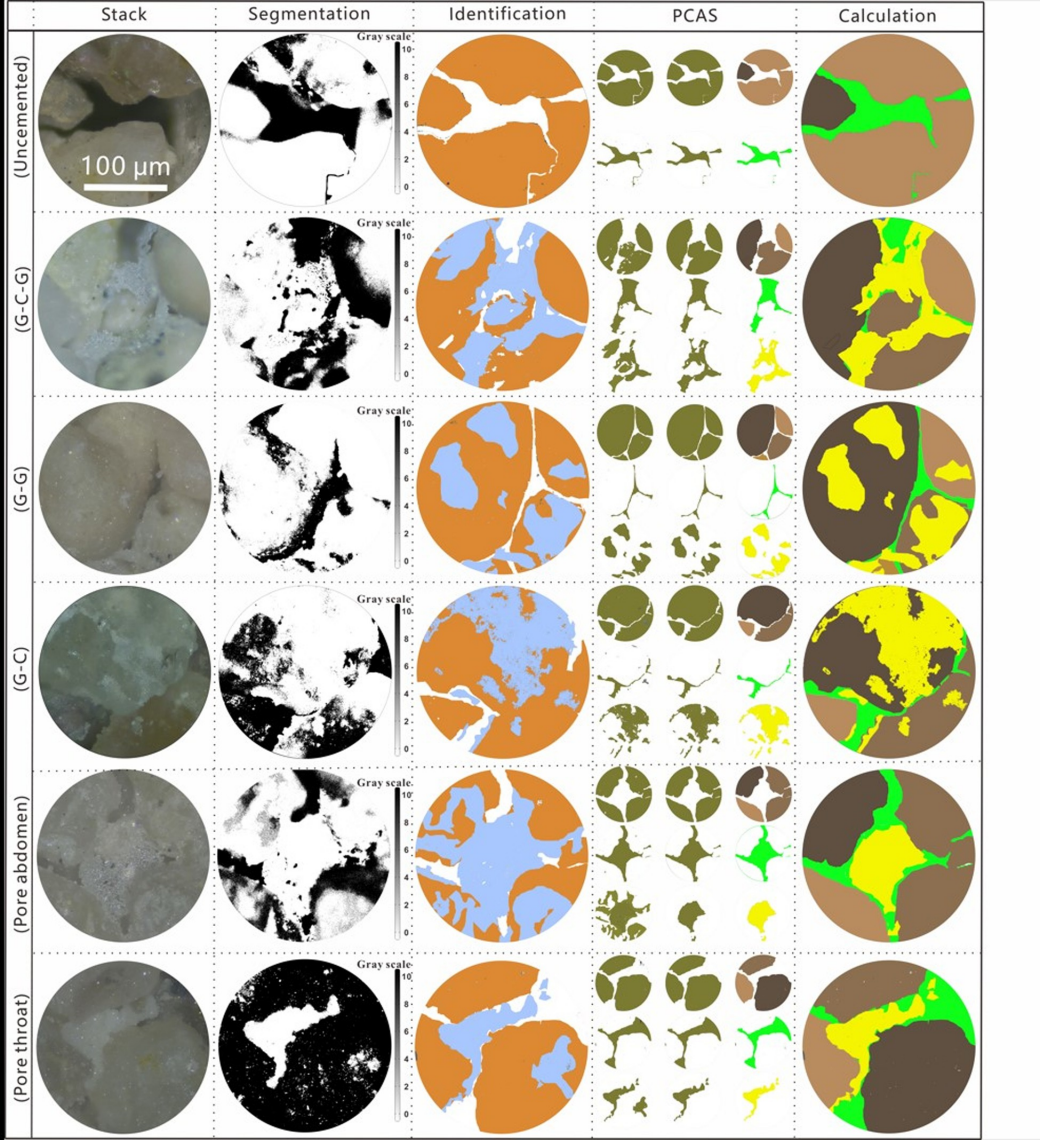
Stack image-based microstructure analysis.

To accurately and quantitatively identify the three phases, the stack image was further processed using threshold segmentation and contour outline filling (Fig 10B and 10C). Subsequently, the PCAS [74] built-in image editor was used to process the clearly identified particles (orange), pores (white), and CaCO_3_ (blue) (Fig 10D), and each area of the three was obtained to calculate the CaCO_3_ (effective) plugging efficiency (Table 4). From top to bottom, the process particles, pores, and CaCO_3_ were respectively processed. According to the analysis of the area data (Table 4) of the specimens, shows that the G-C-G pattern and pore throat plugging efficiency were highest: 68.471% and 64.387%, respectively. At 2.358%, the G-C plugging effect was poor. The PCAS processed images were superimposed (Fig 10E), and an aggregation diagram (green pores, yellow CaCO_3_ particles) was obtained.

**Table 4:** Data extraction from the cementation patterns found in 2D microscope slices.

		Pore area (um^2^)	CaCO_3_ area (um^2^)	Effective CaCO_3_ area (um^2^)	Pore filling rate (%)	Effective sedimentation rate (%)	Filling error (%)	Precipitation error (%)
R	UC	892449.7	0	0	0	0	0	0
	G-C-G	1322752.7	1567827.0	1073499.0	81.156	68.471	5.933%	4.666%
	G- G	318735.5	1927689.9	62869.8	19.720	3.261	3.024%	2.016%
	G-C	497064.5	2461031.0	58026.6	11.674	2.358	5.305%	4.003%
	Pore abdomen	1728083.4	9225785.8	1152809.5	66.710	12.496	4.729%	3.994%
	Pore throat	1433209.5	873628.4	562504.1	39.248	64.387	6.231%	5.061%

Because of biocementation, the flow channel and part of the pore throats between particles were blocked, therefore, the data showed the most obvious low permeability phenomenon [75]. It was concluded that the key pore throats were blocked by a large amount of high-efficiency CaCO_3_ precipitation between the particles; that is, the optimal precipitation amount was used to achieve anti-seepage and water resistance. Therefore, the G-C-G and pore throat sealing patterns were the main patterns in the image. In contrast, inter-particle cementation (G-G and G-C patterns in images), followed by pore throat blockage, which was caused by high strength and weak blockage. The data showed high permeability coefficient and high porosity [75].

Based on the microscopic images of different parts, the characteristics of the hole roar plugging (Fig 10) were explored. It was speculated that the internal cohesion and internal friction angle of the sand columns after treatment significantly increased owing to the effective cementation binding between particles, which improved the strength and stiffness of the sand columns and the anti-seepage characteristics, which play an important role in improving the loose resistance and hydraulic performance of sand columns [76–79].

We employed the image stack approach to enhance the clarity of the acquired microscopic images. We identified three typical cementation patterns present in the biocemented sand: multiple particle encapsulation bridging type (G-C-G), particle layer partial coverage type (G-C), and sporadic filling of the interparticle pores type (specifically, localized cementation at interparticle contact points, G-G). The analysis results indicated that G-C-G exhibited characteristics of multi-particle encapsulation and bridging, with a pore filling rate of 81.2%; G-C featured a particle layer with localized coverage, with a pore filling rate of 19.7%; and G-G manifested as sporadic filling of interparticle pores, with a pore filling rate of 11.7%. G-C-G possessed the optimal cementation effectiveness and impermeability (with an effective sealing rate of 68.5%), whereas G-C (with an effective sealing rate of 2.4%) made a relatively minor contribution to pore filling and flow sealing.

### 3.2 Cementation patterns and volume filling rate (3D)

The qualitative identification and quantitative parameter extraction of the patterns reveal that the presence of biocementation bonds (CaCO_3_) changes the internal structure of the sand specimens treated by MICP, transforming them from a “scattered particles” form (before bio-treatment) of direct contact between particles or particle-particle enclosing the pores in a circle to the “agglomerate particles” form (after treatment) of precipitation-wrapped particles or precipitation filling pores between particles. Further observations demonstrate that the spatial anisotropy of CaCO_3_ distribution, including coating thickness and pore filling ratio surrounding particles, generates different “agglomerate” cementation patterns.

**Fig 11:**
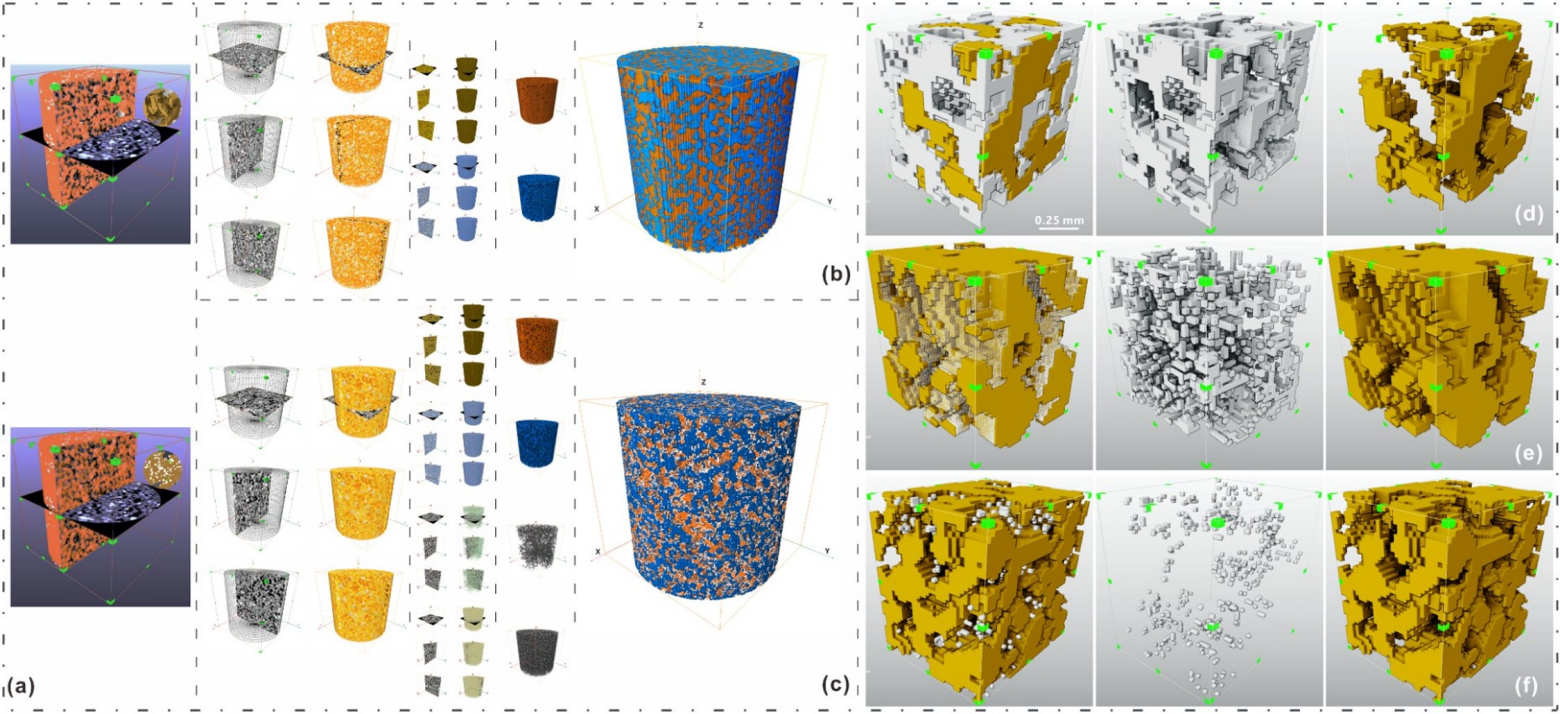
Identification of the cementation patterns of 3D volume reconstructed biocemented sand by capturing the contacts between biocementation bonds and particles. (a) CT scan original image data, (b)-(c) 3D volume reconstruction of sand specimen (Uncemented and cemented, respectively). Among them, sand particles: blue, and biocementation bonds: white, (d)-(f) 3D view of particle cementation patterns of G-C-G, G-C and G-G, respectively. Among them, the patterns are divided into two-component super-positions (left), one-component biocementation bonds (middle), and one-component particles (right).

Consequently, it is essential to identify the cementation patterns and further analyze the pore filling ratio under the corresponding cementation patterns to evaluate the flow sealing efficiency. The 3D reconstruction identification [80,81] results show that the cementation patterns are consistent with those observed under the microscope, that is, the multi-particle encapsulation bridge type (G-C-G), particle layer local coating type (G-C) and intergranular pore scattered filling type (G-G). As shown in Fig 5D–5F, the characterization of the cementation patterns contained three components: particles, pores, and biocementation bonds. The biocementation bonds in the three patterns had different morphologies, expressed as enclosed bridging (middle subgraph of Fig 11D), contiguous stacking (middle subgraph of Fig 11E), and scattered stars (middle subgraph of Fig 11F).

The statistical analysis results of the 3D volume reconstructed biocemented sand presented in Table 2 of SI indicate significant differences in the pore filling ratios among the three patterns: G-C-G, G-G, and G-C, with corresponding values of 83. 9791%, 20.3599%, and 11.5450%, respectively. It is important to note that the different locations and forms of CaCO_3_ precipitation have varying effects on the pore filling ratio and flow sealing efficiency of the biocemented sand. As result, the CaCO_3_ coated on the particle surface contributed less flow sealing, particularly in the flow perpendicular to the sand profile, compared to CaCO_3_ filling the pores between particles.

It is crucial to accurately identify the cementation patterns and analyze the corresponding pore filling ratios to evaluate the flow sealing efficiency. In this study, two types of CaCO_3_ precipitates: total CaCO_3_ and effective seal CaCO_3_, were distinguished and analyzed separately during pattern identification. The results showed significant variations in the pore filling ratios of the three cementation patterns: G-C-G, G-G, and G-C. The highest filling ratios were observed for the G-C-G pattern: 83.979% (area filling ratio) and 77.257% (volume filling ratio) followed by G-G (20.360% and 20.600%) and G-C (11.545% and 11.250%). The microscopic biocementation patterns and pore filling rates captured using the microscope stack images were very similar to the data obtained by 3D reconstruction; for G-C-G, G-G, G-C, the pore filling rates obtained by the two methods differed by 2.7791, 0.66, 0.155, respectively.

### 3.3 Representative elementary volume (REV)

To construct the micro-scale structure and properties, each cube was cut and the CaCO_3_ filling rate was determined. The resulting data, presented in Table 3 of SI, show the area filling rate and overall volumetric filling rate of each layer. The microstructure sizes of the 3D sand specimens reconstructed in this experiment, in the order of G-C-G, G-G, and G-C, were 280 *μ*m × 270 *μ*m × 220 *μ*m, 400 *μ*m × 320 *μ*m × 430 *μ*m and 270 *μ*m × 280 *μ*m × 220 *μ*m, respectively. From Table 3 of SI, their filling rates were 83.979%, 20.360%and 11.545%, respectively.

The filling ratios of the microstructure scales of the three cementation patterns (G-C-G, G-G and G-C) were extended to more microscopic and macroscopic scales to verify that our reconstruction visualization analysis satisfied the REV scale; thus, the average filling ratios of the three patterns were calculated at five scales. By comparing the data in Table 3 of SI, the average filling ratios of the three patterns at the five scales are almost identical (in the 3D data, the difference between the maximum and minimum average filling ratios at the five scales for the three cementation patterns G-C-G, G-G and G-C, are 7.125, 3.085, and 2.641, respectively). Therefore, it was determined that the extraction of cementation patterns conformed to the REV scale; that is, the extraction of microscopic test data supported the model filling rate value at the macro scale. Changing the cementation pattern size had little influence on the pore filling rate, and this process was used to evaluate the REV scale.

### 3.4 Hydraulic barrier and sealing efficiency formed by biocementation

**Fig 12:**
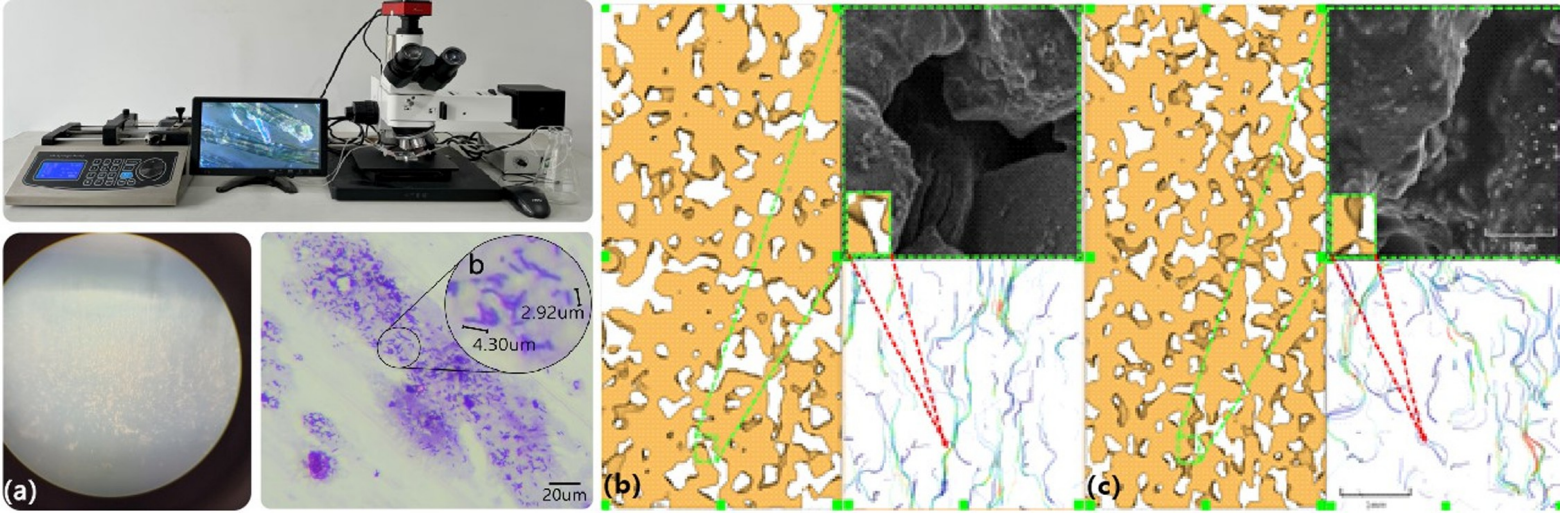
Microscopically visualize biofilms and reproduce the combined water blocking effect of biofilm and cementation by Avizo. (a) Microscopic observation of biofilm staining. (b) SEM images without biofilm, and fluid modeling of pore structure under the action of pure calcium carbonate. (c) SEM images under the combined action of biofilm and calcium carbonate, and fluid simulation of the combined hydraulic barrier of biofilm and calcium carbonate.

The precipitate, together with the accumulated biofilm (EPS), alters the physicochemical properties of the system [3] and substantially changed the microstructure of the porous media. Ebigbo et al. (2010) [82] demonstrated that biofilms could be used as bio-barriers to block leakage pathways by occupying pore space. In addition to the direct effect of mineralized calcium carbonate on the pore microstructure of sand pores, through microscopic staining, SEM electron microscopy scanning, and 3D visualization analysis, we found that biofilm played a role in blocking water during MICP treatment, although they did not contribute much to the modification of the internal structure of sandy soil (Fig 12).

Biofilms are generally considered to be heterogeneous matrices composed of microbial communities and extracellular polymers (EPS, microbial cell metabolites), which are formed by microbial attachment and growth in natural environments and engineered systems. They stabilize spatial organization mainly by providing intercellular scaffolds formed by EPS [83], and are therefore essential for stabilizing sediments [29]. Due to their structural irregularities, biofilms may also have a significant impact on the hydrodynamics of porous media [22].

Comparison of the results of sand porosity and permeability coefficient tests before and after biocementation revealed [72] that the overall decrease in porosity and hydraulic conductivity of sands injected with CS only and those injected with BS only and incubated continuously under suitable conditions differed considerably (weakly in the former and significantly in the latter). Considering the nutrients provided by the latter test to sustain microbial growth and the culture environment suitable for microbial community proliferation and construction of biofilm systems [84], we analyzed that microbial cells and their biofilm systems constructed in conjunction with the EPS acted as positive hydraulic barriers to the permeability of the porous media, and had a certain degree of enhanced potential for microbial mineralization [30].

Comparison of the results shows (Fig 12B and 12C) that the flow lines under the effect of pure calcium carbonate (i.e., no biofilm effect) show uniformity, direction consistency, and insignificant color changes in the pressure cloud diagrams. In contrast, the streamlines under the combined action of biofilm and calcium carbonate showed unevenness, weak directional consistency, and significant color changes in the pressure maps.

Further investigation shows that the sand under the action of pure calcium carbonate is almost isotropic under natural conditions, with uniform distribution of pores, particles and calcium carbonate, and therefore, the pore connectivity is strong. Due to the strong connectivity, the fluid infiltrates uniformly under constant head, which is characterized by a uniform flow rate. In addition, the uniform change in fluid pressure is due to the fact that the pores are not effectively sealed by CaCO_3_, and the infiltration of fluid is not impeded; i.e., the change in pressure is uniform and small (the change in pressure color is not significant, and blue-green performance is dominant). In contrast, the sand under the joint action of biofilm and calcium carbonate is anisotropic, mainly due to the joint action of biofilm and CaCO_3_ in the pore space, especially the biomineralization at the pore throat, and the pore connectivity becomes weaker (the more isolated the pore space is, the smaller the pore allotropic number is, the smaller the pore area is, and the lower the porosity is). That is, under constant head, fluid infiltration is impeded, streamline flow is turbulent and irregular, the fluid range is limited, and the flow velocity increases (red streamlines predominate).

As shown in Fig 12 the hydraulic barrier formed by the combination of the biofilm and CaCO_3_ was more effective than that formed by CaCO_3_ alone.

## 4 Conclusions

In this study, we investigated the application of MICP to loose sandy soils. We analyzed the aggregation patterns of the CaCO_3_ precipitates and inter-particle cementation patterns through microscopic image capture and stack processing to understand the spatial heterogeneity of the pore-scale precipitates. The spatial distribution of CaCO_3_ precipitates and the flow paths of the fluids were reproduced using 3D volume reconstruction, and their effects on the local fluid dynamics were analyzed. In addition, we explored the decreasing feedback between the connecting pores and dominant seepage paths flowing through the biocemented sand. Microscopy and X-CT scanning techniques were used to analyze the cementation patterns produced by MICP in sand. Three cementation patterns (G-C-G, G-C, and G-G) were identified, their spatial distributions and pore-filling ratios were analyzed, and the hydraulic properties of the MICP-treated sands and the microstructures contributing to the seal were evaluated. These experiments verified the positive effects of CaCO_3_ precipitation and biofilm barriers on runner sealing. The main conclusions of this study are as follows.

1. Microscopy and stack treatment analyses showed that the G-C-G pattern exhibited the most effective cementation state, with an effective water sealing efficiency of 68.5%, whereas the G-C pattern had the poorest effective sealing rate of 2.4%.
2. 3D reconstruction confirmed the presence of the G-C-G, G-C, and G-G cementation patterns and showed that the filling rate of the G-C-G pattern was the highest, followed by G-G and G-C.
3. REV scale was used to analyze the filling rate of each size and it was demonstrated that the microstructure of the cementation patterns determined their filling rate, with little variation between sizes.
4. SEM image data and Avizo streamline analysis indicated that the hydraulic barrier formed by combining the biofilm and CaCO_3_ was better than that formed by CaCO_3_ alone.

Overall, these findings suggest that the G-C-G cementation pattern is the most effective for improving anti-seepage and directional cementation using MICP technology.

## Supporting information

S1 Data Three-dimensional reconstruction of porosity, permeability coefficient, pore throat coordination number and other data. (ZIP)

## Abbreviations

MICP: is the microbially induced calcium carbonate precipitation
OD_600_: is the optical density of the bacterial
BS: is the bacterial suspension
CS: is the cementation solution
CCC: is the calcium carbonate content
G-C-G: is the Grain-CaCO_3_-Grain pattern
G-G: is the Grain -Grain pattern
G-C: is the Grain-CaCO_3_ pattern
