# Supplementary material for: Identification and extraction of cementation patterns in sand modified by MICP: New insights at the pore scale
Source: PLoS One. 2024 Mar 21;19(3):e0296437. doi: 10.1371/journal.pone.0296437 (PMC10956867; doi:10.1371/journal.pone.0296437)
Supplement: S1 Data — (ZIP) [file pone.0296437.s001.zip › SI-Data/Data aggregation/Tables.docx]

**Table 1 Data extraction from the cementation patterns found in 2D microscope slices.**

| R |  | Pore area  (um^2^) | CaCO_3_ area  (um^2^) | Effective CaCO_3_ area  (um^2^) | Pore filling rate  (%) | Effective sedimentation rate  (%) | Filling error  (%) | Precipitation error  (%) |
| --- | --- | --- | --- | --- | --- | --- | --- | --- |
|  | UC | 892449.7 | 0 | 0 | 0 | 0 | 0 | 0 |
|  | G-C-G | 1322752.7 | 1567827.0 | 1073499.0 | 81.156 | 68.471 | 5.933% | 4.666% |
|  | G- G | 318735.5 | 1927689.9 | 62869.8 | 19.720 | 3.261 | 3.024% | 2.016% |
|  | G-C | 497064.5 | 2461031.0 | 58026.6 | 11.674 | 2.358 | 5.305% | 4.003% |
|  | Pore abdomen | 1728083.4 | 9225785.8 | 1152809.5 | 66.710 | 12.496 | 4.729% | 3.994% |
|  | Pore throat | 1433209.5 | 873628.4 | 562504.1 | 39.248 | 64.387 | 6.231% | 5.061% |

**Table 2 Volume data extraction from the identified cementation patterns in 3D reconstructed sand.**

| Slice layer no. | | | Area proportion (%) | | | | | | | | | | | | | | | | | | | | | |
| --- | --- | --- | --- | --- | --- | --- | --- | --- | --- | --- | --- | --- | --- | --- | --- | --- | --- | --- | --- | --- | --- | --- | --- | --- |
|  |  |  | G-C-G pattern | | | | | | | G-G pattern | | | | | | | | G-C pattern | | | | | | |
|  |  |  | Total CaCO_3_ | Effective seal CaCO_3_ | | Pore | | Pore filling ratio | | Total CaCO_3_ | | Effective seal CaCO_3_ | | Pore | | Pore filling ratio | | Total CaCO_3_ | | Effective seal CaCO_3_ | | Pore | | Pore filling ratio |
| 1 | | | 41.793 | 37.02 | | 59.606 | | 62.107 | | 7.142 | | 5.913 | | 42.477 | | 13.921 | | 21.063 | | 4.875 | | 68.847 | | 9.986 |
| 2 | | | 40.007 | 35.621 | | 55.296 | | 66.227 | | 18.362 | | 13.304 | | 39.694 | | 33.516 | | 51.878 | | 4.756 | | 63.496 | | 10.64 |
| 4 | | | 45.577 | 41.054 | | 60.961 | | 68.986 | | 11.530 | | 7.391 | | 46.433 | | 15.918 | | 27.448 | | 3.329 | | 56.362 | | 7.681 |
| 6 | | | 45.191 | 41.887 | | 68.966 | | 60.736 | | 8.021 | | 6.652 | | 38.694 | | 17.191 | | 25.212 | | 3.924 | | 59.691 | | 8.249 |
| 7 | | | 44.014 | 41.764 | | 66.749 | | 65.564 | | 8.428 | | 4.435 | | 36.303 | | 12.217 | | 20.645 | | 5.47 | | 58.502 | | 11.059 |
| 8 | | | 44.465 | 40.655 | | 66.01 | | 66.134 | | 10.019 | | 5.174 | | 22.956 | | 22.539 | | 32.558 | | 5.351 | | 64.566 | | 11.385 |
| 10 | | | 47.313 | 45.828 | | 61.453 | | 81.082 | | 9.874 | | 6.652 | | 32.129 | | 20.704 | | 30.578 | | 3.448 | | 61.95 | | 8.794 |
| 12 | | | 56.430 | 54.877 | | 57.636 | | 91.743 | | 6.947 | | 3.696 | | 26.173 | | 14.121 | | 21.068 | | 5.351 | | 60.88 | | 12.075 |
| 15 | | | 49.753 | 48.581 | | 57.02 | | 81.693 | | 6.793 | | 6.652 | | 18.999 | | 35.012 | | 41.805 | | 5.826 | | 51.367 | | 15.235 |
| 17 | | | 54.883 | 52.645 | | 55.296 | | 93.399 | | 10.794 | | 7.391 | | 36.086 | | 20.482 | | 31.276 | | 3.686 | | 34.958 | | 16.265 |
| 18 | | | 56.663 | 55.37 | | 53.571 | | 101.49 | | 8.205 | | 3.696 | | 16.782 | | 22.024 | | 30.229 | | 4.875 | | 37.337 | | 15.735 |
| 20 | | | 52.389 | 49.305 | | 51.724 | | 95.324 | | 6.527 | | 5.174 | | 36.086 | | 14.338 | | 20.865 | | 4.756 | | 52.675 | | 10.927 |
| 22 | | | 58.542 | 57.172 | | 51.601 | | 108.859 | | 6.896 | | 5.174 | | 20.304 | | 25.483 | | 32.379 | | 6.302 | | 75.268 | | 9.701 |
| Average value | | | / | | | | | 83.9791 | | / | | | | | | 20.3599 | | / | | | | | | 11.545 |
| Volume proportion (%) | | | | | | | | | | | | | | | | | | | | | | | | |
|  | G-C-G pattern | | | | | | | | G-G pattern | | | | | | | | G-C pattern | | | | | | | |
| Max | | 74.754 | | 62.192 | 68.9 | | 90.179 | | 22.690 | | 17 | | 61.4 | | 27.700 | | 29.556 | | 6.726 | | 57.814 | | 11.634 | |
| Min | | 43.103 | | 32.020 | 49.6 | | 64.516 | | 9.313 | | 0 | | 13.3 | | 0.000 | | 18.787 | | 4.162 | | 35.672 | | 11.667 | |
| Mean | | 54.621 | | 44.763 | 57.9 | | 77.257 | | 14.456 | | 7 | | 29.7 | | 23.600 | | 30.430 | | 6.652 | | 59.132 | | 11.250 | |

**Table 3** **Evaluation of the fluctuation of microscale parameters extracted from a 3D volume reconstruction model with the size of sampling domain.**

| Slice layer |  | G-C-G pattern | | | | | | G-G pattern | | | | | | G-C pattern | | | | | |
| --- | --- | --- | --- | --- | --- | --- | --- | --- | --- | --- | --- | --- | --- | --- | --- | --- | --- | --- | --- |
|  | Slice layer no. | Sampling domain (um×um×um) | | | | | Standard deviation | Sampling domain (um×um×um) | | | | | Standard deviation | Sampling domain (um×um×um) | | | | | Standard deviation |
|  |  | 180×170×120 | 210×230×190 | 280×270×220 | 330×300×280 | 360×380×340 |  | 280×250×320 | 300×260×360 | 400×320×430 | 420×360×460 | 510×430×510 |  | 170×180×120 | 220×230×170 | 270×280×220 | 320×330×270 | 370×380×320 |  |
|  |  | 3672000 | 9177000 | 16632000 | 27720000 | 46512000 |  | 22400000 | 28080000 | 55040000 | 69552000 | 111843000 |  | 3672000 | 8602000 | 16632000 | 28512000 | 44992000 |  |
|  |  | Pore filling ratio | | | | |  | Pore filling ratio | | | | |  | Pore filling ratio | | | | |  |
|  | 1 | 58.454 | 57.907 | 62.107 | 57.967 | 60.753 | 1.893 | 13.102 | 15.313 | 13.921 | 12.993 | 15.187 | 1.108 | 9.399 | 10.985 | 9.986 | 9.32 | 10.894 | 0.795 |
|  | 2 | 62.331 | 60.462 | 66.227 | 61.812 | 65.248 | 2.426 | 31.544 | 36.868 | 33.516 | 31.282 | 36.563 | 2.666 | 10.014 | 11.704 | 10.640 | 9.931 | 11.607 | 0.846 |
|  | 3 | 63.147 | 62.289 | 67.094 | 62.621 | 66.193 | 2.212 | 32.820 | 38.358 | 34.871 | 32.546 | 38.041 | 2.774 | 8.652 | 10.112 | 9.193 | 8.58 | 10.029 | 0.731 |
|  | 4 | 64.928 | 64.211 | 68.986 | 64.387 | 68.257 | 2.283 | 14.982 | 17.510 | 15.918 | 14.857 | 17.365 | 1.266 | 7.229 | 8.449 | 7.681 | 7.169 | 8.379 | 0.611 |
|  | 5 | 60.984 | 64.581 | 64.795 | 60.475 | 63.685 | 2.036 | 19.999 | 23.374 | 21.249 | 19.832 | 23.181 | 1.691 | 10.046 | 11.741 | 10.674 | 9.962 | 11.644 | 0.849 |
|  | 6 | 57.163 | 57.512 | 60.736 | 56.687 | 59.257 | 1.685 | 16.180 | 18.91 | 17.191 | 16.045 | 18.754 | 1.368 | 7.764 | 9.074 | 8.249 | 7.699 | 8.999 | 0.656 |
|  | 7 | 61.707 | 66.847 | 65.564 | 61.193 | 64.524 | 2.447 | 11.498 | 13.439 | 12.217 | 11.403 | 13.328 | 0.972 | 10.408 | 12.165 | 11.059 | 10.322 | 12.064 | 0.880 |
|  | 8 | 62.244 | 61.601 | 66.134 | 61.725 | 65.146 | 2.115 | 21.213 | 24.793 | 22.539 | 21.036 | 24.588 | 1.793 | 10.715 | 12.524 | 11.385 | 10.626 | 12.420 | 0.906 |
|  | 9 | 62.669 | 66.128 | 66.586 | 62.147 | 65.639 | 2.068 | 16.440 | 19.215 | 17.468 | 16.303 | 19.056 | 1.390 | 8.612 | 10.065 | 9.150 | 8.540 | 9.982 | 0.728 |
|  | 10 | 76.312 | 82.574 | 81.082 | 75.677 | 81.453 | 3.182 | 19.486 | 22.774 | 20.704 | 19.324 | 22.586 | 1.647 | 8.277 | 9.673 | 8.794 | 8.208 | 9.593 | 0.699 |
|  | 11 | 84.652 | 93.218 | 89.943 | 83.947 | 91.120 | 4.084 | 43.837 | 51.235 | 46.577 | 43.472 | 50.811 | 3.705 | 8.271 | 9.667 | 8.788 | 8.202 | 9.587 | 0.699 |
|  | 12 | 86.346 | 93.183 | 91.743 | 85.627 | 93.083 | 3.713 | 13.290 | 15.533 | 14.121 | 13.180 | 15.405 | 1.123 | 11.365 | 13.283 | 12.075 | 11.270 | 13.173 | 0.961 |
|  | 13 | 87.002 | 91.881 | 92.440 | 86.277 | 93.844 | 3.417 | 12.560 | 14.680 | 13.345 | 12.455 | 14.558 | 1.062 | 16.040 | 18.746 | 17.042 | 15.906 | 18.591 | 1.355 |
|  | 14 | 83.058 | 91.488 | 88.249 | 82.366 | 89.272 | 3.994 | 13.515 | 15.796 | 14.360 | 13.403 | 15.665 | 1.142 | 15.216 | 17.784 | 16.167 | 15.089 | 17.637 | 1.286 |
|  | 15 | 76.888 | 79.300 | 81.693 | 76.247 | 82.120 | 2.684 | 32.952 | 38.513 | 35.012 | 32.678 | 38.195 | 2.785 | 14.339 | 16.759 | 15.235 | 14.219 | 16.620 | 1.212 |
|  | 16 | 83.551 | 85.050 | 88.773 | 82.855 | 89.843 | 3.132 | 24.883 | 29.082 | 26.438 | 24.675 | 28.841 | 2.103 | 12.428 | 14.526 | 13.205 | 12.325 | 14.405 | 1.050 |
|  | 17 | 87.905 | 94.201 | 93.399 | 87.172 | 94.890 | 3.676 | 19.277 | 22.530 | 20.482 | 19.117 | 22.344 | 1.629 | 15.308 | 17.892 | 16.265 | 15.181 | 17.744 | 1.294 |
|  | 18 | 95.520 | 106.358 | 101.49 | 94.724 | 103.716 | 5.092 | 20.728 | 24.226 | 22.024 | 20.556 | 24.026 | 1.752 | 14.809 | 17.309 | 15.735 | 14.686 | 17.165 | 1.252 |
|  | 19 | 99.629 | 108.103 | 105.856 | 98.799 | 108.479 | 4.646 | 3.970 | 4.640 | 4.218 | 3.937 | 4.601 | 0.335 | 13.721 | 16.037 | 14.579 | 13.607 | 15.904 | 1.160 |
|  | 20 | 89.717 | 95.143 | 95.324 | 88.969 | 96.990 | 3.629 | 13.495 | 15.772 | 14.338 | 13.382 | 15.641 | 1.140 | 10.284 | 12.020 | 10.927 | 10.199 | 11.920 | 0.869 |
|  | 21 | 88.453 | 93.433 | 93.981 | 87.716 | 95.525 | 3.506 | 20.974 | 24.514 | 22.285 | 20.799 | 24.311 | 1.773 | 10.156 | 11.870 | 10.791 | 10.072 | 11.772 | 0.858 |
|  | 22 | 102.456 | 112.227 | 108.859 | 101.602 | 111.755 | 5.061 | 23.984 | 28.031 | 25.483 | 23.784 | 27.800 | 2.027 | 9.130 | 10.671 | 9.701 | 9.054 | 10.583 | 0.772 |
|  | 23 | 122.782 | 136.178 | 130.456 | 121.759 | 135.316 | 6.786 | 24.000 | 29.023 | 0.000 | 23.014 | 27.920 | 11.896 | 7.729 | 9.033 | 8.212 | 7.665 | 8.959 | 0.653 |
|  | Average value | 79.039 | 83.647 | 83.979 | 78.380 | 84.613 | 3.294 | 20.206 | 23.658 | 20.360 | 20.003 | 23.425 | 2.137 | 10.866 | 12.699 | 11.545 | 10.775 | 12.594 | 0.918 |
|  | Max-min | 6.233 | | | | | / | 3.655 | | | | | / | 1.924 | | | | | / |
|  | Pattern  average value | 85.078 | | | | | / | 21.564 | | | | | / | 11.696 | | | | | / |
| 3D | Max | 85.129 | 83.949 | 90.179 | 87.643 | 100.999 |  | 26.859 | 30.107 | 27.700 | 25.528 | 31.070 |  | 8.715 | 12.122 | 11.634 | 11.310 | 12.128 |  |
|  | Min | 58.123 | 60.130 | 64.516 | 59.991 | 73.073 |  | 0.502 | 4.280 | 0.000 | 1.234 | 0.455 |  | 9.831 | 13.351 | 11.667 | 10.189 | 12.745 |  |
|  | Median | 72.675 | 72.474 | 77.257 | 66.790 | 91.215 |  | 20.767 | 24.908 | 21.300 | 20.088 | 23.880 |  | 10.917 | 12.187 | 11.579 | 10.033 | 13.055 |  |
|  | Mean | 79.700 | 83.119 | 83.499 | 78.090 | 85.215 |  | 23.482 | 23.869 | 23.600 | 22.907 | 25.992 |  | 10.405 | 13.046 | 11.250 | 10.808 | 11.854 |  |
|  | Pattern  average value | 81.925 | | | | |  | 23.970 | | | | |  | 11.273 | | | | |  |
