# Supplementary figures and images for: Identification and extraction of cementation patterns in sand modified by MICP: New insights at the pore scale (part 2 of 2)
Source: PLoS One. 2024 Mar 21;19(3):e0296437. doi: 10.1371/journal.pone.0296437 (PMC10956867; doi:10.1371/journal.pone.0296437)

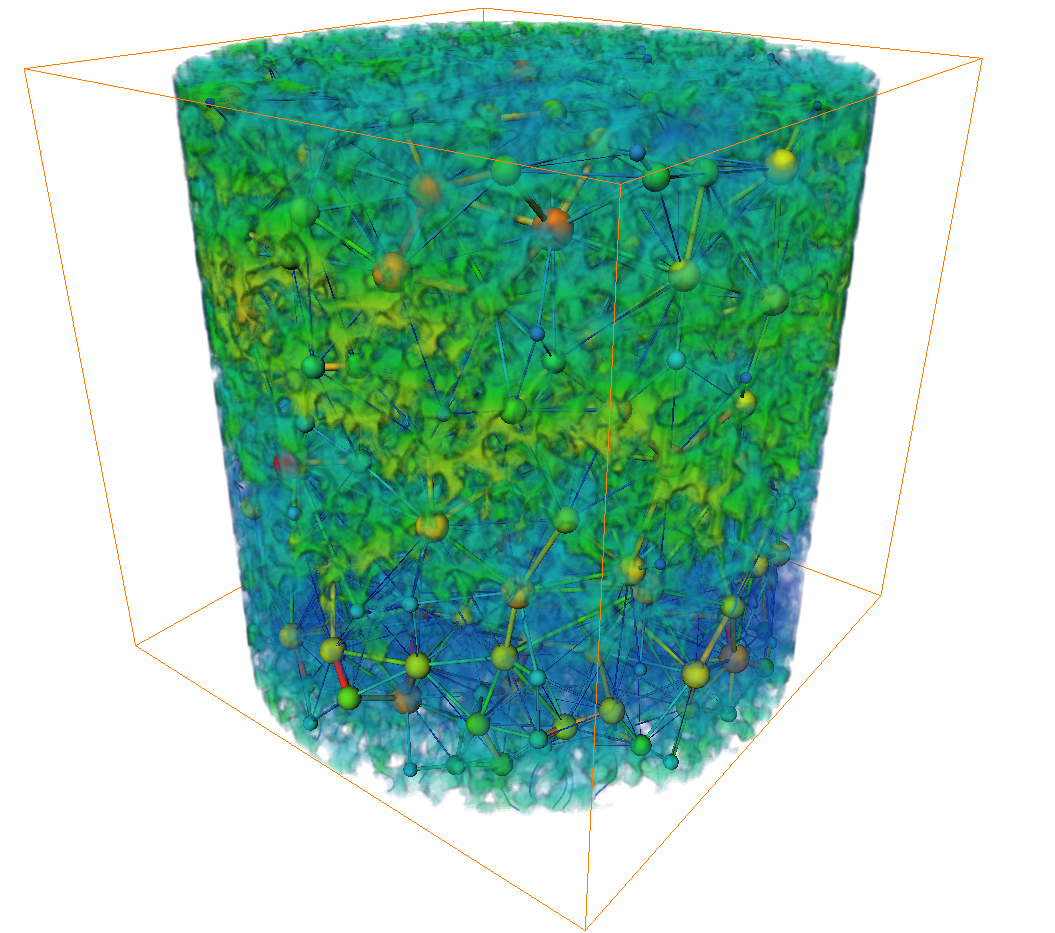

Supplement: S1 Data — (ZIP) [file pone.0296437.s001.zip › SI-Data/Data aggregation/unbiomineralization sample/3D unbiomineralization sample/26-yali jia liuxian 9 .png]

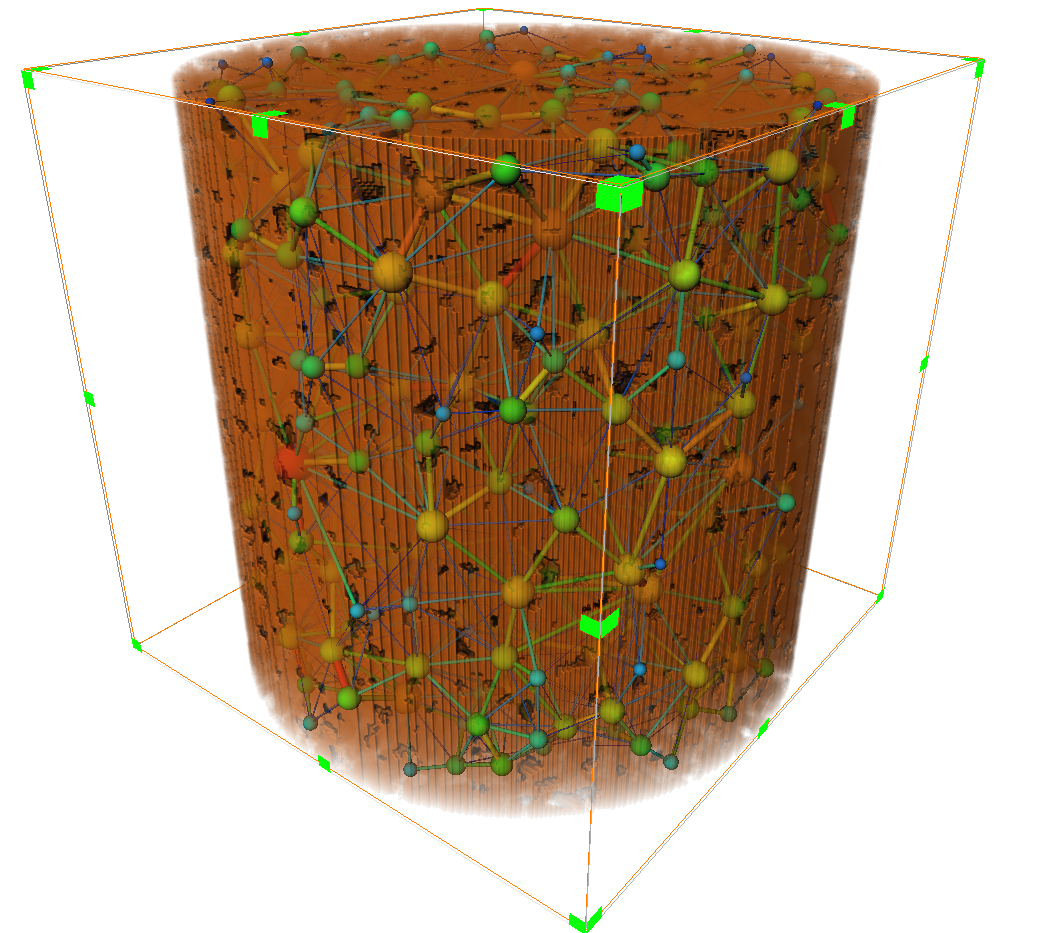

Supplement: S1 Data — (ZIP) [file pone.0296437.s001.zip › SI-Data/Data aggregation/unbiomineralization sample/3D unbiomineralization sample/27 keli yu kongxi wanguo moxing .png]

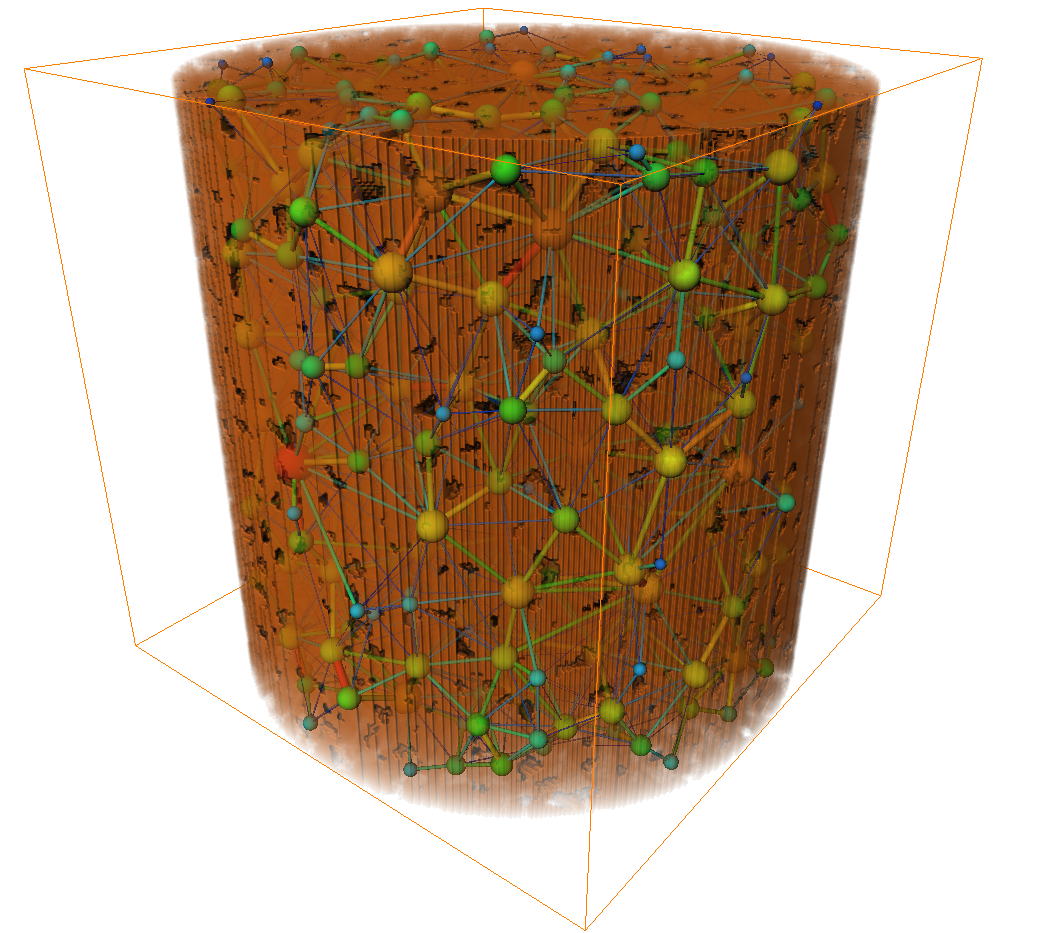

Supplement: S1 Data — (ZIP) [file pone.0296437.s001.zip › SI-Data/Data aggregation/unbiomineralization sample/3D unbiomineralization sample/27 keli yu kongxi wanguo moxing 1 .png]

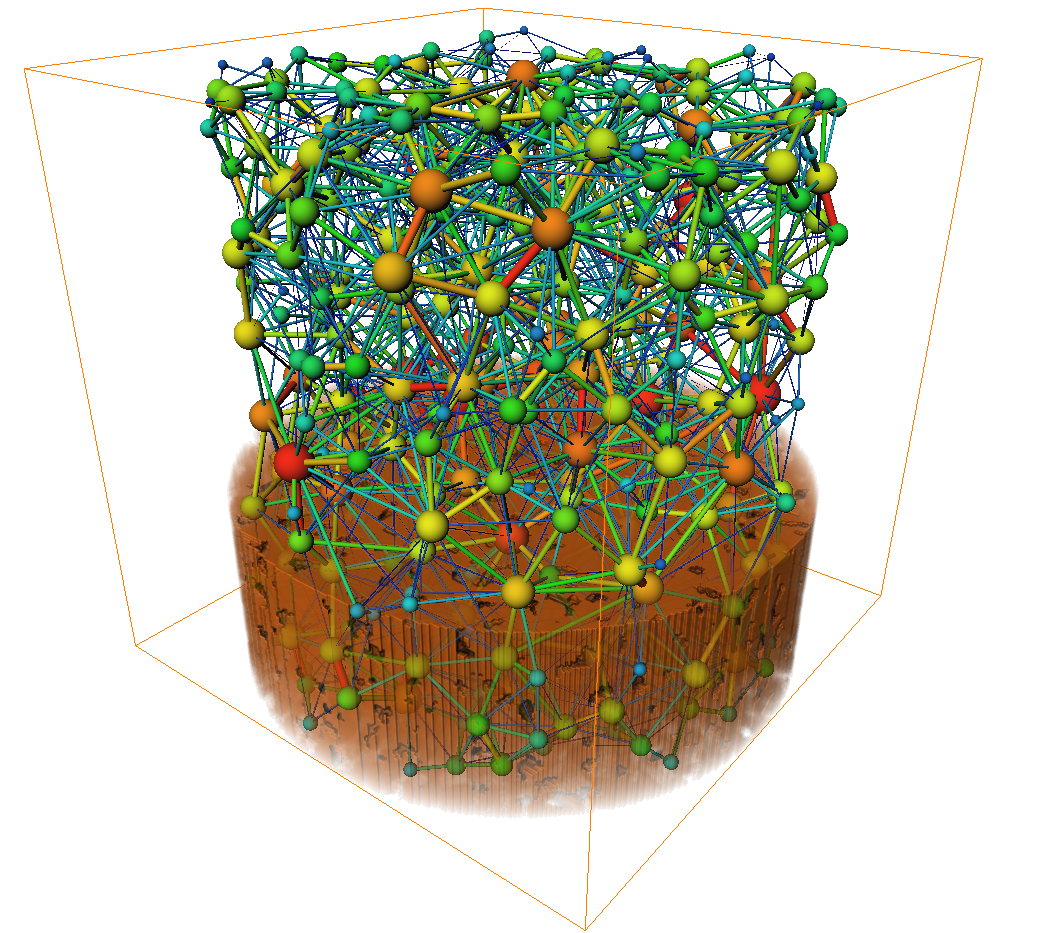

Supplement: S1 Data — (ZIP) [file pone.0296437.s001.zip › SI-Data/Data aggregation/unbiomineralization sample/3D unbiomineralization sample/27 keli yu kongxi wanguo moxing 2 .png]

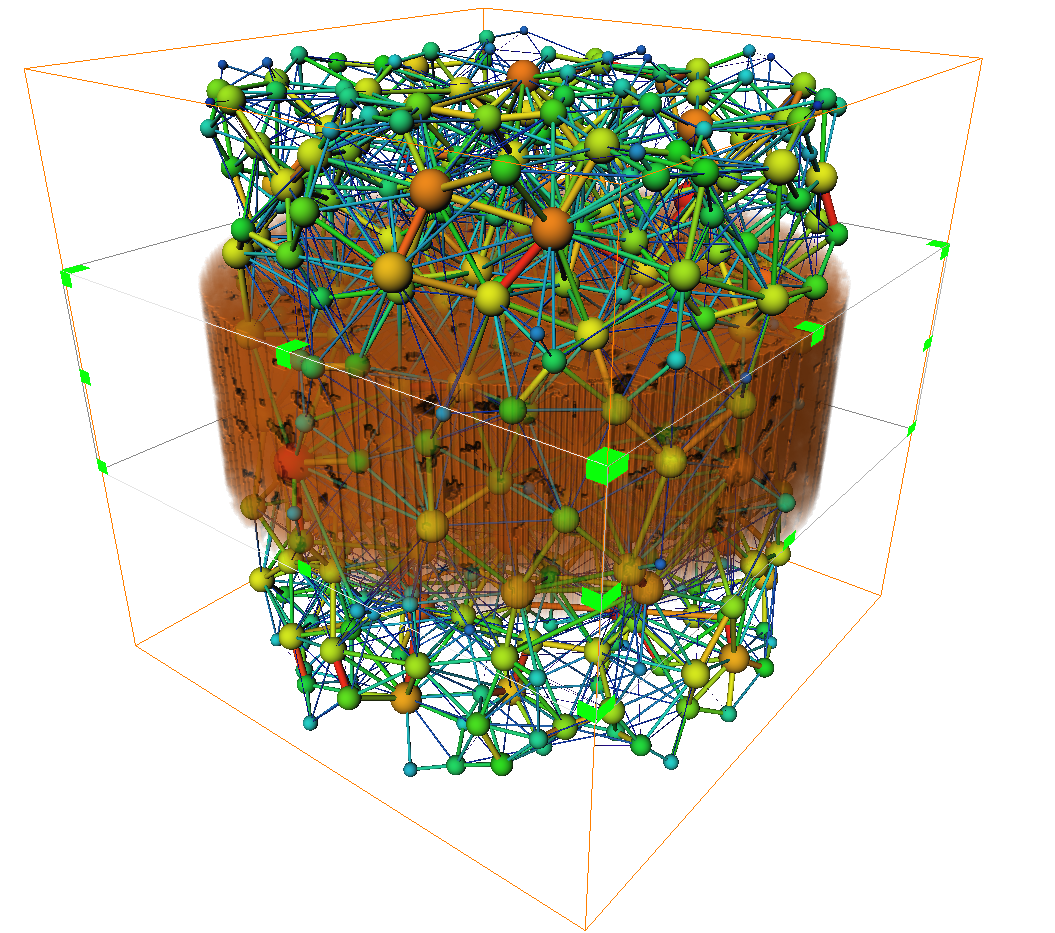

Supplement: S1 Data — (ZIP) [file pone.0296437.s001.zip › SI-Data/Data aggregation/unbiomineralization sample/3D unbiomineralization sample/27 keli yu kongxi wanguo moxing 4 .png]

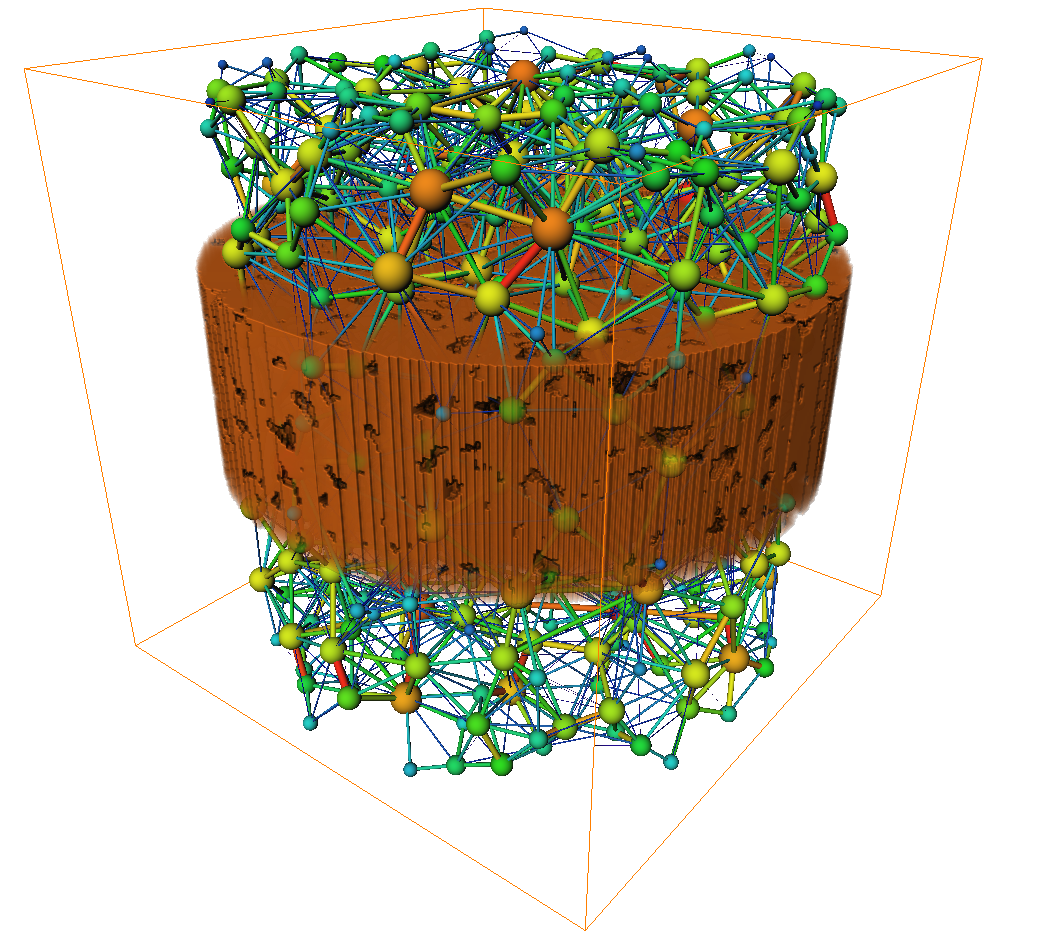

Supplement: S1 Data — (ZIP) [file pone.0296437.s001.zip › SI-Data/Data aggregation/unbiomineralization sample/3D unbiomineralization sample/27 keli yu kongxi wanguo moxing 6 .png]

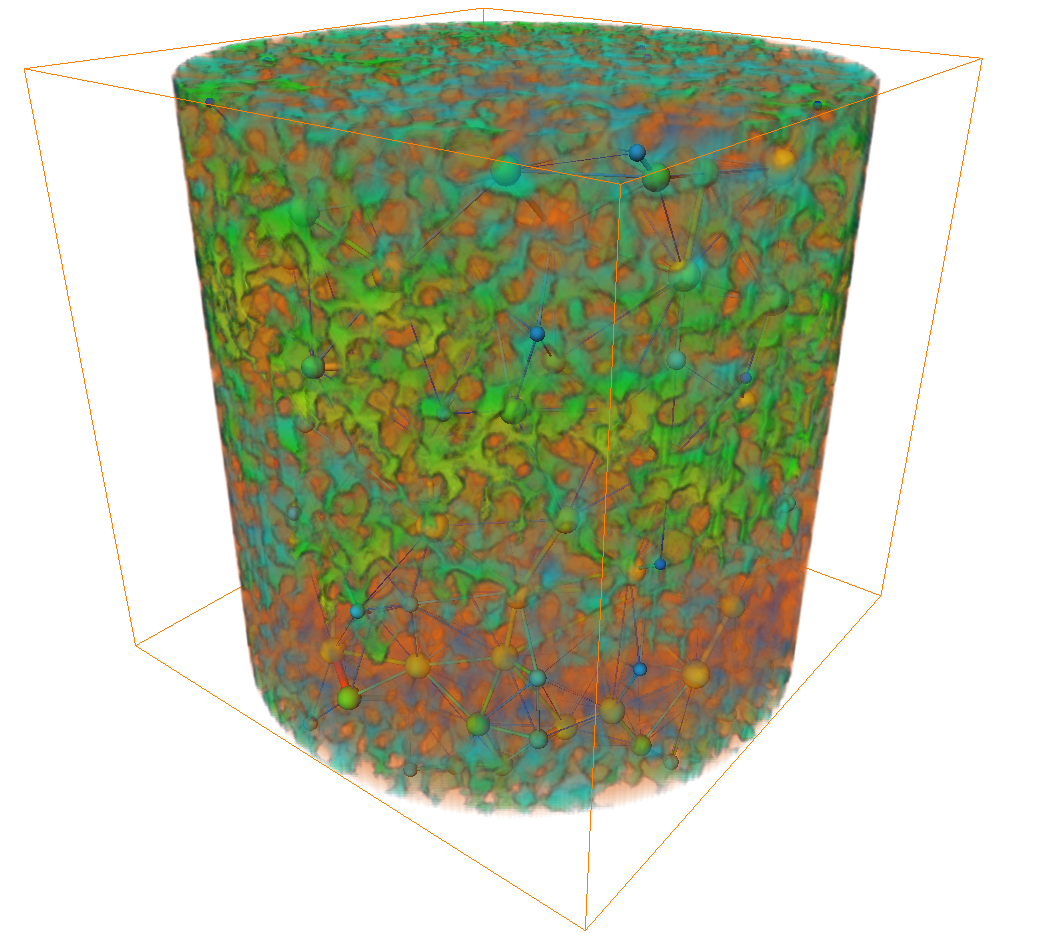

Supplement: S1 Data — (ZIP) [file pone.0296437.s001.zip › SI-Data/Data aggregation/unbiomineralization sample/3D unbiomineralization sample/27 keli,kongxi wanguo moxing,qiugunmoxing, 7 .png]

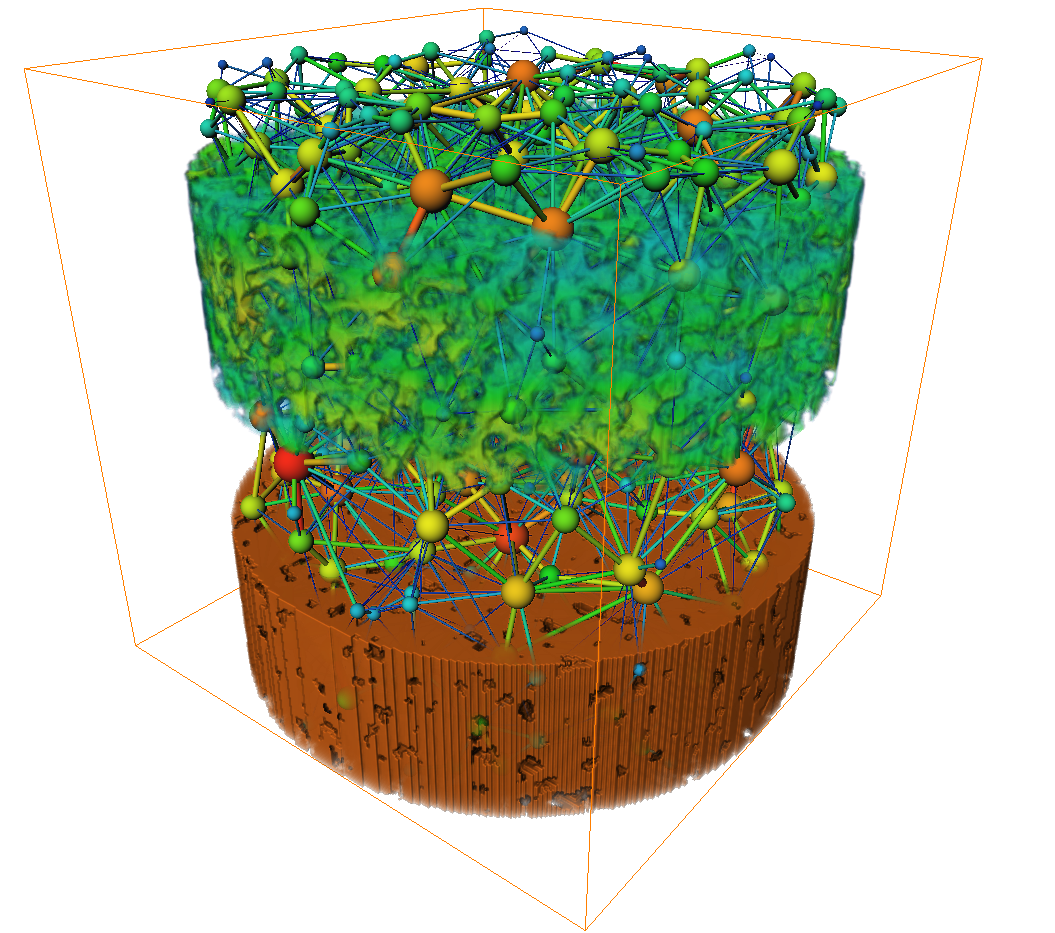

Supplement: S1 Data — (ZIP) [file pone.0296437.s001.zip › SI-Data/Data aggregation/unbiomineralization sample/3D unbiomineralization sample/27 keli,kongxi wanguo moxing,qiugunmoxing,4.png]

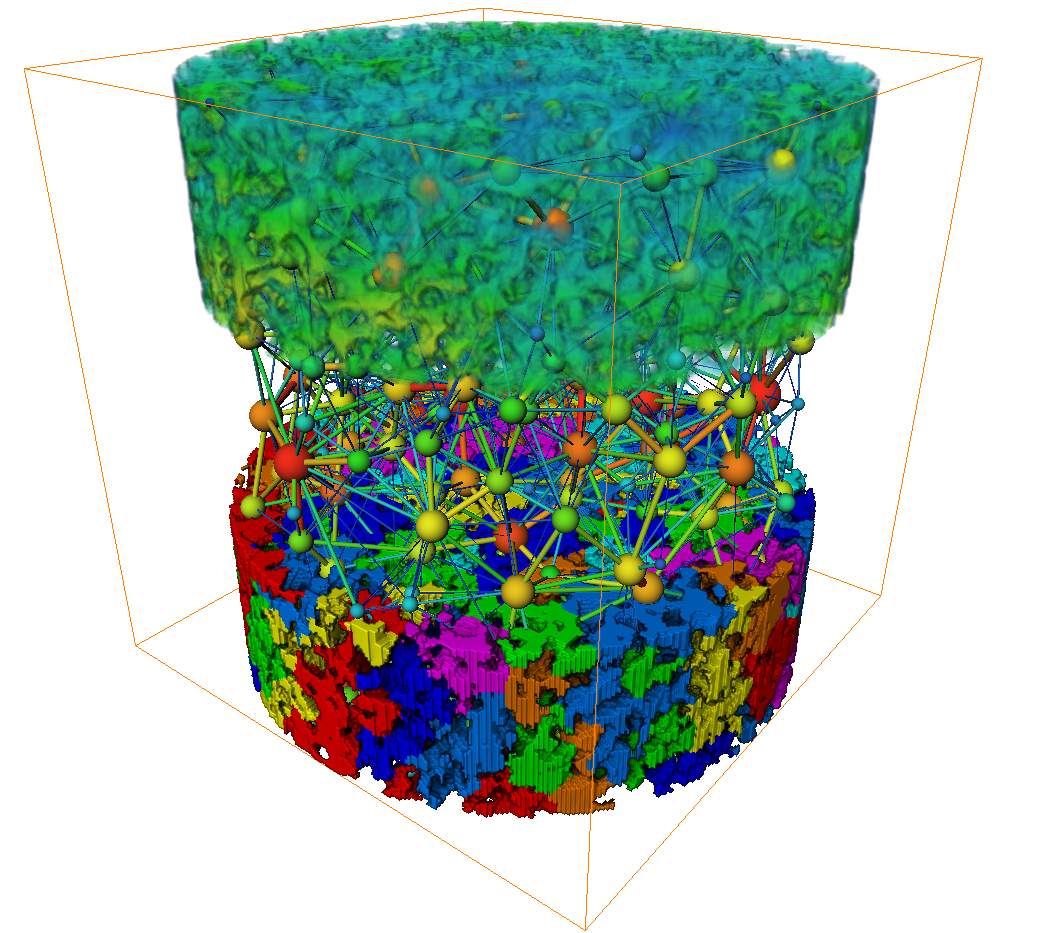

Supplement: S1 Data — (ZIP) [file pone.0296437.s001.zip › SI-Data/Data aggregation/unbiomineralization sample/3D unbiomineralization sample/27 keli,kongxi wanguo moxing,qiugunmoxing,5.png]

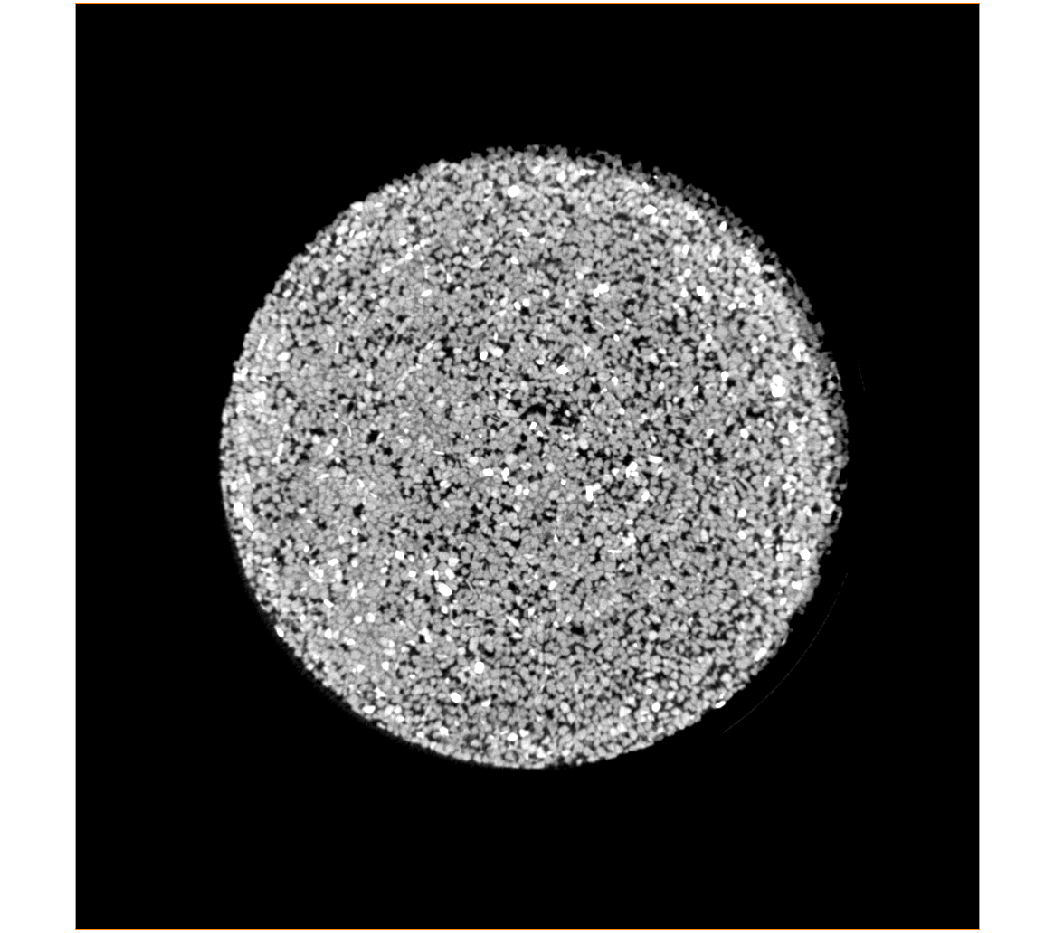

Supplement: S1 Data — (ZIP) [file pone.0296437.s001.zip › SI-Data/Data aggregation/unbiomineralization sample/3D unbiomineralization sample/4-xy.png]

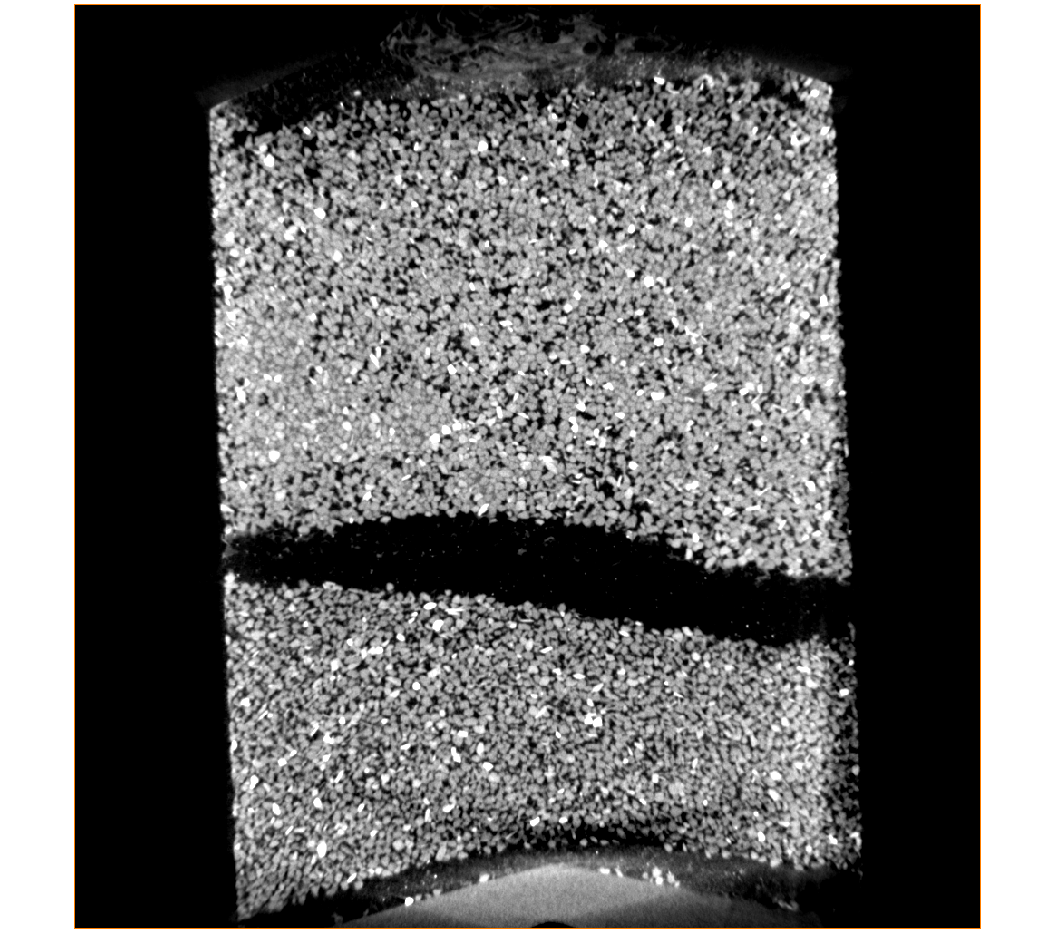

Supplement: S1 Data — (ZIP) [file pone.0296437.s001.zip › SI-Data/Data aggregation/unbiomineralization sample/3D unbiomineralization sample/4-xz.png]

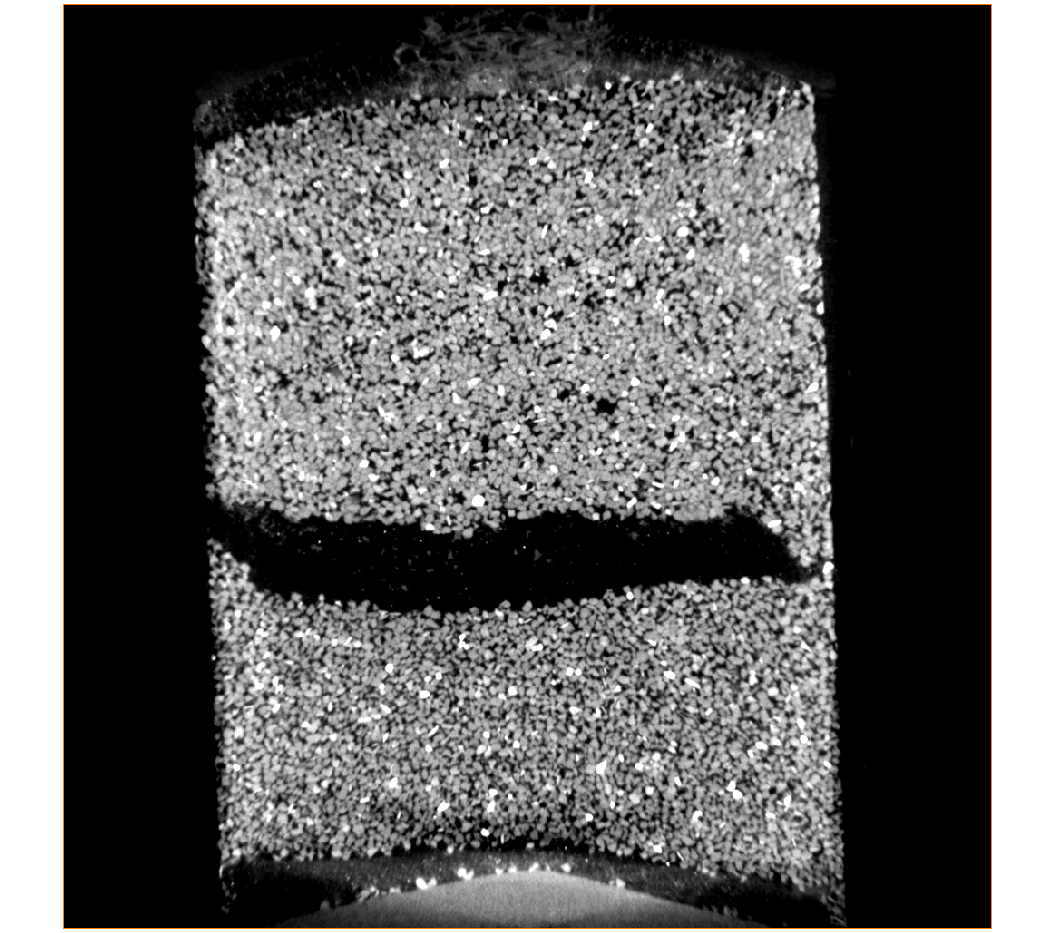

Supplement: S1 Data — (ZIP) [file pone.0296437.s001.zip › SI-Data/Data aggregation/unbiomineralization sample/3D unbiomineralization sample/4-yz.png]

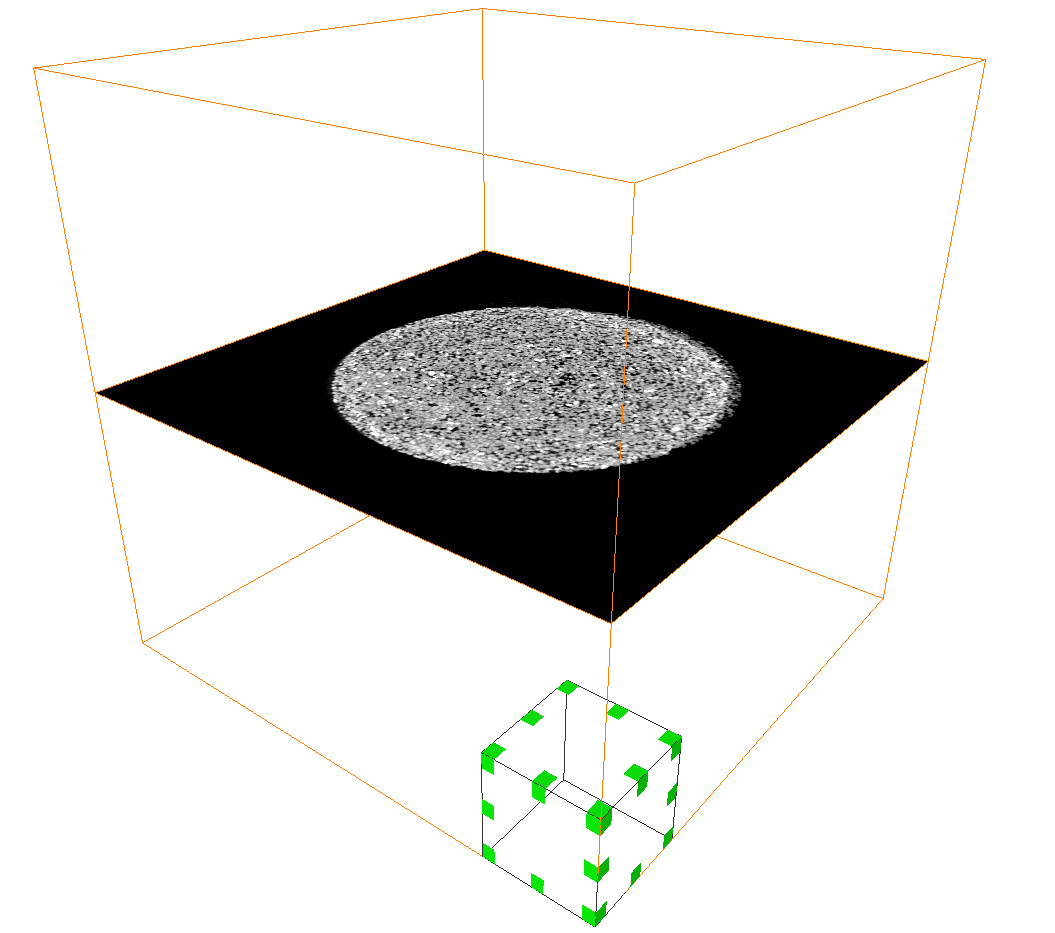

Supplement: S1 Data — (ZIP) [file pone.0296437.s001.zip › SI-Data/Data aggregation/unbiomineralization sample/3D unbiomineralization sample/5-xy.png]

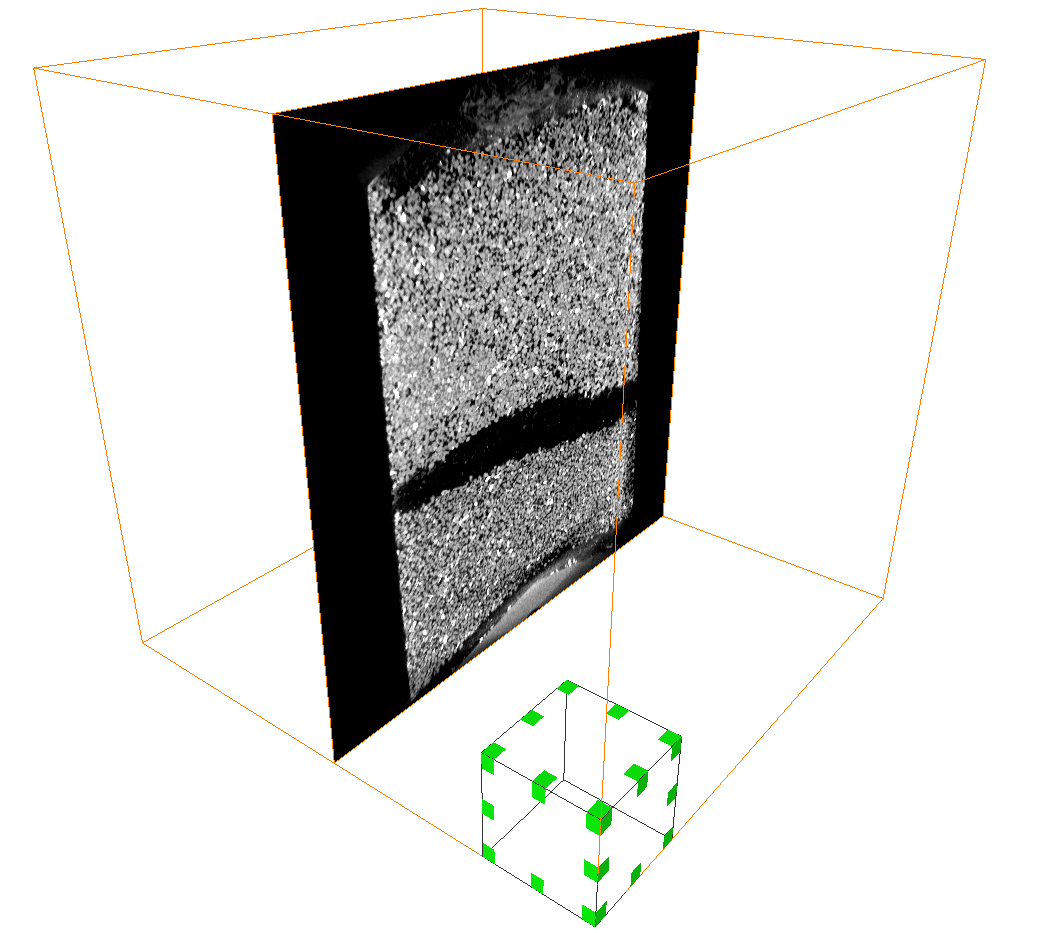

Supplement: S1 Data — (ZIP) [file pone.0296437.s001.zip › SI-Data/Data aggregation/unbiomineralization sample/3D unbiomineralization sample/5-xz.png]

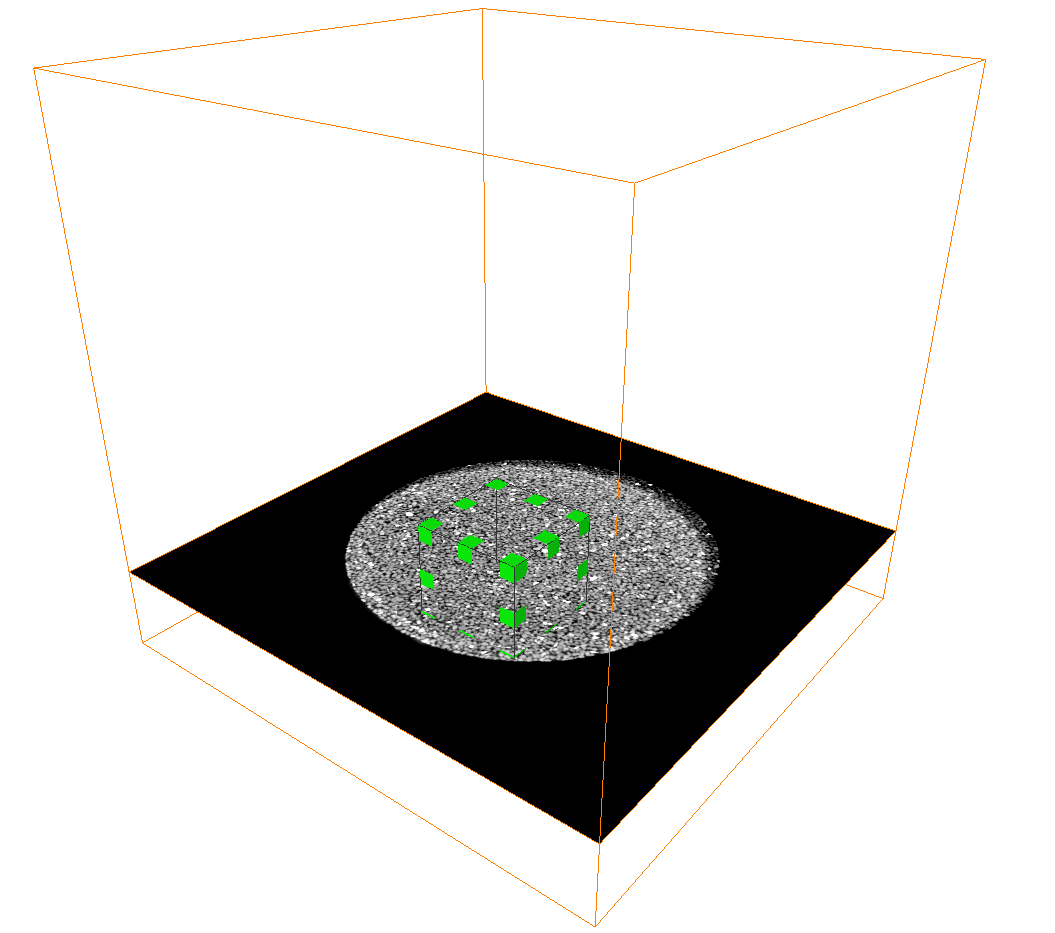

Supplement: S1 Data — (ZIP) [file pone.0296437.s001.zip › SI-Data/Data aggregation/unbiomineralization sample/3D unbiomineralization sample/6-xy.png]

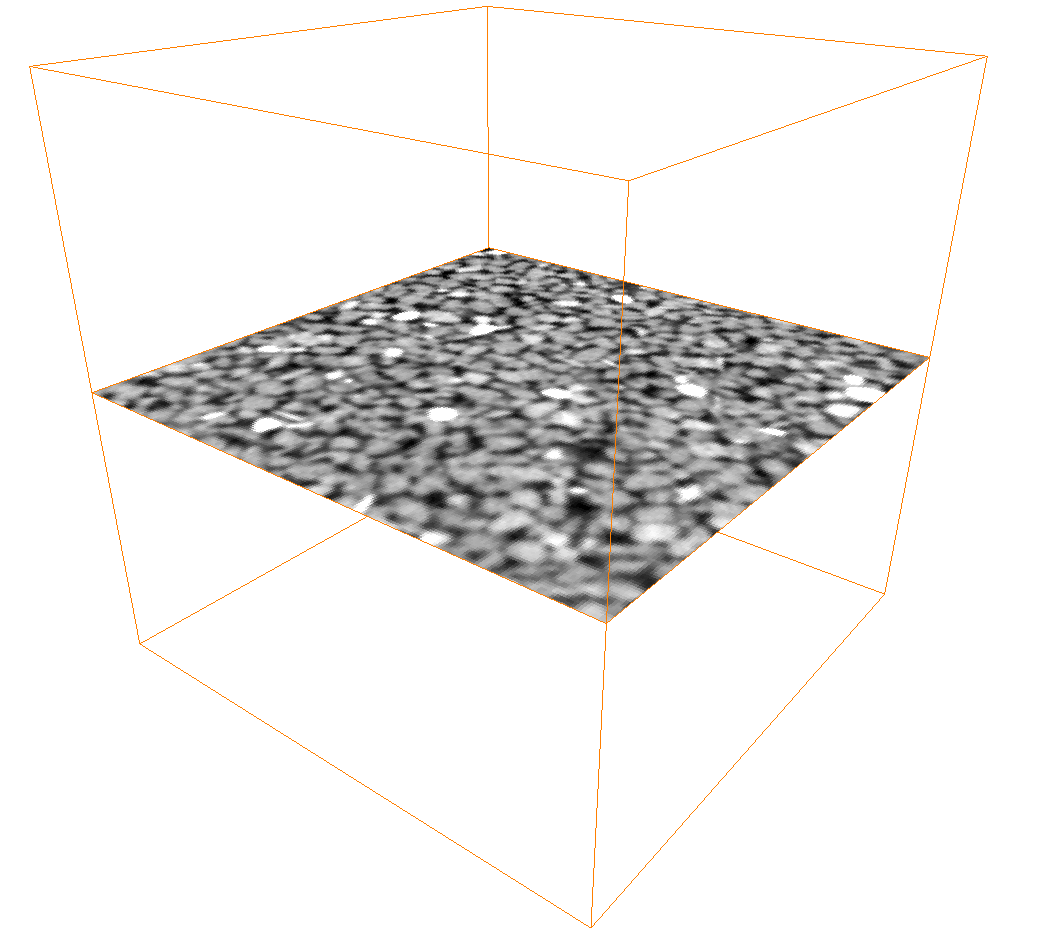

Supplement: S1 Data — (ZIP) [file pone.0296437.s001.zip › SI-Data/Data aggregation/unbiomineralization sample/3D unbiomineralization sample/7-xy.png]

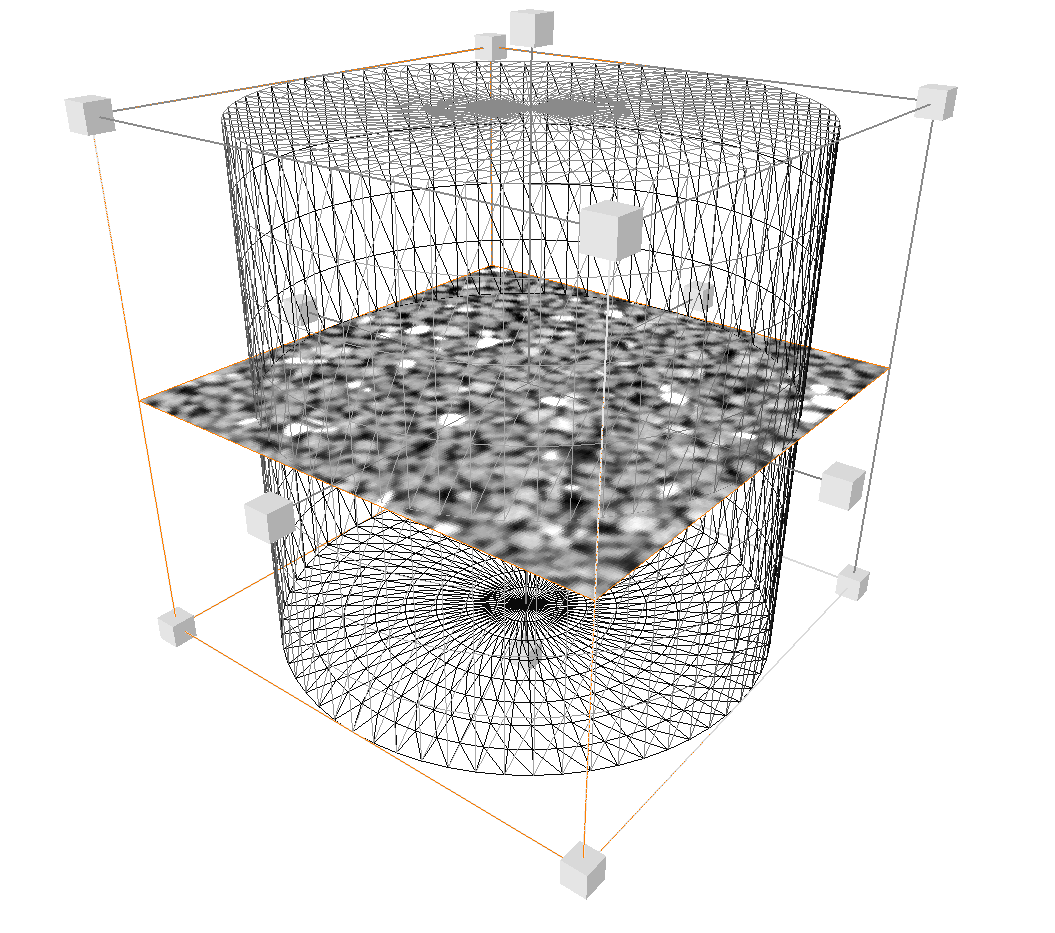

Supplement: S1 Data — (ZIP) [file pone.0296437.s001.zip › SI-Data/Data aggregation/unbiomineralization sample/3D unbiomineralization sample/8-xy.png]

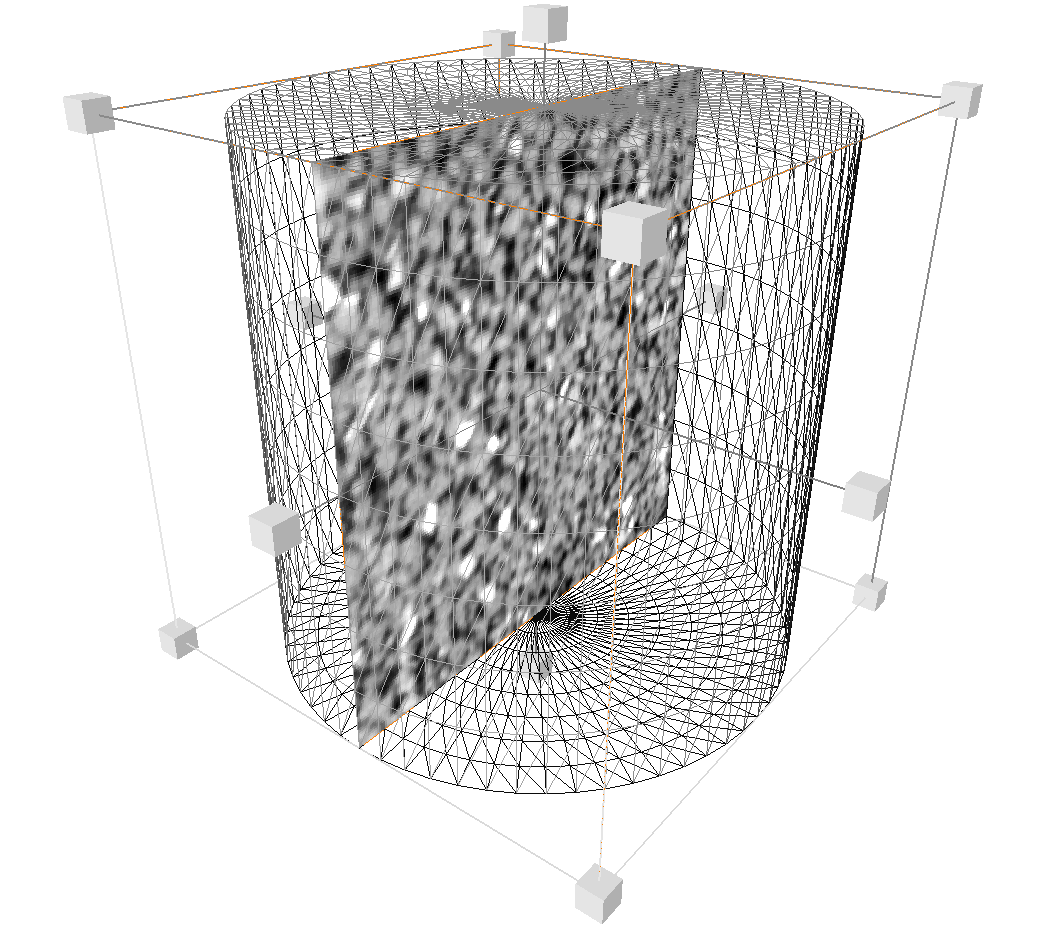

Supplement: S1 Data — (ZIP) [file pone.0296437.s001.zip › SI-Data/Data aggregation/unbiomineralization sample/3D unbiomineralization sample/8-xz.png]

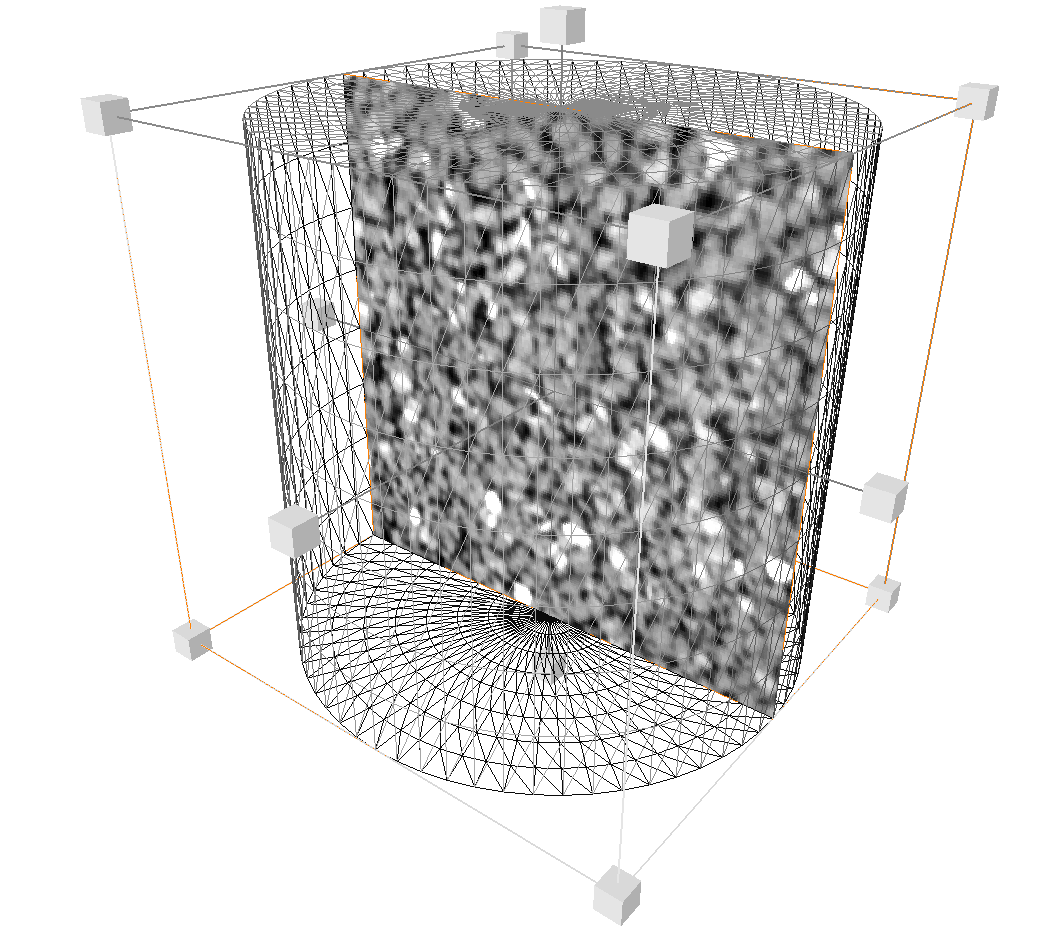

Supplement: S1 Data — (ZIP) [file pone.0296437.s001.zip › SI-Data/Data aggregation/unbiomineralization sample/3D unbiomineralization sample/8-yz.png]

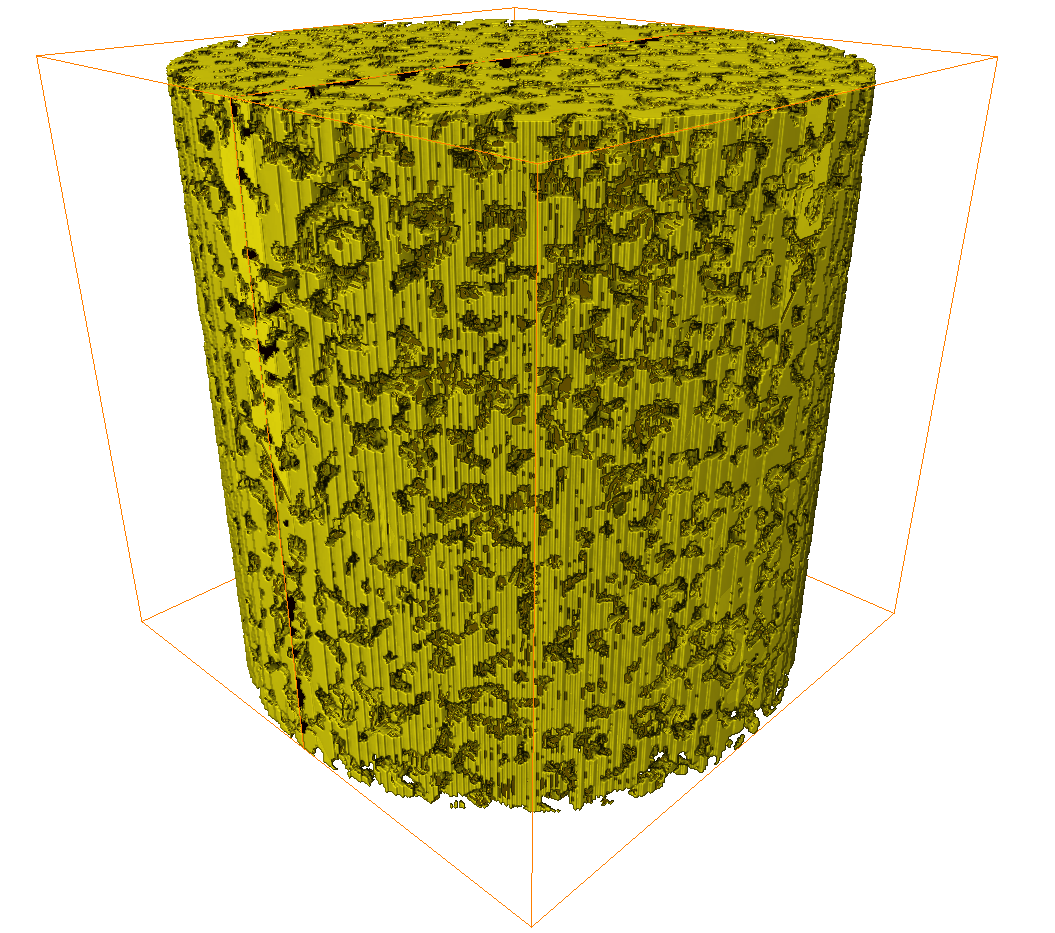

Supplement: S1 Data — (ZIP) [file pone.0296437.s001.zip › SI-Data/Data aggregation/unbiomineralization sample/unbiomineralization sample/1 keli volume Rendreing x-y (3D)-zhengdengzhou .png]

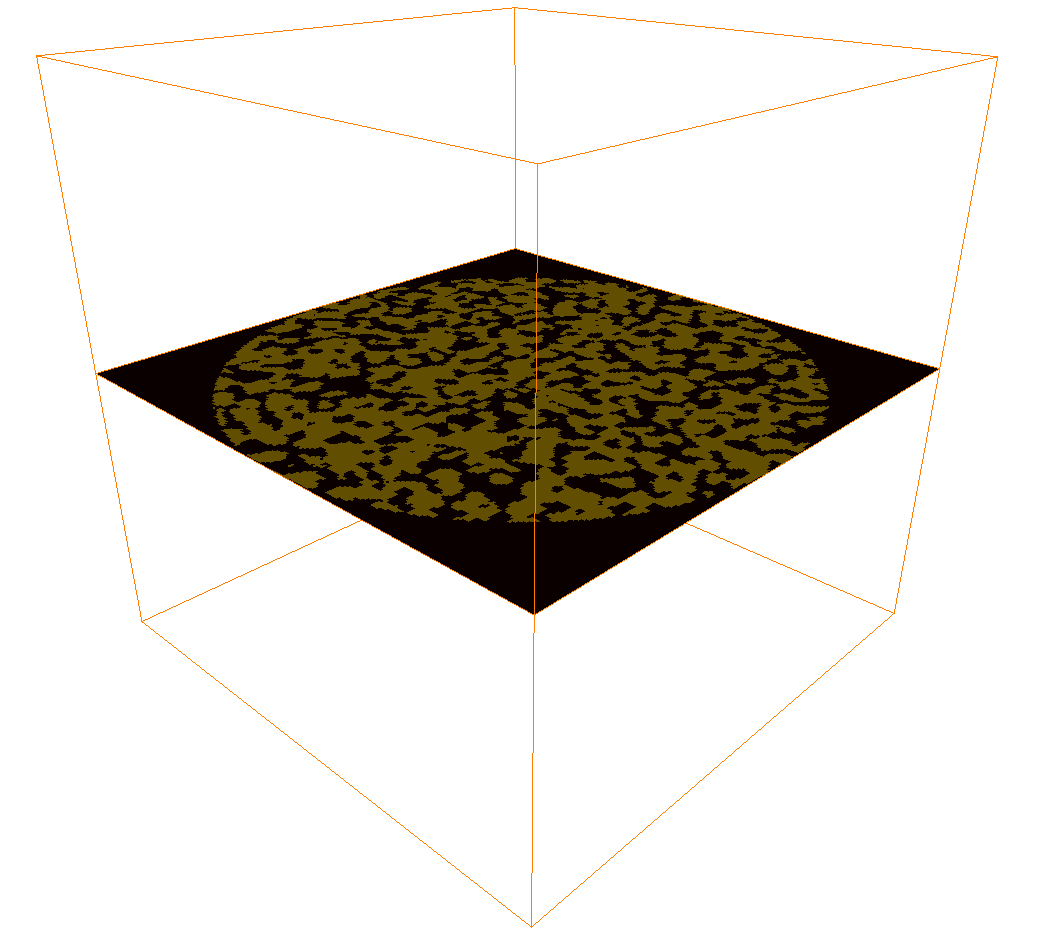

Supplement: S1 Data — (ZIP) [file pone.0296437.s001.zip › SI-Data/Data aggregation/unbiomineralization sample/unbiomineralization sample/1 keli yuzhifenge x-y (2D)-zhengdengzhou .png]

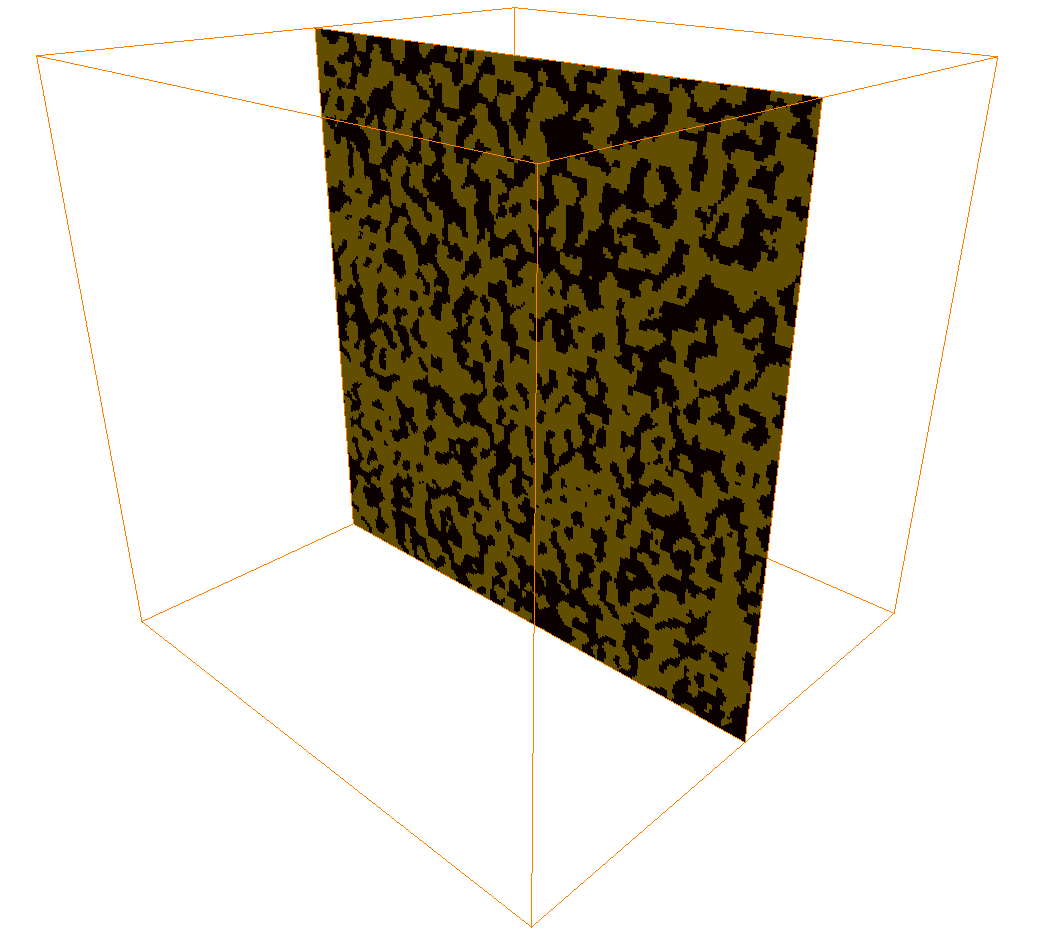

Supplement: S1 Data — (ZIP) [file pone.0296437.s001.zip › SI-Data/Data aggregation/unbiomineralization sample/unbiomineralization sample/1 keli yuzhifenge x-z (2D)-zhengdengzhou .png]

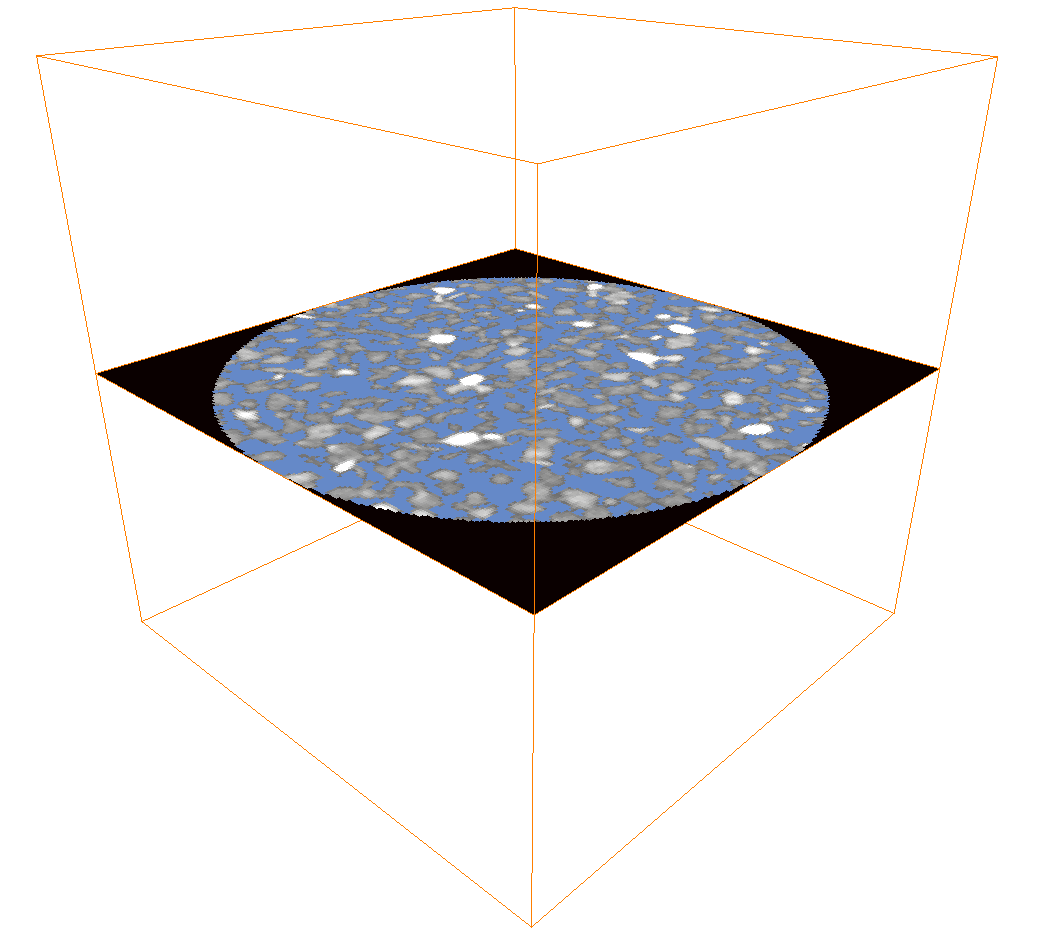

Supplement: S1 Data — (ZIP) [file pone.0296437.s001.zip › SI-Data/Data aggregation/unbiomineralization sample/unbiomineralization sample/1 kongxi yuzhifenge x-y (2D)-zhengdengzhou .png]

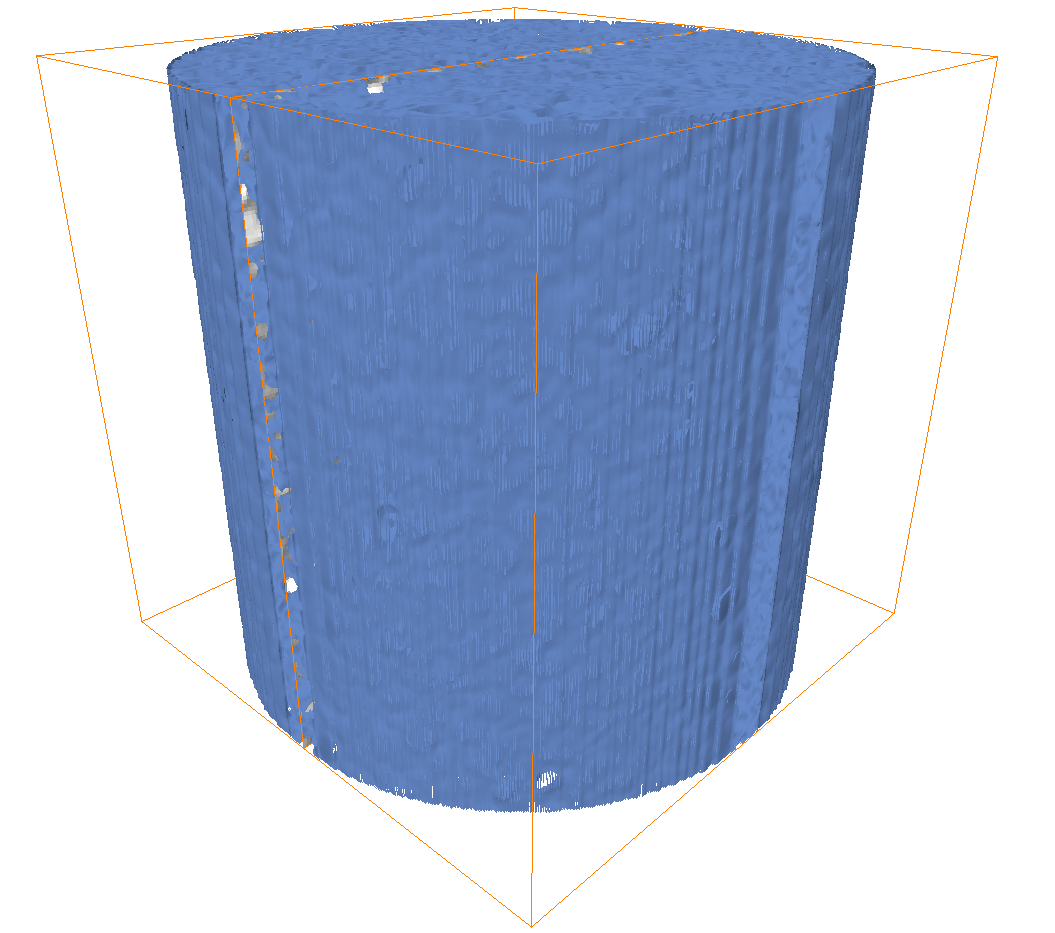

Supplement: S1 Data — (ZIP) [file pone.0296437.s001.zip › SI-Data/Data aggregation/unbiomineralization sample/unbiomineralization sample/1 kongxi yuzhifenge x-y (3D)-zhengdengzhou .png]

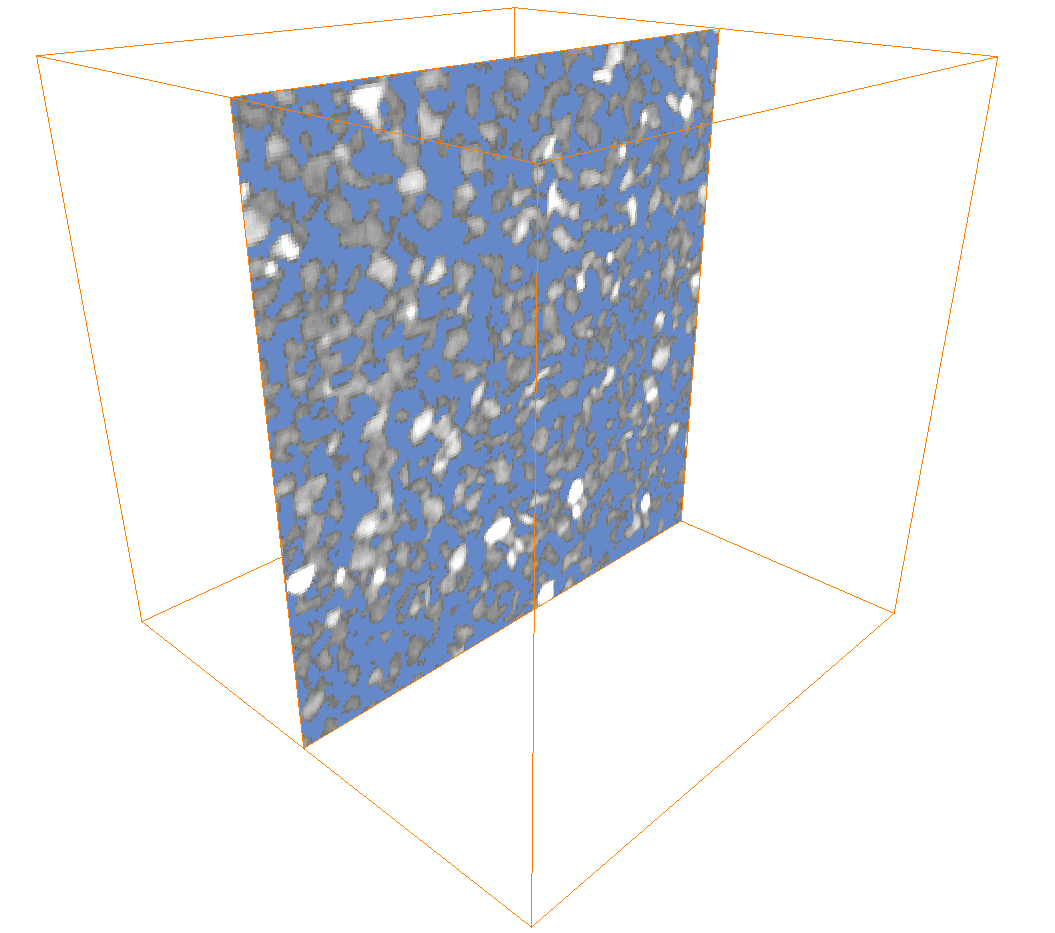

Supplement: S1 Data — (ZIP) [file pone.0296437.s001.zip › SI-Data/Data aggregation/unbiomineralization sample/unbiomineralization sample/1 kongxi yuzhifenge y-z (2D)-zhengdengzhou .png]

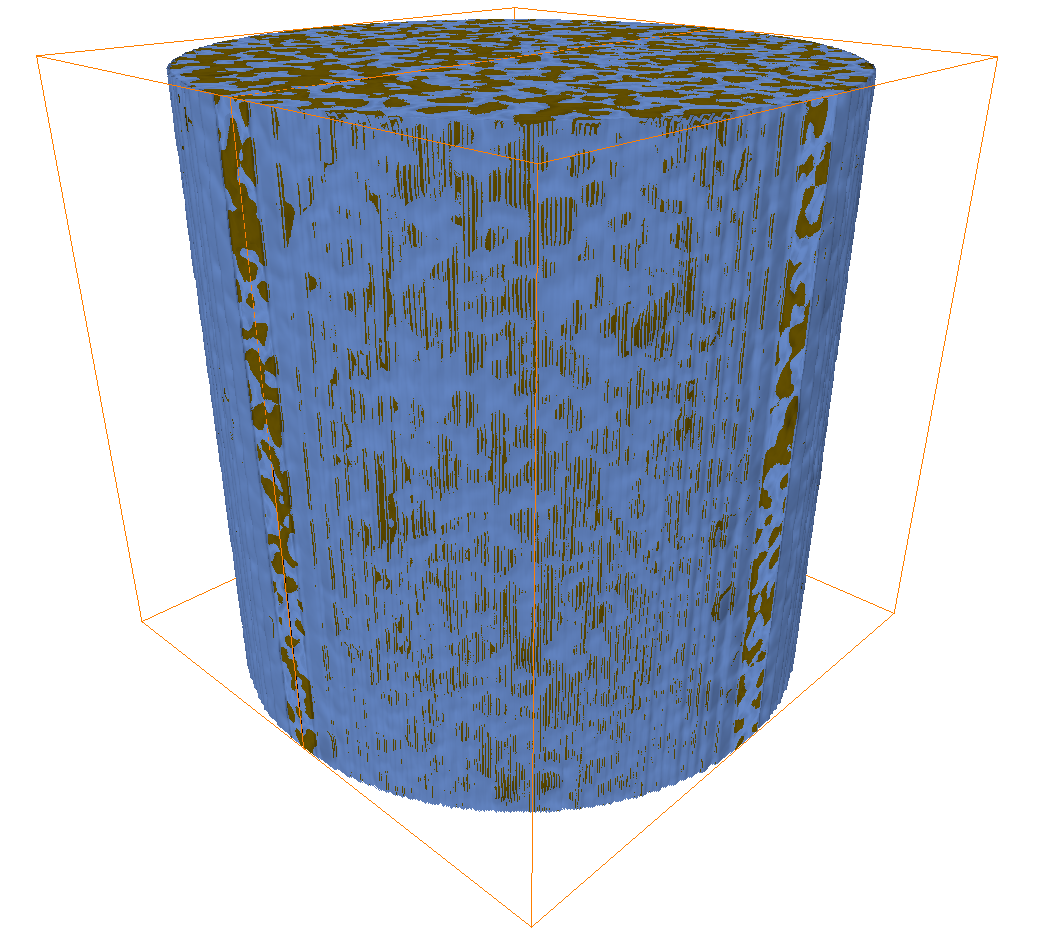

Supplement: S1 Data — (ZIP) [file pone.0296437.s001.zip › SI-Data/Data aggregation/unbiomineralization sample/unbiomineralization sample/1 kongxi he keli yuzhifenge x-y (3D)-zhengdengzhou .png]

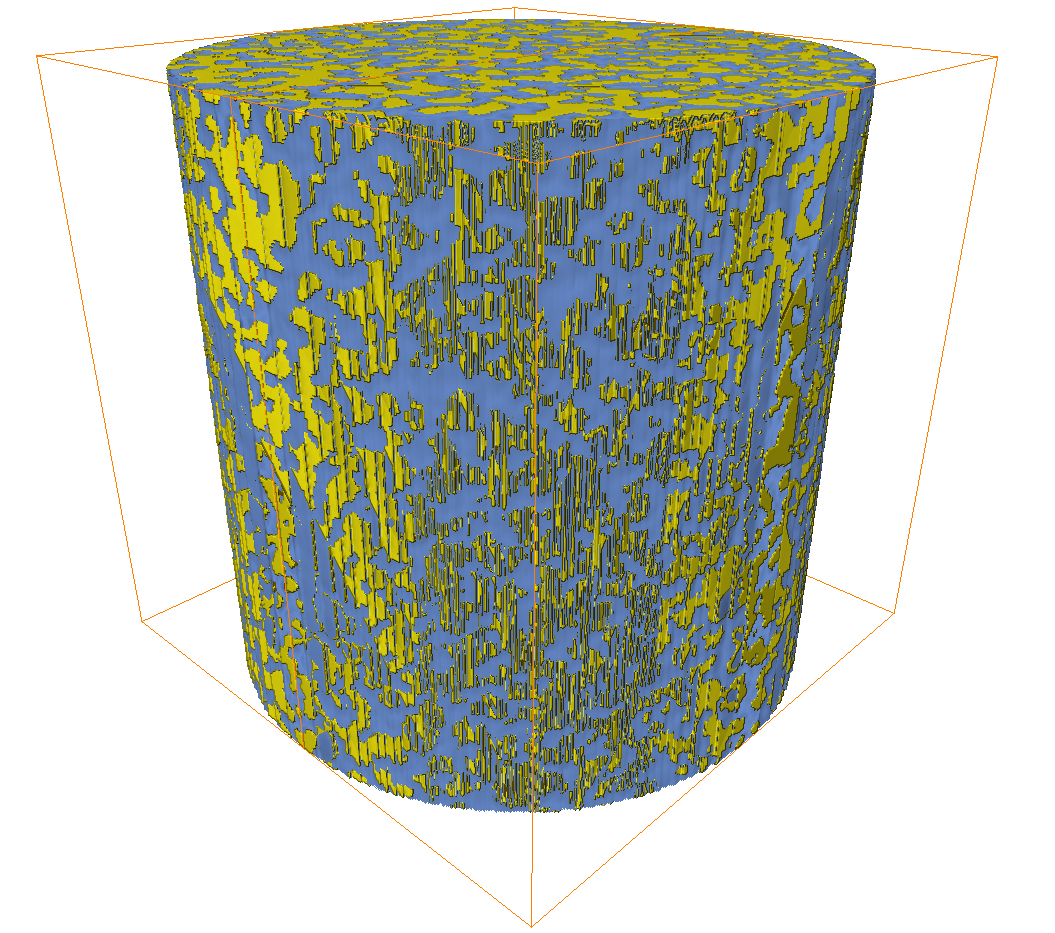

Supplement: S1 Data — (ZIP) [file pone.0296437.s001.zip › SI-Data/Data aggregation/unbiomineralization sample/unbiomineralization sample/1 kongxi he keli yuzhifenge x-y (3D)-zhengdengzhou(keli he kongxi ) .png]

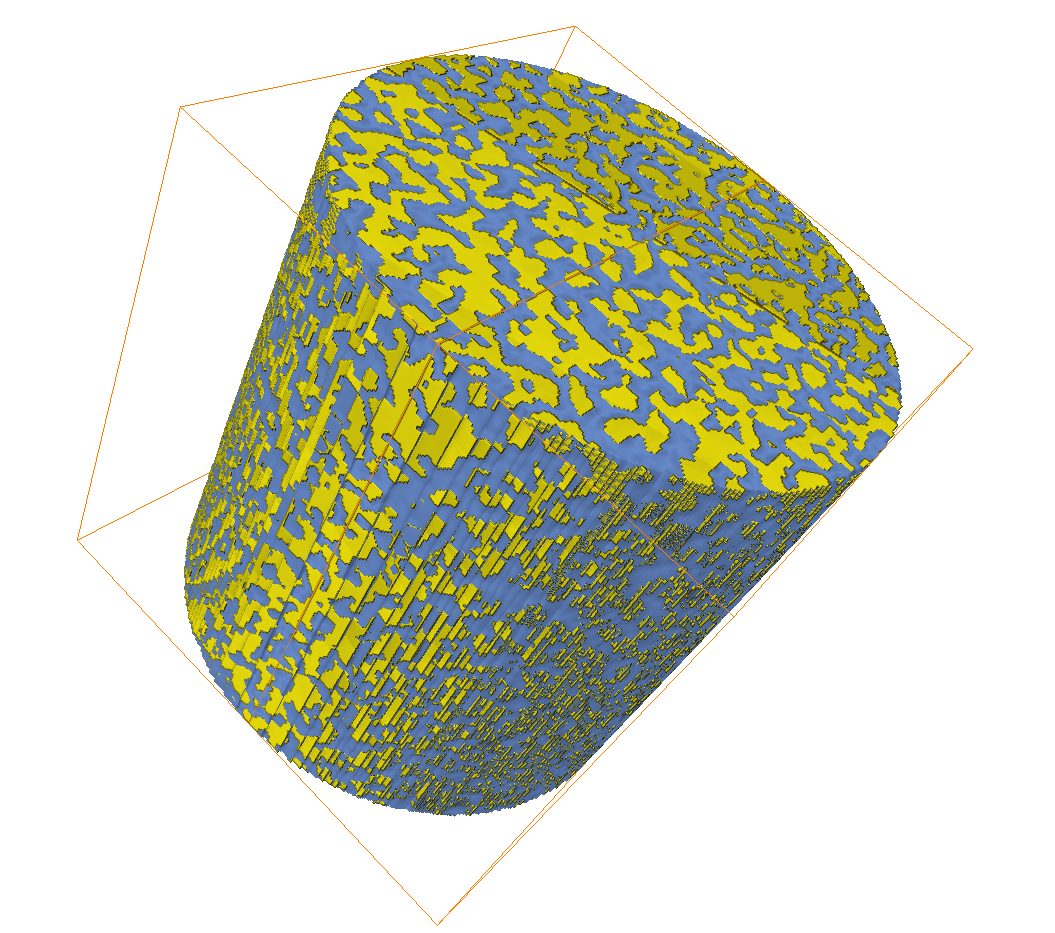

Supplement: S1 Data — (ZIP) [file pone.0296437.s001.zip › SI-Data/Data aggregation/unbiomineralization sample/unbiomineralization sample/1 kongxi he keli yuzhifenge x-y (3D)-zhengdengzhou(keli he kongxi 1) .png]

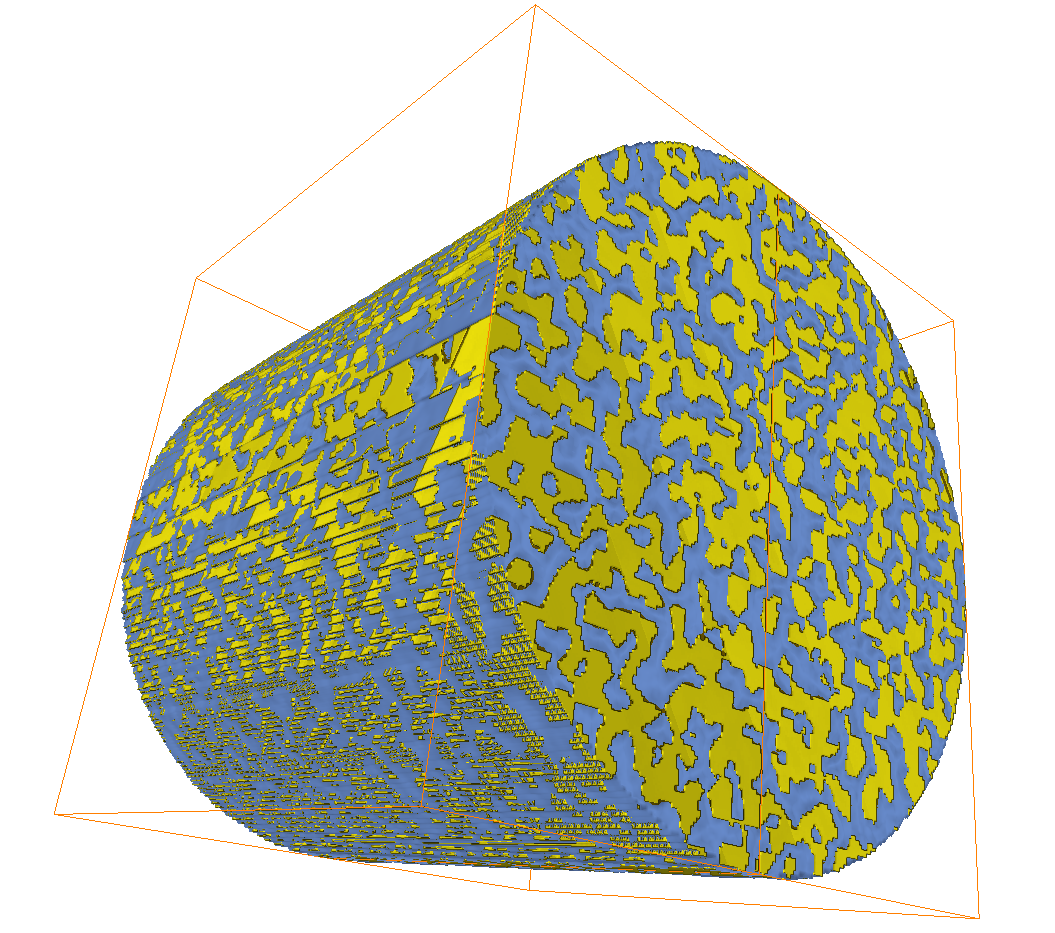

Supplement: S1 Data — (ZIP) [file pone.0296437.s001.zip › SI-Data/Data aggregation/unbiomineralization sample/unbiomineralization sample/1 kongxi he keli yuzhifenge x-y (3D)-zhengdengzhou(keli he kongxi 2) .png]

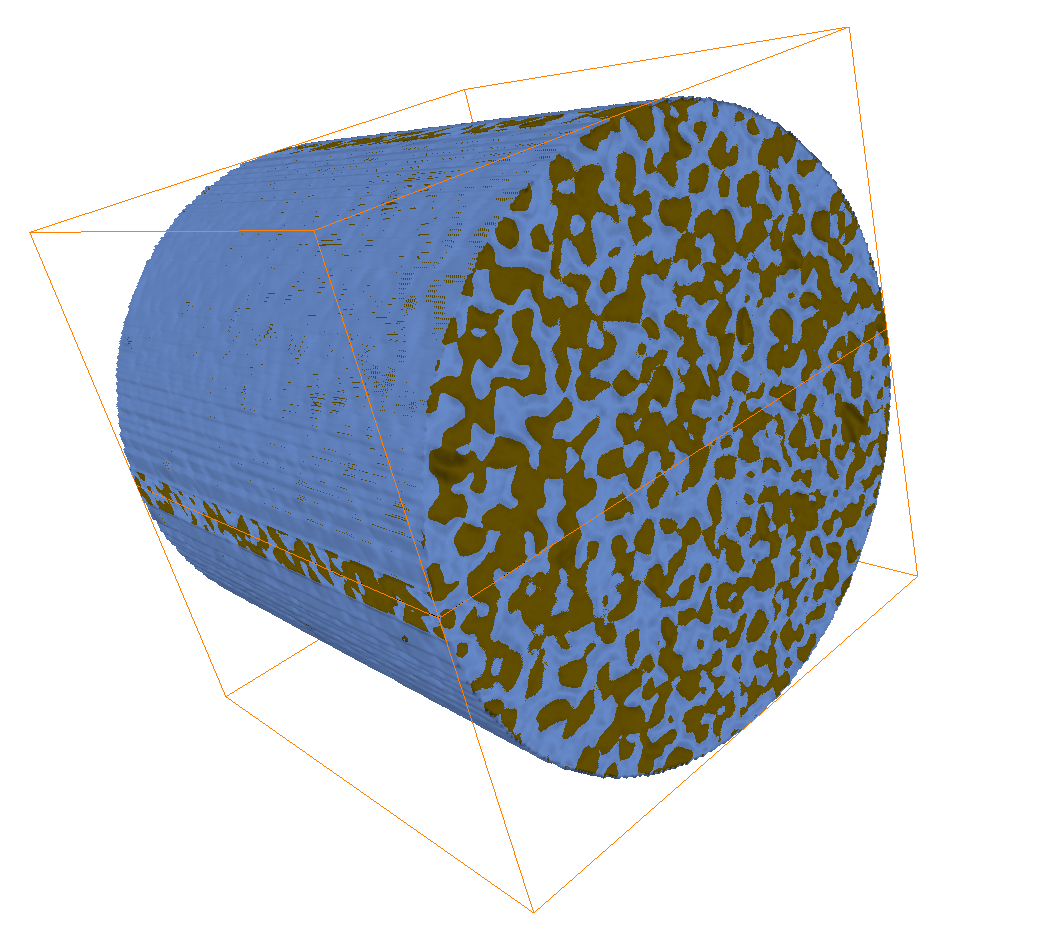

Supplement: S1 Data — (ZIP) [file pone.0296437.s001.zip › SI-Data/Data aggregation/unbiomineralization sample/unbiomineralization sample/1 kongxi he keli yuzhifenge x-y (3D)-zhengdengzhou1 .png]

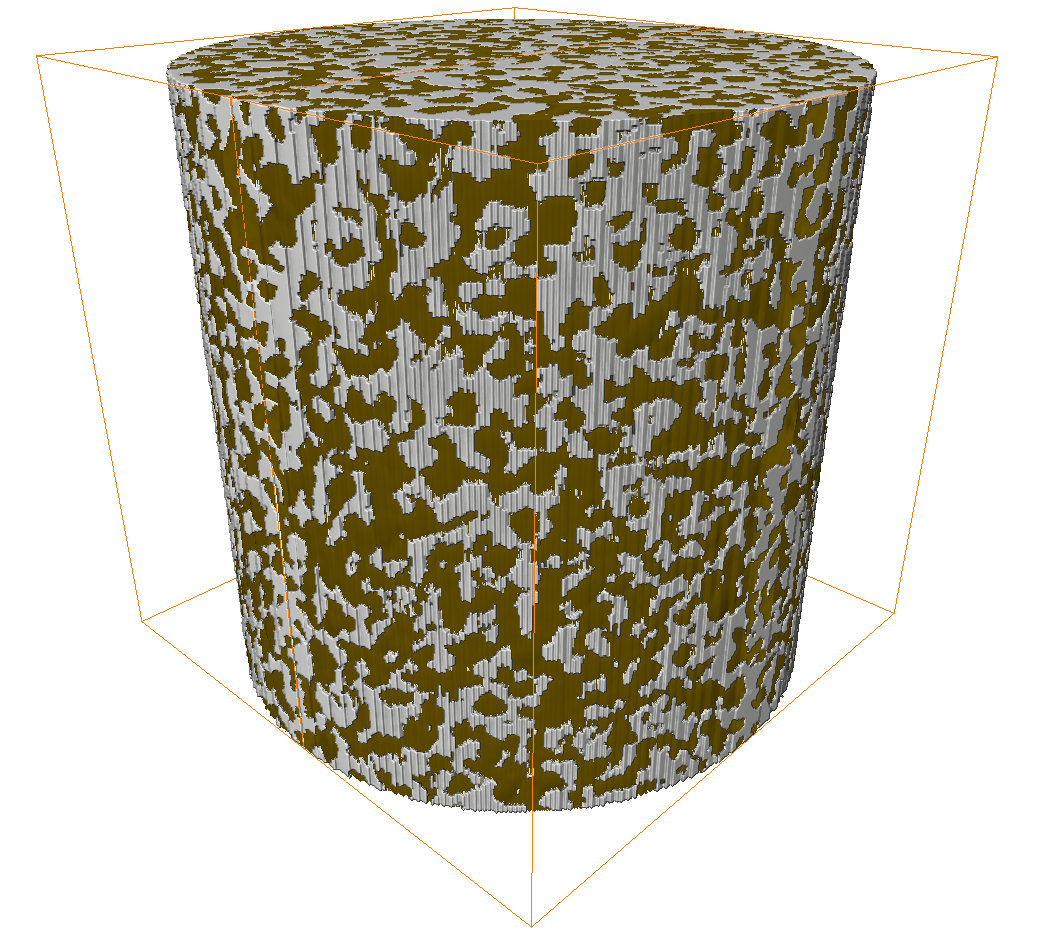

Supplement: S1 Data — (ZIP) [file pone.0296437.s001.zip › SI-Data/Data aggregation/unbiomineralization sample/unbiomineralization sample/2 kongxi jia keli volume Rendreing x-y (3D)-zhengdengzhou .png]

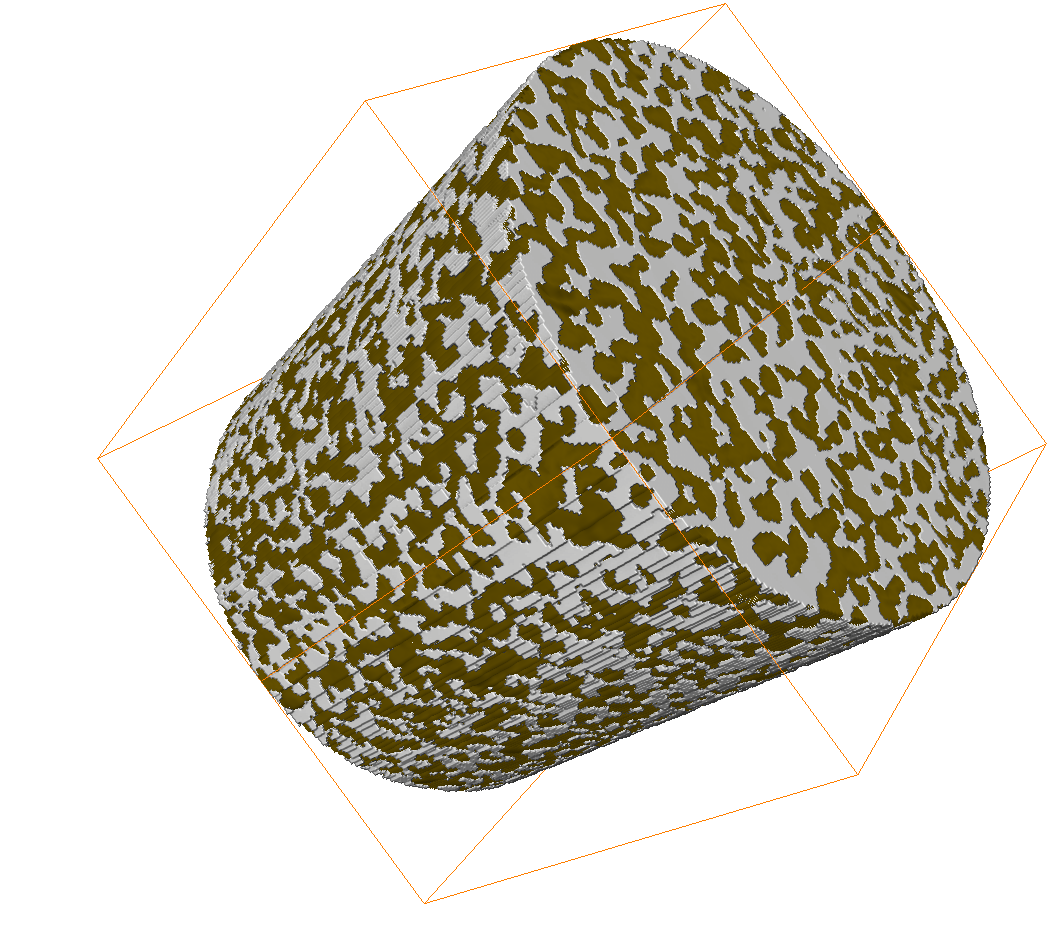

Supplement: S1 Data — (ZIP) [file pone.0296437.s001.zip › SI-Data/Data aggregation/unbiomineralization sample/unbiomineralization sample/2 kongxi jia keli volume Rendreing x-y (3D)-zhengdengzhou1 .png]

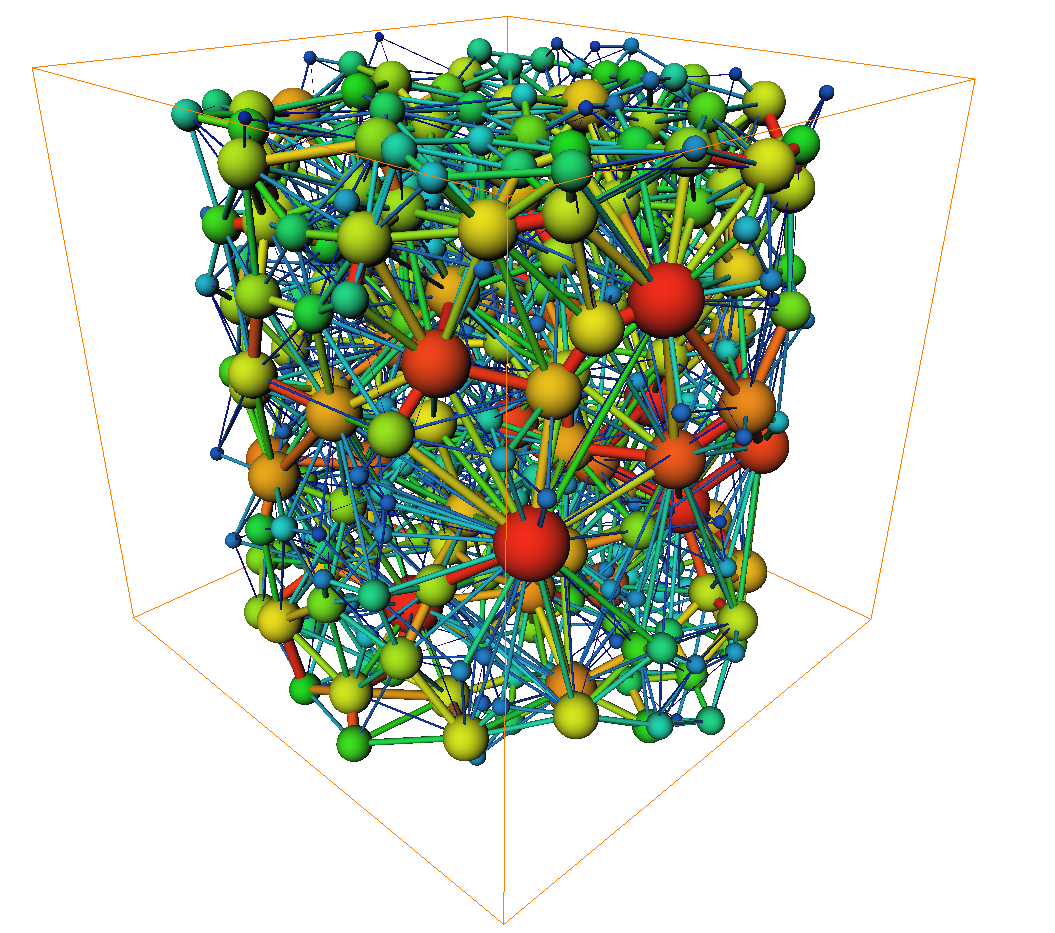

Supplement: S1 Data — (ZIP) [file pone.0296437.s001.zip › SI-Data/Data aggregation/unbiomineralization sample/unbiomineralization sample/3 quchu guli kongxi kongxiwangluomoxing x-y (3D)-zhengdengzhou .png]

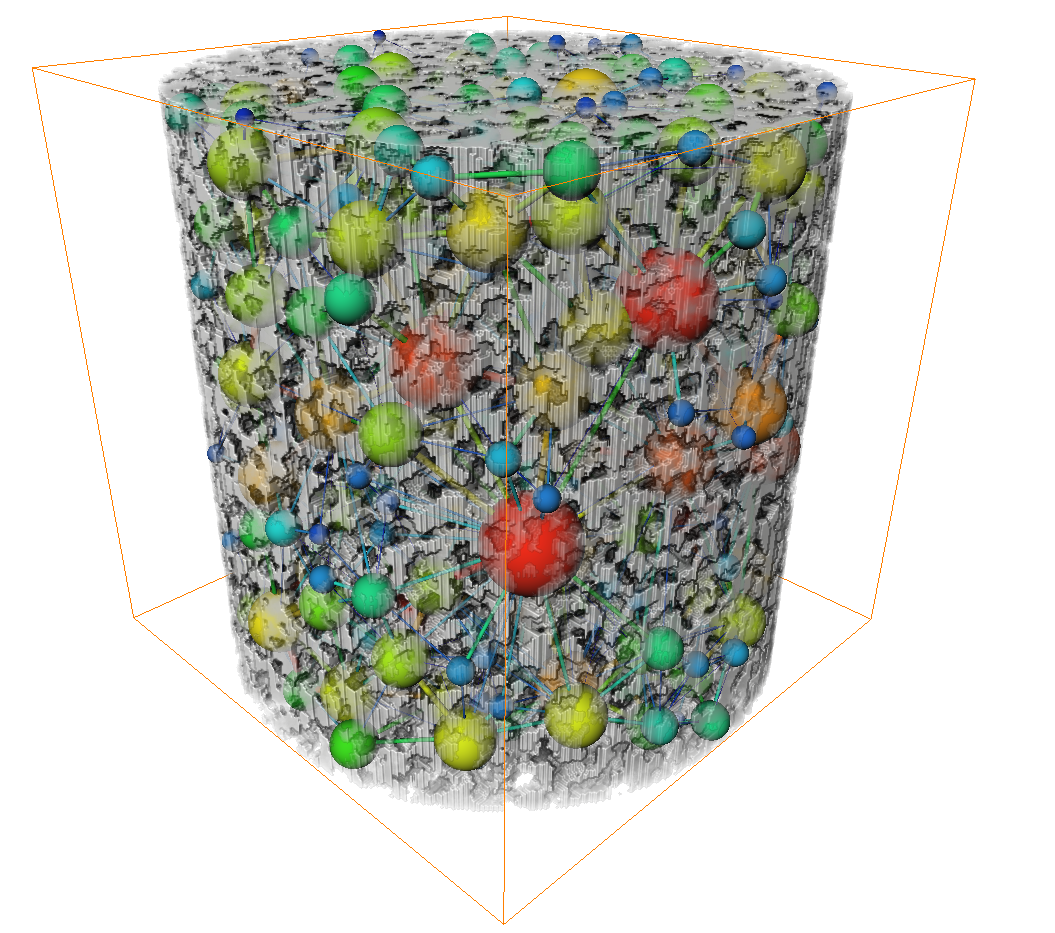

Supplement: S1 Data — (ZIP) [file pone.0296437.s001.zip › SI-Data/Data aggregation/unbiomineralization sample/unbiomineralization sample/3 quchu guli kongxi kongxiwangluomoxing jia keli x-y (3D)-zhengdengzhou1 .png]

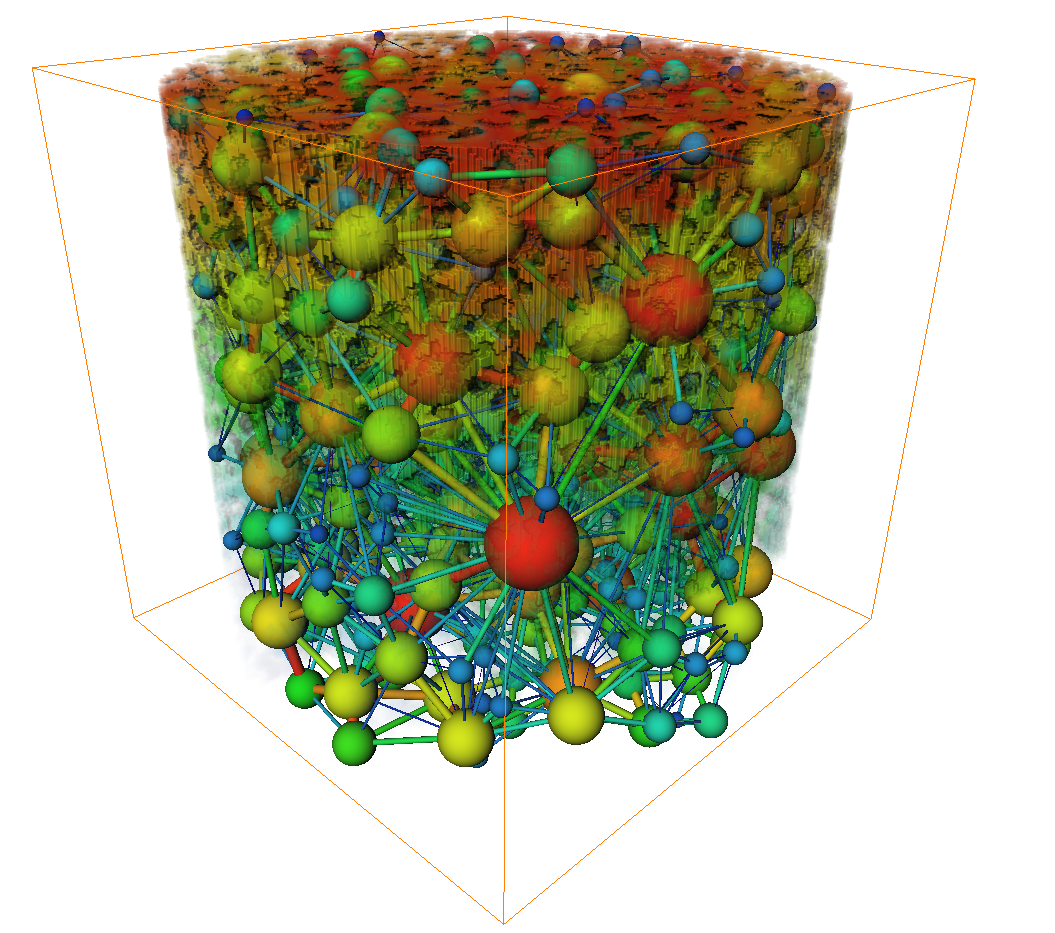

Supplement: S1 Data — (ZIP) [file pone.0296437.s001.zip › SI-Data/Data aggregation/unbiomineralization sample/unbiomineralization sample/3 quchu guli kongxi kongxiwangluomoxing jia yuan kongxi x-y (3D)-zhengdengzhou1 .png]

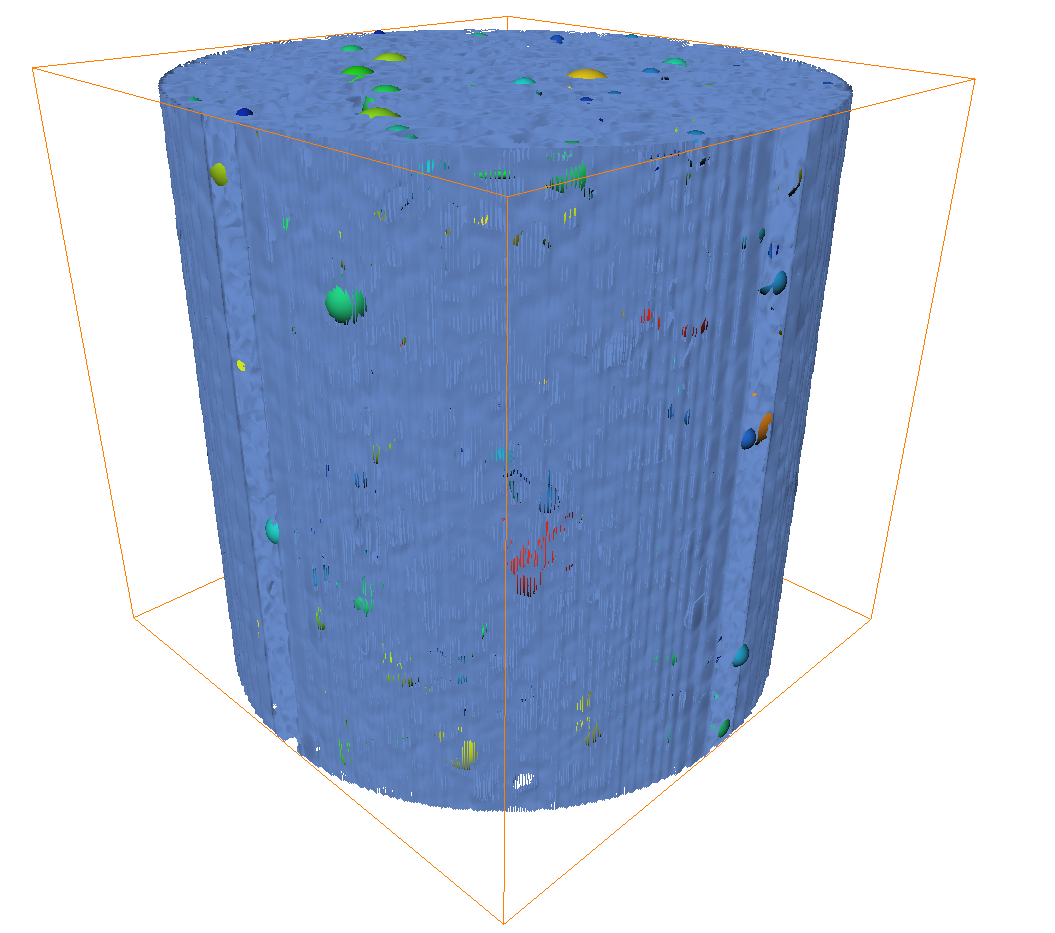

Supplement: S1 Data — (ZIP) [file pone.0296437.s001.zip › SI-Data/Data aggregation/unbiomineralization sample/unbiomineralization sample/3 quchu guli kongxi kongxiwangluomoxing jia yuzhifenge x-y (3D)-zhengdengzhou1 .png]

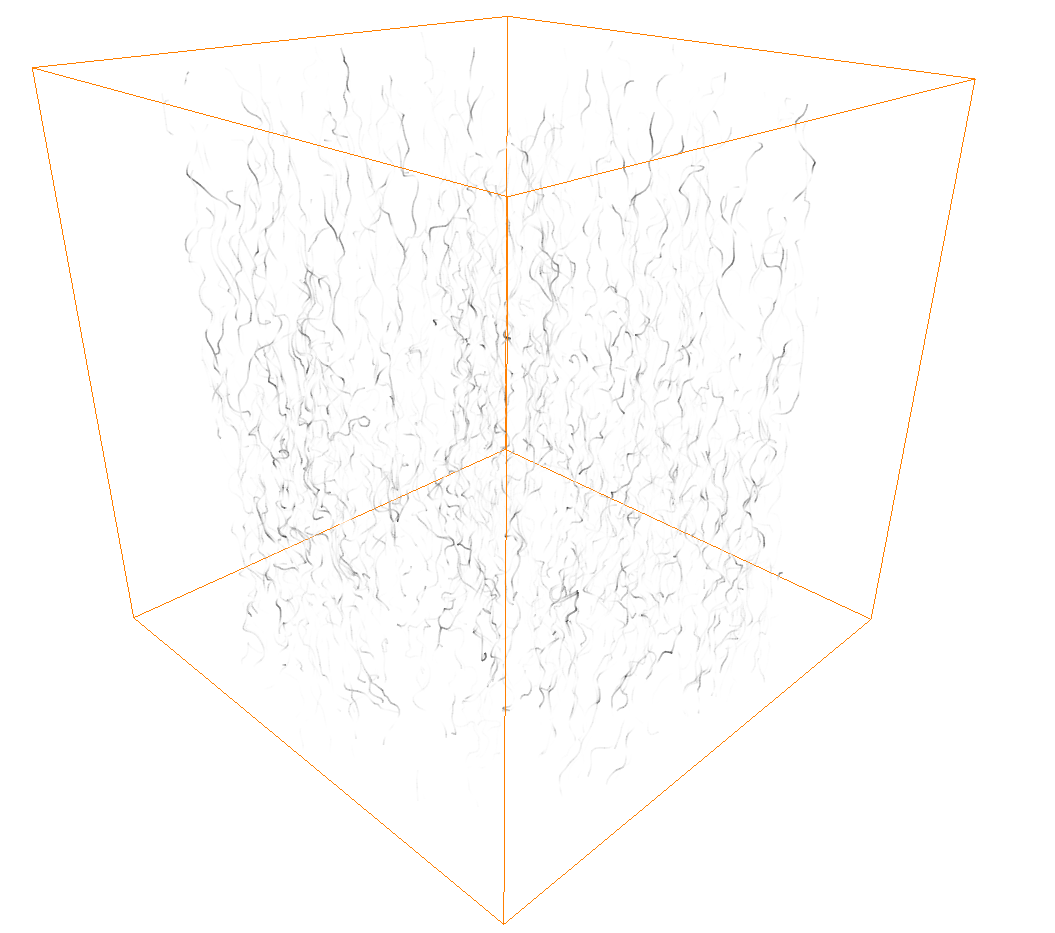

Supplement: S1 Data — (ZIP) [file pone.0296437.s001.zip › SI-Data/Data aggregation/unbiomineralization sample/unbiomineralization sample/3 quchu guli kongxi liuxian x-y (3D)-zhengdengzhou1 .png]

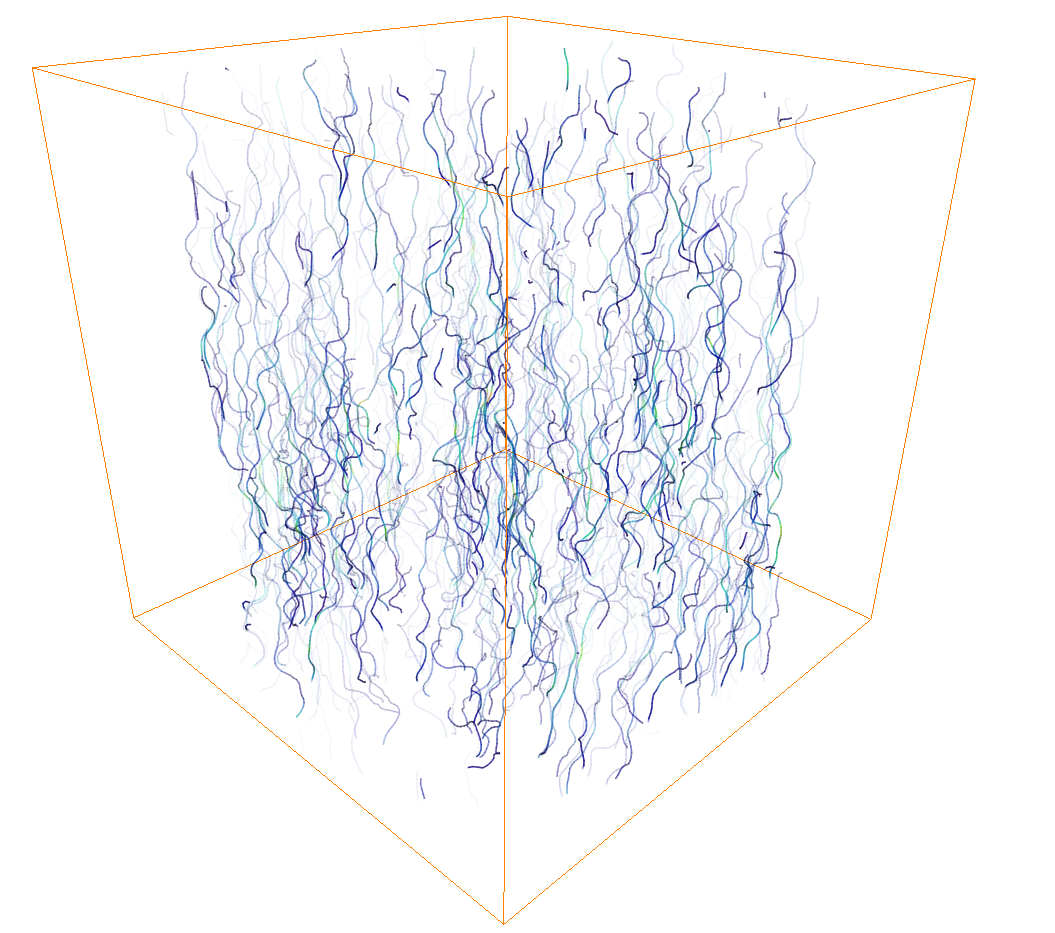

Supplement: S1 Data — (ZIP) [file pone.0296437.s001.zip › SI-Data/Data aggregation/unbiomineralization sample/unbiomineralization sample/3 quchu guli kongxi liuxian1 x-y (3D)-zhengdengzhou1 .png]

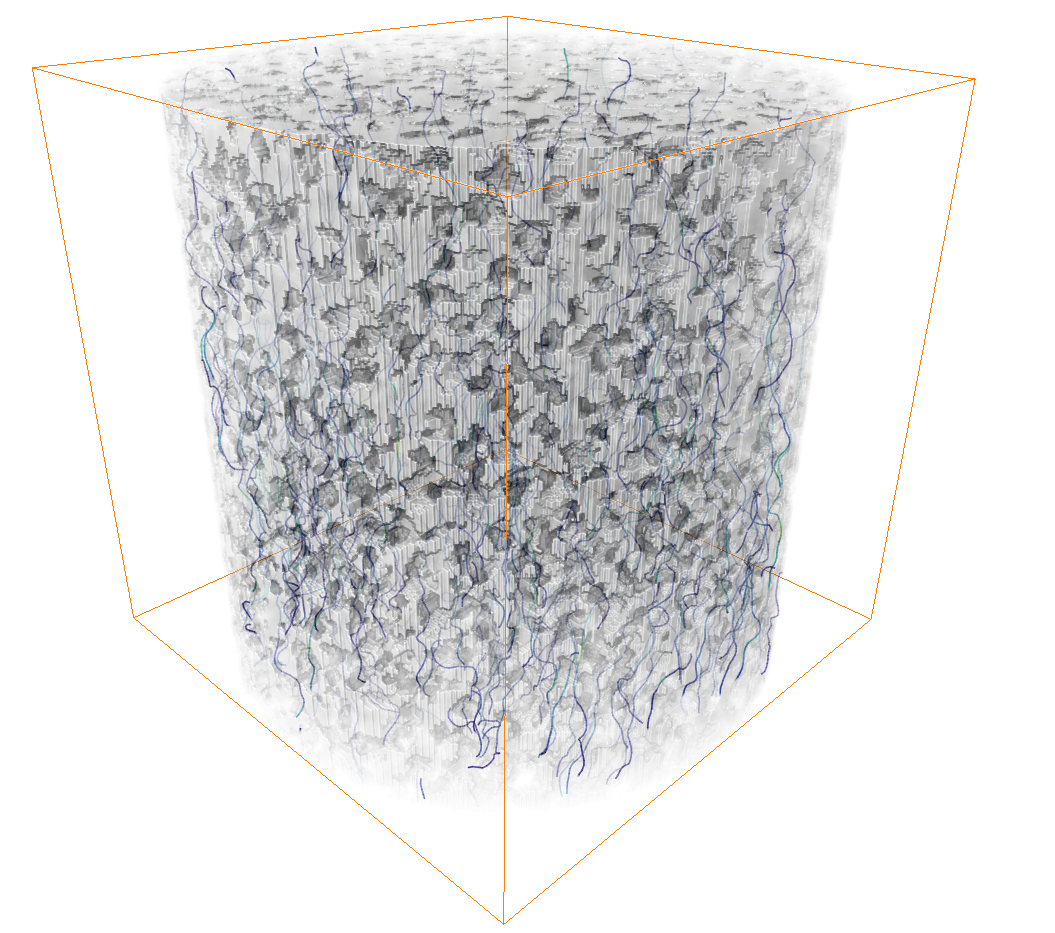

Supplement: S1 Data — (ZIP) [file pone.0296437.s001.zip › SI-Data/Data aggregation/unbiomineralization sample/unbiomineralization sample/3 quchu guli kongxi liuxian2 jia kongxi x-y (3D)-zhengdengzhou1 .png]

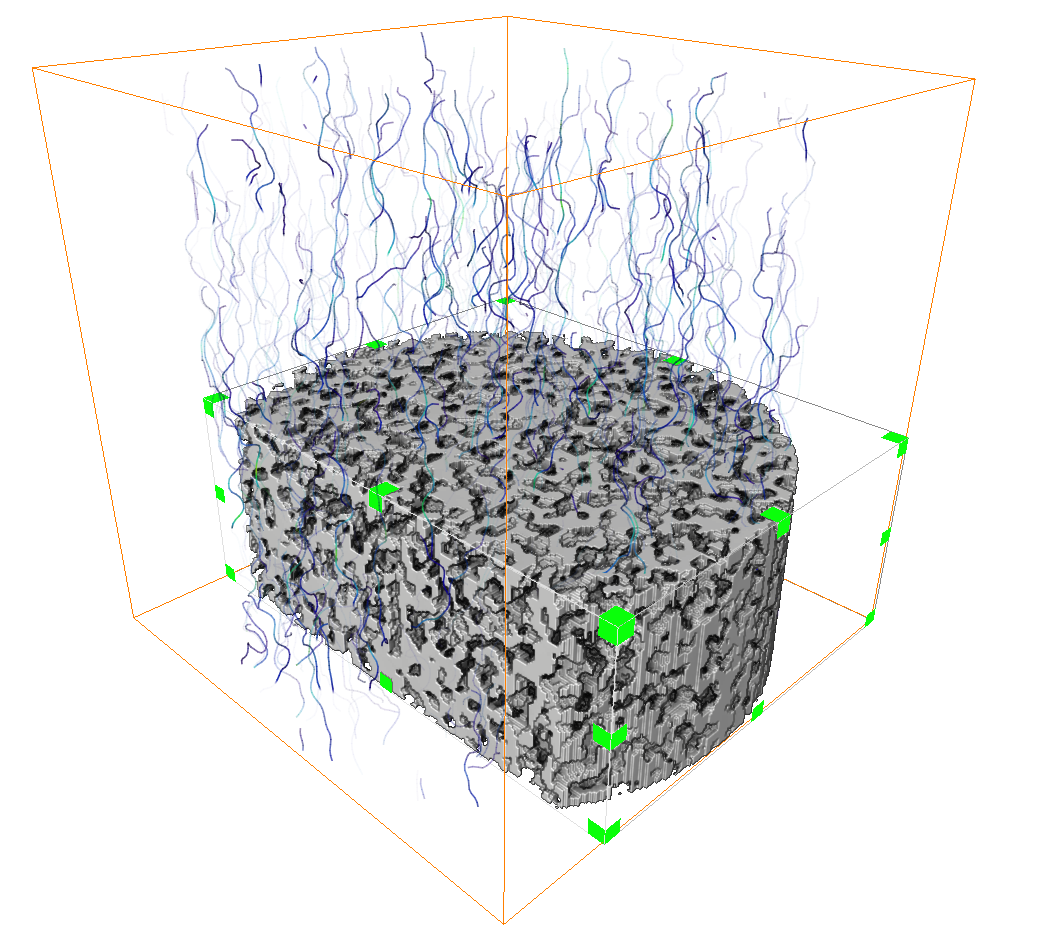

Supplement: S1 Data — (ZIP) [file pone.0296437.s001.zip › SI-Data/Data aggregation/unbiomineralization sample/unbiomineralization sample/3 quchu guli kongxi liuxian2 jia kongxi4 x-y (3D)-zhengdengzhou1 .png]

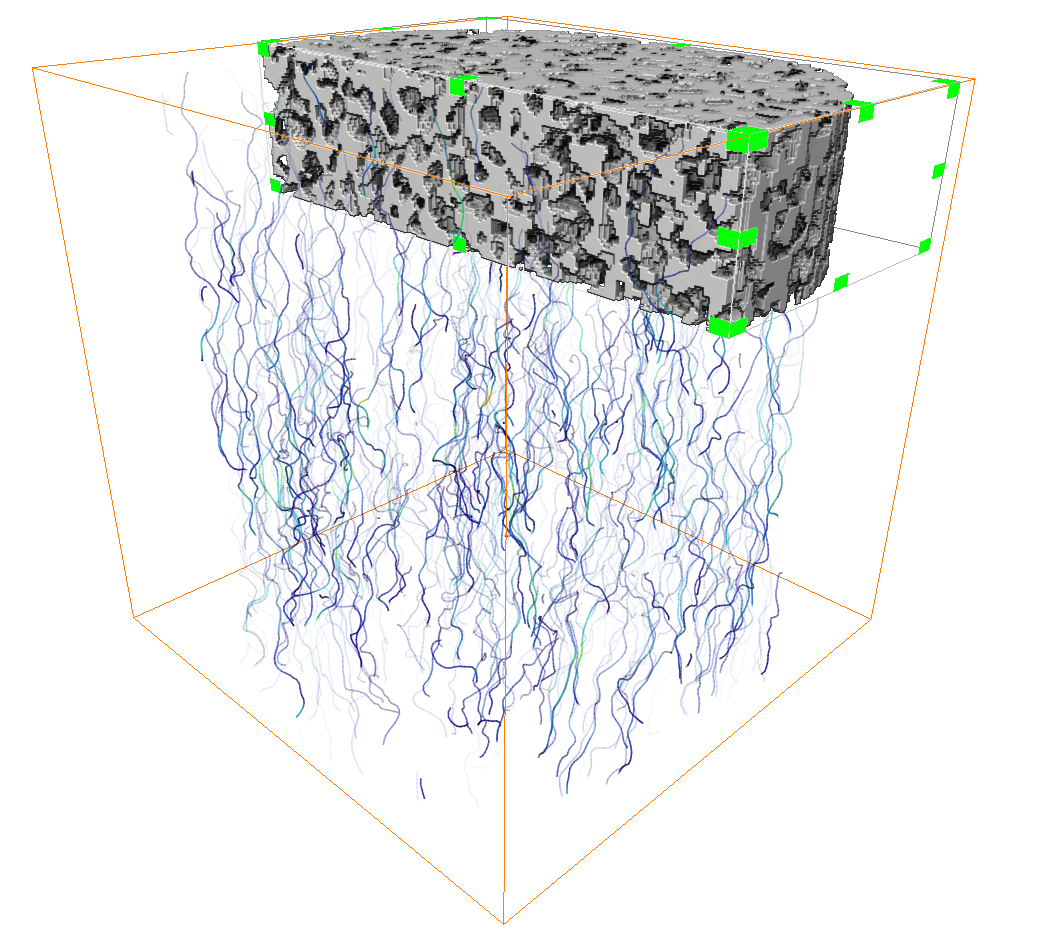

Supplement: S1 Data — (ZIP) [file pone.0296437.s001.zip › SI-Data/Data aggregation/unbiomineralization sample/unbiomineralization sample/3 quchu guli kongxi liuxian2 jia kongxi8 x-y (3D)-zhengdengzhou1 .png]

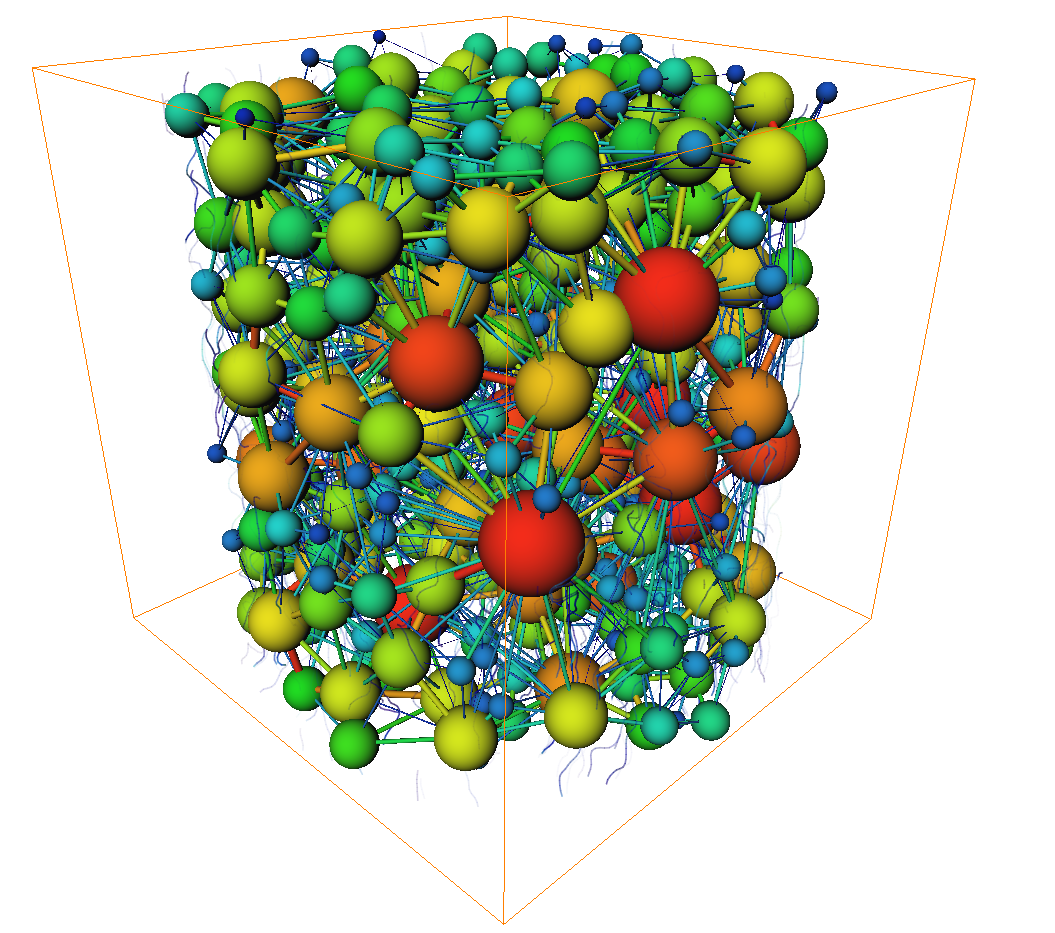

Supplement: S1 Data — (ZIP) [file pone.0296437.s001.zip › SI-Data/Data aggregation/unbiomineralization sample/unbiomineralization sample/3 quchu guli kongxi liuxian2 jia kongxiwangluomoxing x-y (3D)-zhengdengzhou1 .png]

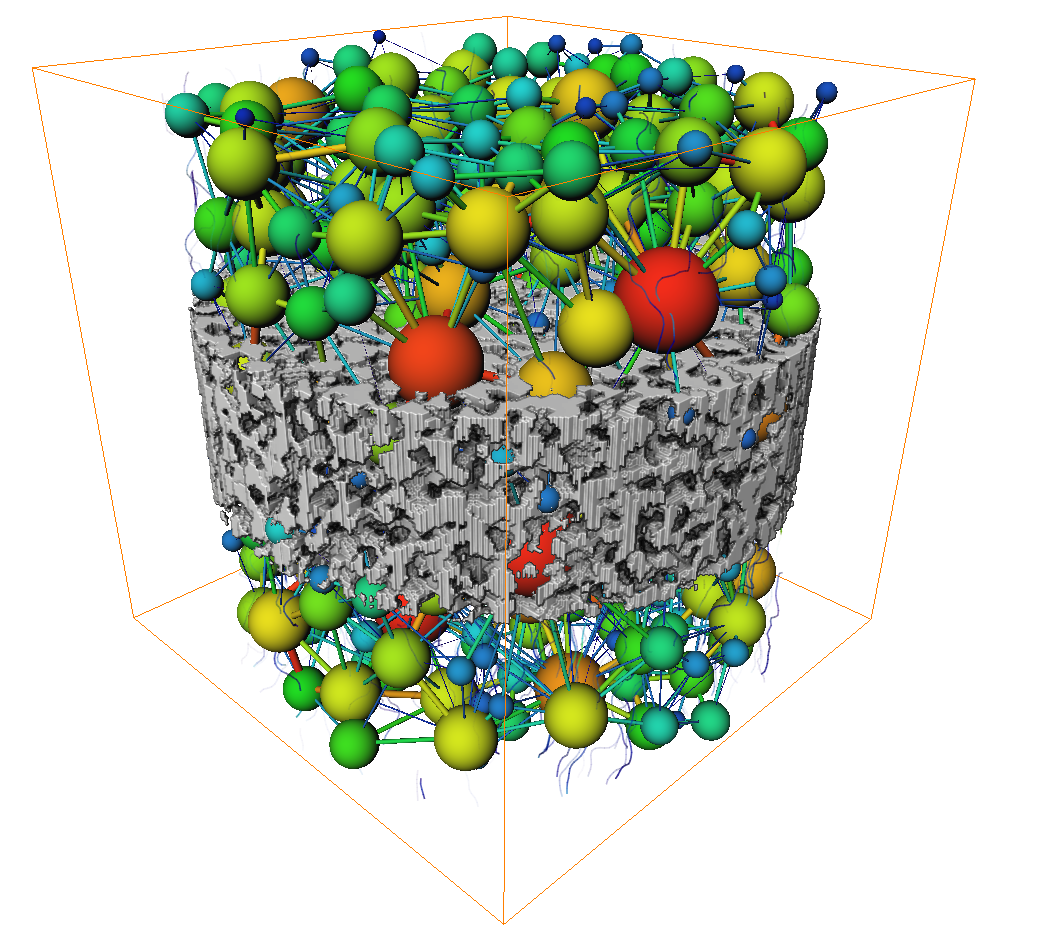

Supplement: S1 Data — (ZIP) [file pone.0296437.s001.zip › SI-Data/Data aggregation/unbiomineralization sample/unbiomineralization sample/3 quchu guli kongxi liuxian2 jia kongxiwangluomoxing jia kongxi 4 x-y (3D)-zhengdengzhou1 .png]

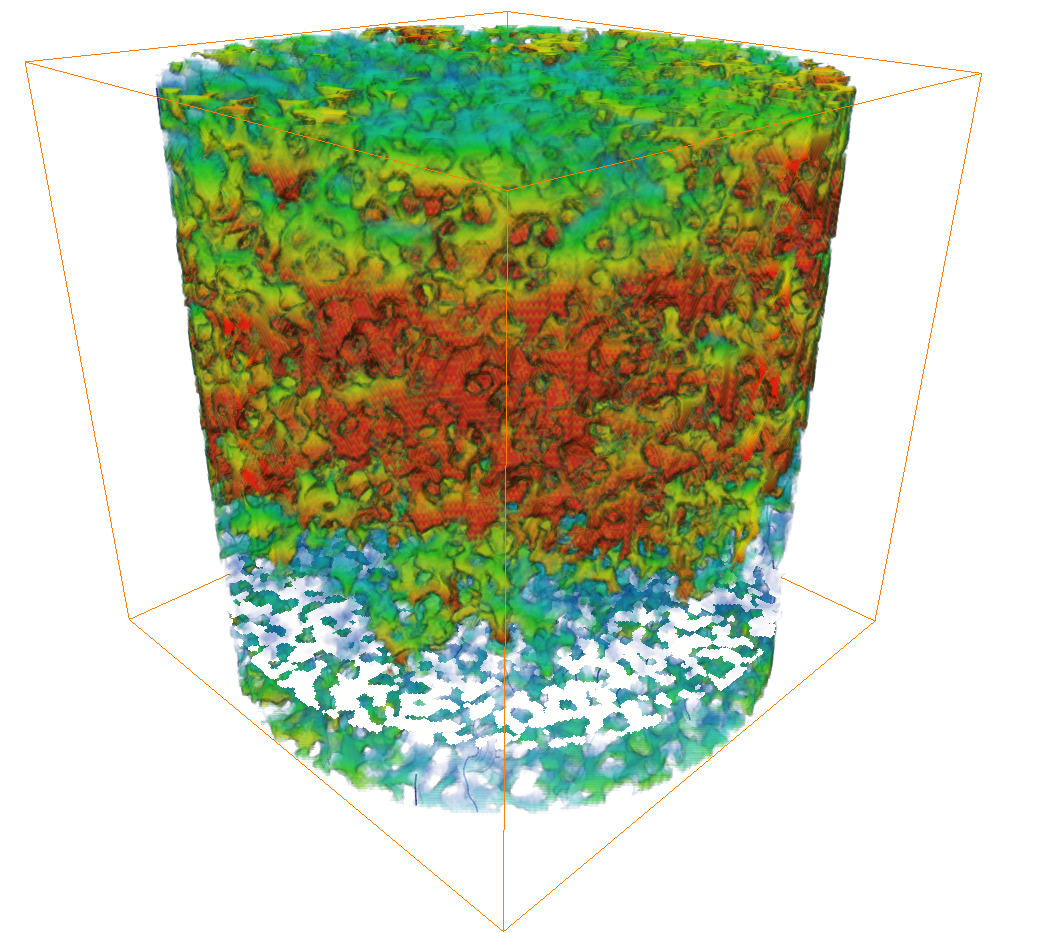

Supplement: S1 Data — (ZIP) [file pone.0296437.s001.zip › SI-Data/Data aggregation/unbiomineralization sample/unbiomineralization sample/3 quchu guli kongxi shentouya jia liuxian x-y (3D)-zhengdengzhou1 .png]

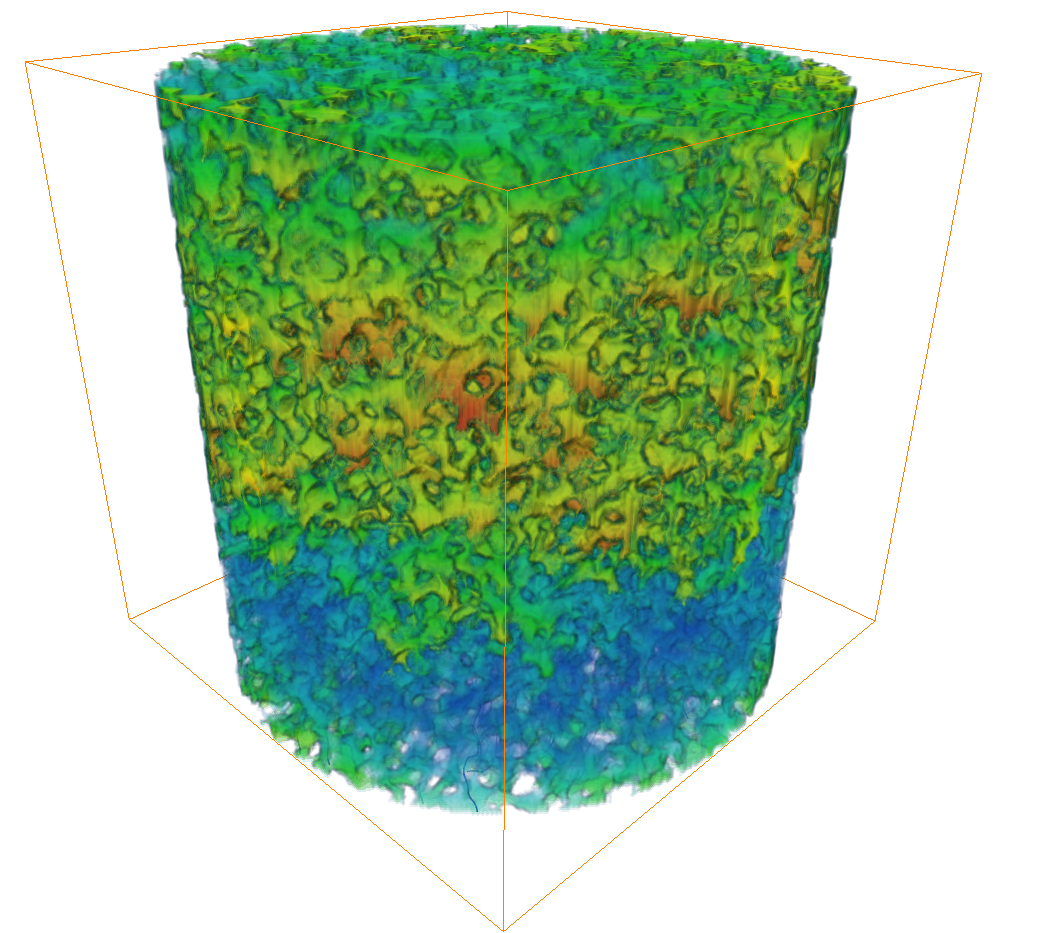

Supplement: S1 Data — (ZIP) [file pone.0296437.s001.zip › SI-Data/Data aggregation/unbiomineralization sample/unbiomineralization sample/3 quchu guli kongxi shentouya jia liuxian jia 2 x-y (3D)-zhengdengzhou1 .png]

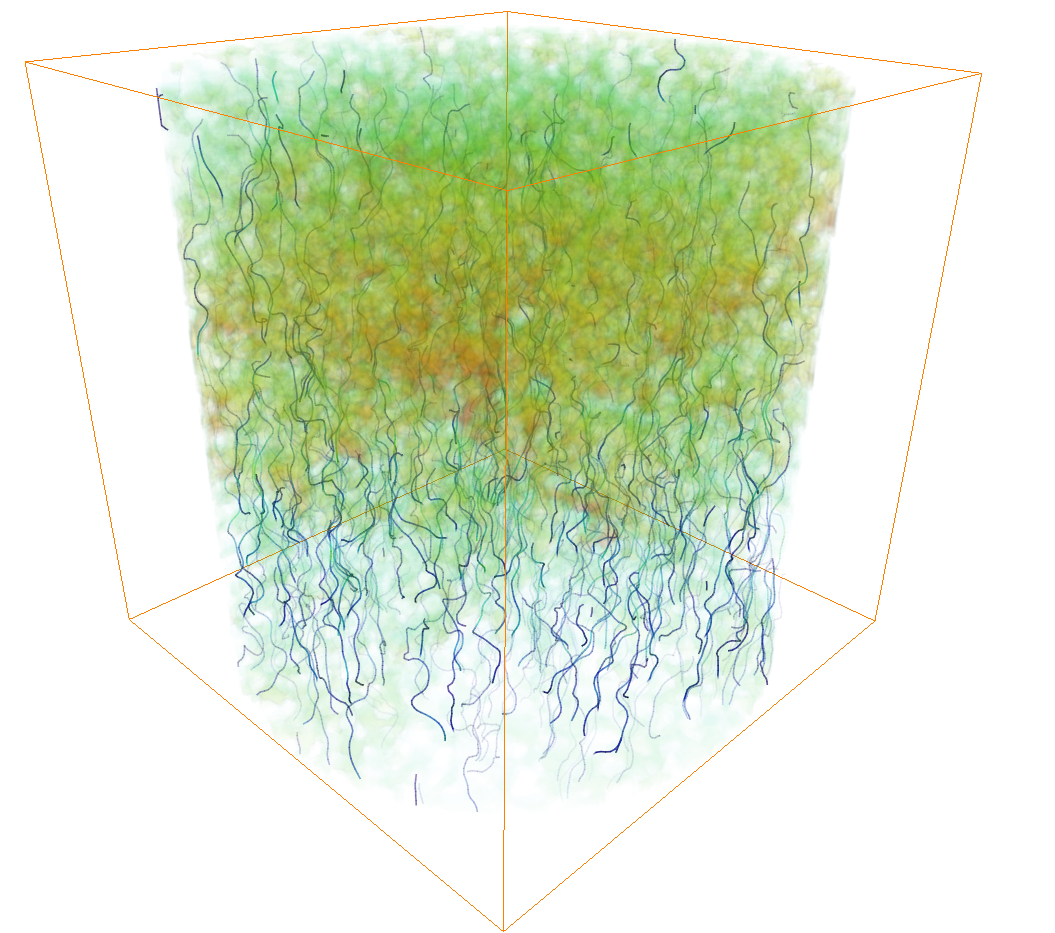

Supplement: S1 Data — (ZIP) [file pone.0296437.s001.zip › SI-Data/Data aggregation/unbiomineralization sample/unbiomineralization sample/3 quchu guli kongxi shentouya jia liuxian jia 4 x-y (3D)-zhengdengzhou1 .png]

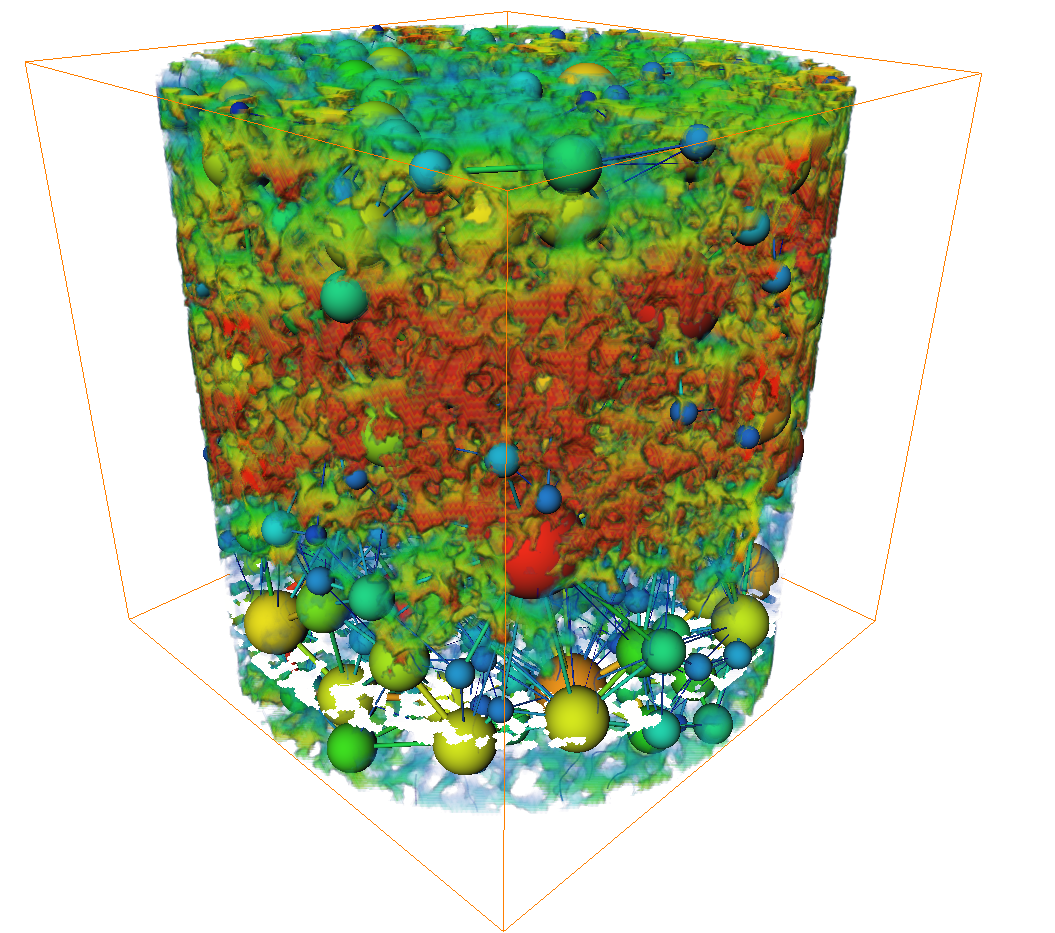

Supplement: S1 Data — (ZIP) [file pone.0296437.s001.zip › SI-Data/Data aggregation/unbiomineralization sample/unbiomineralization sample/3 quchu guli kongxi shentouya jia liuxian jia kongxiwangluomoxing x-y (3D)-zhengdengzhou1 .png]

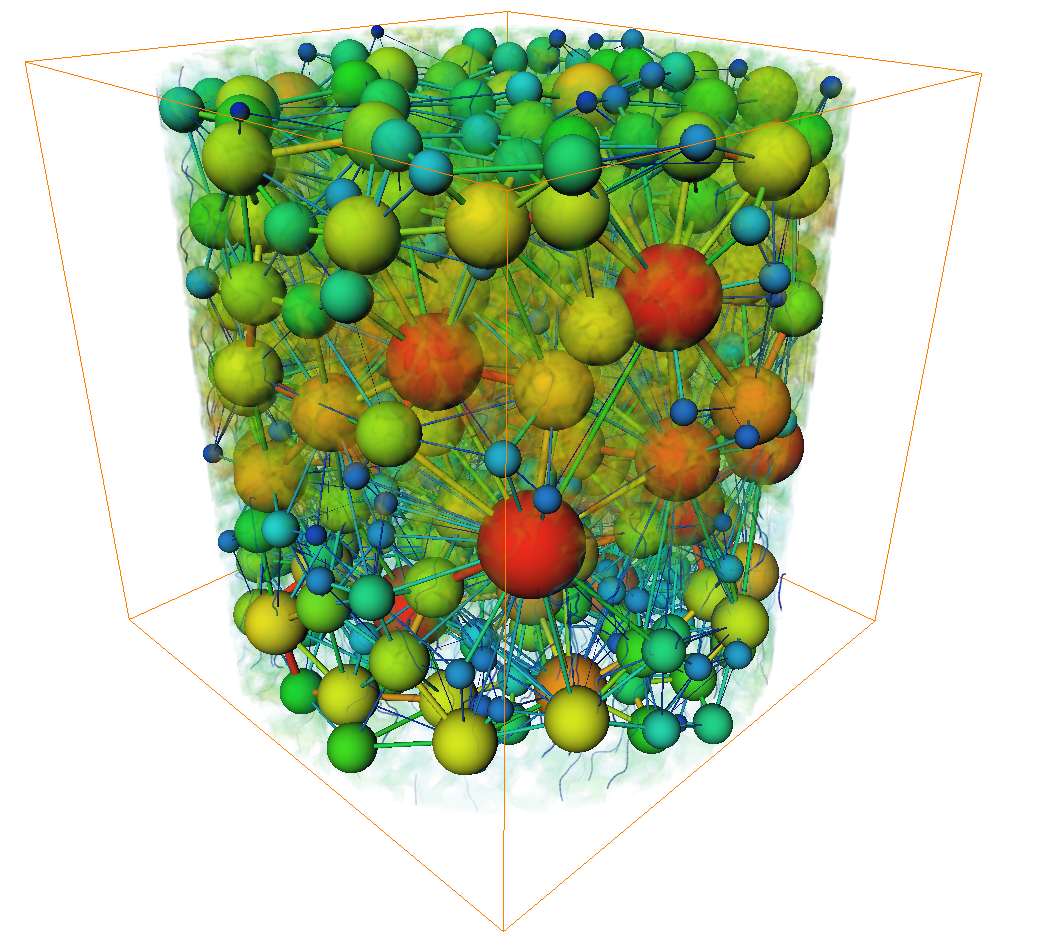

Supplement: S1 Data — (ZIP) [file pone.0296437.s001.zip › SI-Data/Data aggregation/unbiomineralization sample/unbiomineralization sample/3 quchu guli kongxi shentouya jia liuxian jia kongxiwangluomoxing4 x-y (3D)-zhengdengzhou1 .png]
