# Supplementary figures and images for: Identification and extraction of cementation patterns in sand modified by MICP: New insights at the pore scale (part 1 of 2)
Source: PLoS One. 2024 Mar 21;19(3):e0296437. doi: 10.1371/journal.pone.0296437 (PMC10956867; doi:10.1371/journal.pone.0296437)

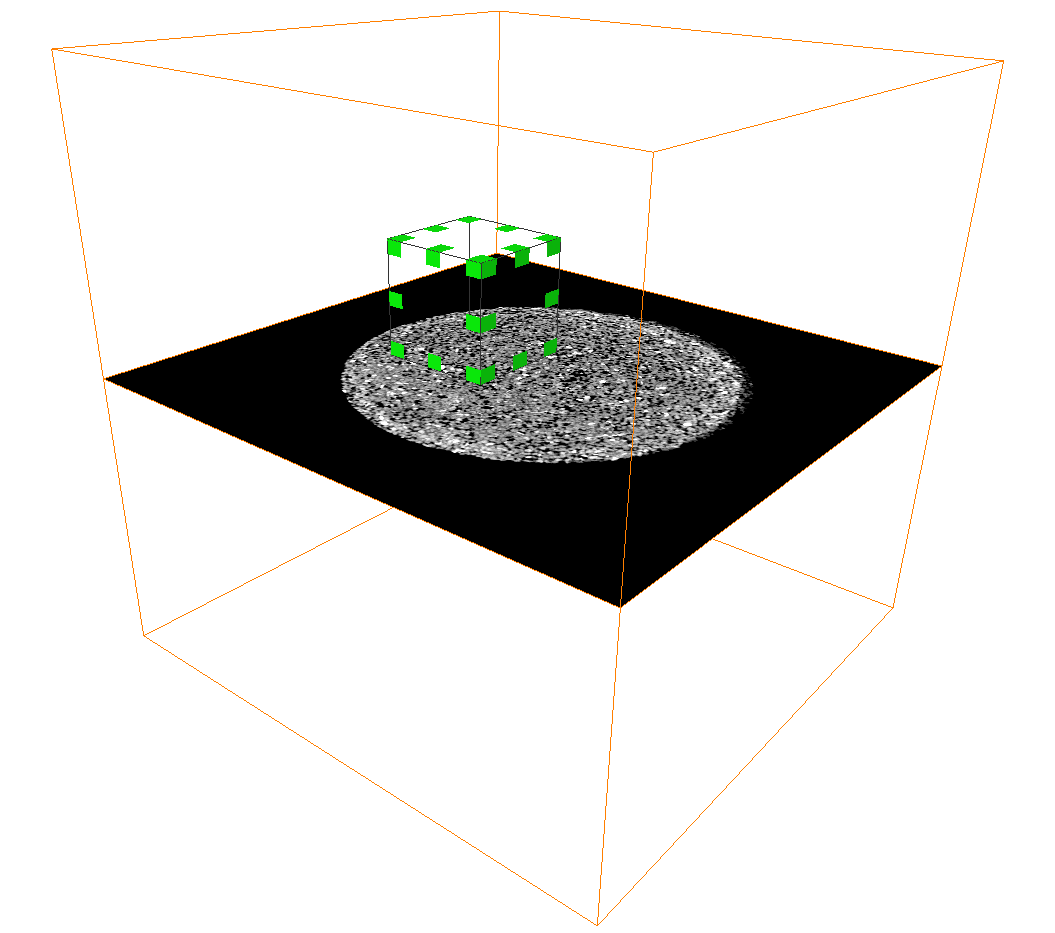

Supplement: S1 Data — (ZIP) [file pone.0296437.s001.zip › SI-Data/Data aggregation/Biomineralization sample/Biomineralization-3D reconstruction of the fig/1-1.png]

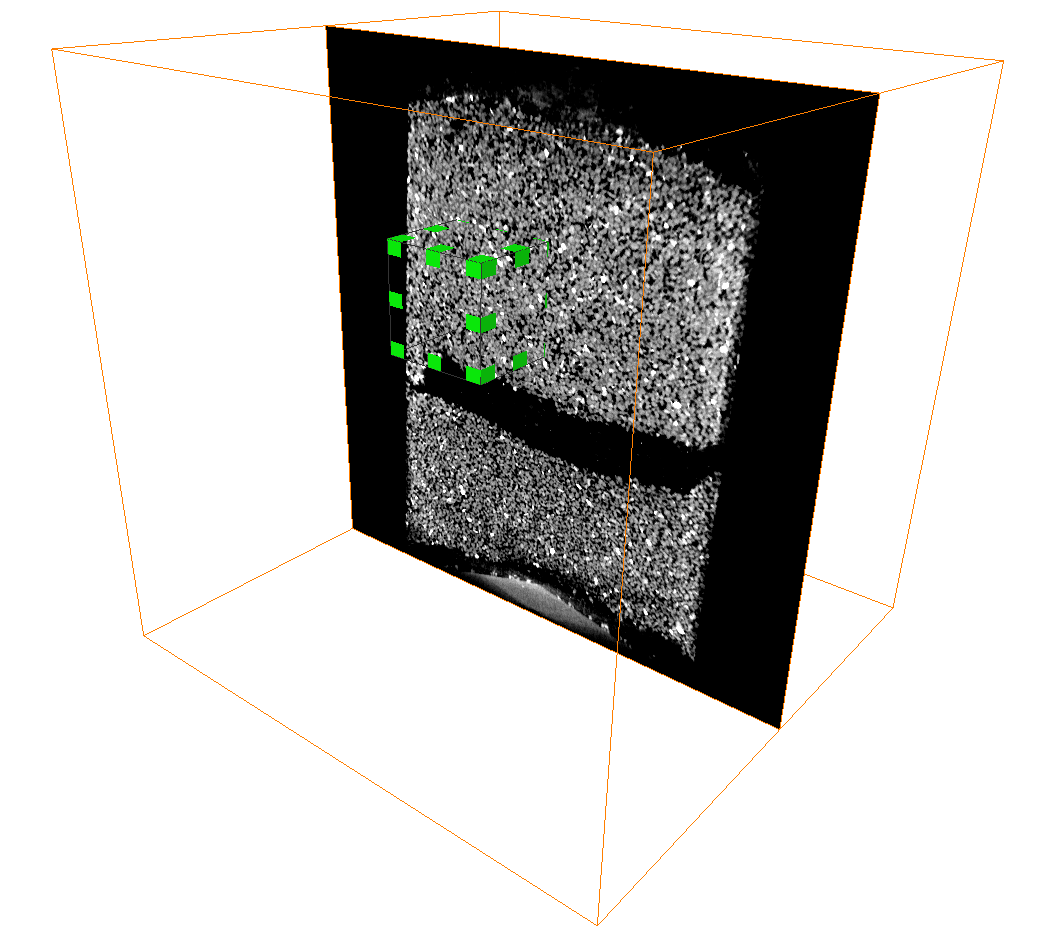

Supplement: S1 Data — (ZIP) [file pone.0296437.s001.zip › SI-Data/Data aggregation/Biomineralization sample/Biomineralization-3D reconstruction of the fig/1-3.png]

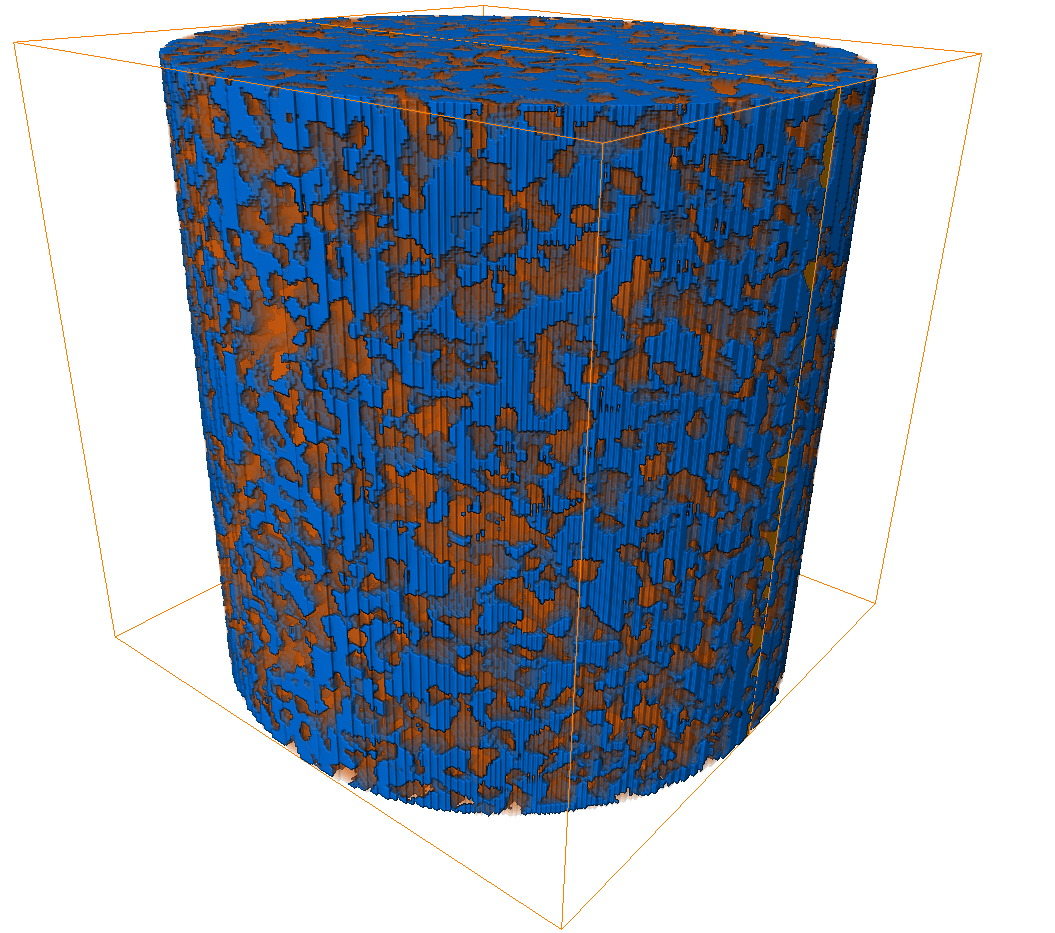

Supplement: S1 Data — (ZIP) [file pone.0296437.s001.zip › SI-Data/Data aggregation/Biomineralization sample/Biomineralization-3D reconstruction of the fig/10-1.png]

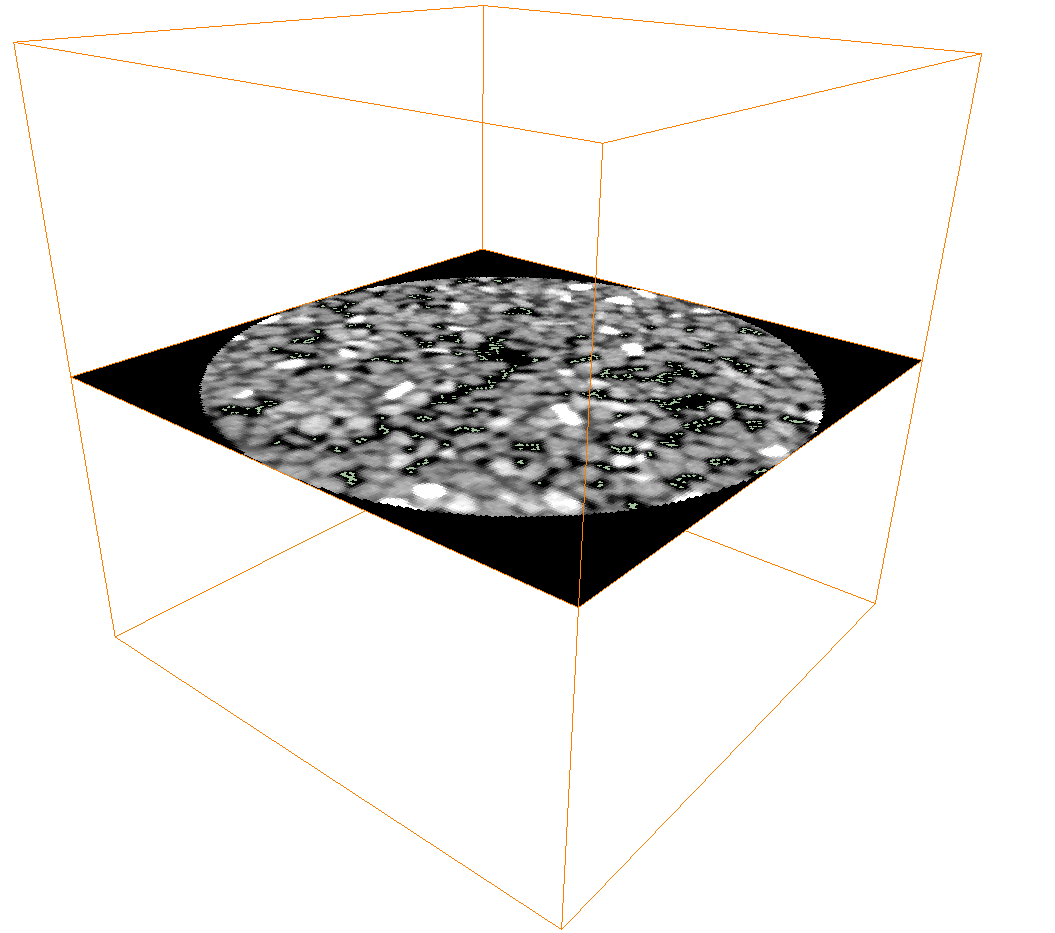

Supplement: S1 Data — (ZIP) [file pone.0296437.s001.zip › SI-Data/Data aggregation/Biomineralization sample/Biomineralization-3D reconstruction of the fig/11-1.png]

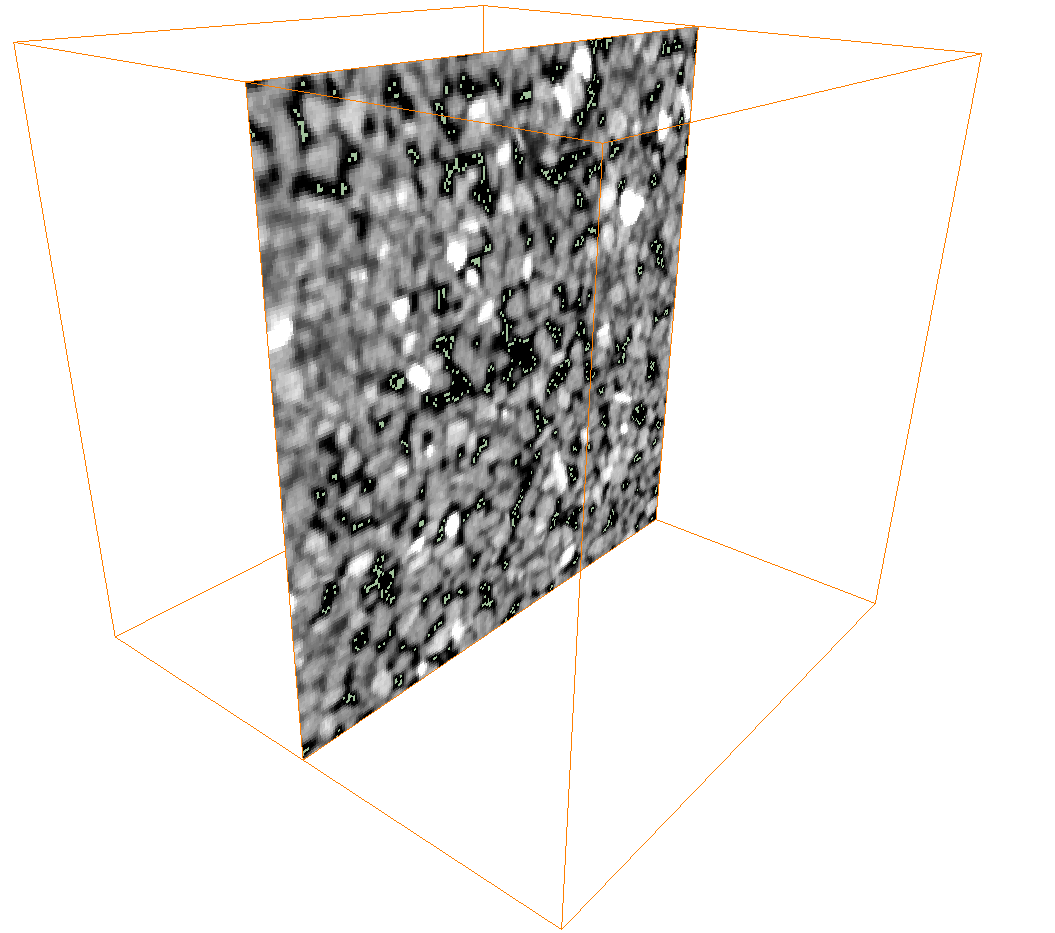

Supplement: S1 Data — (ZIP) [file pone.0296437.s001.zip › SI-Data/Data aggregation/Biomineralization sample/Biomineralization-3D reconstruction of the fig/11-2.png]

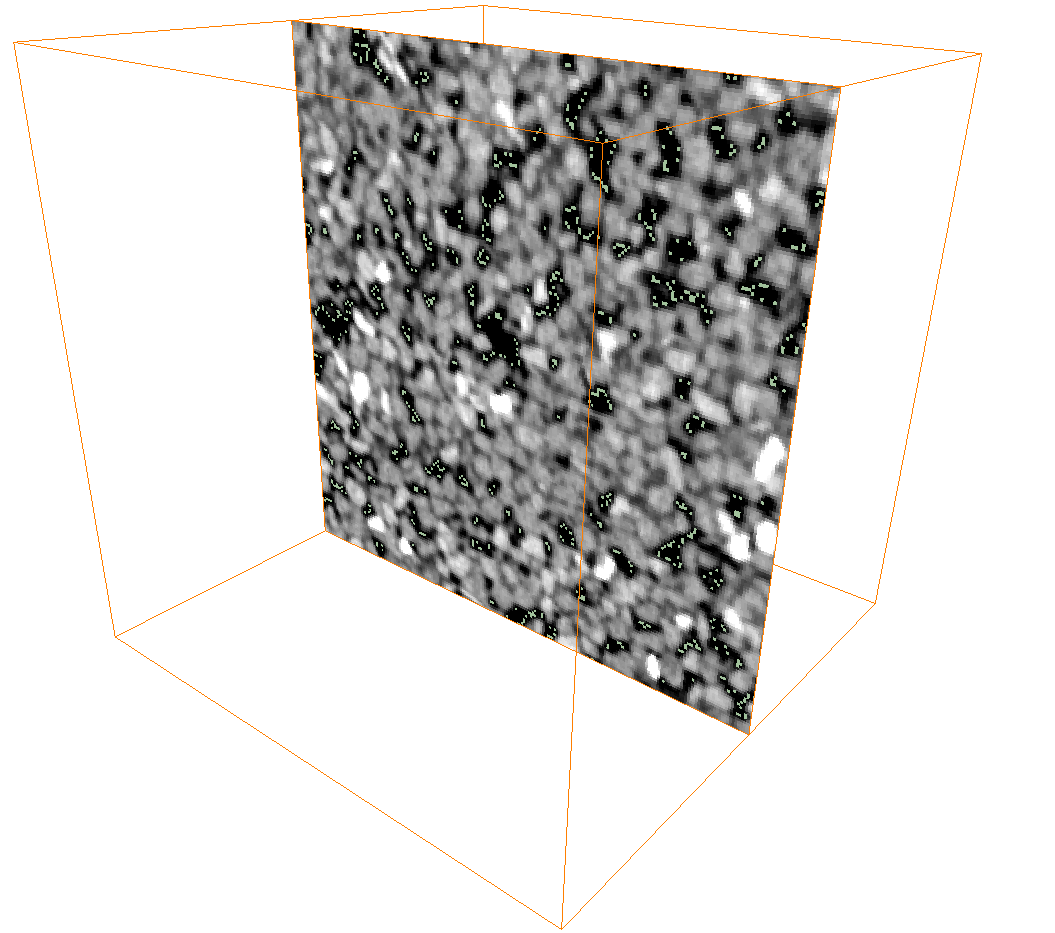

Supplement: S1 Data — (ZIP) [file pone.0296437.s001.zip › SI-Data/Data aggregation/Biomineralization sample/Biomineralization-3D reconstruction of the fig/11-3.png]

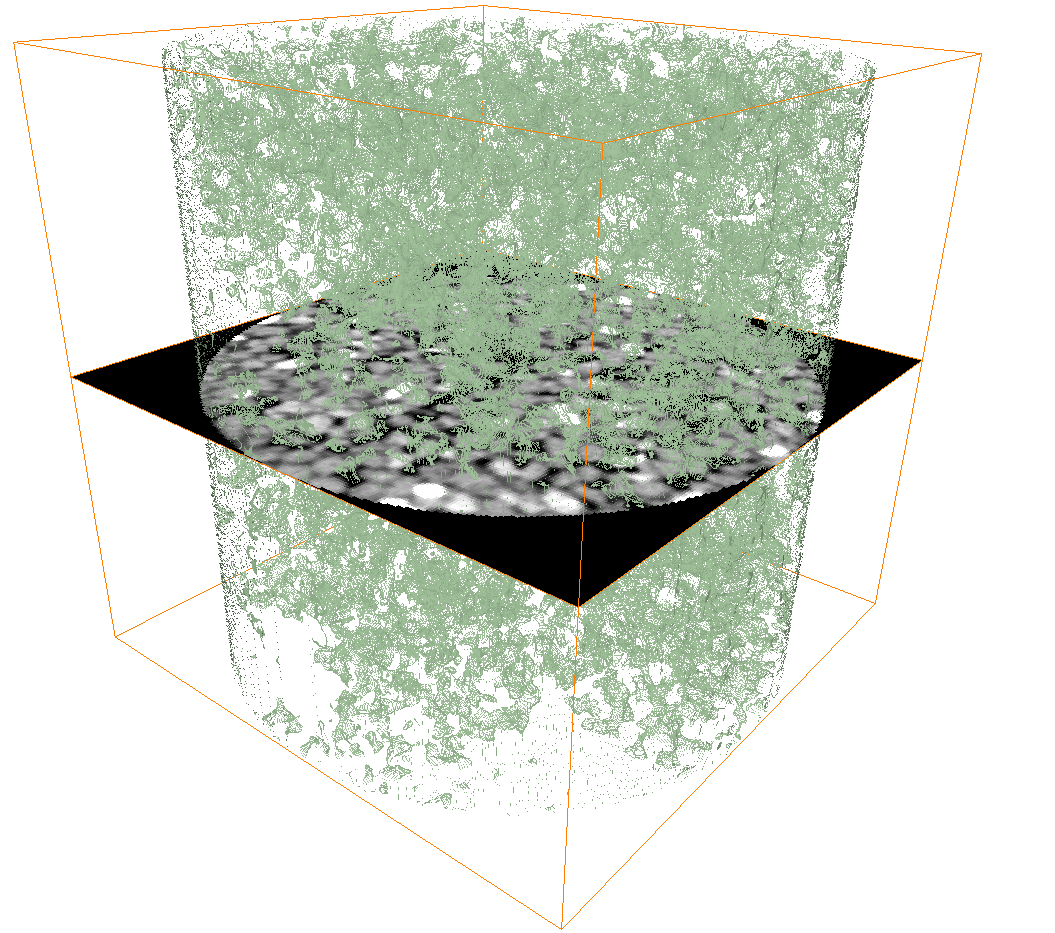

Supplement: S1 Data — (ZIP) [file pone.0296437.s001.zip › SI-Data/Data aggregation/Biomineralization sample/Biomineralization-3D reconstruction of the fig/12-1.png]

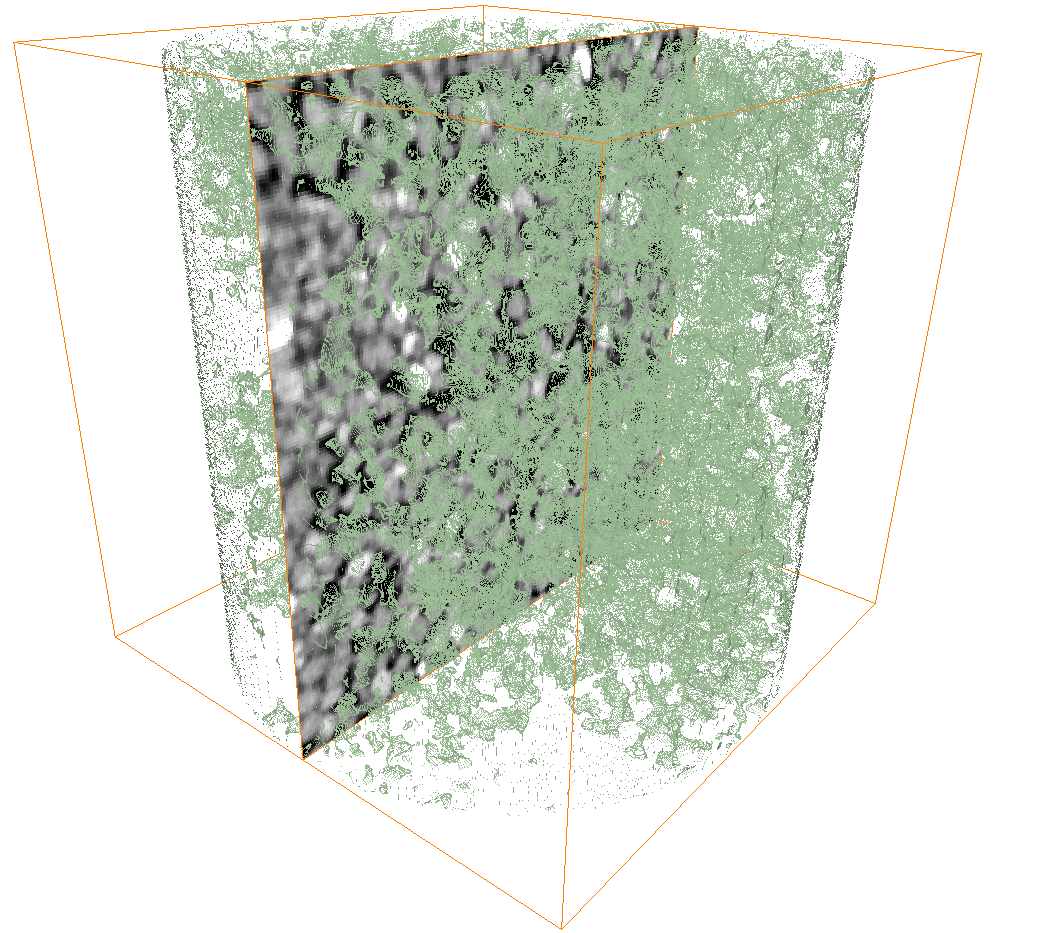

Supplement: S1 Data — (ZIP) [file pone.0296437.s001.zip › SI-Data/Data aggregation/Biomineralization sample/Biomineralization-3D reconstruction of the fig/12-2.png]

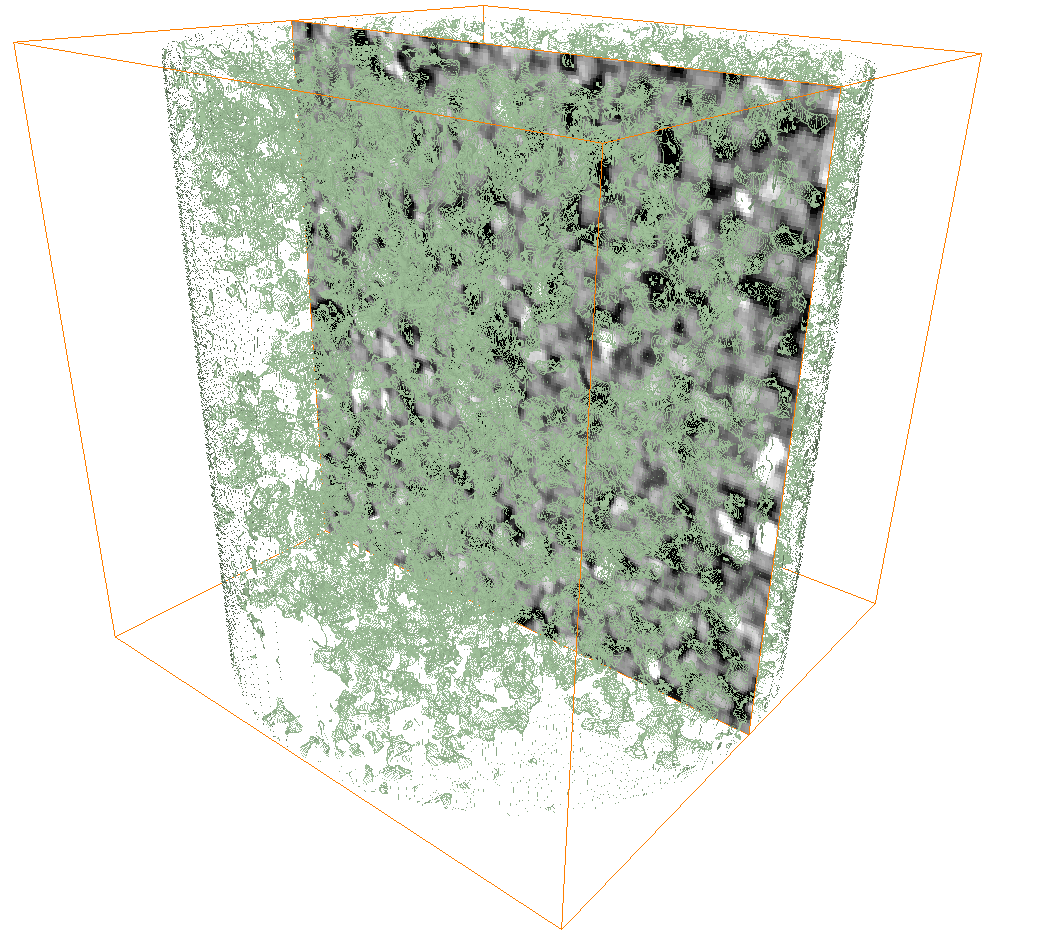

Supplement: S1 Data — (ZIP) [file pone.0296437.s001.zip › SI-Data/Data aggregation/Biomineralization sample/Biomineralization-3D reconstruction of the fig/12-3.png]

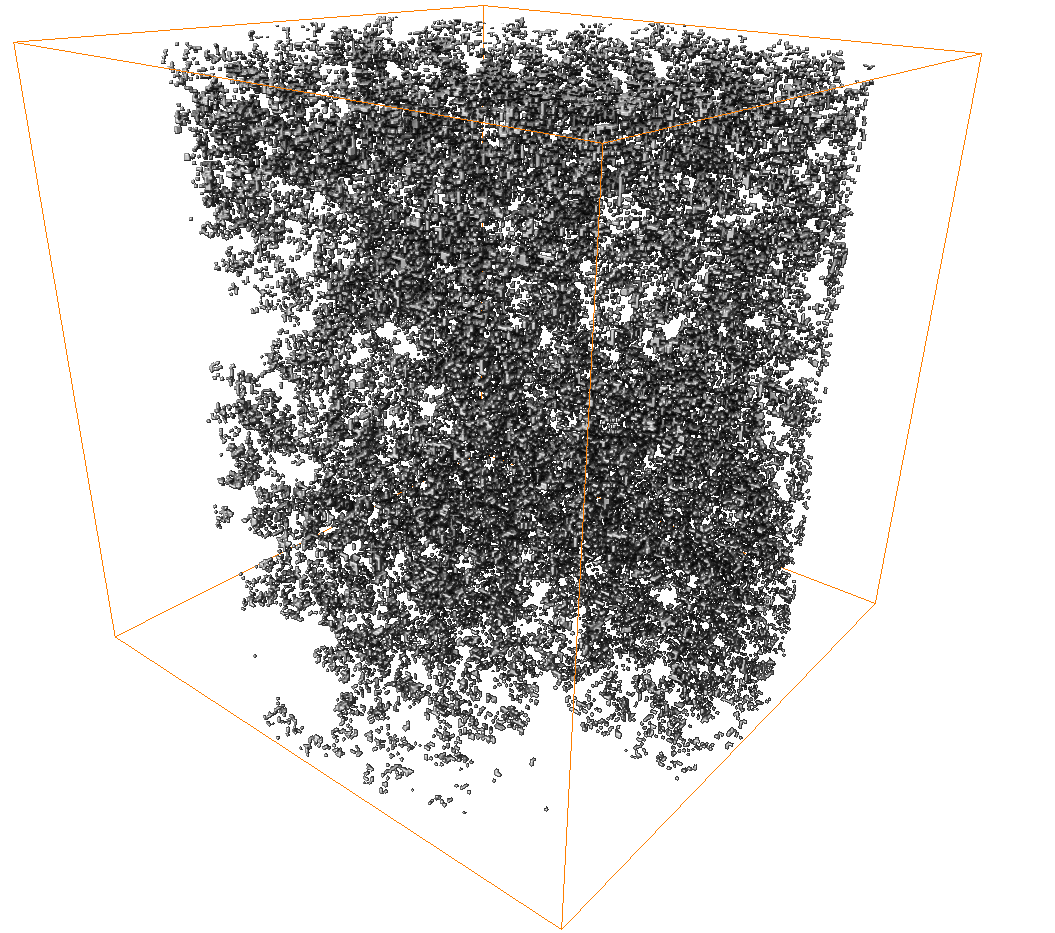

Supplement: S1 Data — (ZIP) [file pone.0296437.s001.zip › SI-Data/Data aggregation/Biomineralization sample/Biomineralization-3D reconstruction of the fig/13.png]

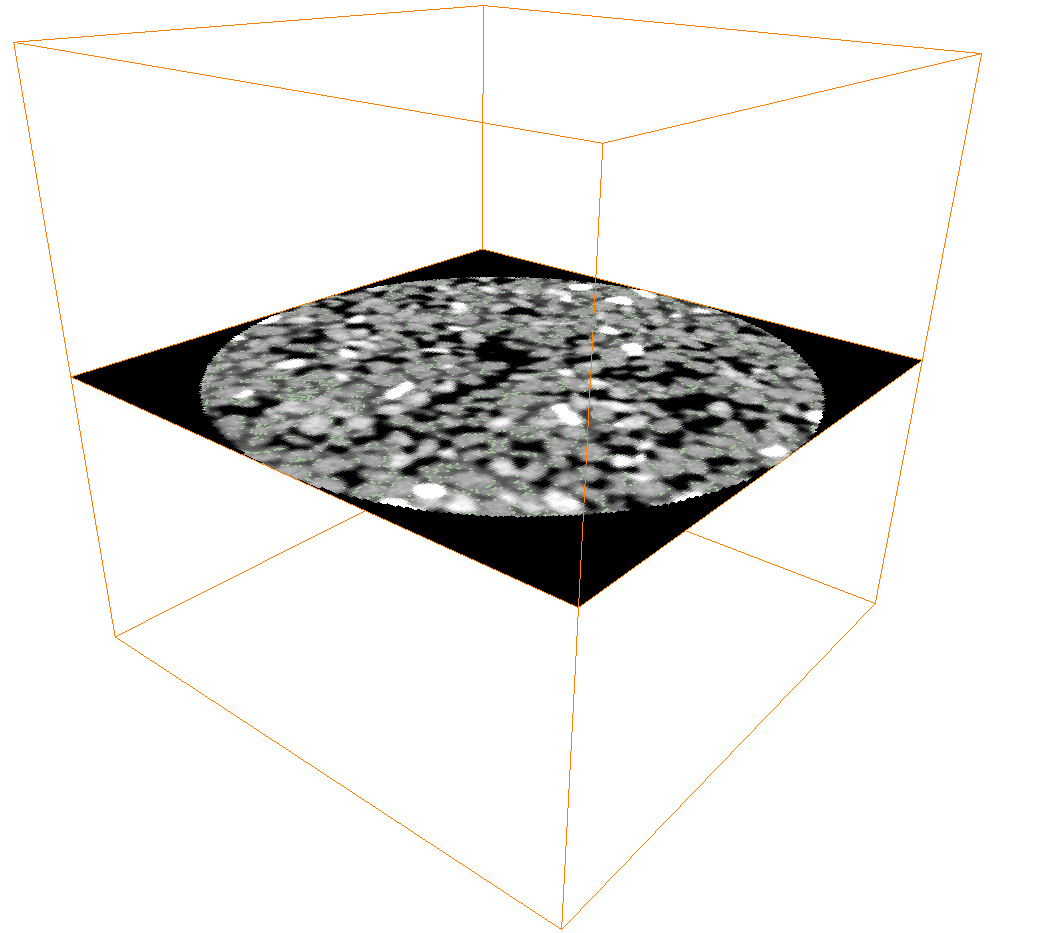

Supplement: S1 Data — (ZIP) [file pone.0296437.s001.zip › SI-Data/Data aggregation/Biomineralization sample/Biomineralization-3D reconstruction of the fig/14-1.png]

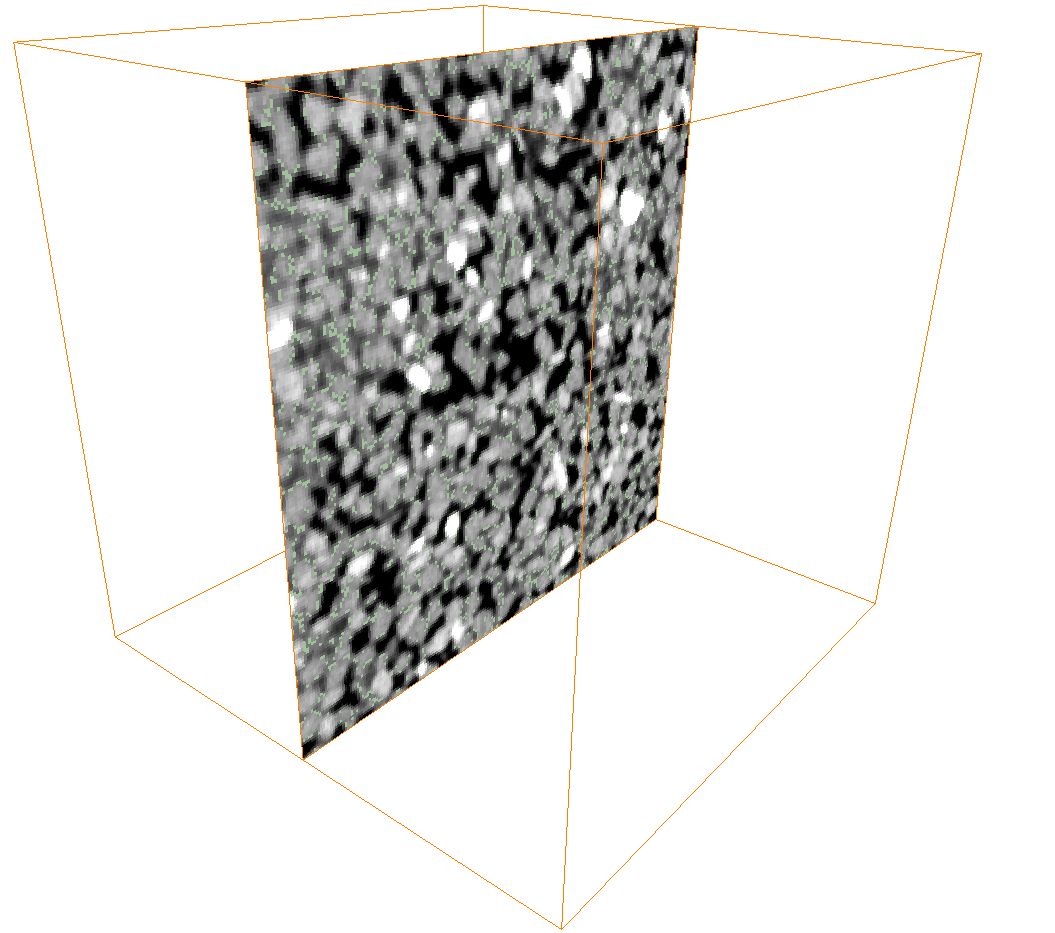

Supplement: S1 Data — (ZIP) [file pone.0296437.s001.zip › SI-Data/Data aggregation/Biomineralization sample/Biomineralization-3D reconstruction of the fig/14-2.png]

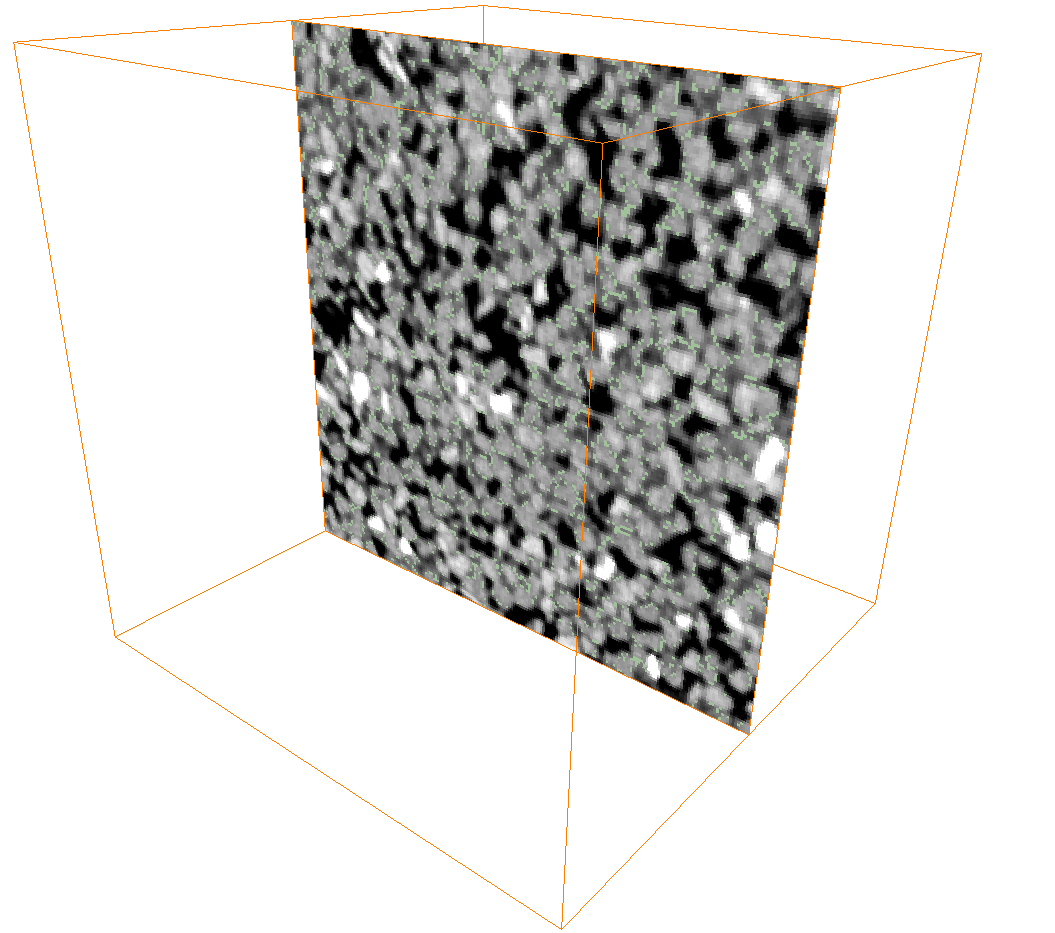

Supplement: S1 Data — (ZIP) [file pone.0296437.s001.zip › SI-Data/Data aggregation/Biomineralization sample/Biomineralization-3D reconstruction of the fig/14-3.png]

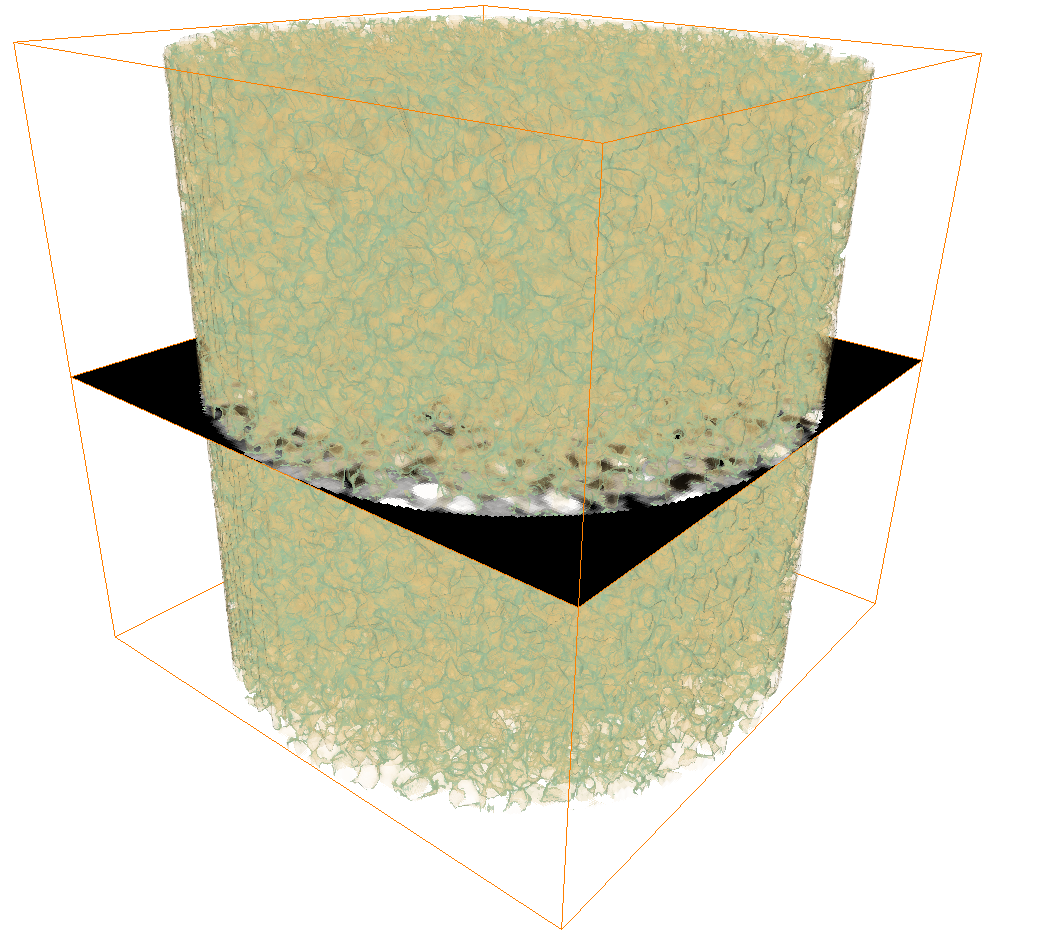

Supplement: S1 Data — (ZIP) [file pone.0296437.s001.zip › SI-Data/Data aggregation/Biomineralization sample/Biomineralization-3D reconstruction of the fig/15-1.png]

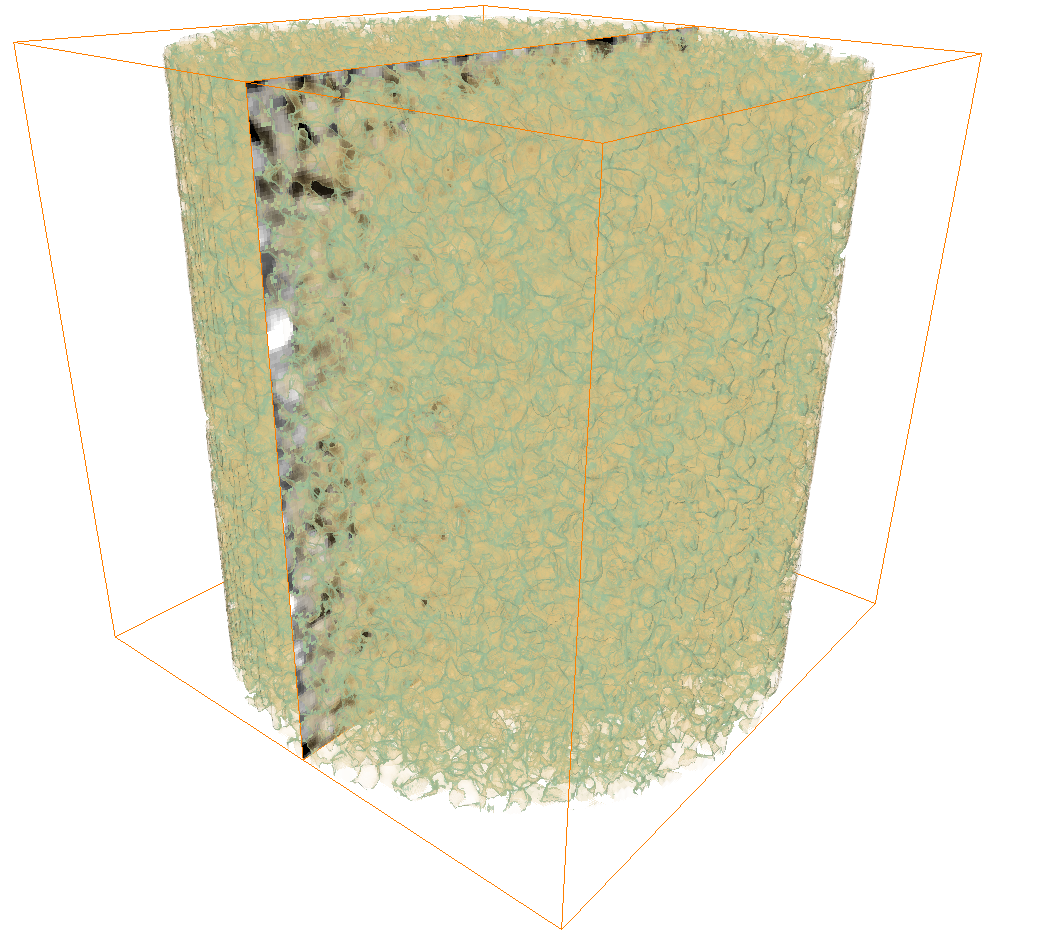

Supplement: S1 Data — (ZIP) [file pone.0296437.s001.zip › SI-Data/Data aggregation/Biomineralization sample/Biomineralization-3D reconstruction of the fig/15-2.png]

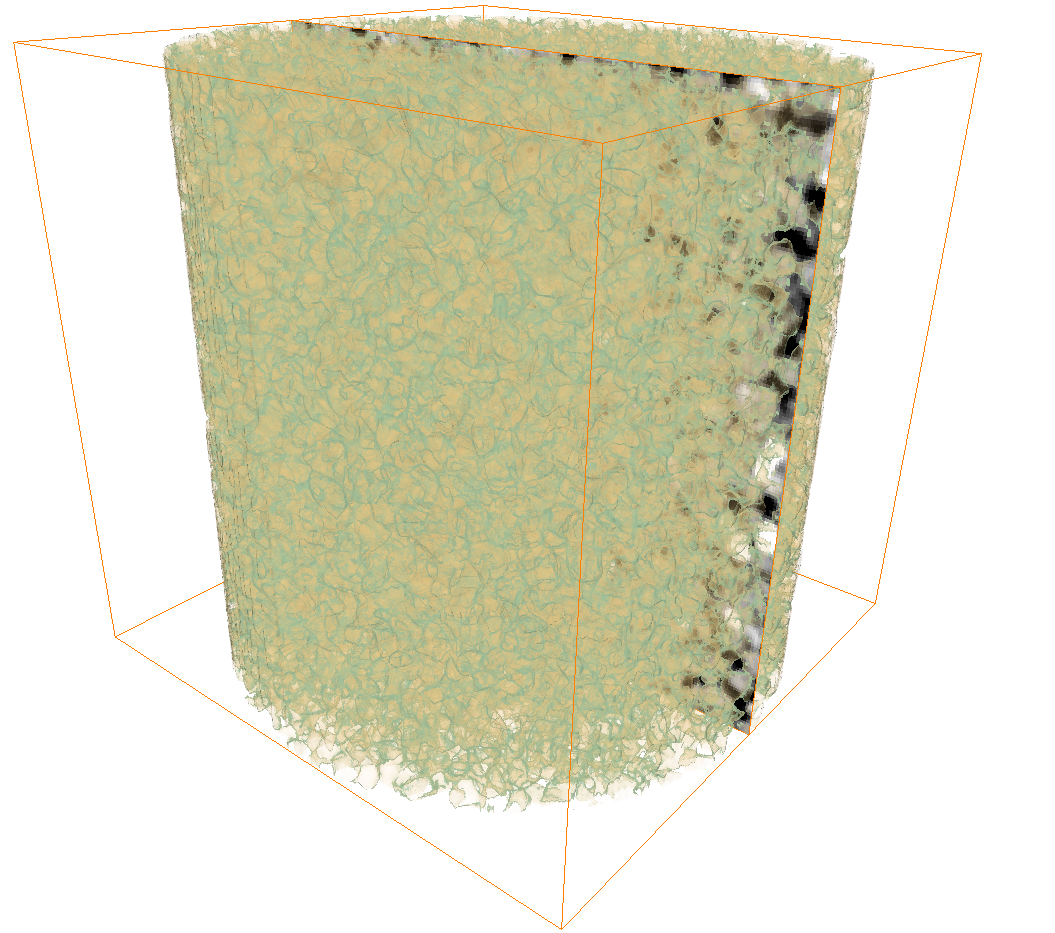

Supplement: S1 Data — (ZIP) [file pone.0296437.s001.zip › SI-Data/Data aggregation/Biomineralization sample/Biomineralization-3D reconstruction of the fig/15-3.png]

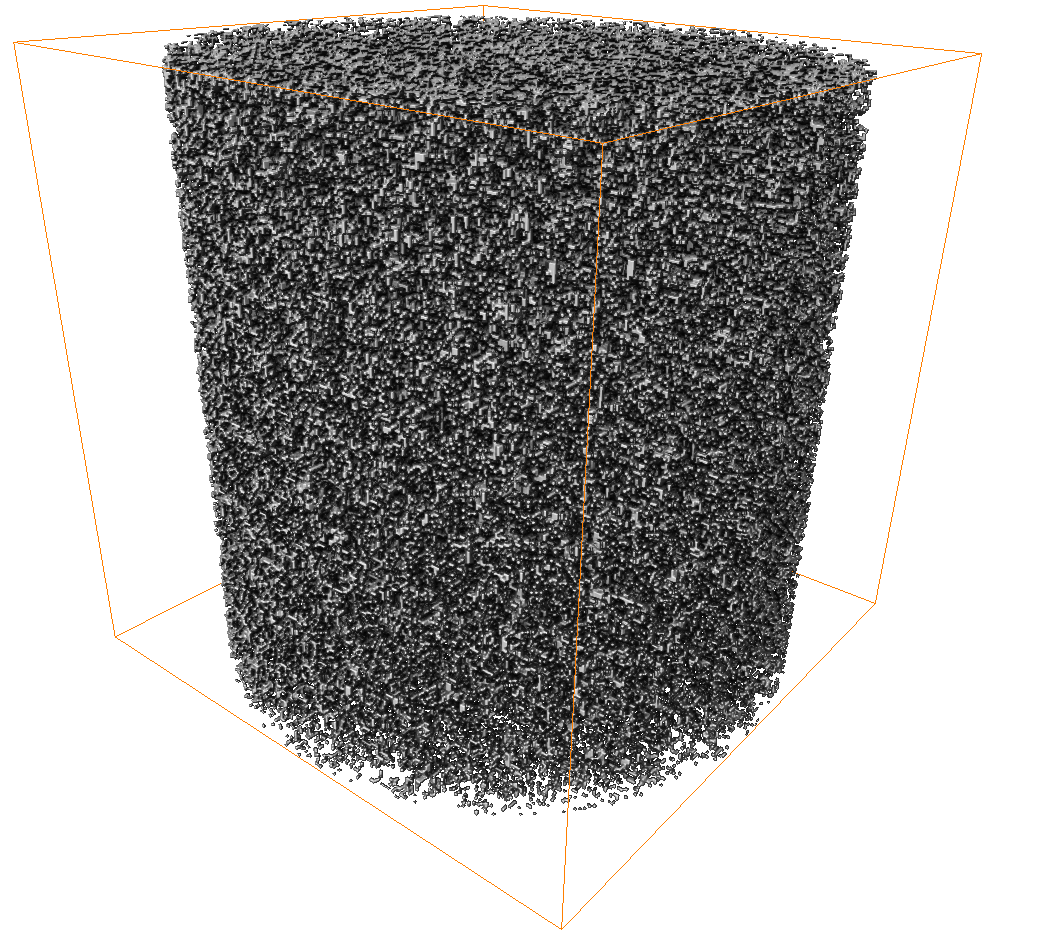

Supplement: S1 Data — (ZIP) [file pone.0296437.s001.zip › SI-Data/Data aggregation/Biomineralization sample/Biomineralization-3D reconstruction of the fig/16-1.png]

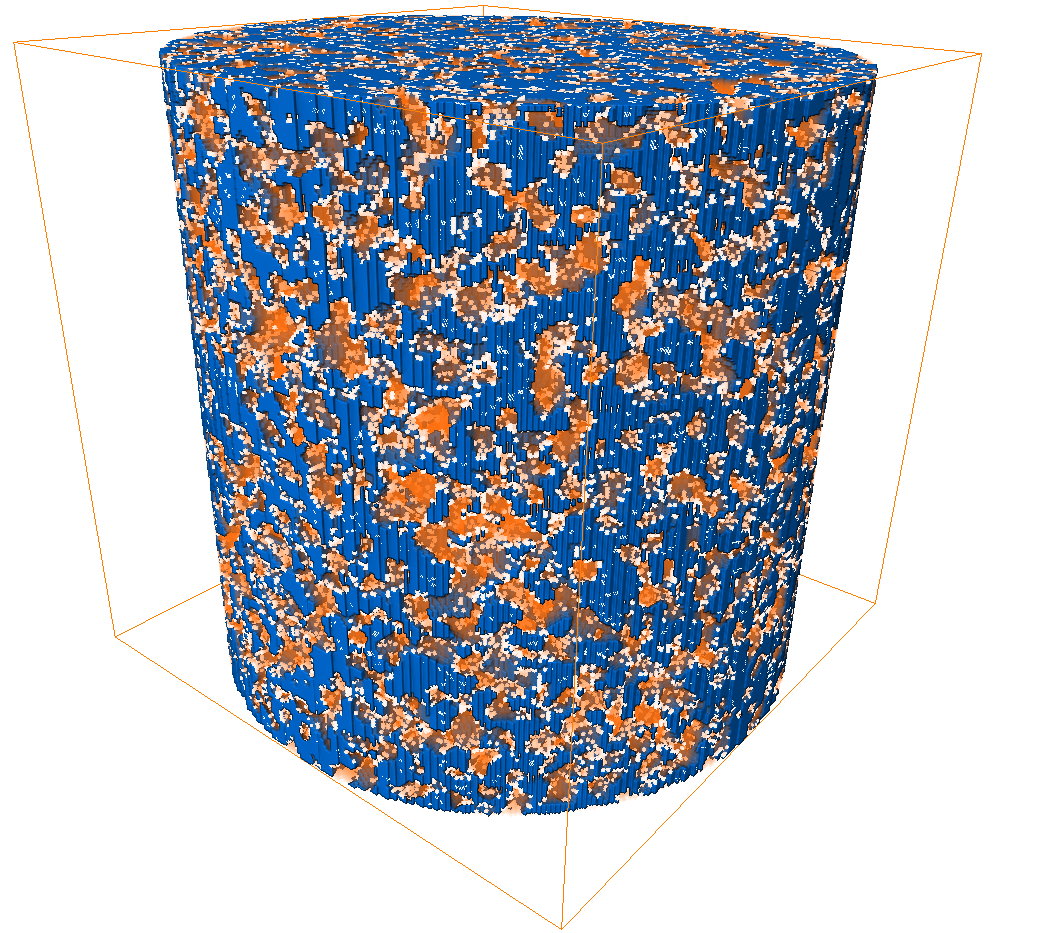

Supplement: S1 Data — (ZIP) [file pone.0296437.s001.zip › SI-Data/Data aggregation/Biomineralization sample/Biomineralization-3D reconstruction of the fig/17-3.png]

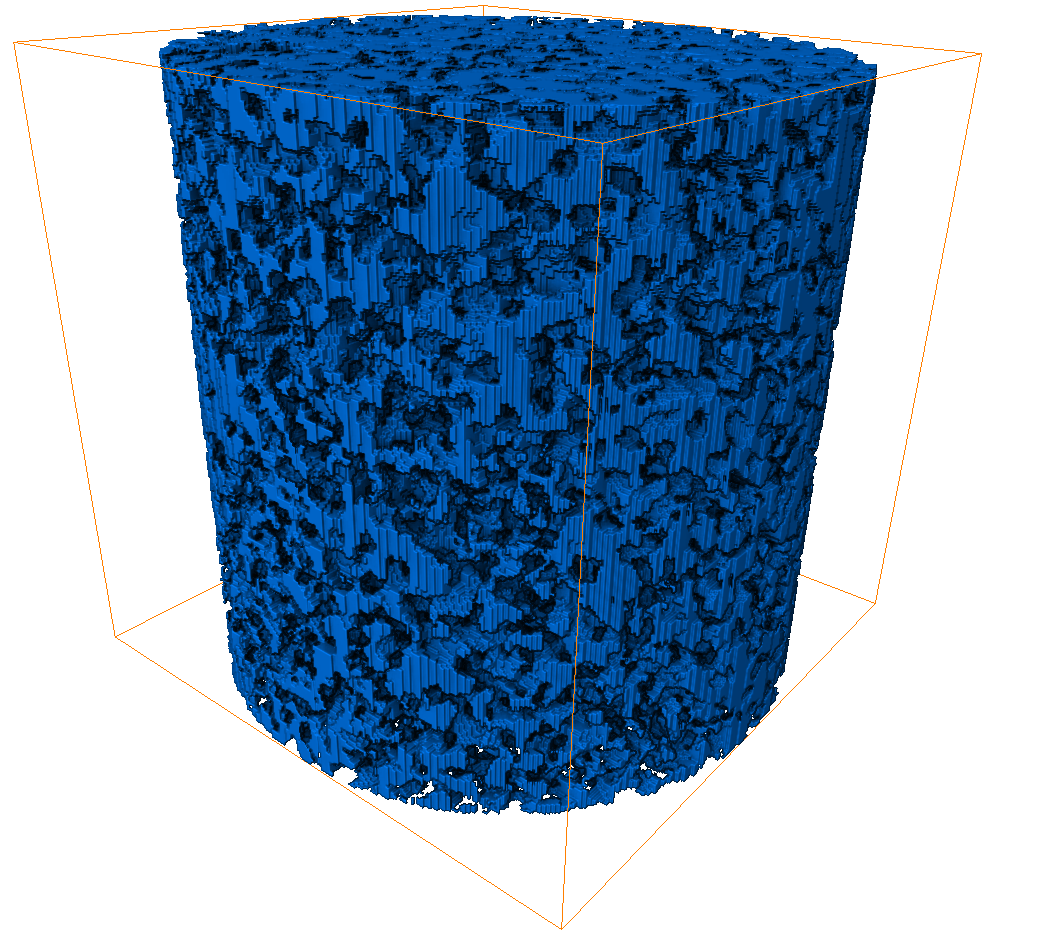

Supplement: S1 Data — (ZIP) [file pone.0296437.s001.zip › SI-Data/Data aggregation/Biomineralization sample/Biomineralization-3D reconstruction of the fig/18-1((Removal of isolated pores)).png]

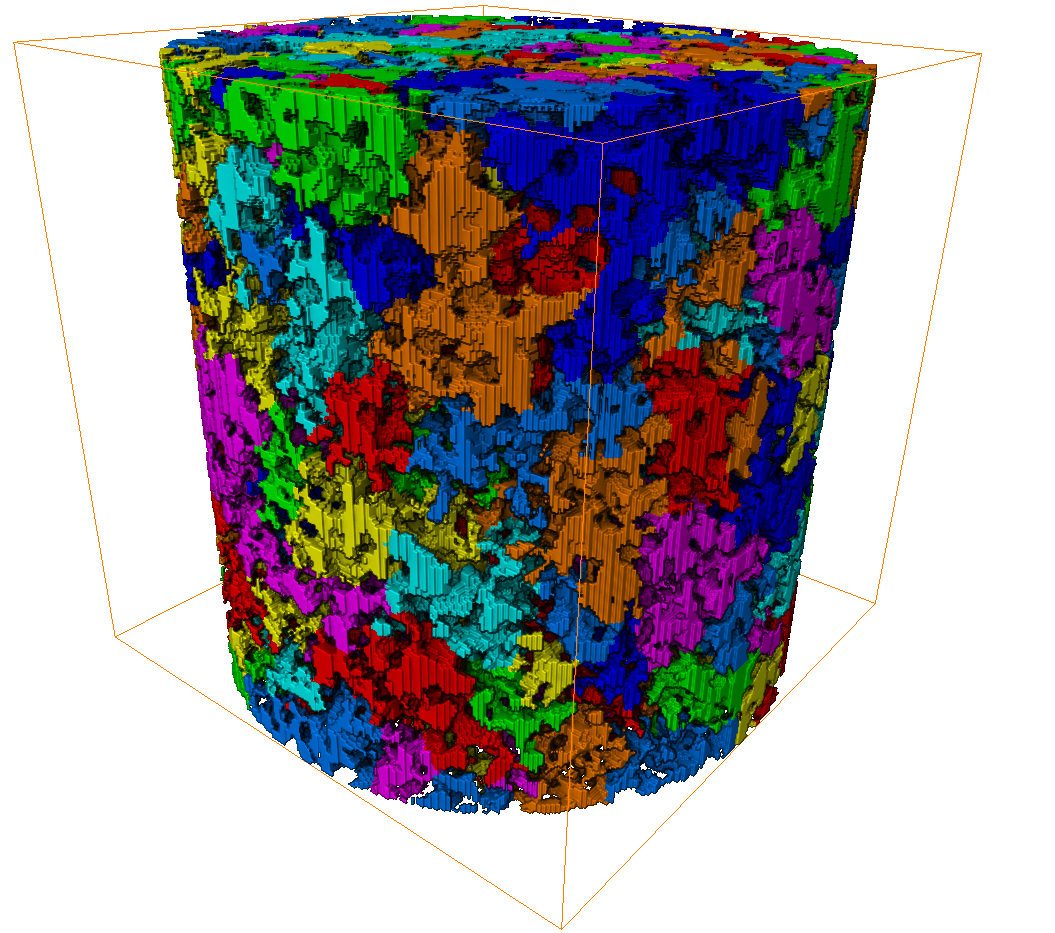

Supplement: S1 Data — (ZIP) [file pone.0296437.s001.zip › SI-Data/Data aggregation/Biomineralization sample/Biomineralization-3D reconstruction of the fig/19-1((Removal of isolated pores)).png]

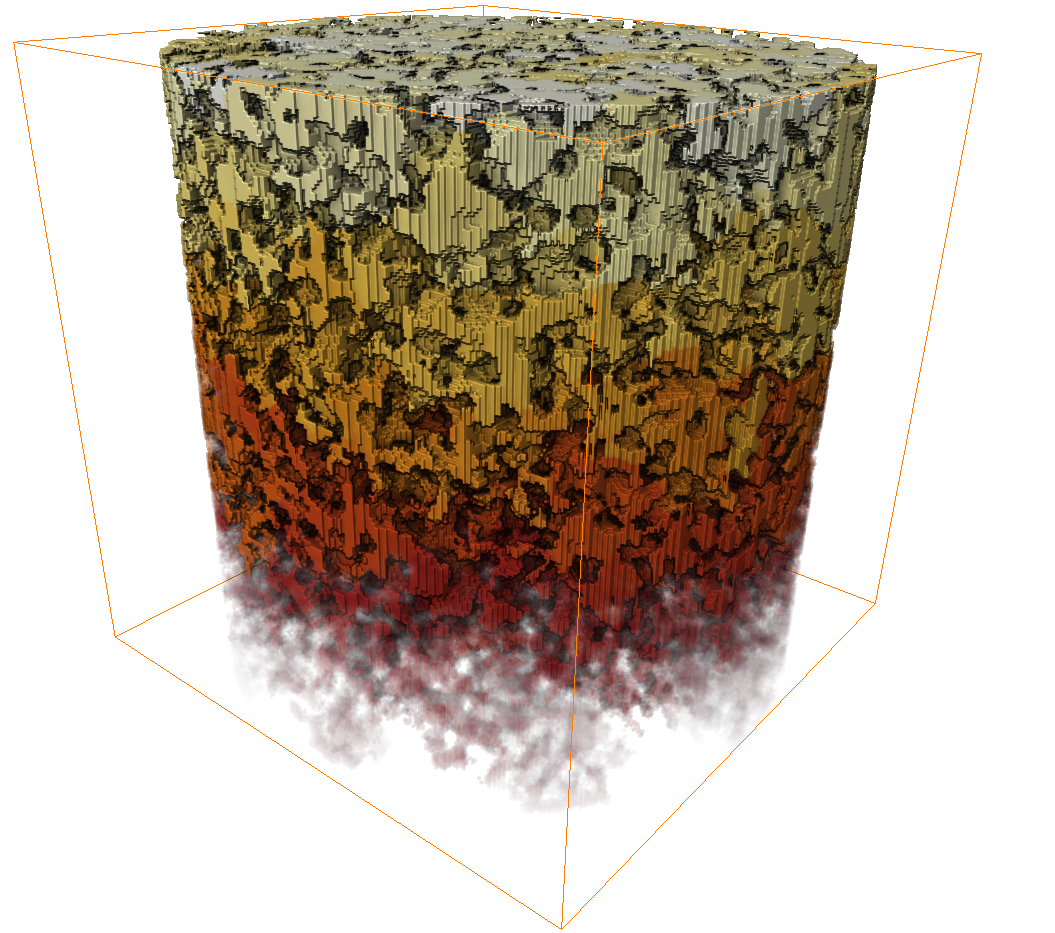

Supplement: S1 Data — (ZIP) [file pone.0296437.s001.zip › SI-Data/Data aggregation/Biomineralization sample/Biomineralization-3D reconstruction of the fig/19-2((Removal of isolated pores)).png]

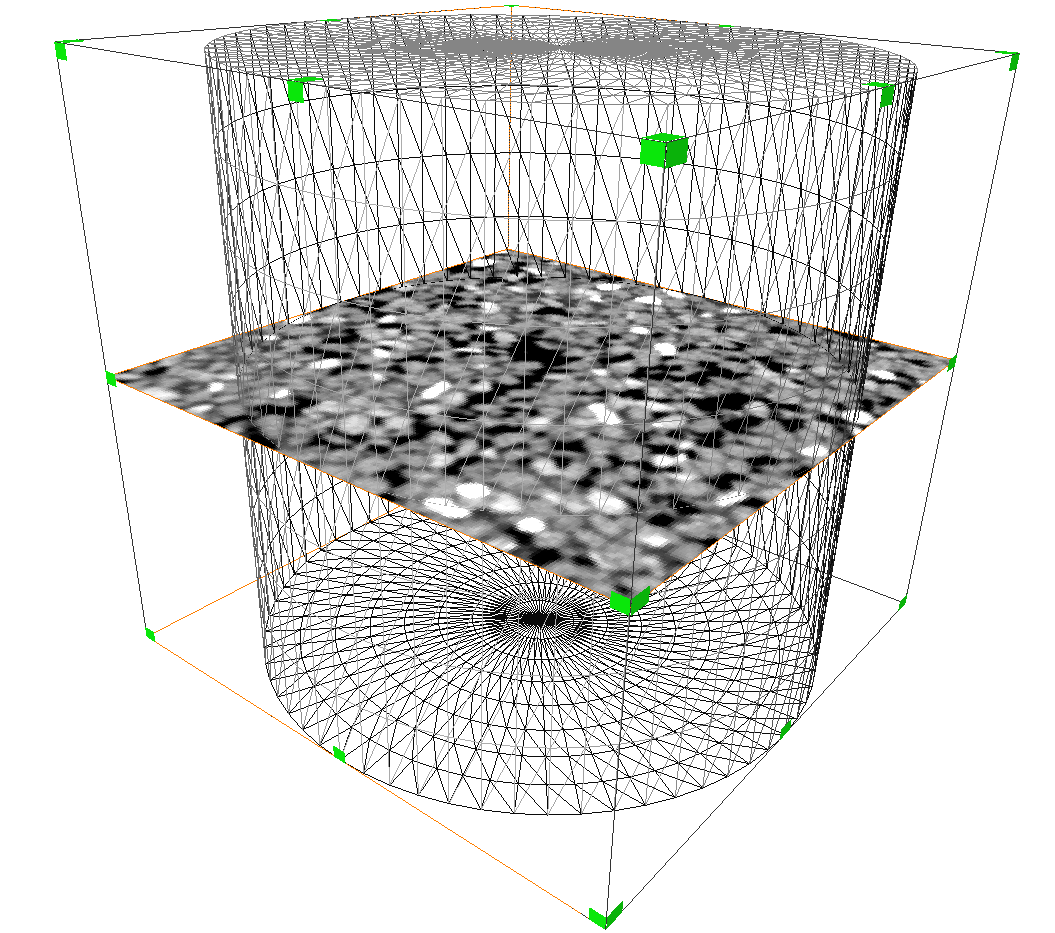

Supplement: S1 Data — (ZIP) [file pone.0296437.s001.zip › SI-Data/Data aggregation/Biomineralization sample/Biomineralization-3D reconstruction of the fig/2-1.png]

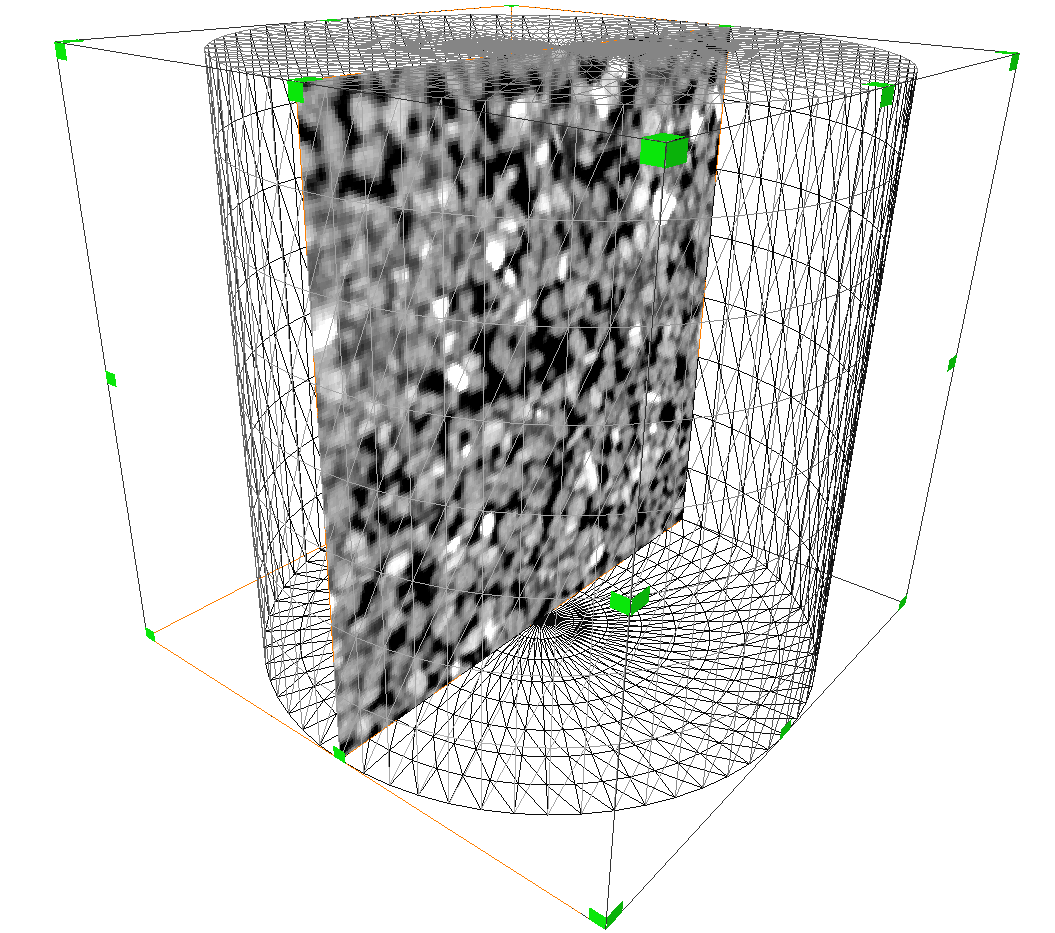

Supplement: S1 Data — (ZIP) [file pone.0296437.s001.zip › SI-Data/Data aggregation/Biomineralization sample/Biomineralization-3D reconstruction of the fig/2-2.png]

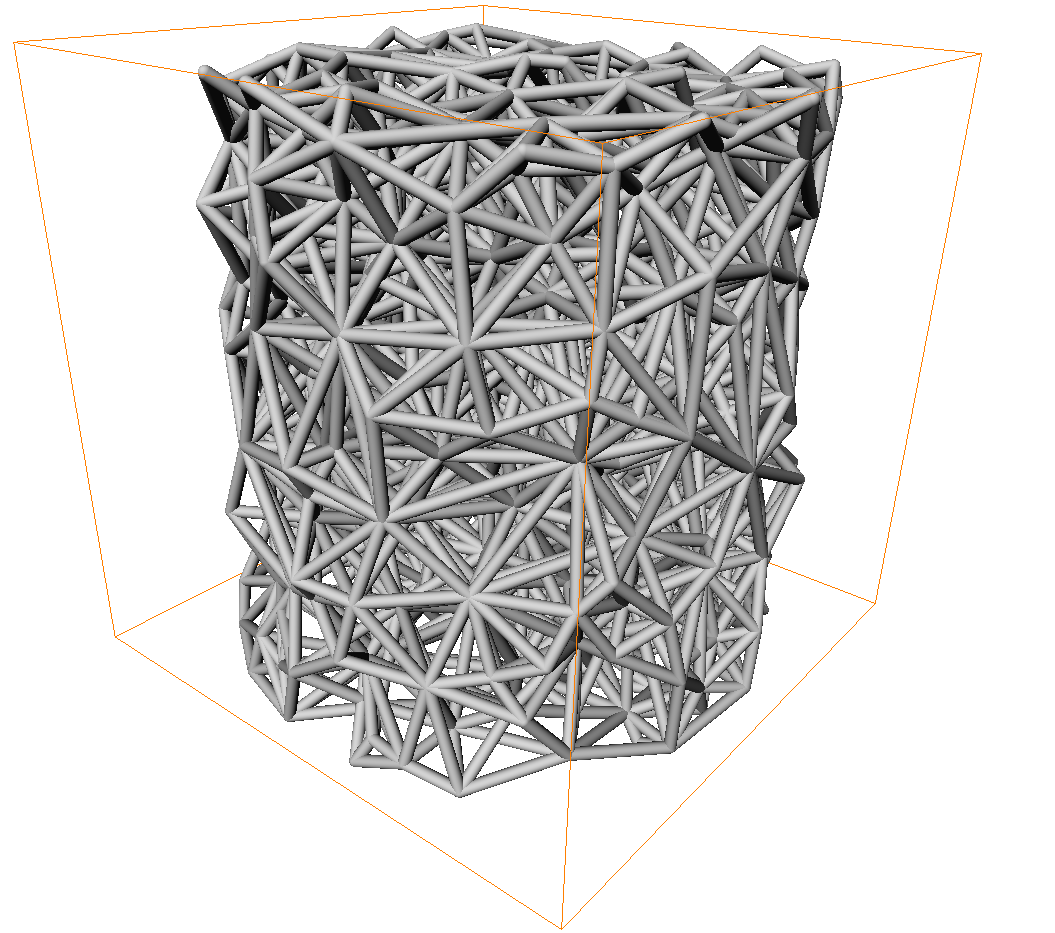

Supplement: S1 Data — (ZIP) [file pone.0296437.s001.zip › SI-Data/Data aggregation/Biomineralization sample/Biomineralization-3D reconstruction of the fig/20-1.png]

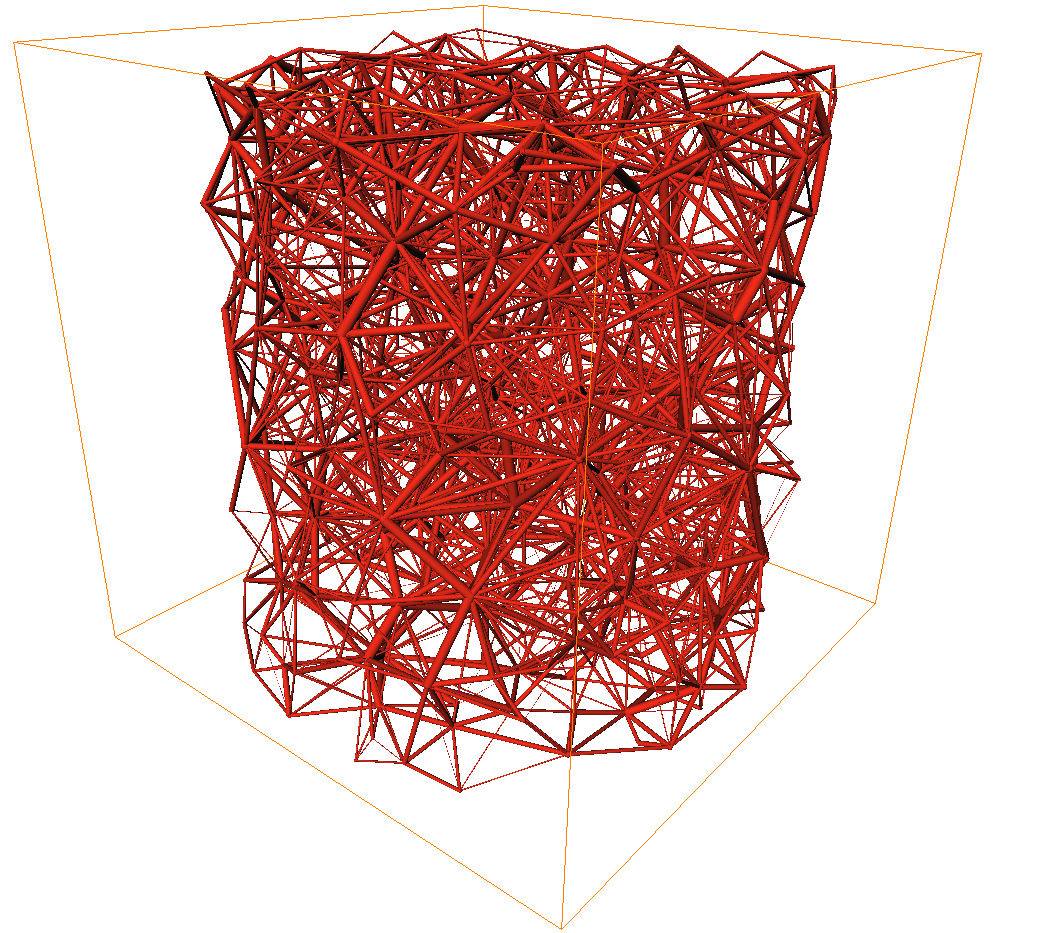

Supplement: S1 Data — (ZIP) [file pone.0296437.s001.zip › SI-Data/Data aggregation/Biomineralization sample/Biomineralization-3D reconstruction of the fig/20-2.png]

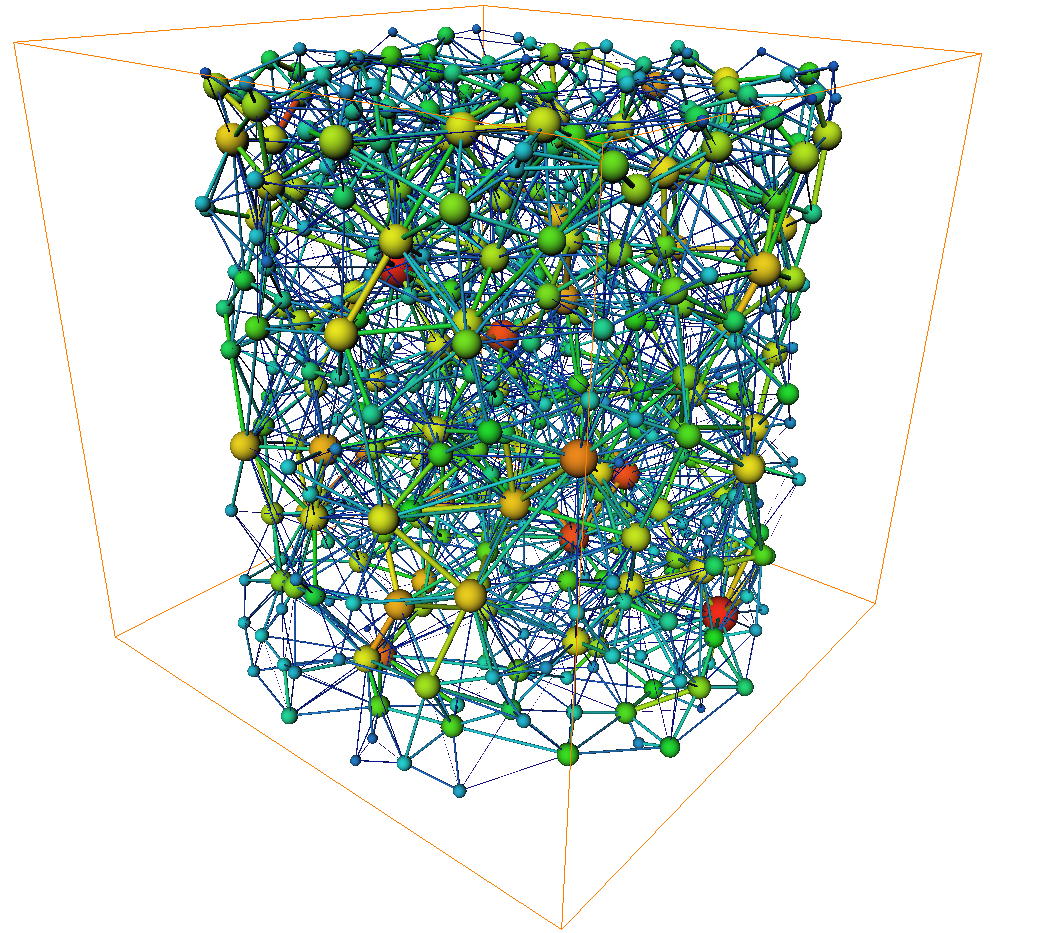

Supplement: S1 Data — (ZIP) [file pone.0296437.s001.zip › SI-Data/Data aggregation/Biomineralization sample/Biomineralization-3D reconstruction of the fig/20-3.png]

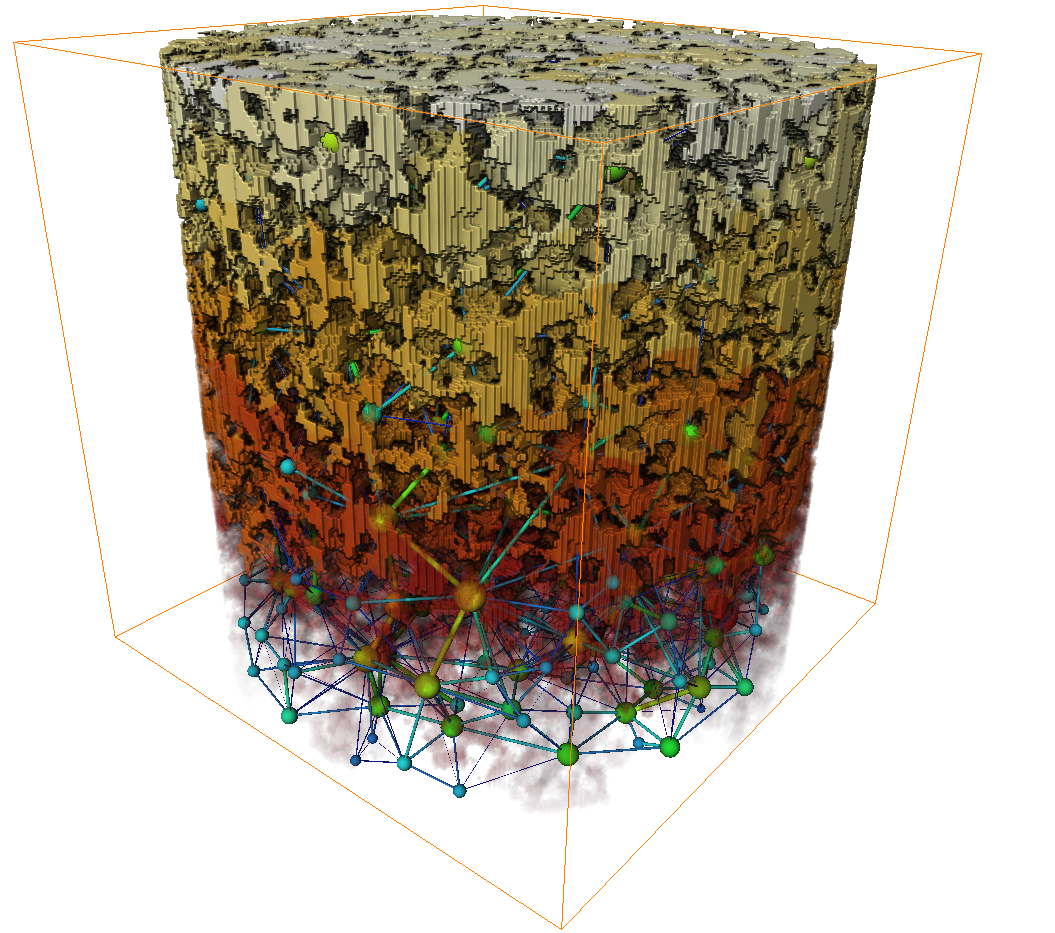

Supplement: S1 Data — (ZIP) [file pone.0296437.s001.zip › SI-Data/Data aggregation/Biomineralization sample/Biomineralization-3D reconstruction of the fig/21-1.png]

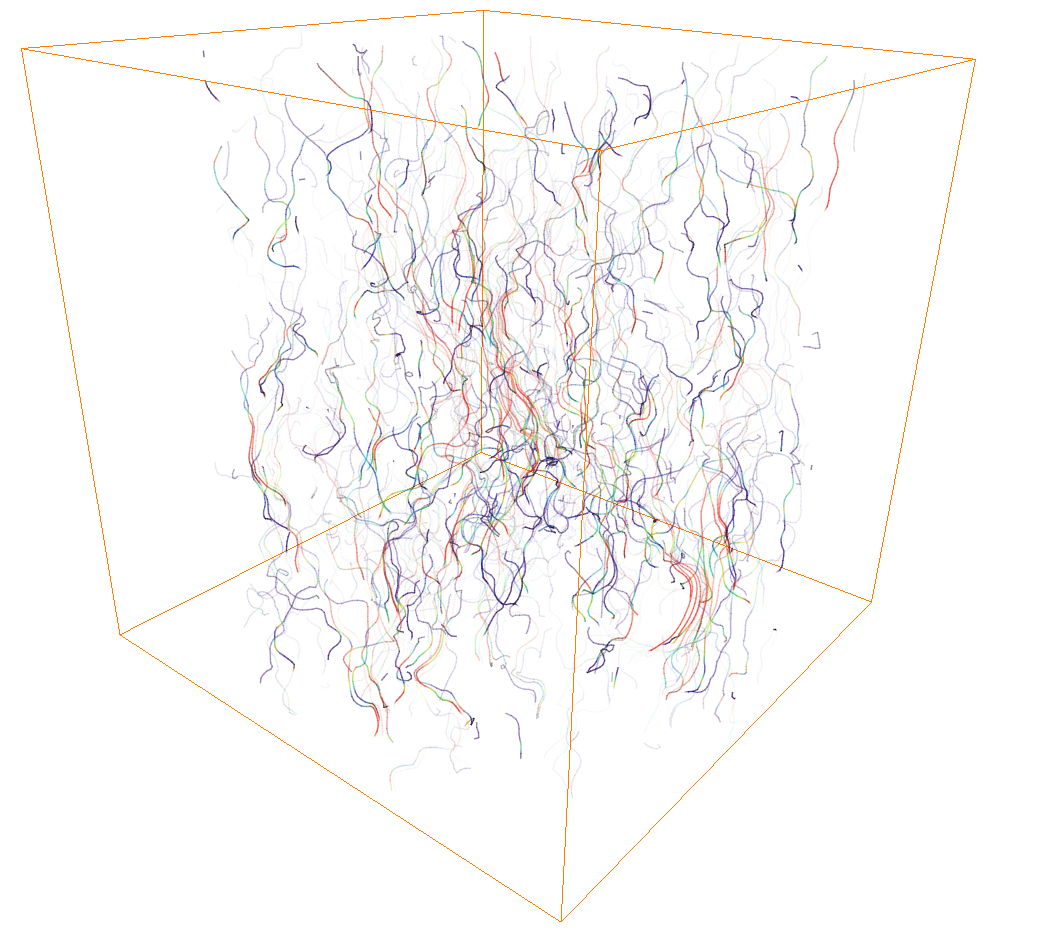

Supplement: S1 Data — (ZIP) [file pone.0296437.s001.zip › SI-Data/Data aggregation/Biomineralization sample/Biomineralization-3D reconstruction of the fig/22-1.png]

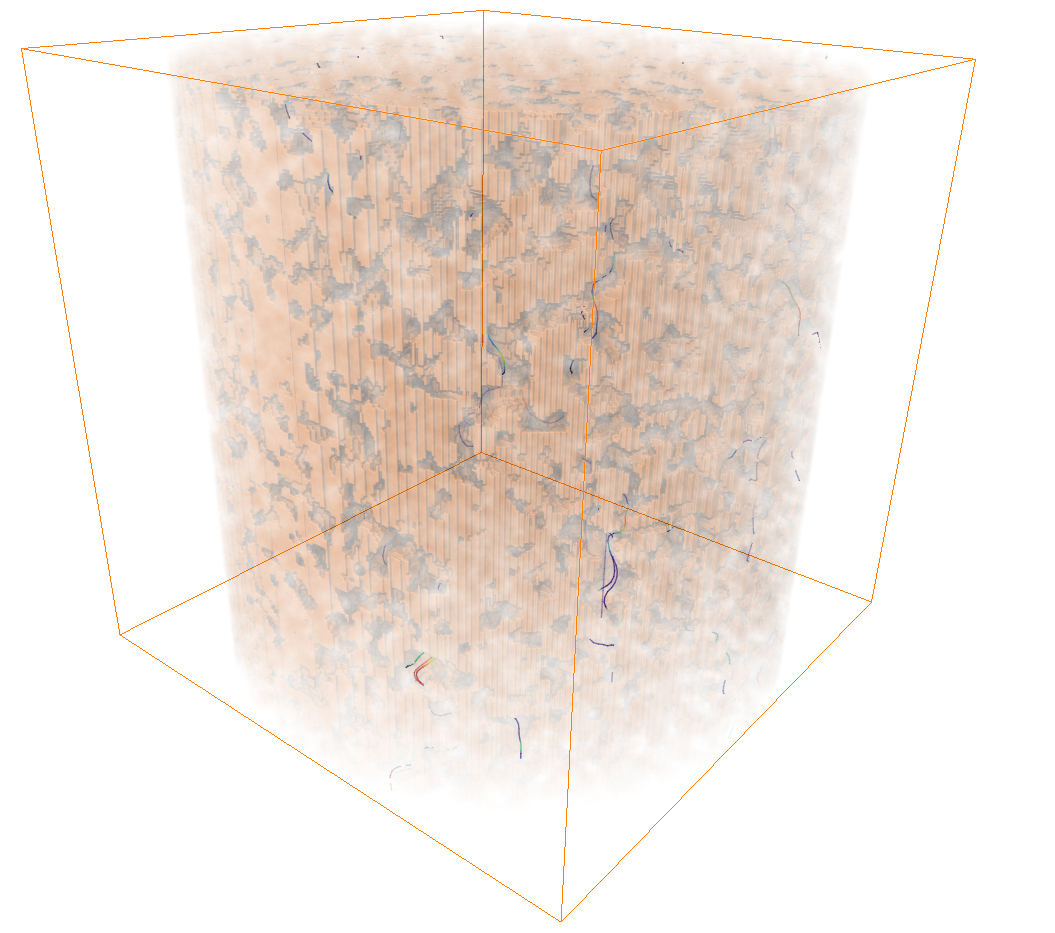

Supplement: S1 Data — (ZIP) [file pone.0296437.s001.zip › SI-Data/Data aggregation/Biomineralization sample/Biomineralization-3D reconstruction of the fig/23-1.png]

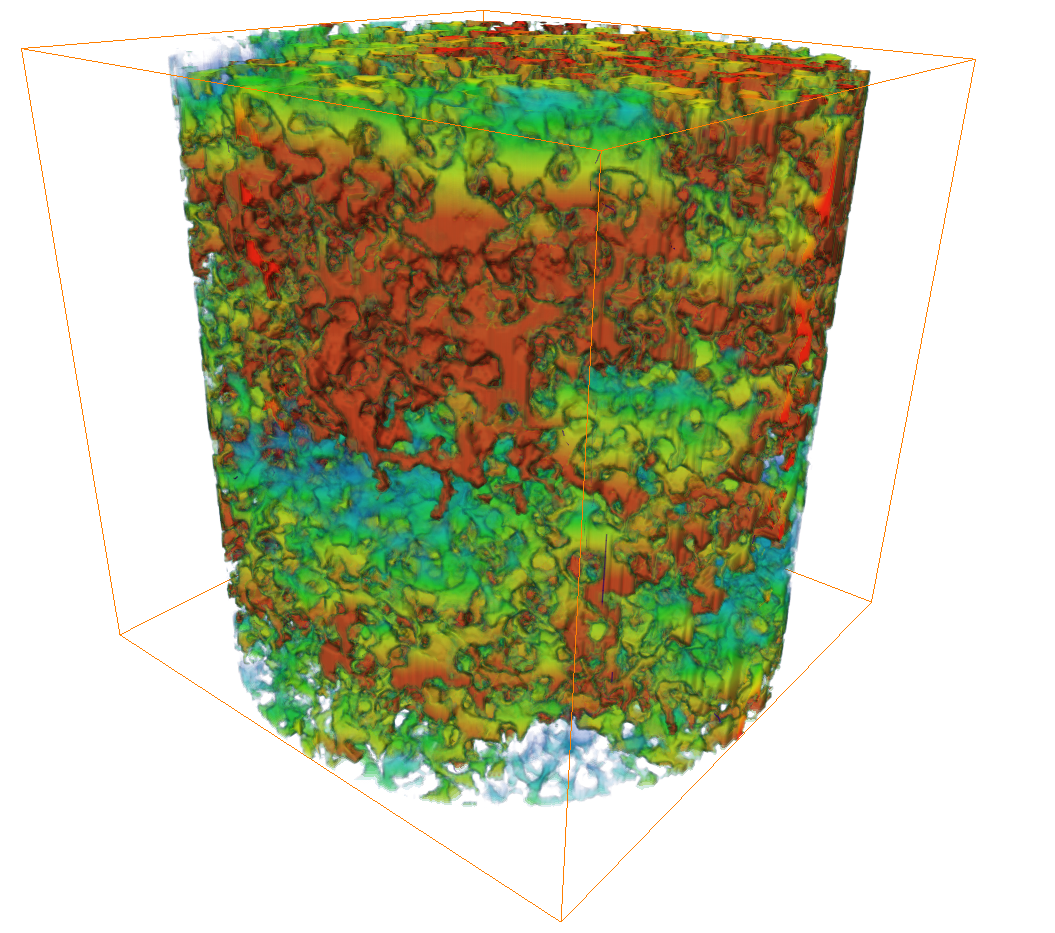

Supplement: S1 Data — (ZIP) [file pone.0296437.s001.zip › SI-Data/Data aggregation/Biomineralization sample/Biomineralization-3D reconstruction of the fig/24-1.png]

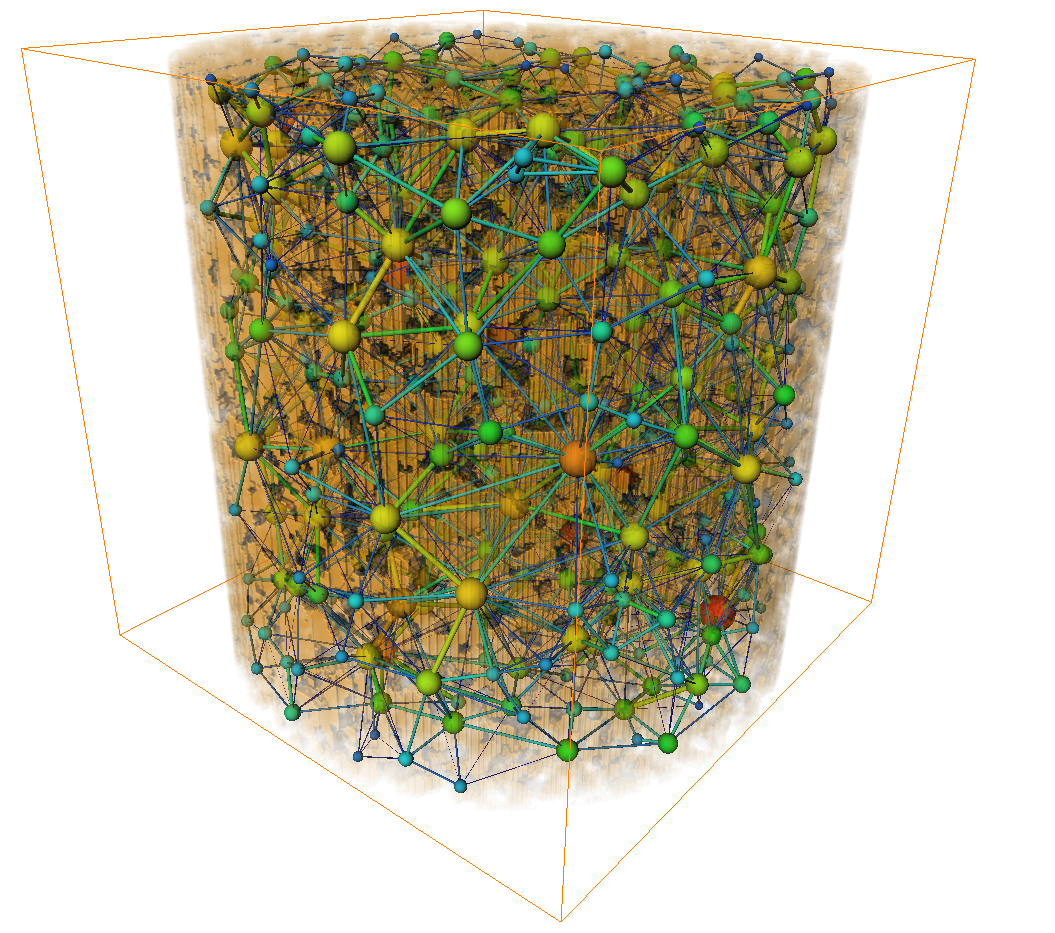

Supplement: S1 Data — (ZIP) [file pone.0296437.s001.zip › SI-Data/Data aggregation/Biomineralization sample/Biomineralization-3D reconstruction of the fig/25-1.png]

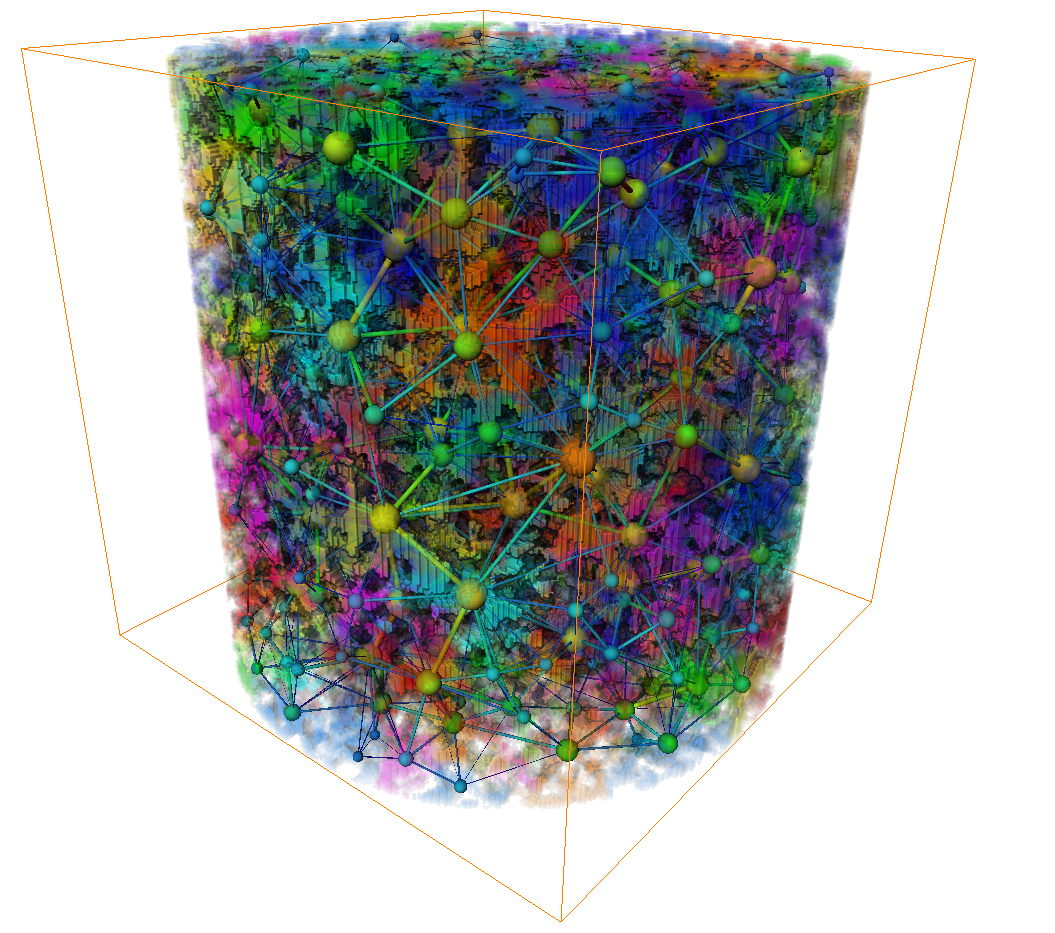

Supplement: S1 Data — (ZIP) [file pone.0296437.s001.zip › SI-Data/Data aggregation/Biomineralization sample/Biomineralization-3D reconstruction of the fig/26-1.png]

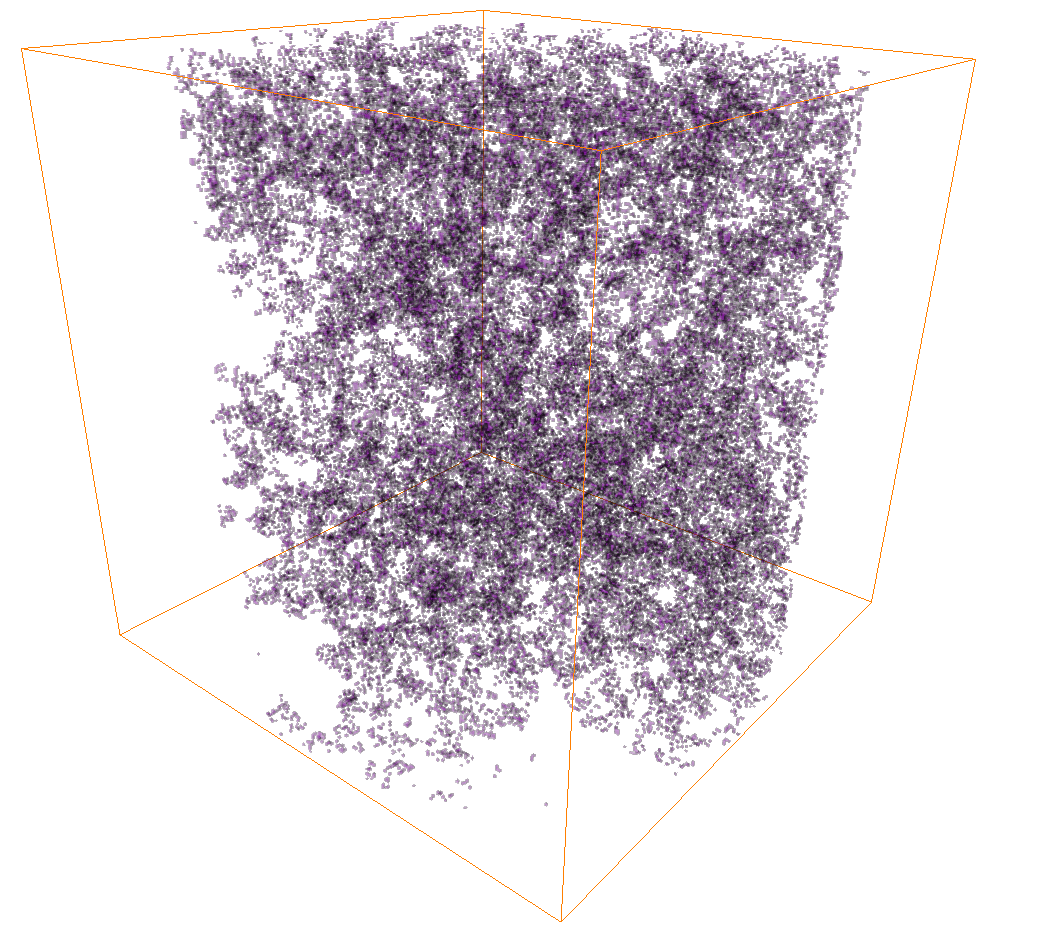

Supplement: S1 Data — (ZIP) [file pone.0296437.s001.zip › SI-Data/Data aggregation/Biomineralization sample/Biomineralization-3D reconstruction of the fig/27-1.png]

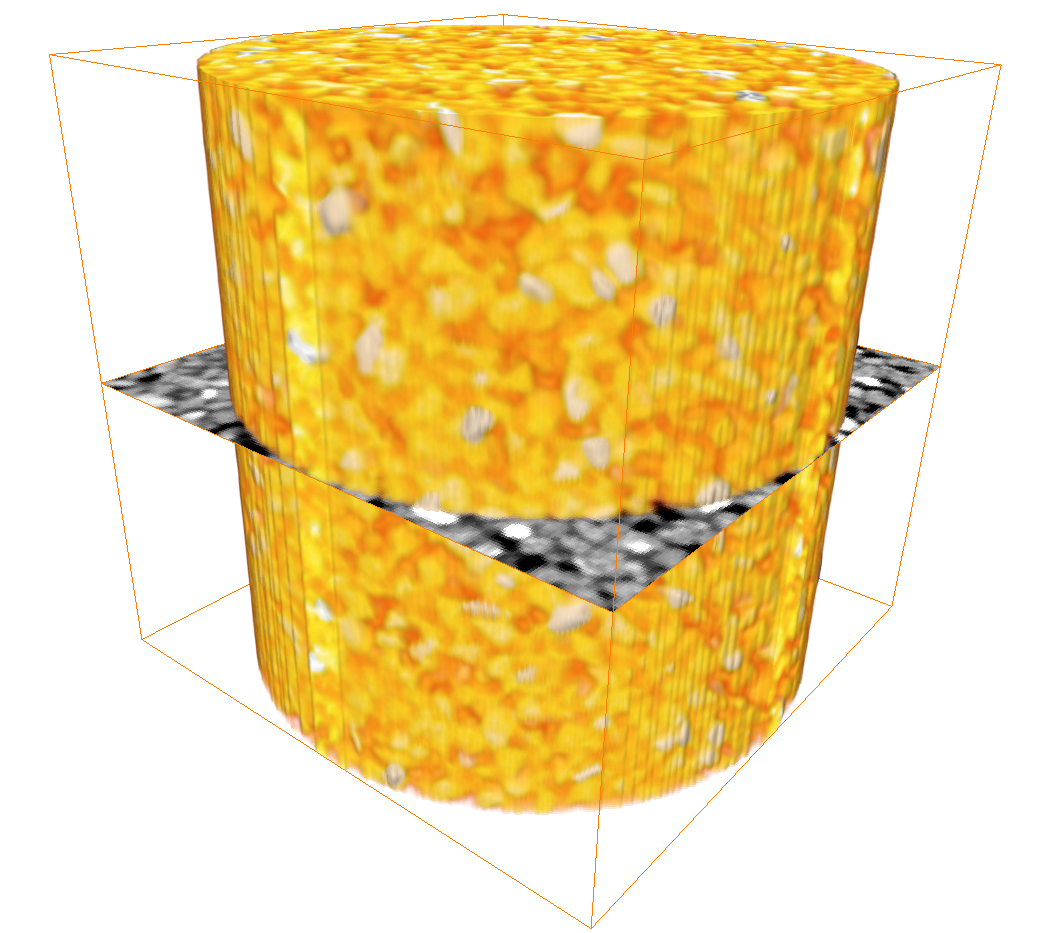

Supplement: S1 Data — (ZIP) [file pone.0296437.s001.zip › SI-Data/Data aggregation/Biomineralization sample/Biomineralization-3D reconstruction of the fig/3-1.png]

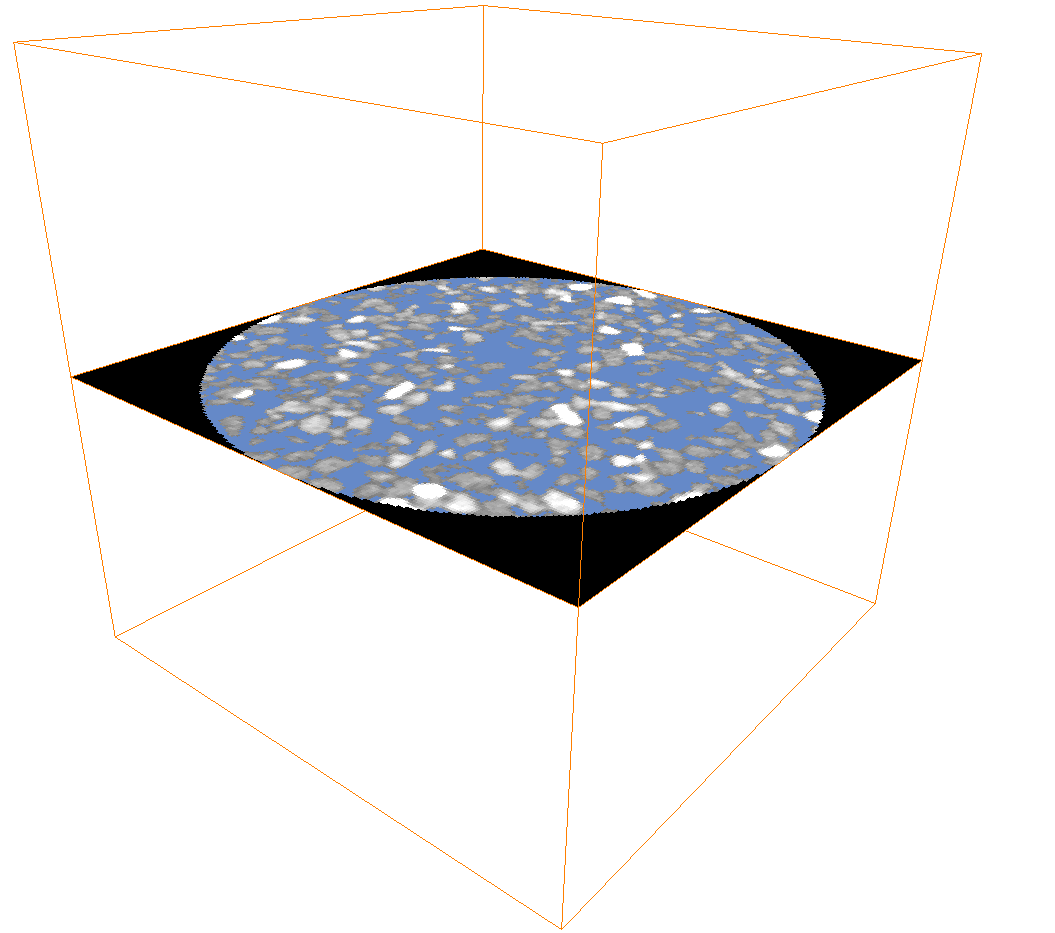

Supplement: S1 Data — (ZIP) [file pone.0296437.s001.zip › SI-Data/Data aggregation/Biomineralization sample/Biomineralization-3D reconstruction of the fig/4-1.png]

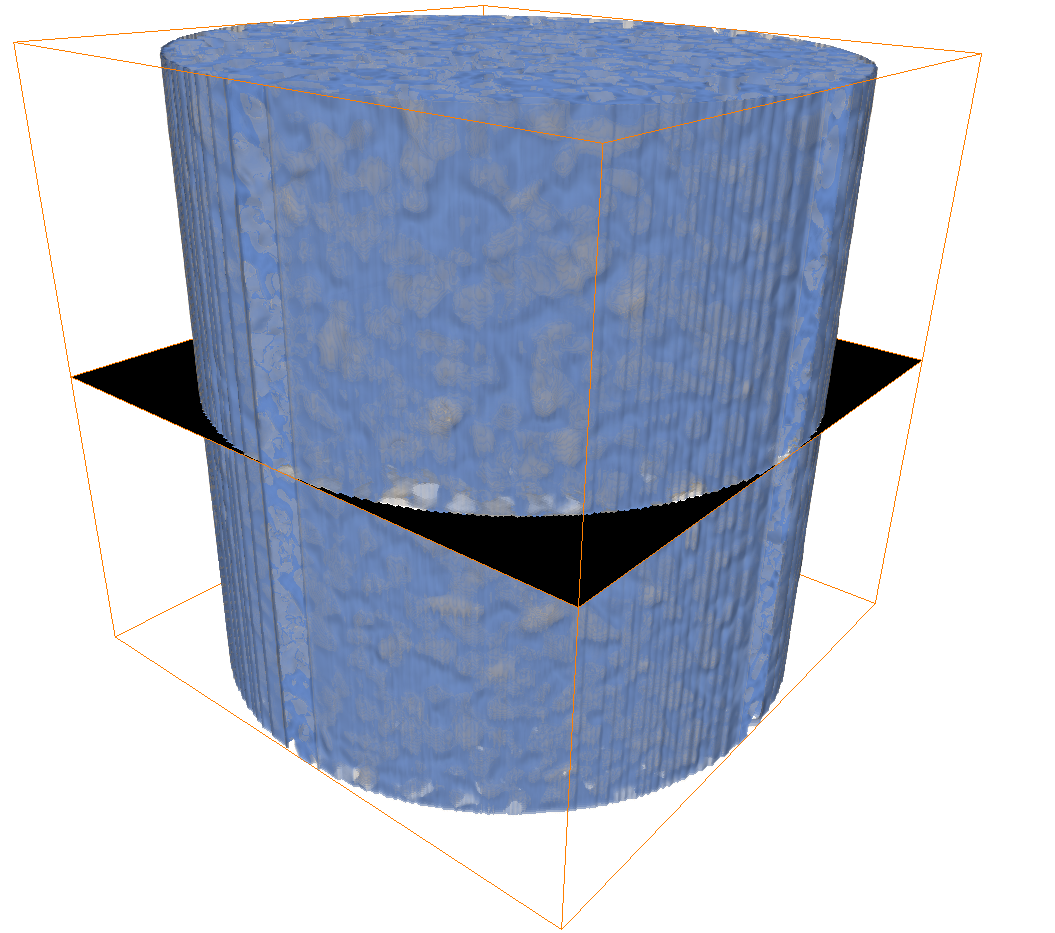

Supplement: S1 Data — (ZIP) [file pone.0296437.s001.zip › SI-Data/Data aggregation/Biomineralization sample/Biomineralization-3D reconstruction of the fig/5-1.png]

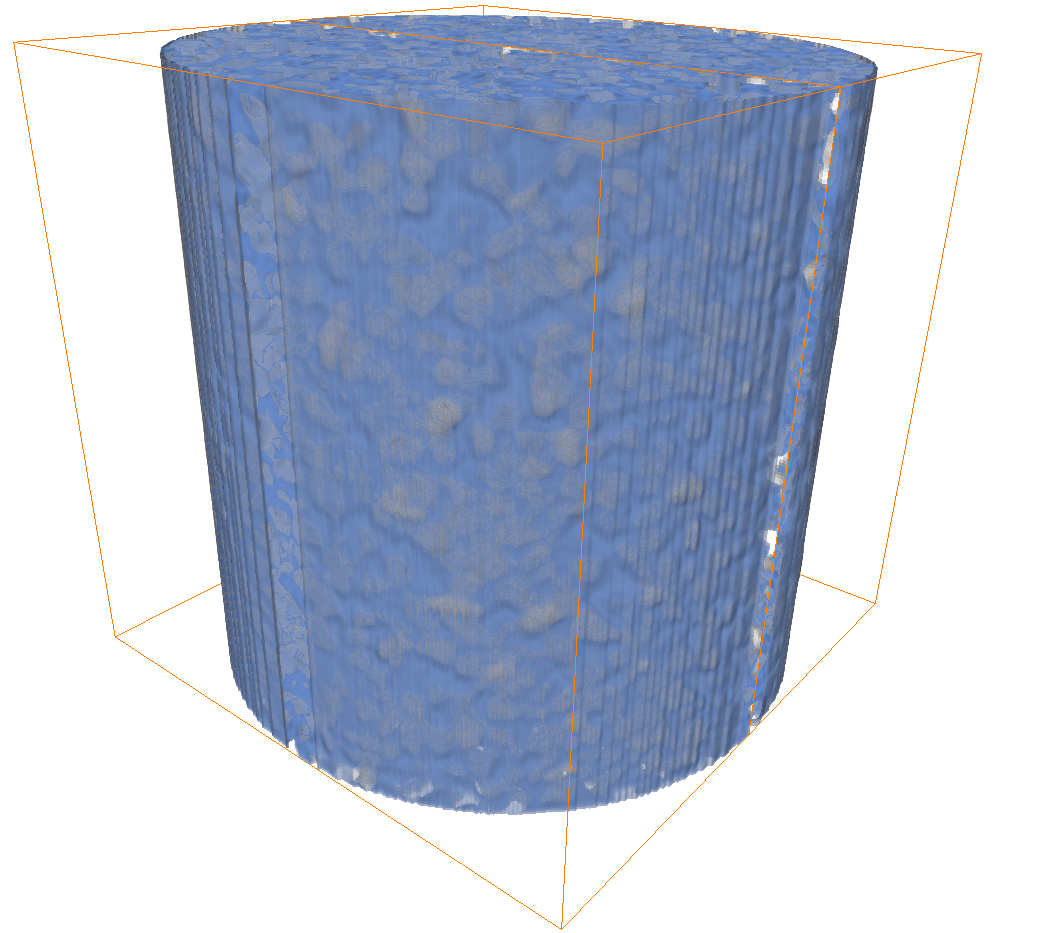

Supplement: S1 Data — (ZIP) [file pone.0296437.s001.zip › SI-Data/Data aggregation/Biomineralization sample/Biomineralization-3D reconstruction of the fig/5-3.png]

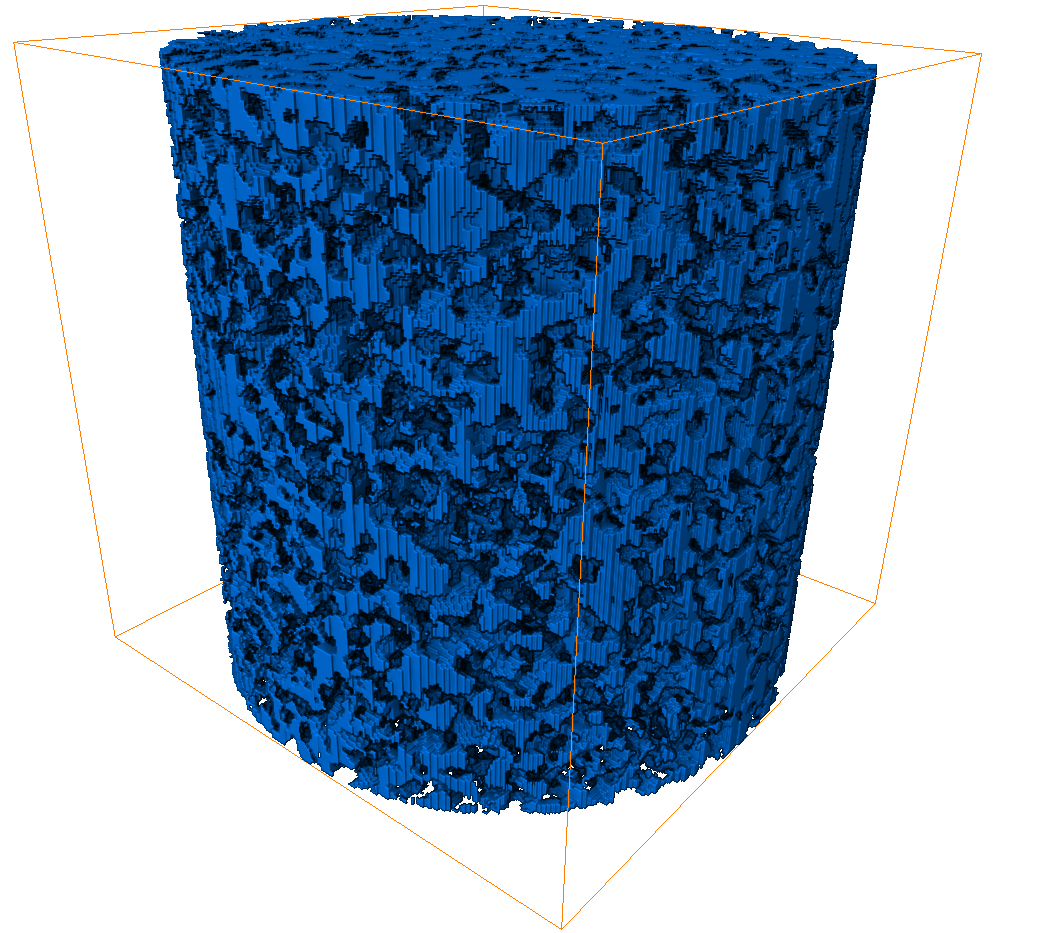

Supplement: S1 Data — (ZIP) [file pone.0296437.s001.zip › SI-Data/Data aggregation/Biomineralization sample/Biomineralization-3D reconstruction of the fig/6-1.png]

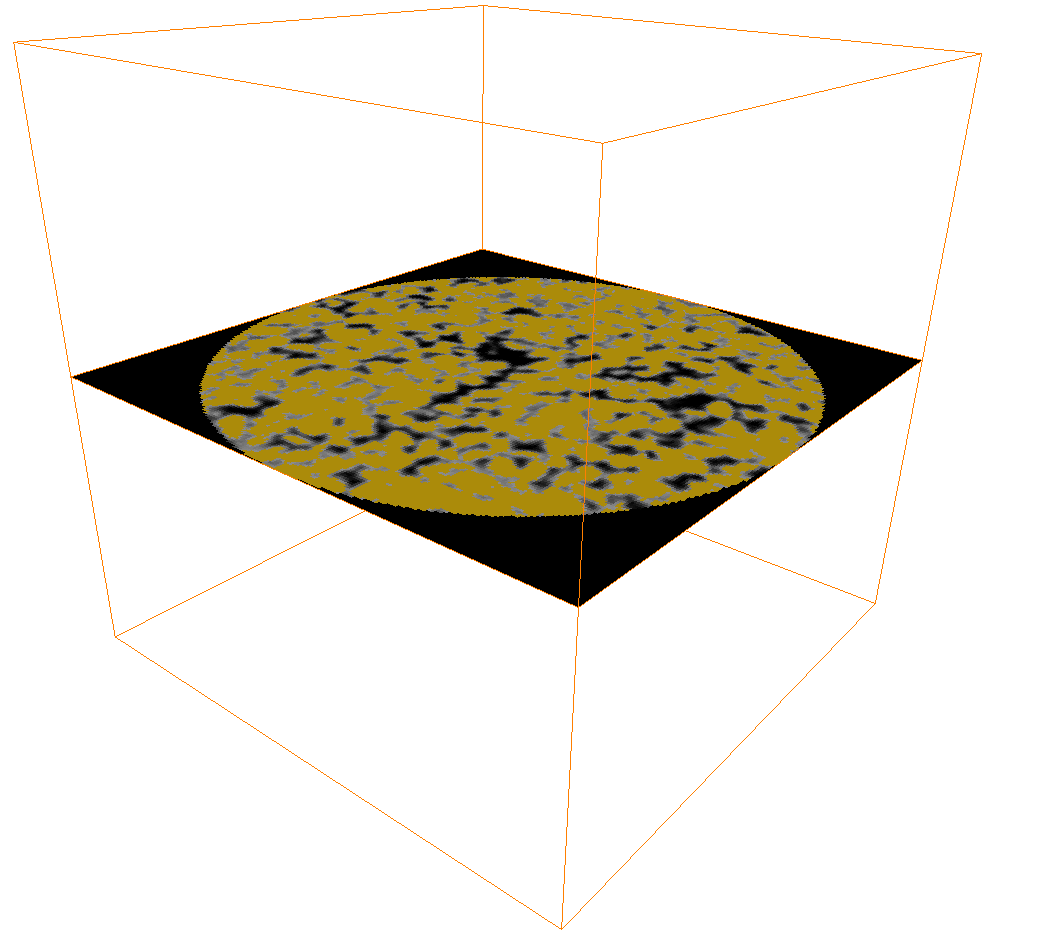

Supplement: S1 Data — (ZIP) [file pone.0296437.s001.zip › SI-Data/Data aggregation/Biomineralization sample/Biomineralization-3D reconstruction of the fig/7-1.png]

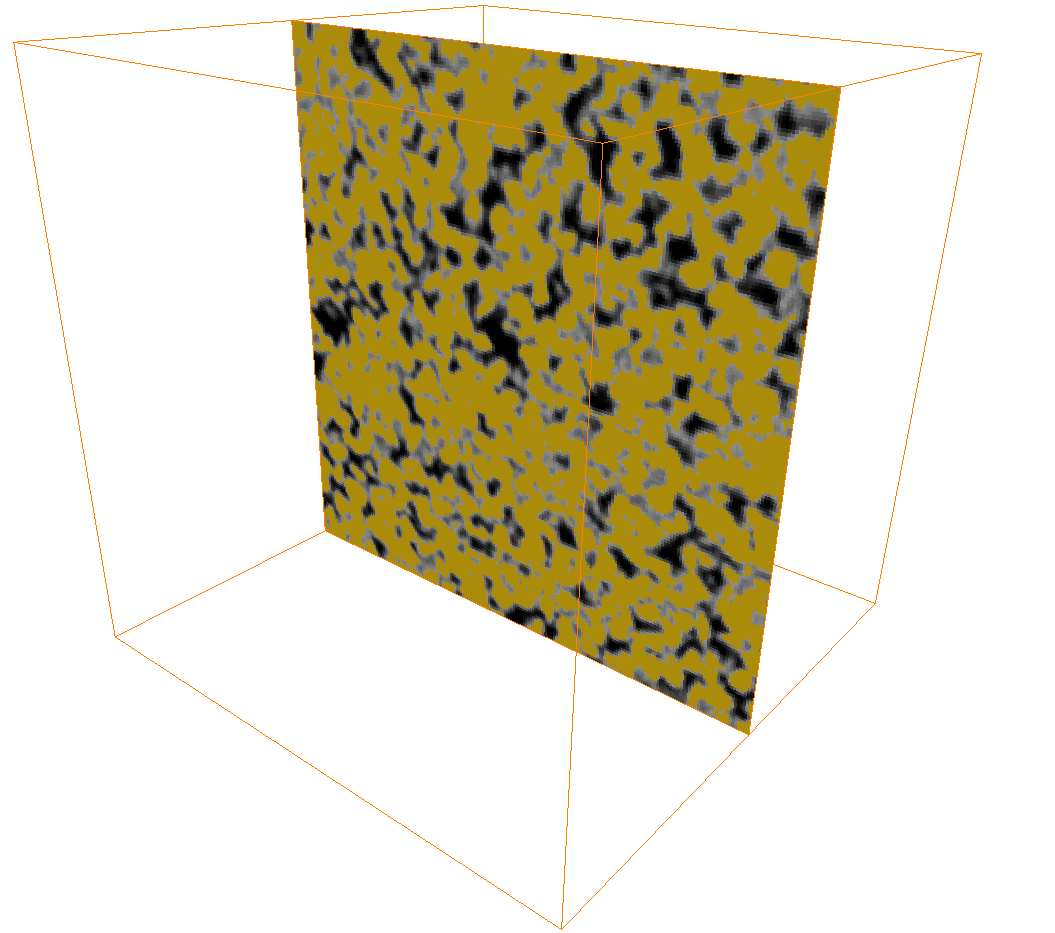

Supplement: S1 Data — (ZIP) [file pone.0296437.s001.zip › SI-Data/Data aggregation/Biomineralization sample/Biomineralization-3D reconstruction of the fig/7-3.png]

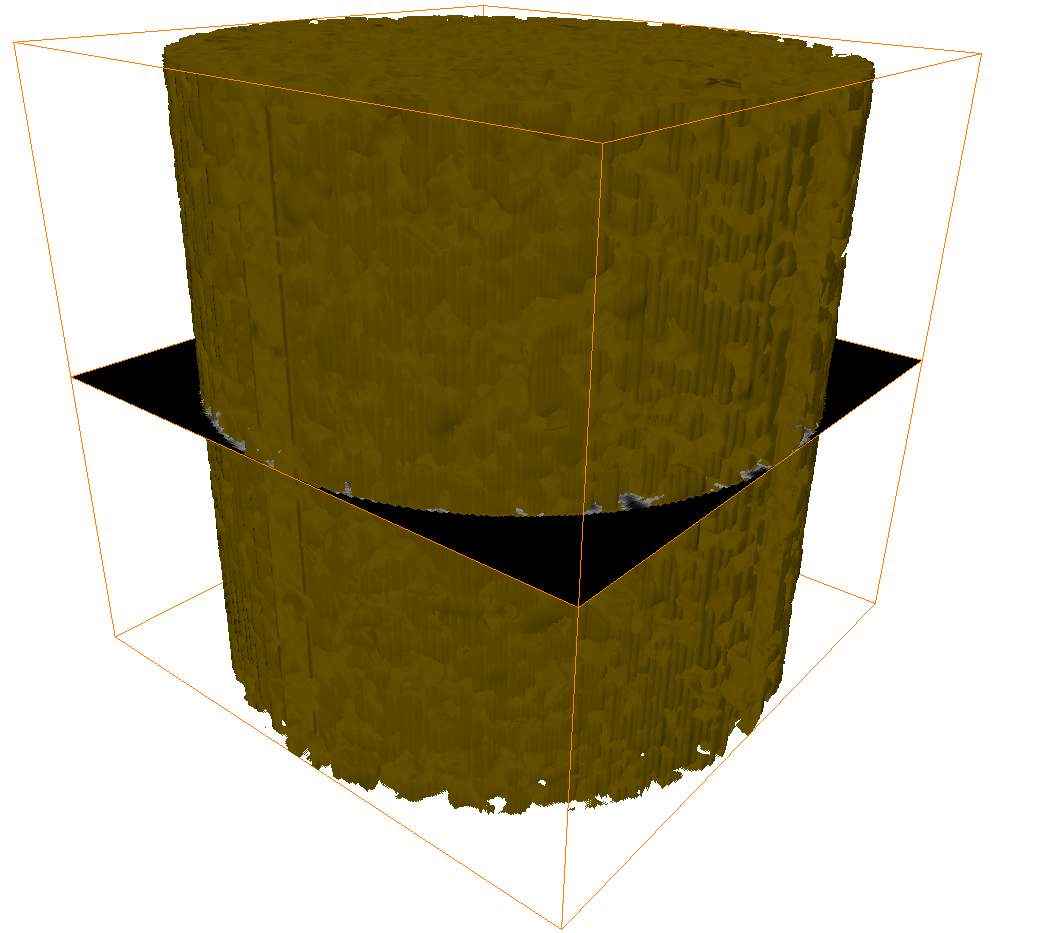

Supplement: S1 Data — (ZIP) [file pone.0296437.s001.zip › SI-Data/Data aggregation/Biomineralization sample/Biomineralization-3D reconstruction of the fig/8-1.png]

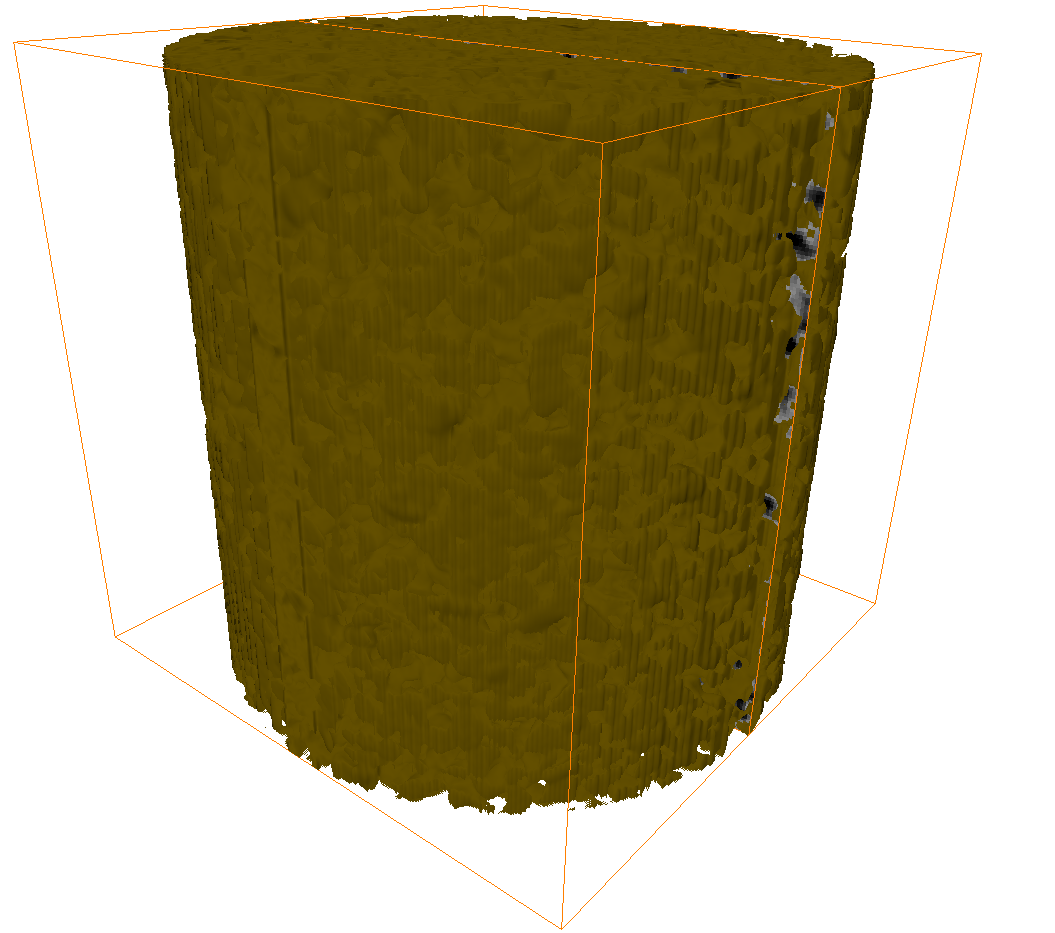

Supplement: S1 Data — (ZIP) [file pone.0296437.s001.zip › SI-Data/Data aggregation/Biomineralization sample/Biomineralization-3D reconstruction of the fig/8-3.png]

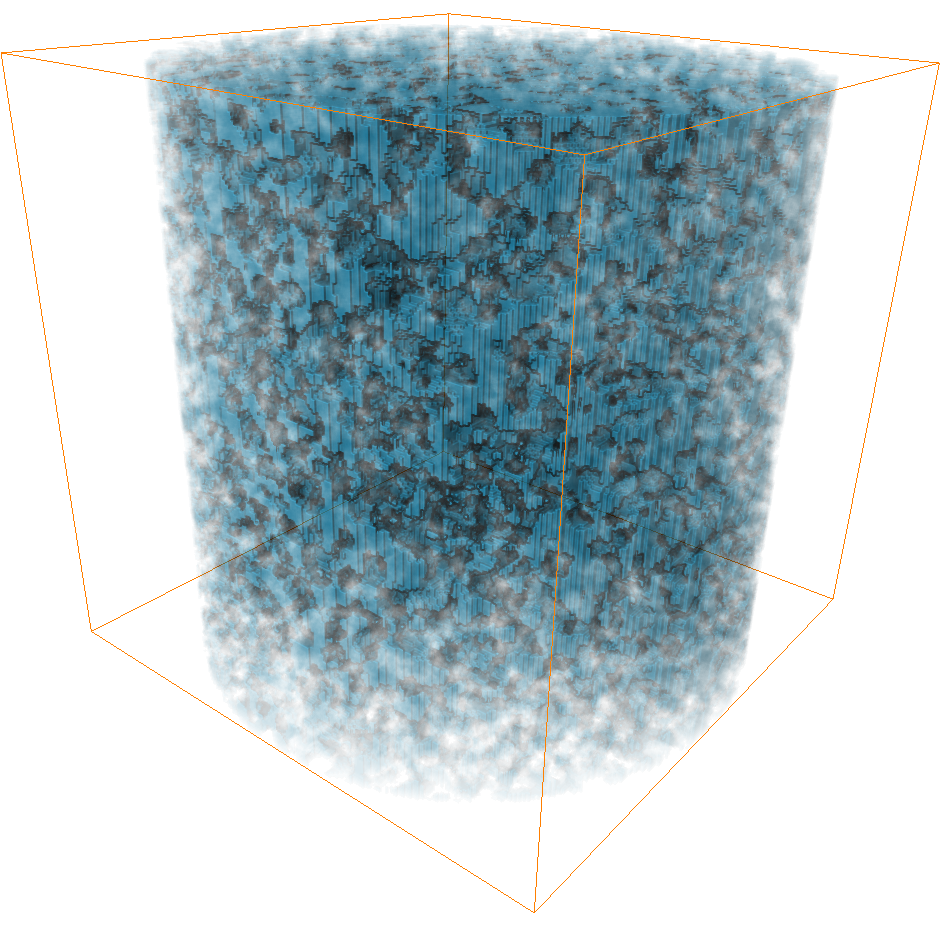

Supplement: S1 Data — (ZIP) [file pone.0296437.s001.zip › SI-Data/Data aggregation/Biomineralization sample/Biomineralization-3D reconstruction of the fig/color/1-1.png]

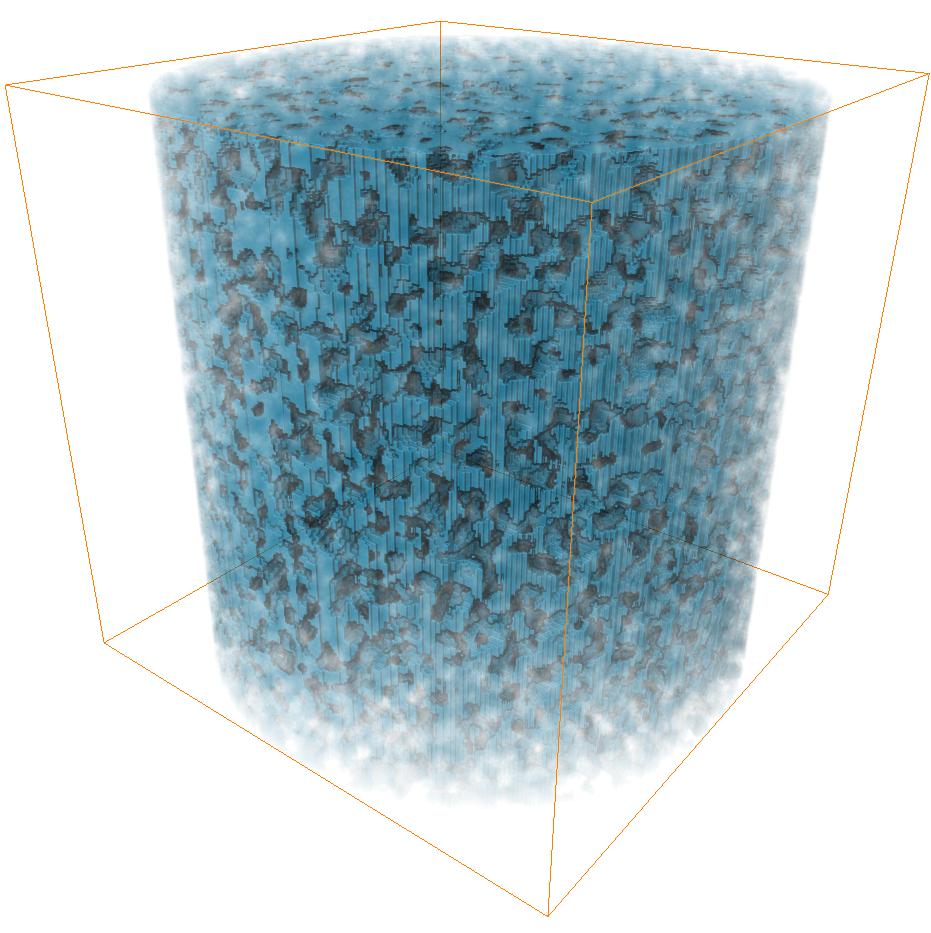

Supplement: S1 Data — (ZIP) [file pone.0296437.s001.zip › SI-Data/Data aggregation/Biomineralization sample/Biomineralization-3D reconstruction of the fig/color/1-2.png]

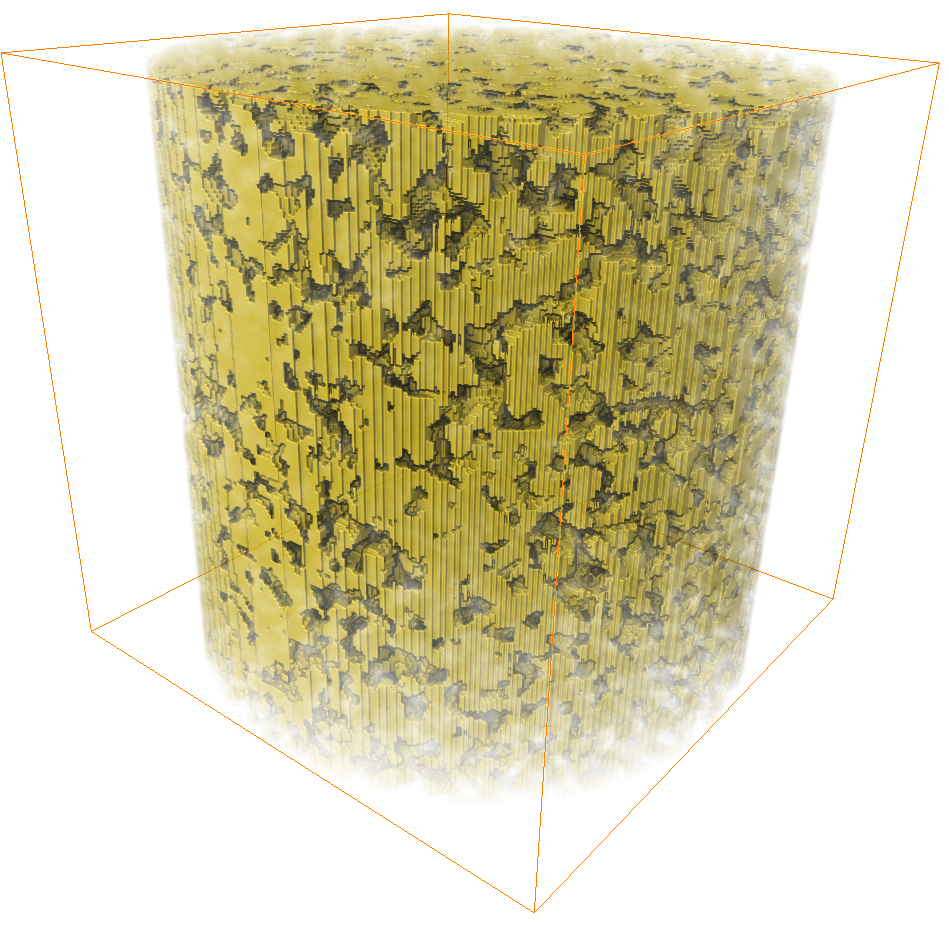

Supplement: S1 Data — (ZIP) [file pone.0296437.s001.zip › SI-Data/Data aggregation/Biomineralization sample/Biomineralization-3D reconstruction of the fig/color/2-1.png]

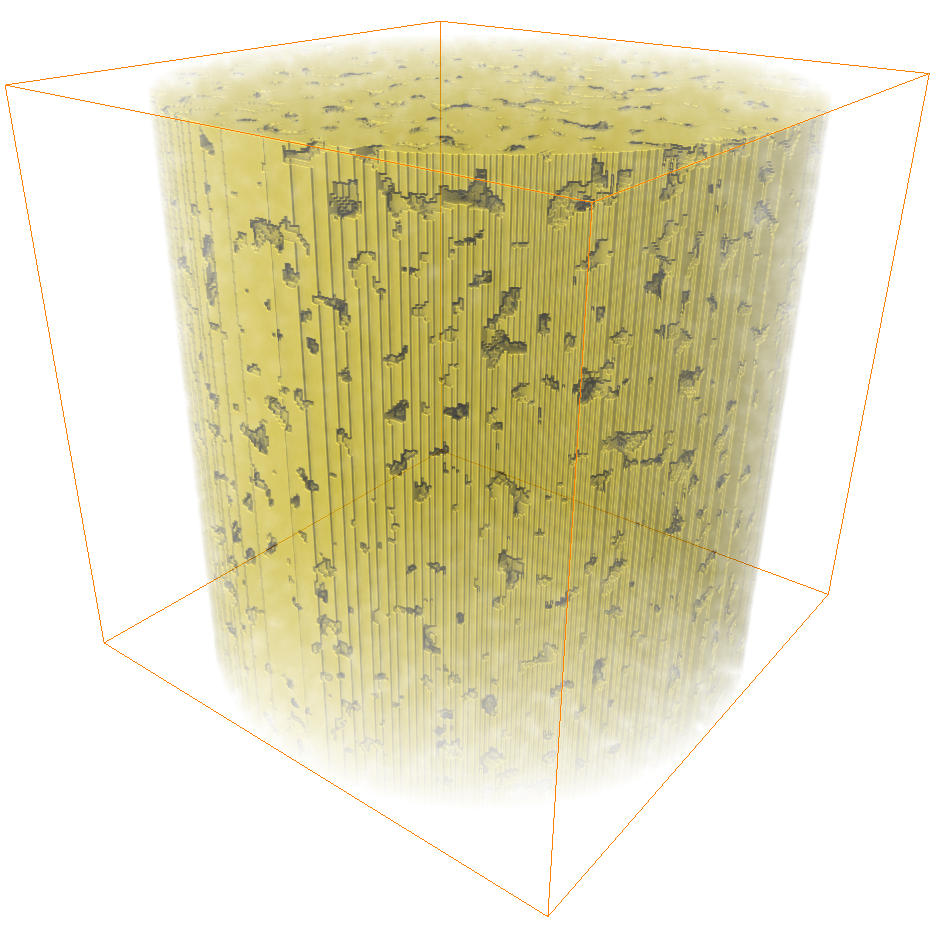

Supplement: S1 Data — (ZIP) [file pone.0296437.s001.zip › SI-Data/Data aggregation/Biomineralization sample/Biomineralization-3D reconstruction of the fig/color/2-2 .png]

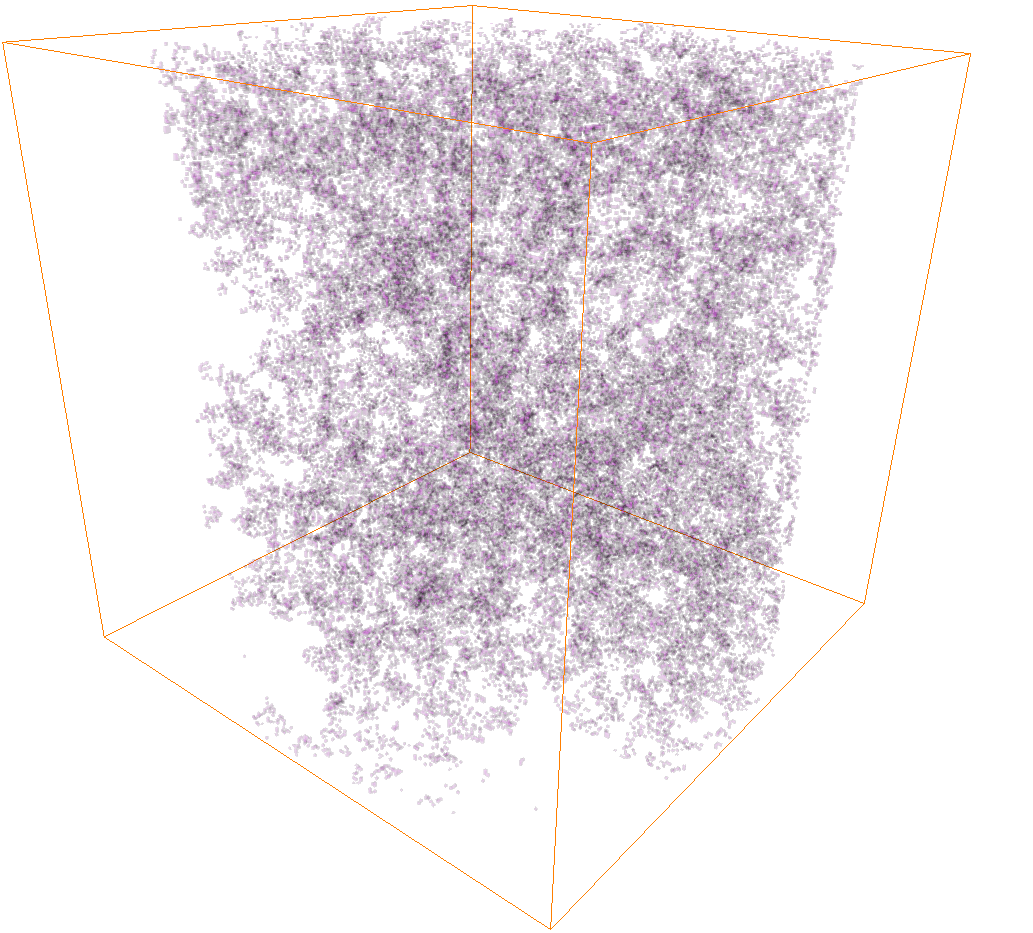

Supplement: S1 Data — (ZIP) [file pone.0296437.s001.zip › SI-Data/Data aggregation/Biomineralization sample/Biomineralization-3D reconstruction of the fig/color/3-1.png]

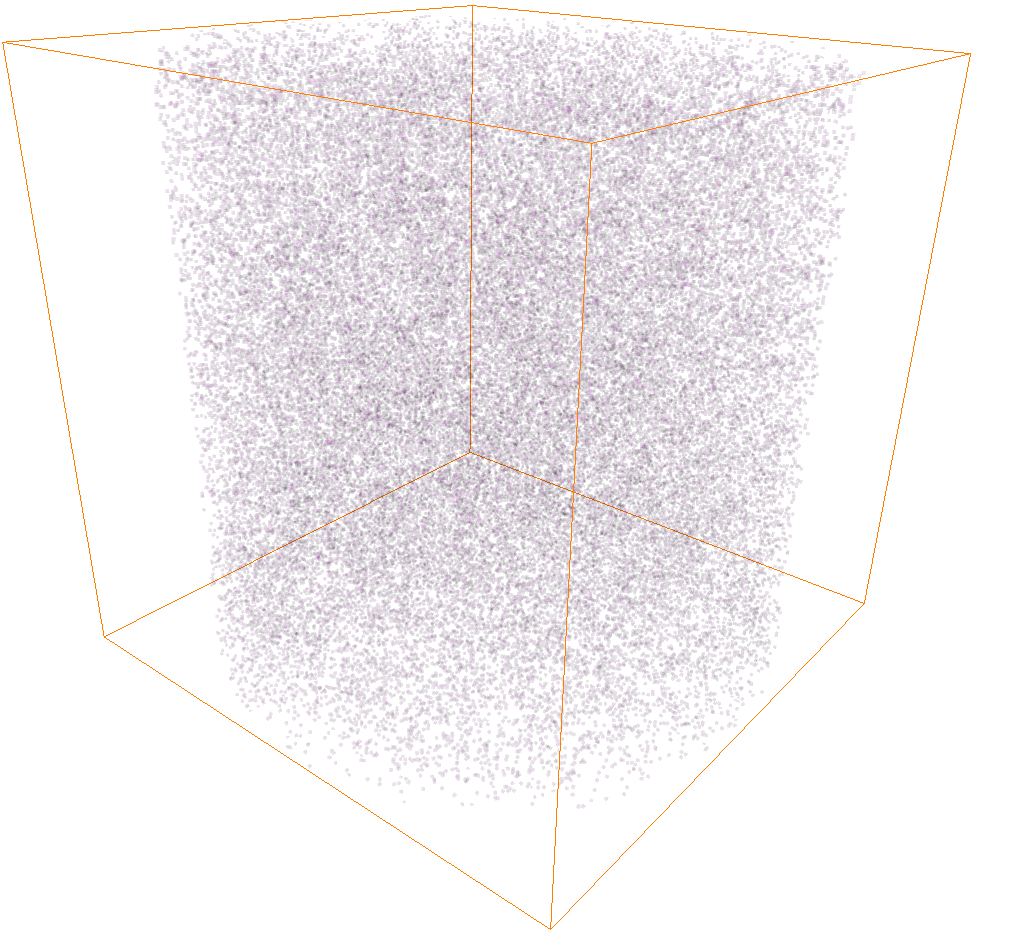

Supplement: S1 Data — (ZIP) [file pone.0296437.s001.zip › SI-Data/Data aggregation/Biomineralization sample/Biomineralization-3D reconstruction of the fig/color/4-1.png]

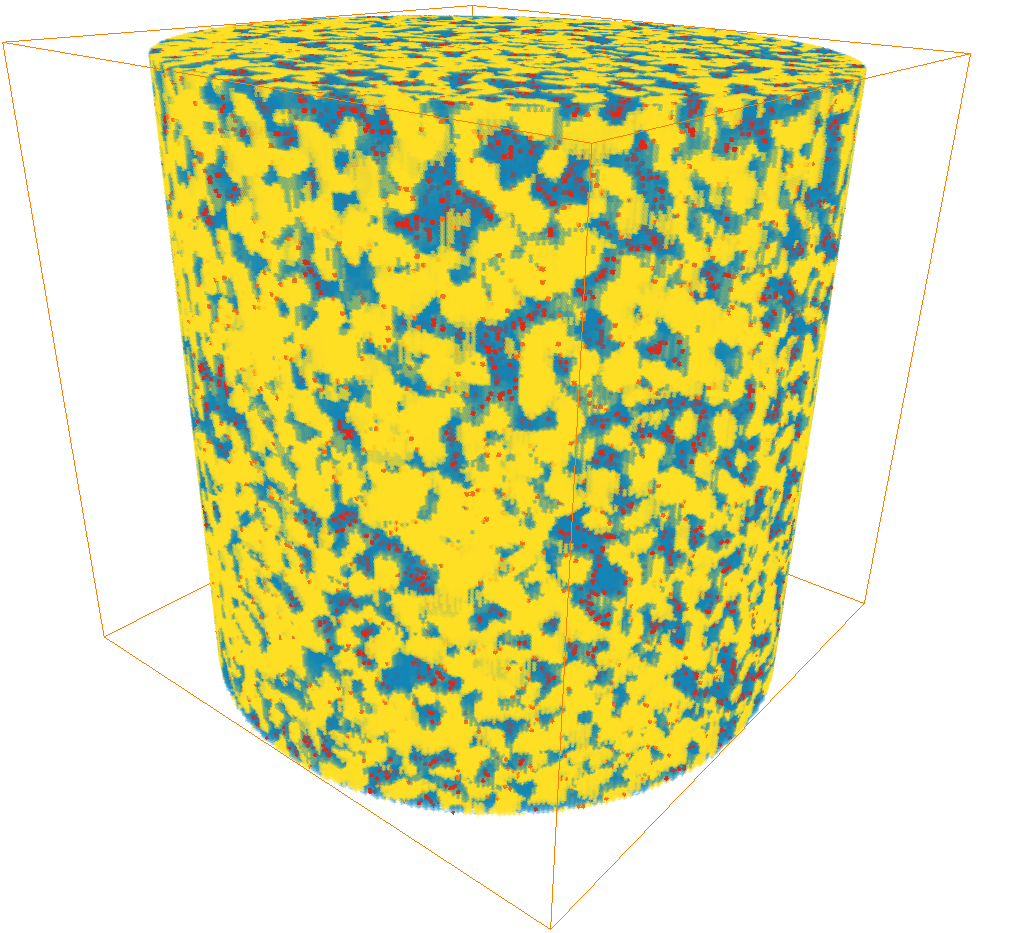

Supplement: S1 Data — (ZIP) [file pone.0296437.s001.zip › SI-Data/Data aggregation/Biomineralization sample/Biomineralization-3D reconstruction of the fig/color/5-1.png]

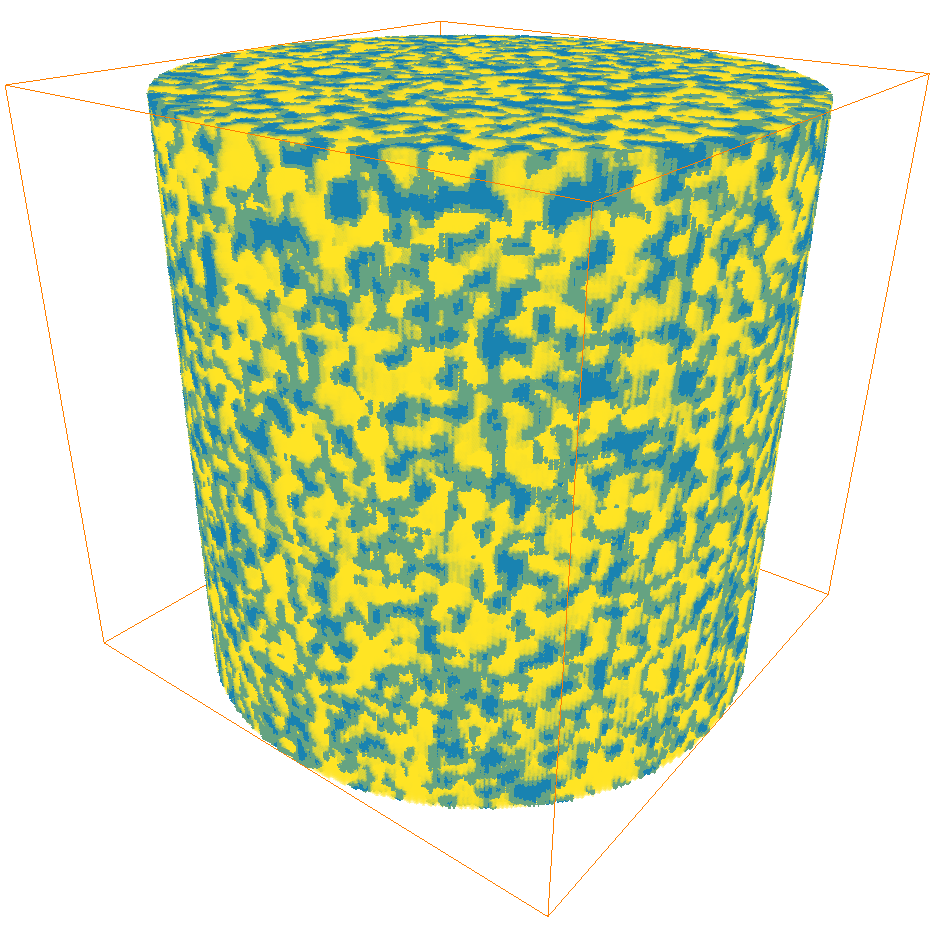

Supplement: S1 Data — (ZIP) [file pone.0296437.s001.zip › SI-Data/Data aggregation/Biomineralization sample/Biomineralization-3D reconstruction of the fig/color/5-2.png]

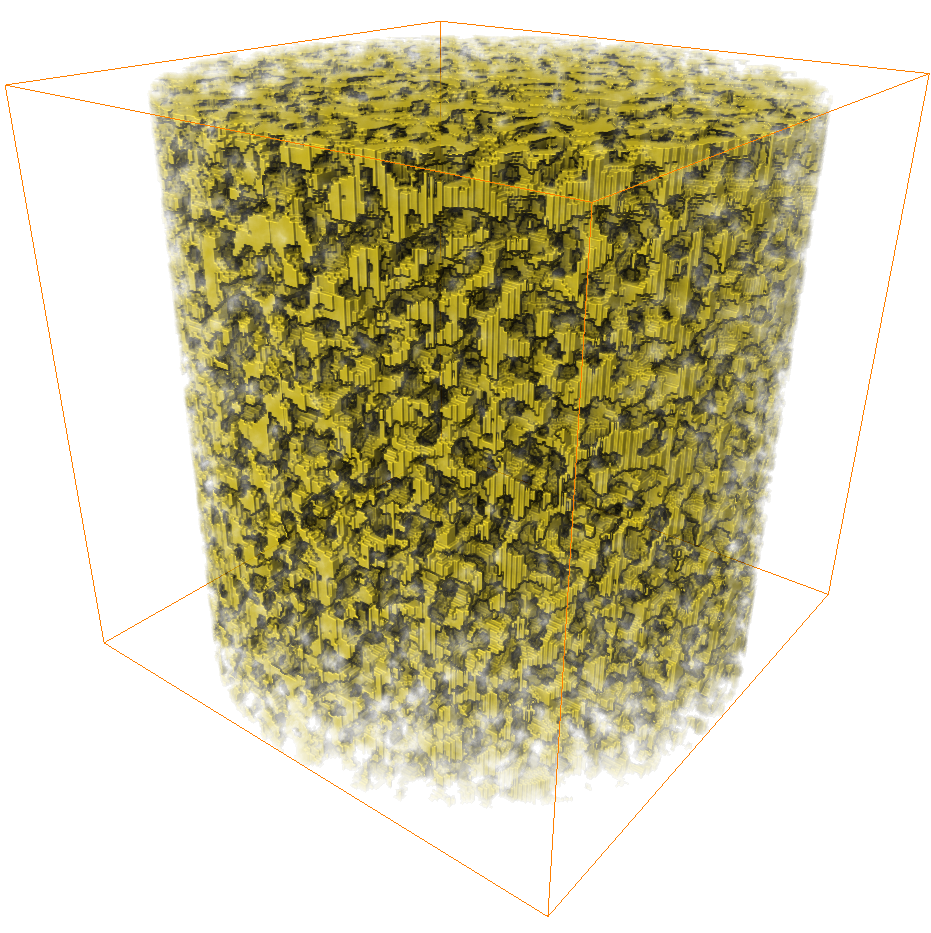

Supplement: S1 Data — (ZIP) [file pone.0296437.s001.zip › SI-Data/Data aggregation/Biomineralization sample/Biomineralization-3D reconstruction of the fig/color/6-1.png]

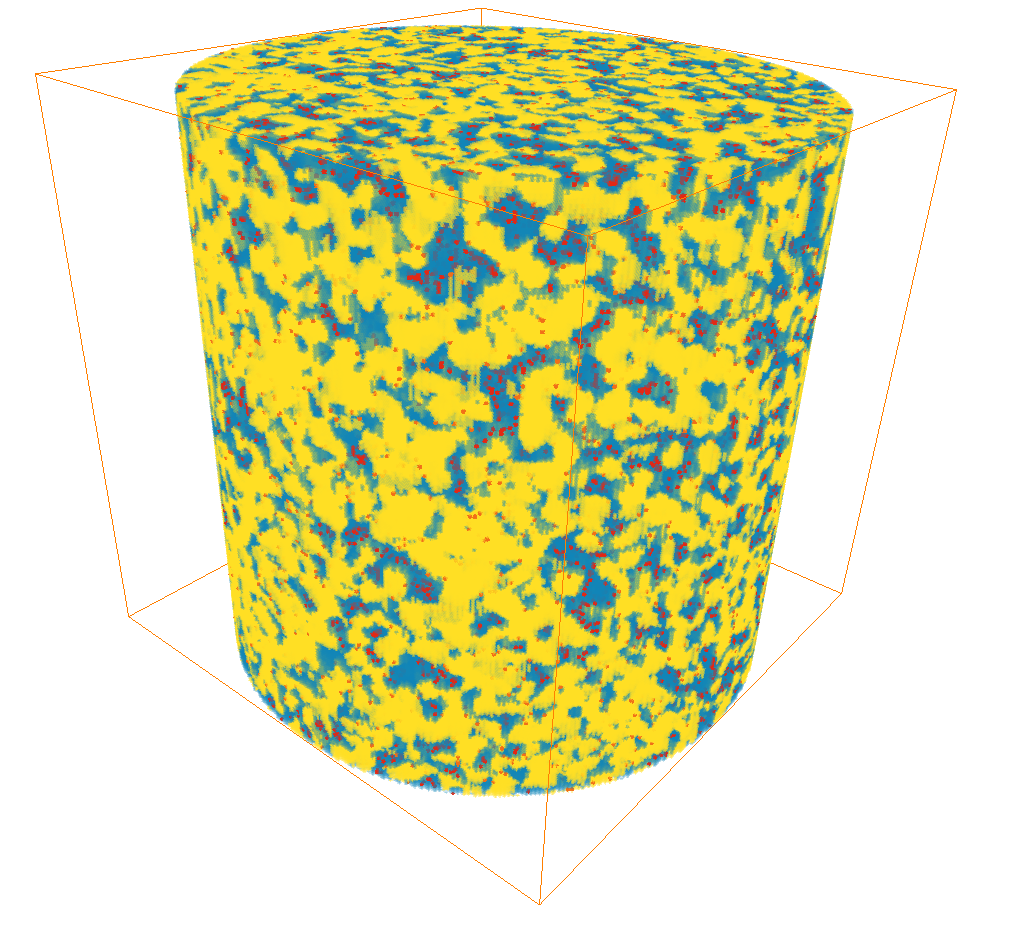

Supplement: S1 Data — (ZIP) [file pone.0296437.s001.zip › SI-Data/Data aggregation/Biomineralization sample/Biomineralization-3D reconstruction of the fig/color/7-1.png]

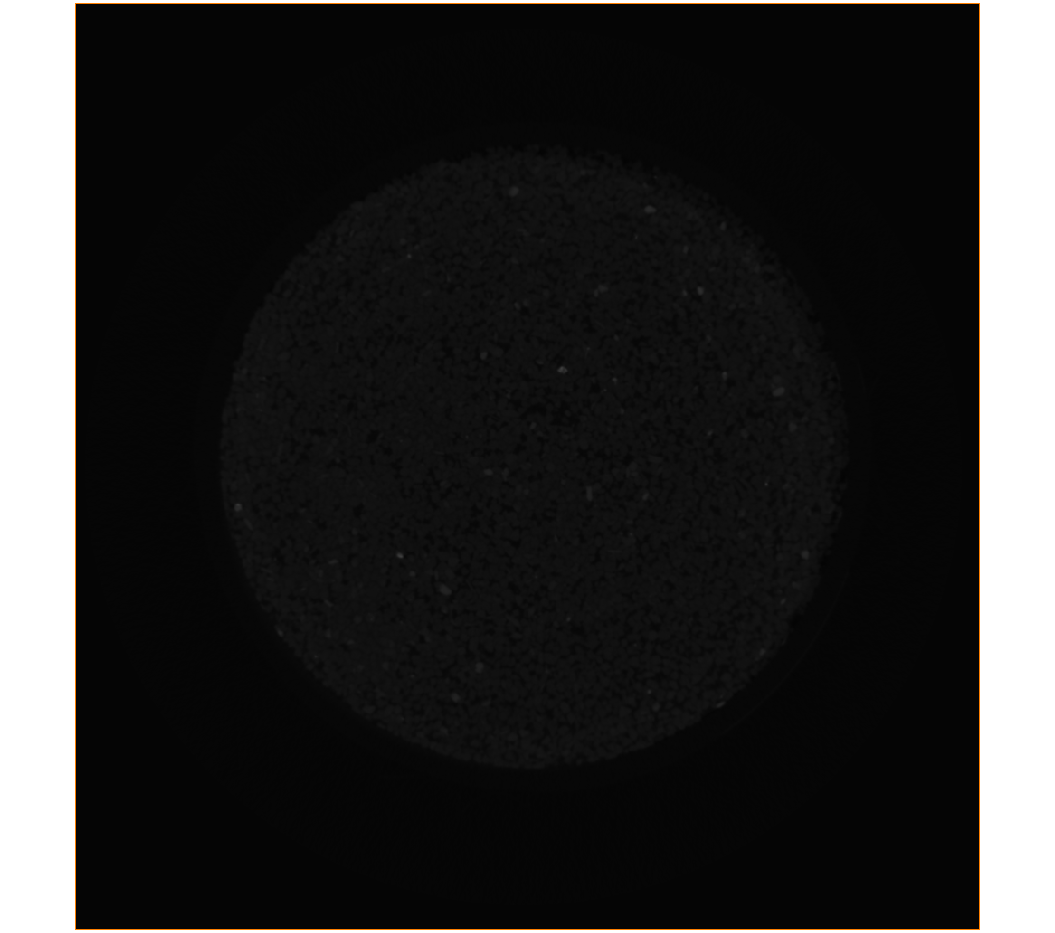

Supplement: S1 Data — (ZIP) [file pone.0296437.s001.zip › SI-Data/Data aggregation/unbiomineralization sample/3D unbiomineralization sample/1.png]

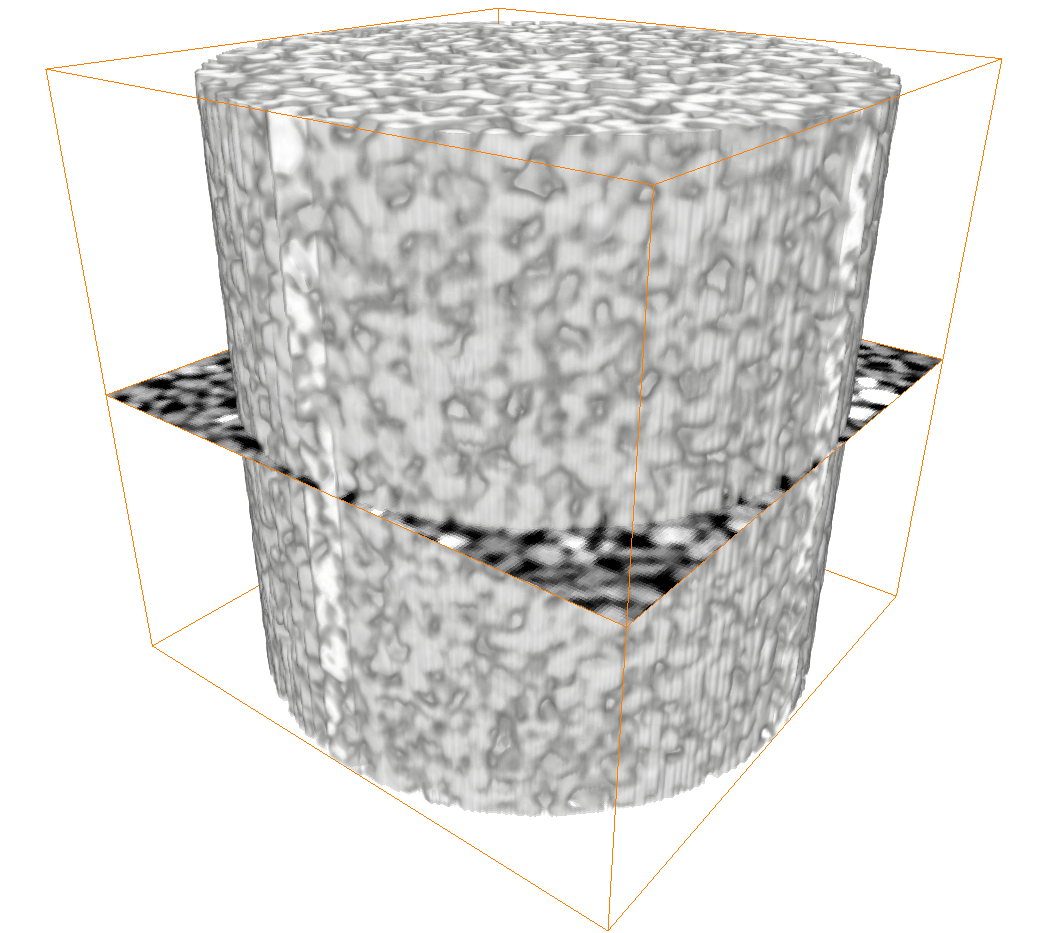

Supplement: S1 Data — (ZIP) [file pone.0296437.s001.zip › SI-Data/Data aggregation/unbiomineralization sample/3D unbiomineralization sample/10-xy.png]

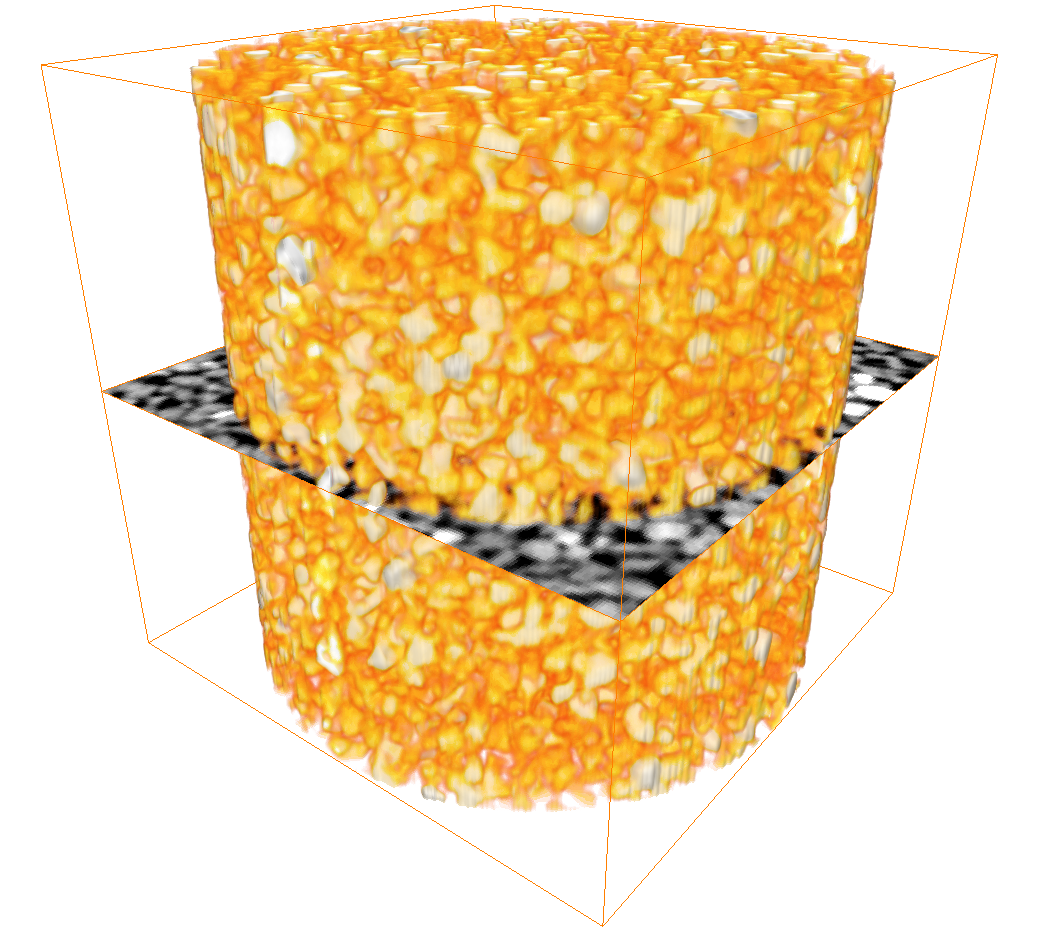

Supplement: S1 Data — (ZIP) [file pone.0296437.s001.zip › SI-Data/Data aggregation/unbiomineralization sample/3D unbiomineralization sample/11-xy.png]

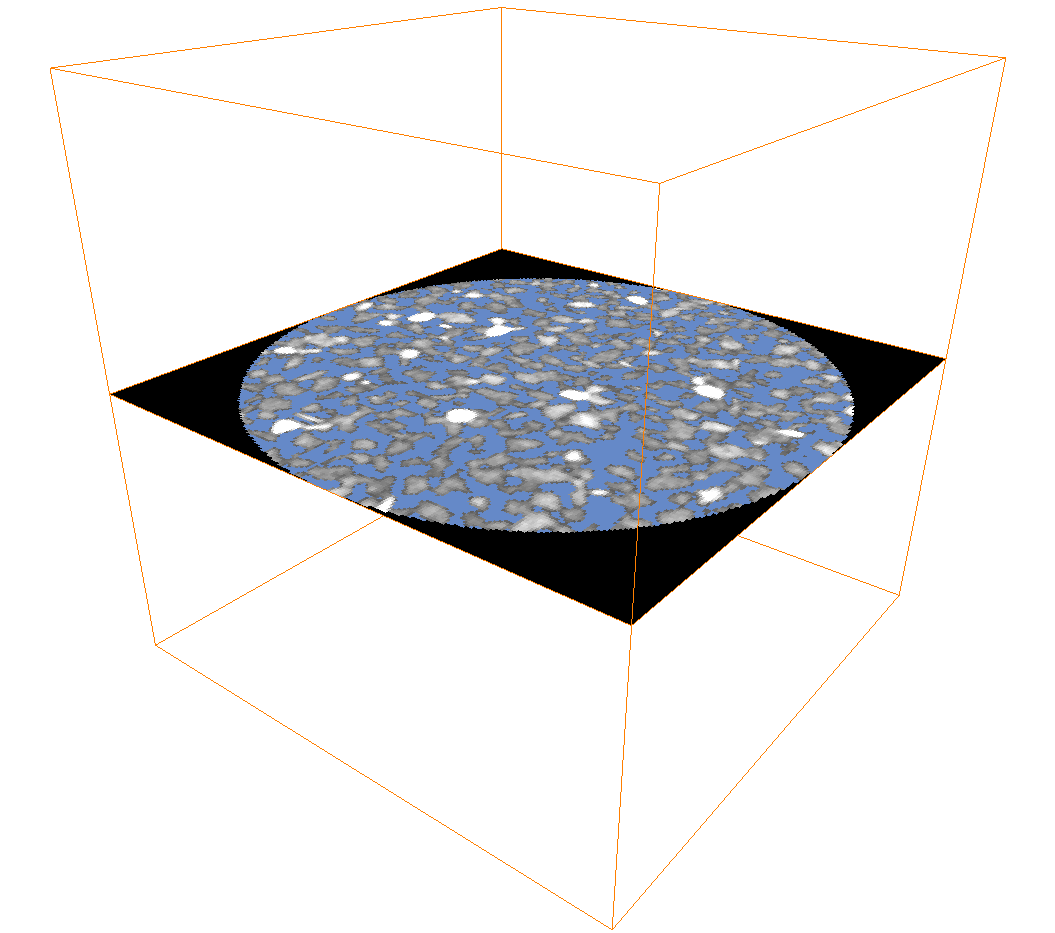

Supplement: S1 Data — (ZIP) [file pone.0296437.s001.zip › SI-Data/Data aggregation/unbiomineralization sample/3D unbiomineralization sample/12-xy.png]

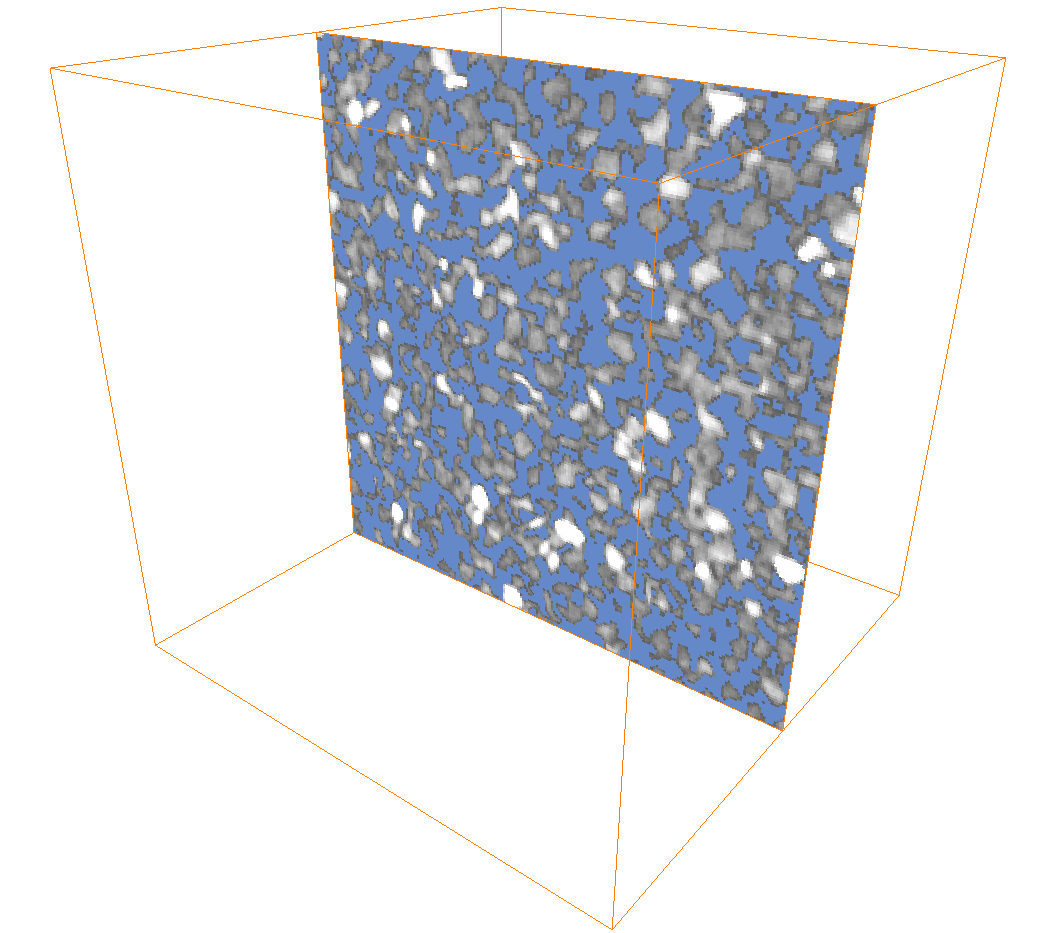

Supplement: S1 Data — (ZIP) [file pone.0296437.s001.zip › SI-Data/Data aggregation/unbiomineralization sample/3D unbiomineralization sample/12-yz.png]

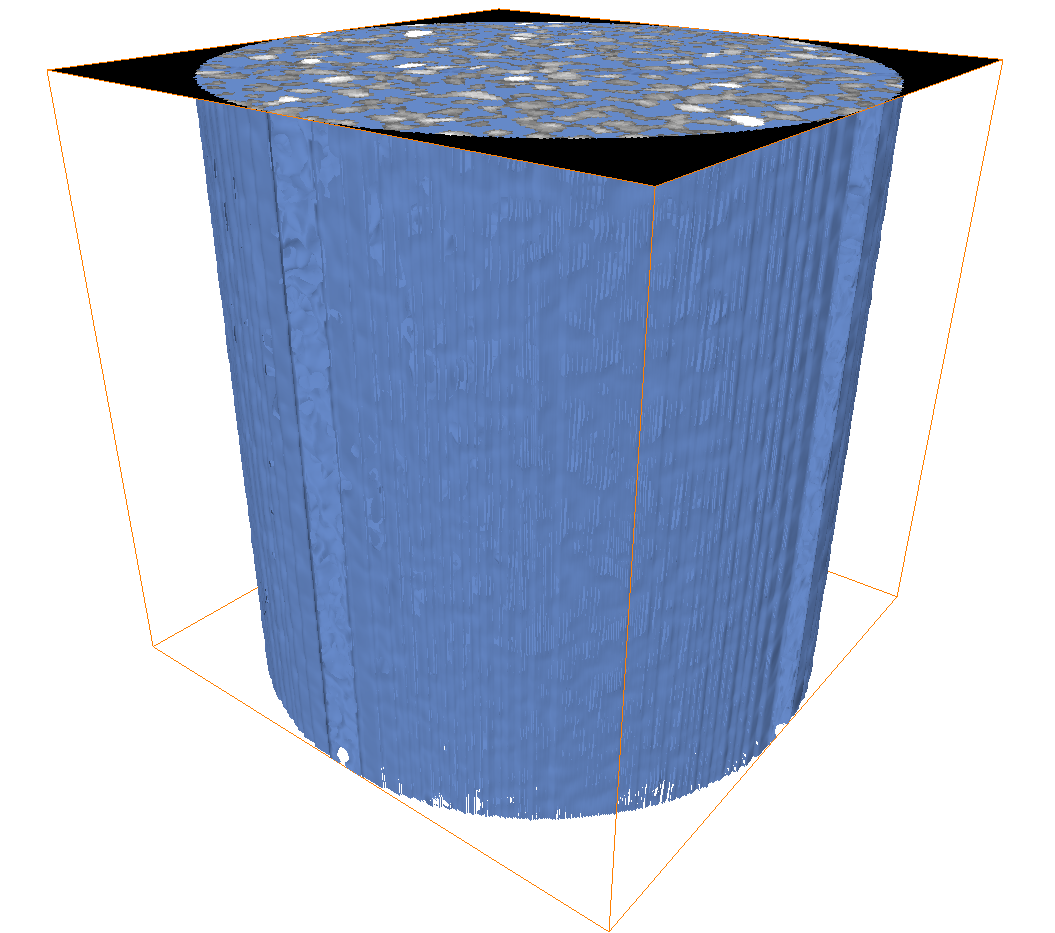

Supplement: S1 Data — (ZIP) [file pone.0296437.s001.zip › SI-Data/Data aggregation/unbiomineralization sample/3D unbiomineralization sample/13-xy1.png]

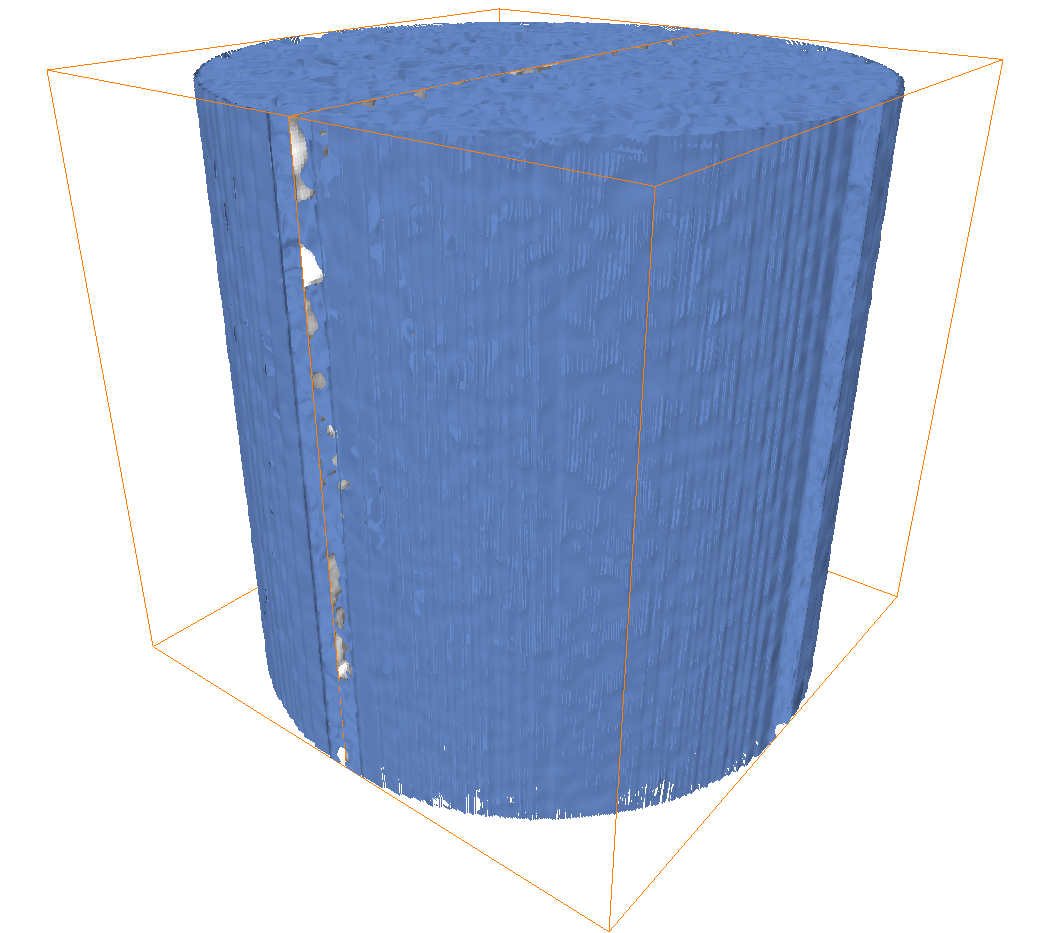

Supplement: S1 Data — (ZIP) [file pone.0296437.s001.zip › SI-Data/Data aggregation/unbiomineralization sample/3D unbiomineralization sample/13-xz.png]

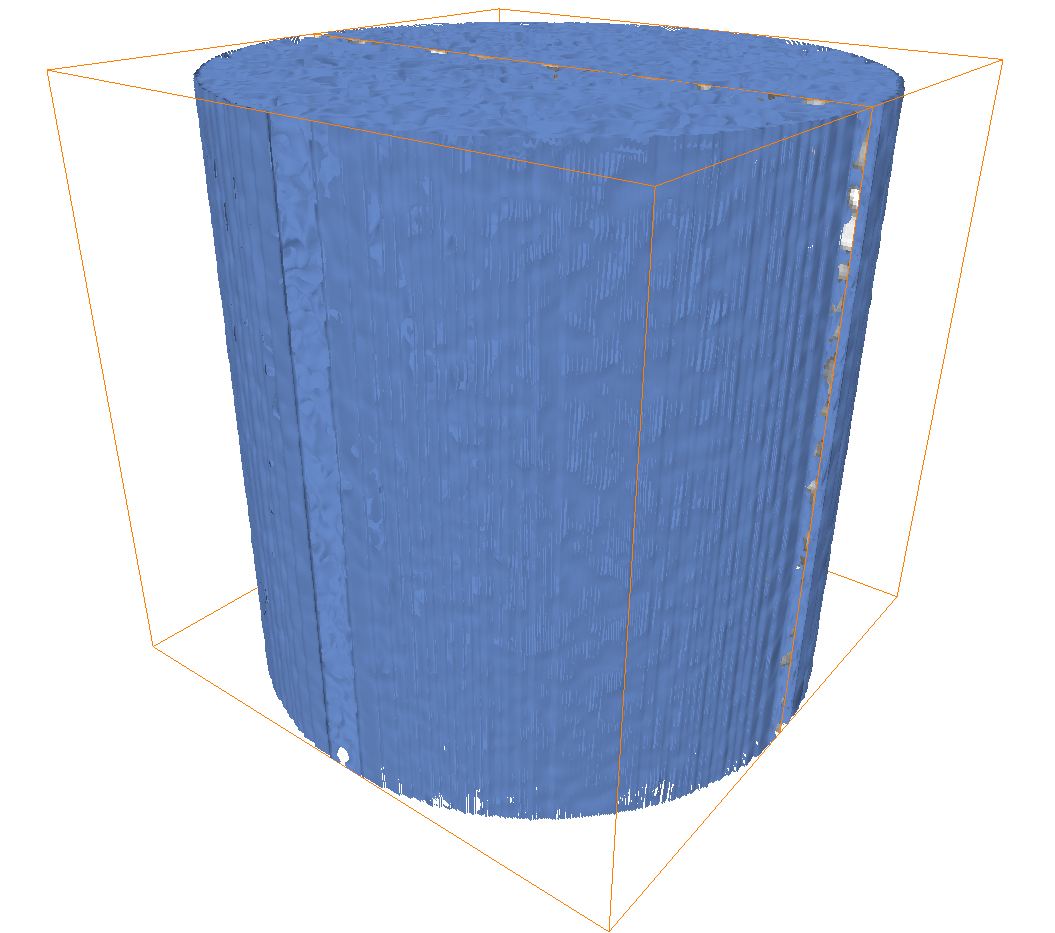

Supplement: S1 Data — (ZIP) [file pone.0296437.s001.zip › SI-Data/Data aggregation/unbiomineralization sample/3D unbiomineralization sample/13-yz.png]

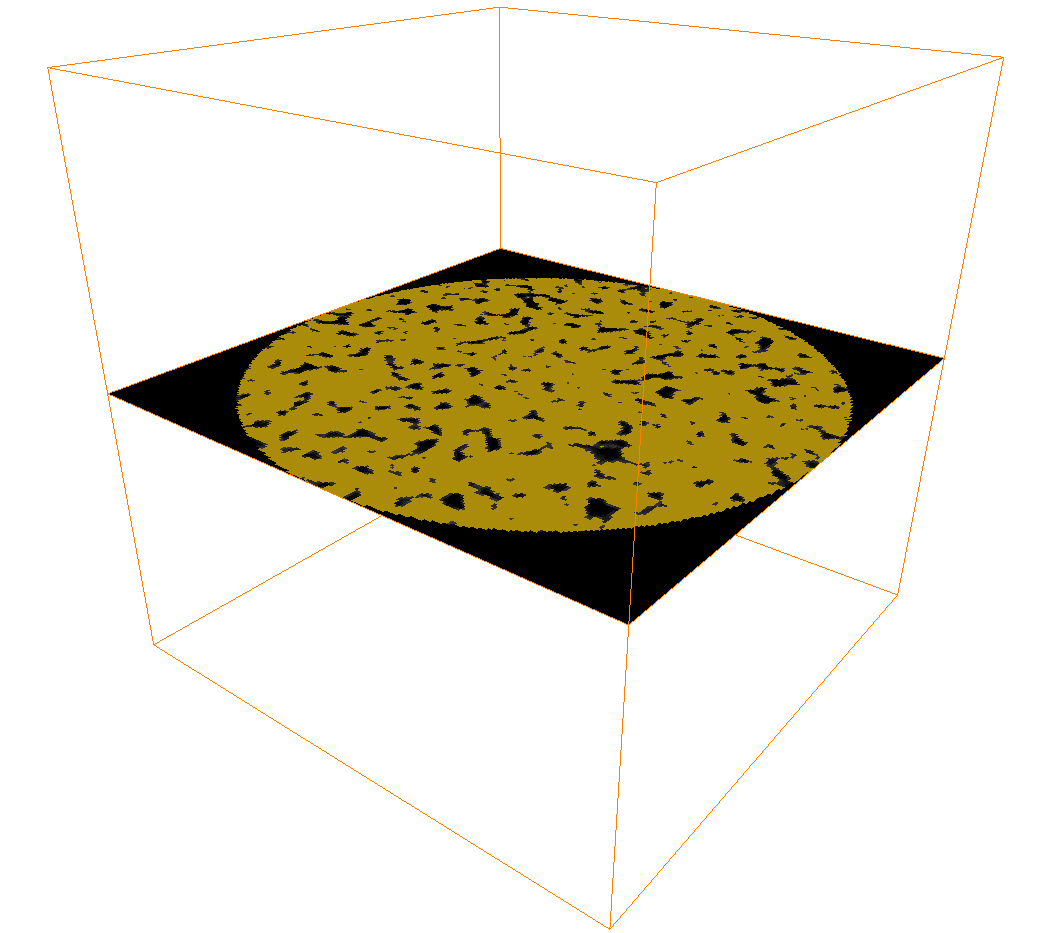

Supplement: S1 Data — (ZIP) [file pone.0296437.s001.zip › SI-Data/Data aggregation/unbiomineralization sample/3D unbiomineralization sample/14-xy.png]

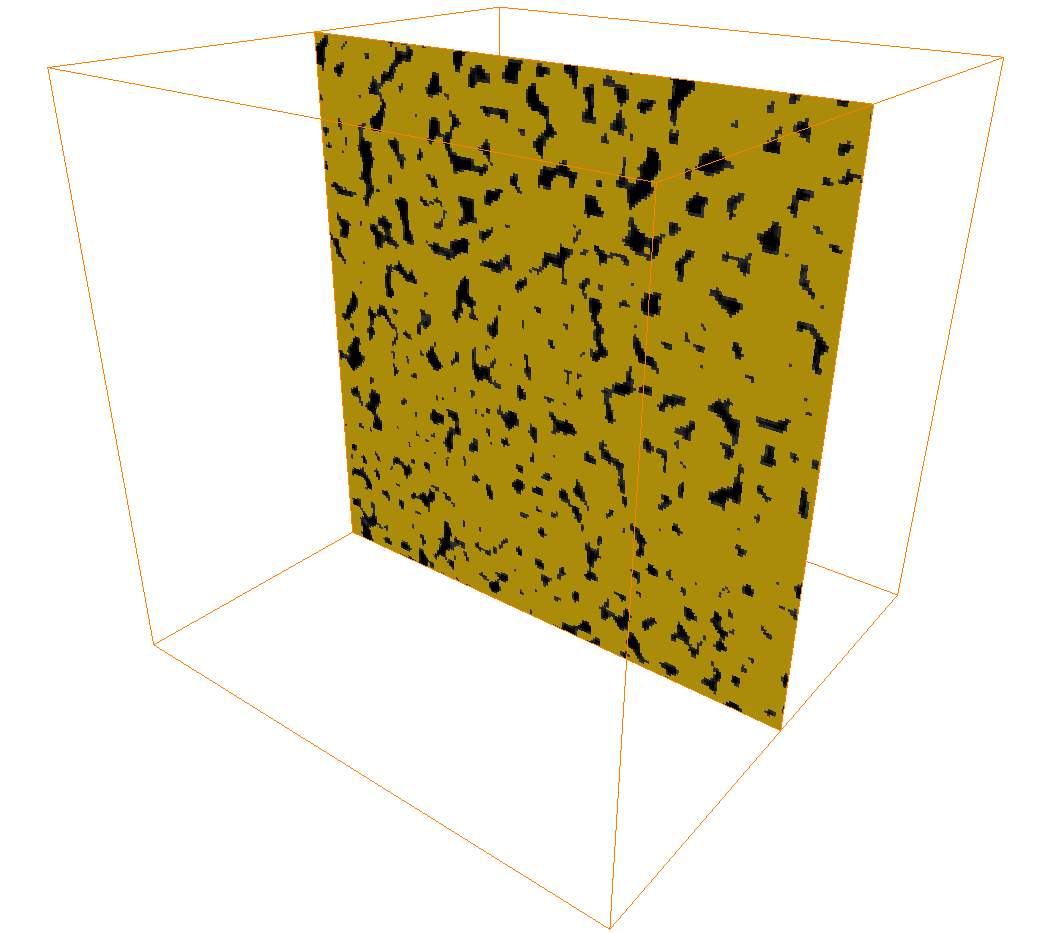

Supplement: S1 Data — (ZIP) [file pone.0296437.s001.zip › SI-Data/Data aggregation/unbiomineralization sample/3D unbiomineralization sample/14-yz.png]

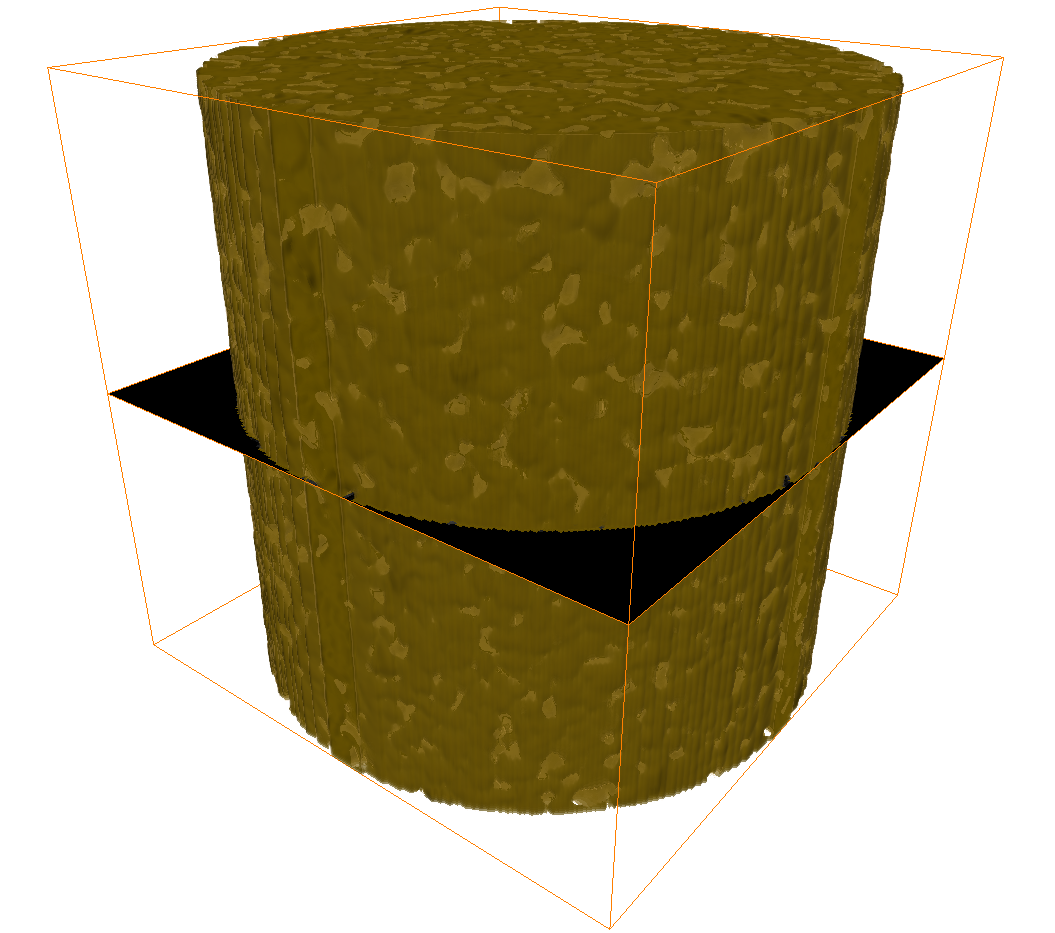

Supplement: S1 Data — (ZIP) [file pone.0296437.s001.zip › SI-Data/Data aggregation/unbiomineralization sample/3D unbiomineralization sample/15-xy.png]

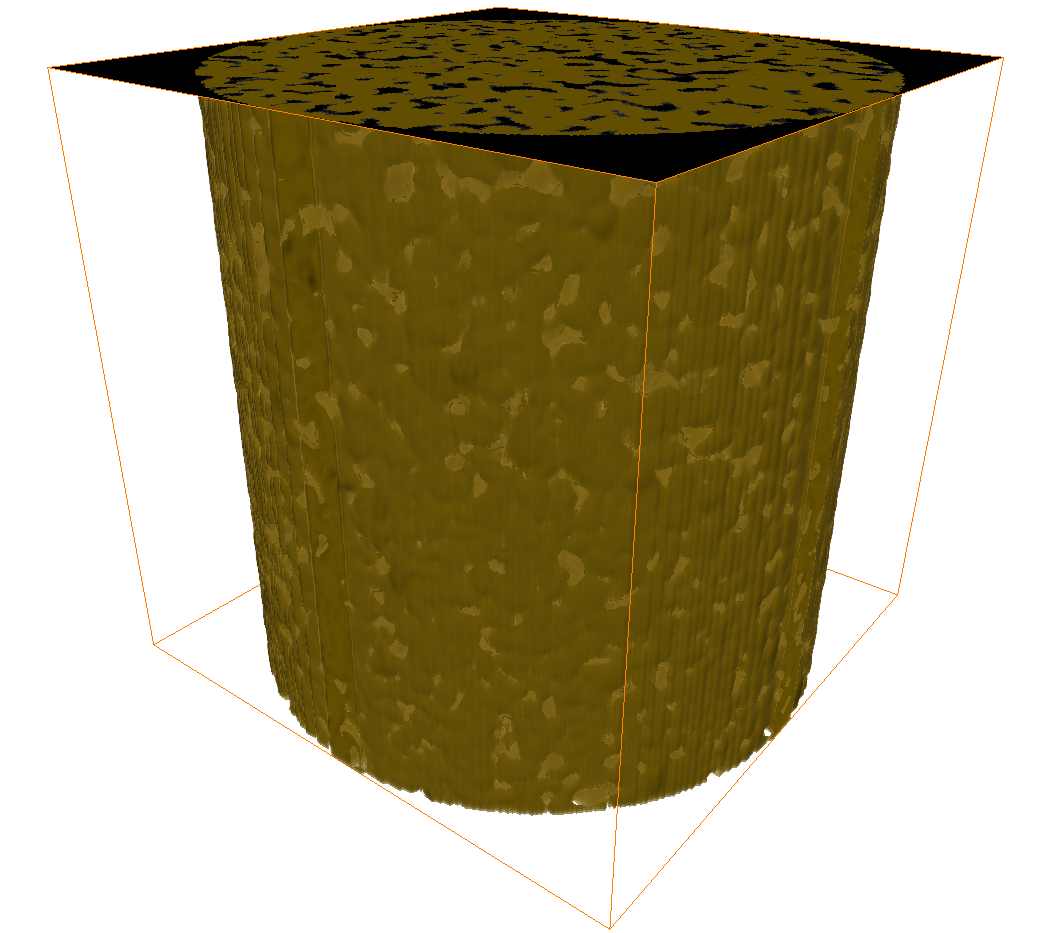

Supplement: S1 Data — (ZIP) [file pone.0296437.s001.zip › SI-Data/Data aggregation/unbiomineralization sample/3D unbiomineralization sample/15-xy1.png]

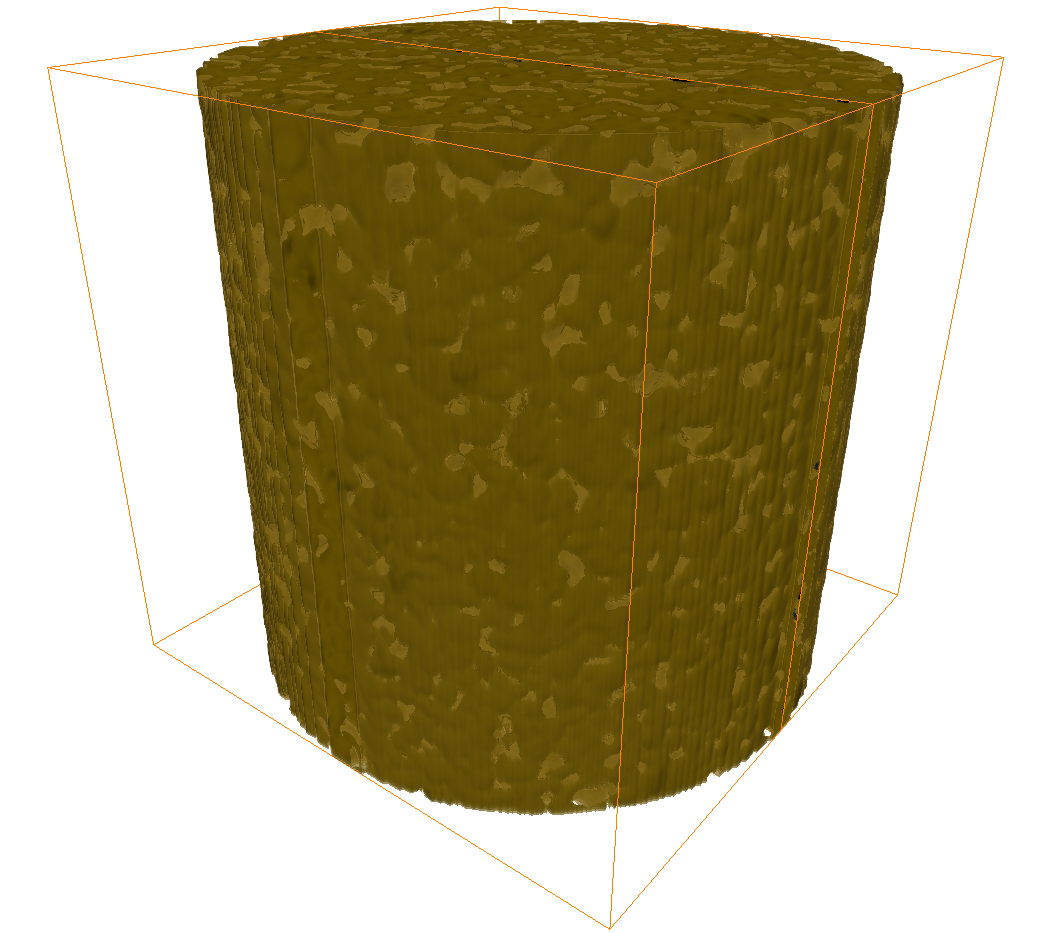

Supplement: S1 Data — (ZIP) [file pone.0296437.s001.zip › SI-Data/Data aggregation/unbiomineralization sample/3D unbiomineralization sample/15-yz.png]

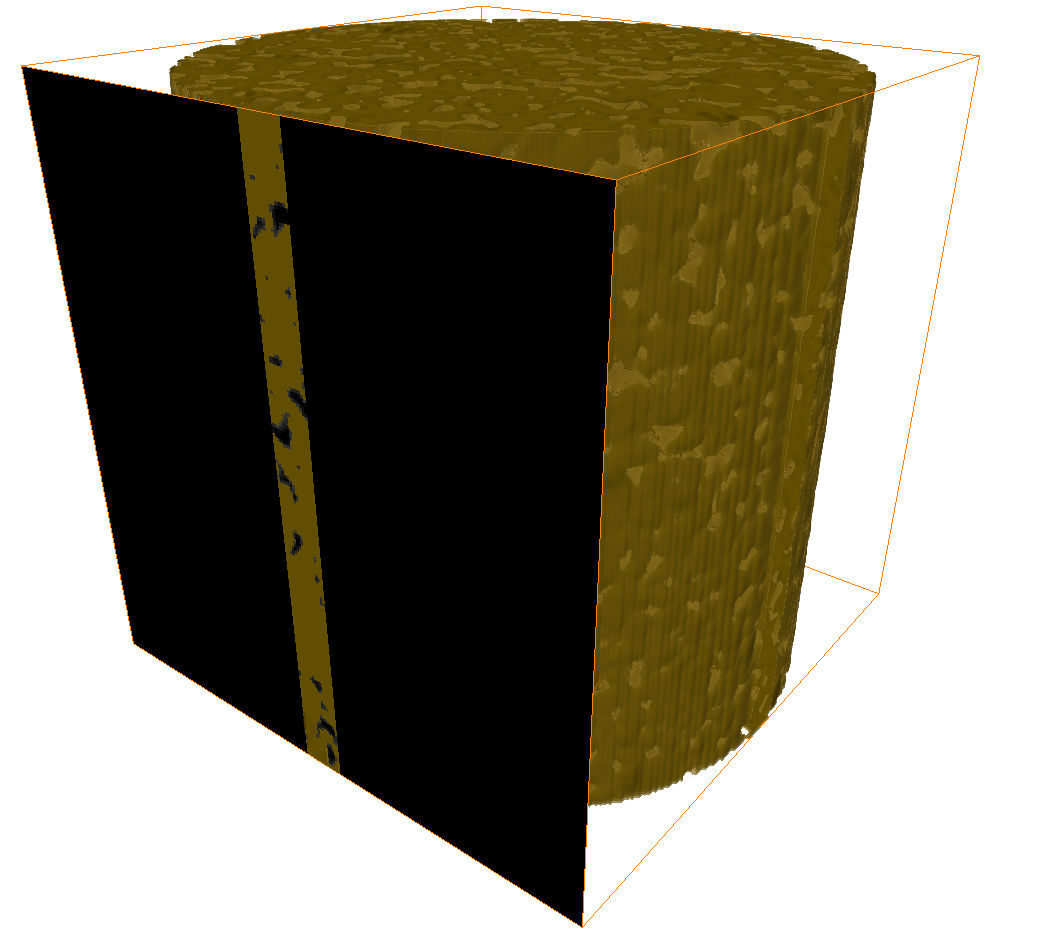

Supplement: S1 Data — (ZIP) [file pone.0296437.s001.zip › SI-Data/Data aggregation/unbiomineralization sample/3D unbiomineralization sample/15-yz1.png]

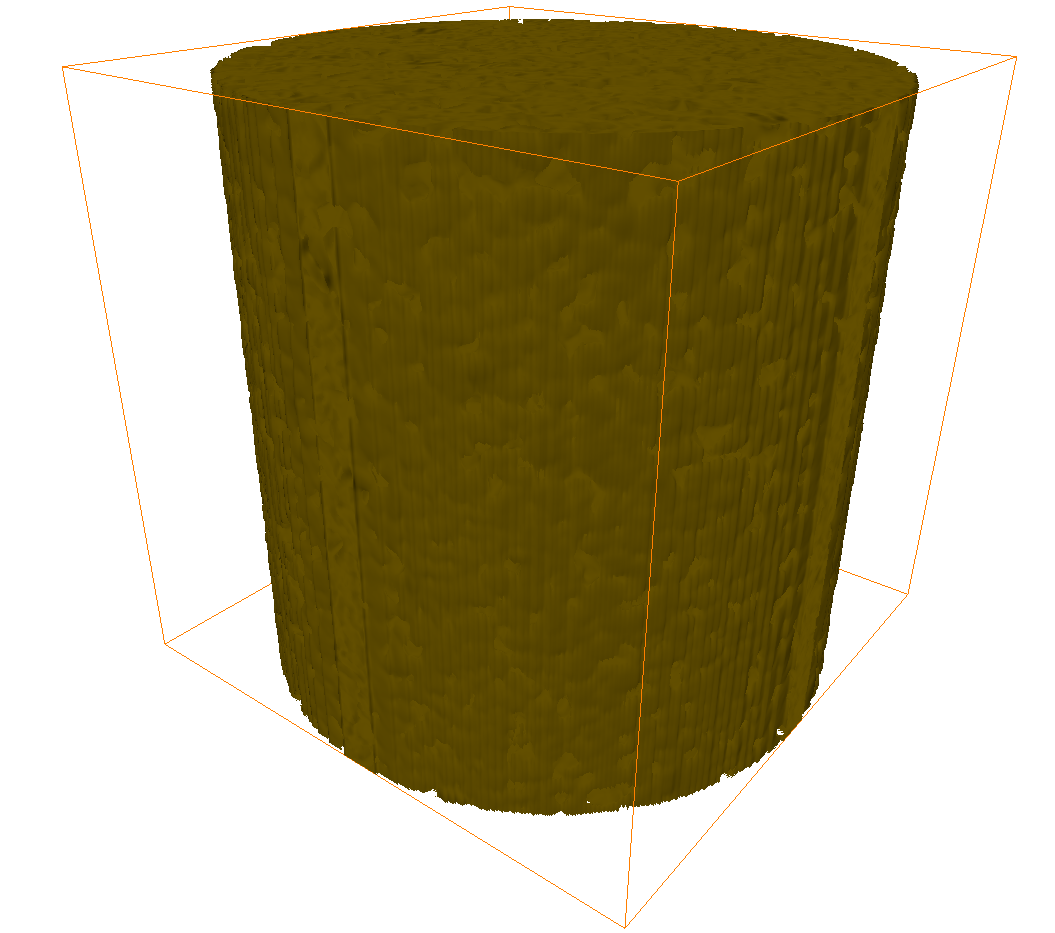

Supplement: S1 Data — (ZIP) [file pone.0296437.s001.zip › SI-Data/Data aggregation/unbiomineralization sample/3D unbiomineralization sample/16-.png]

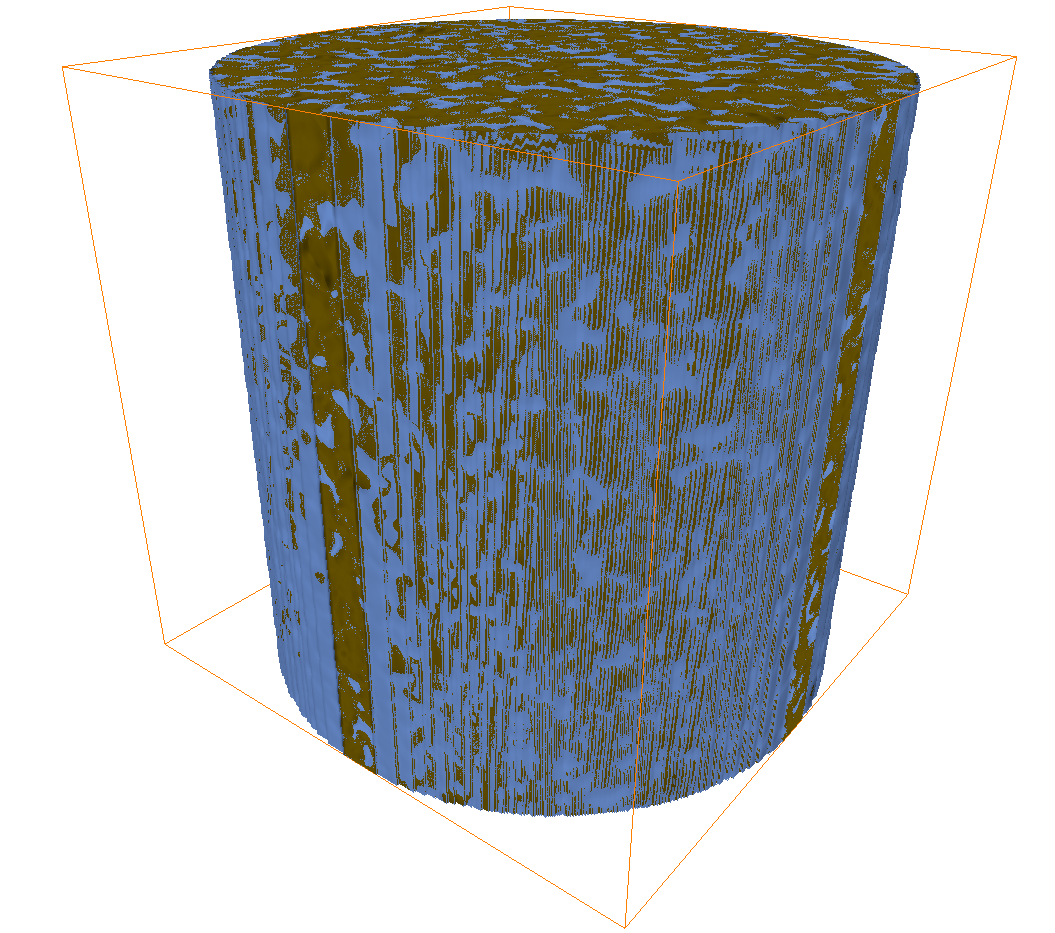

Supplement: S1 Data — (ZIP) [file pone.0296437.s001.zip › SI-Data/Data aggregation/unbiomineralization sample/3D unbiomineralization sample/17-.png]

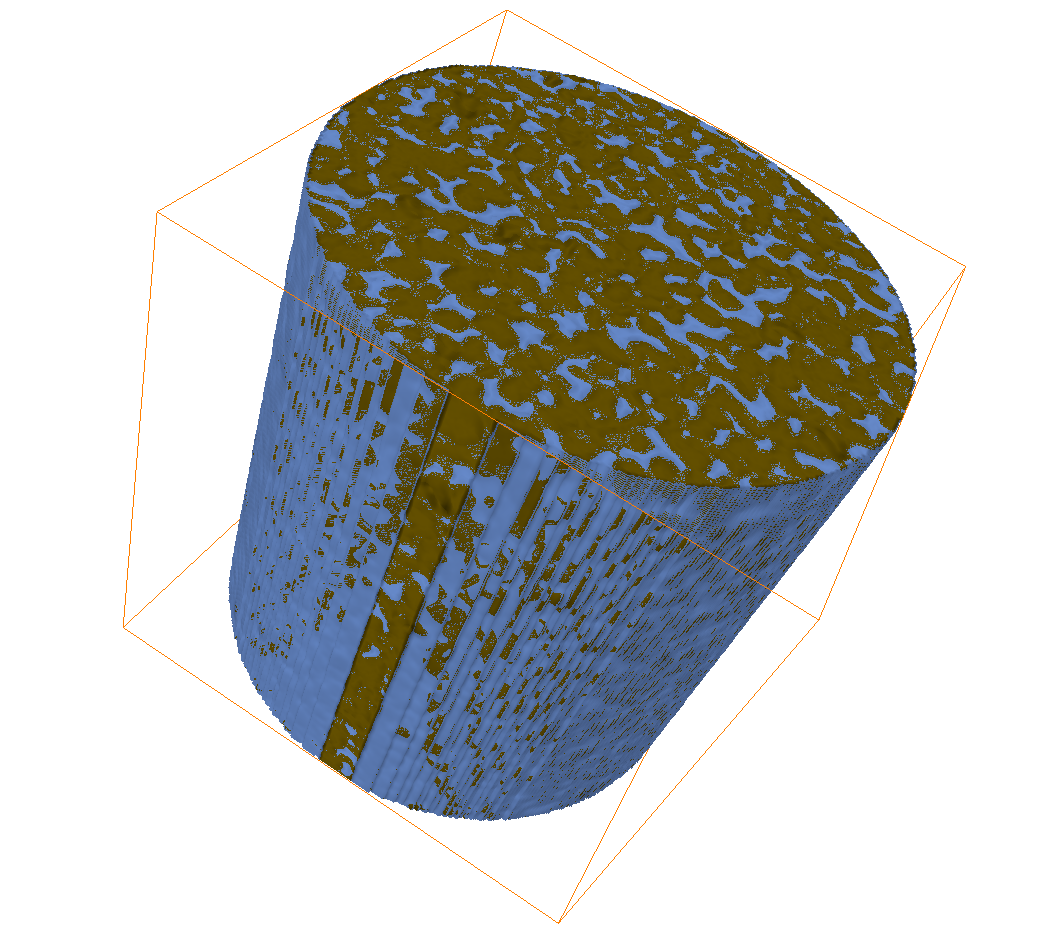

Supplement: S1 Data — (ZIP) [file pone.0296437.s001.zip › SI-Data/Data aggregation/unbiomineralization sample/3D unbiomineralization sample/17-1.png]

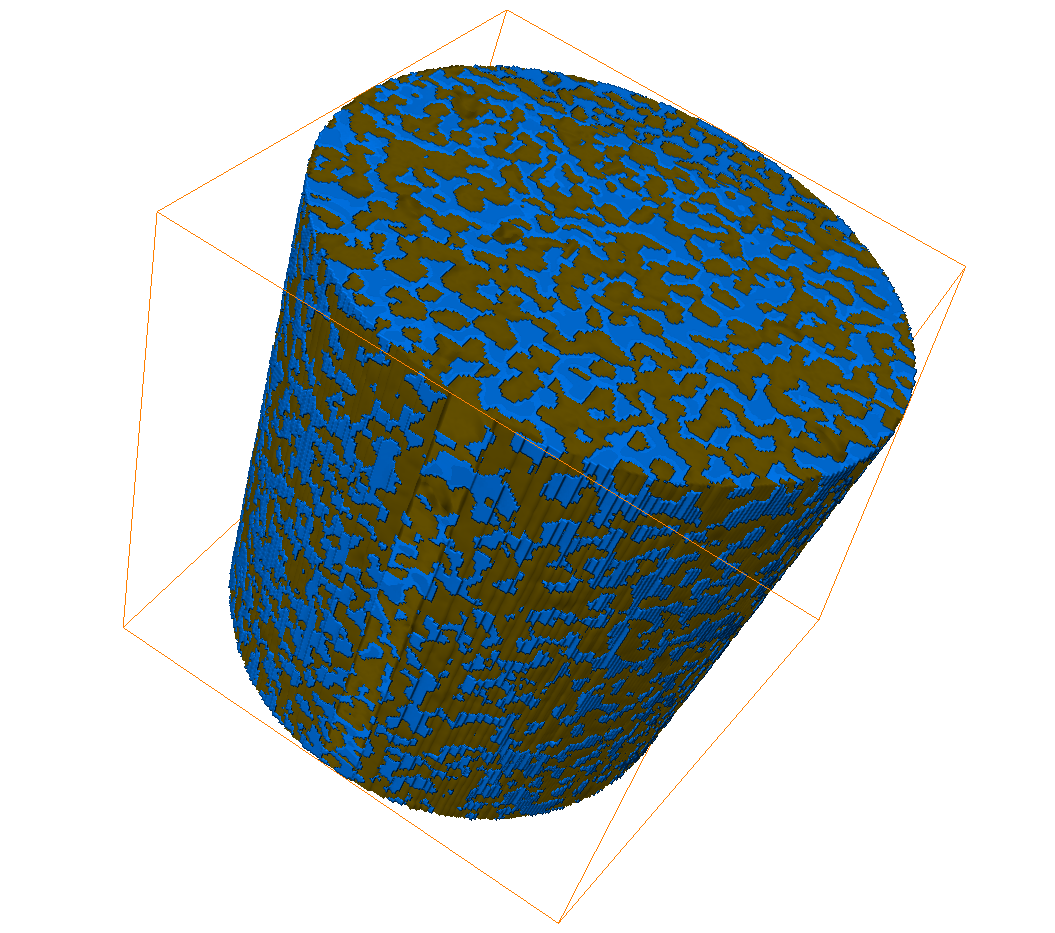

Supplement: S1 Data — (ZIP) [file pone.0296437.s001.zip › SI-Data/Data aggregation/unbiomineralization sample/3D unbiomineralization sample/17-2.png]

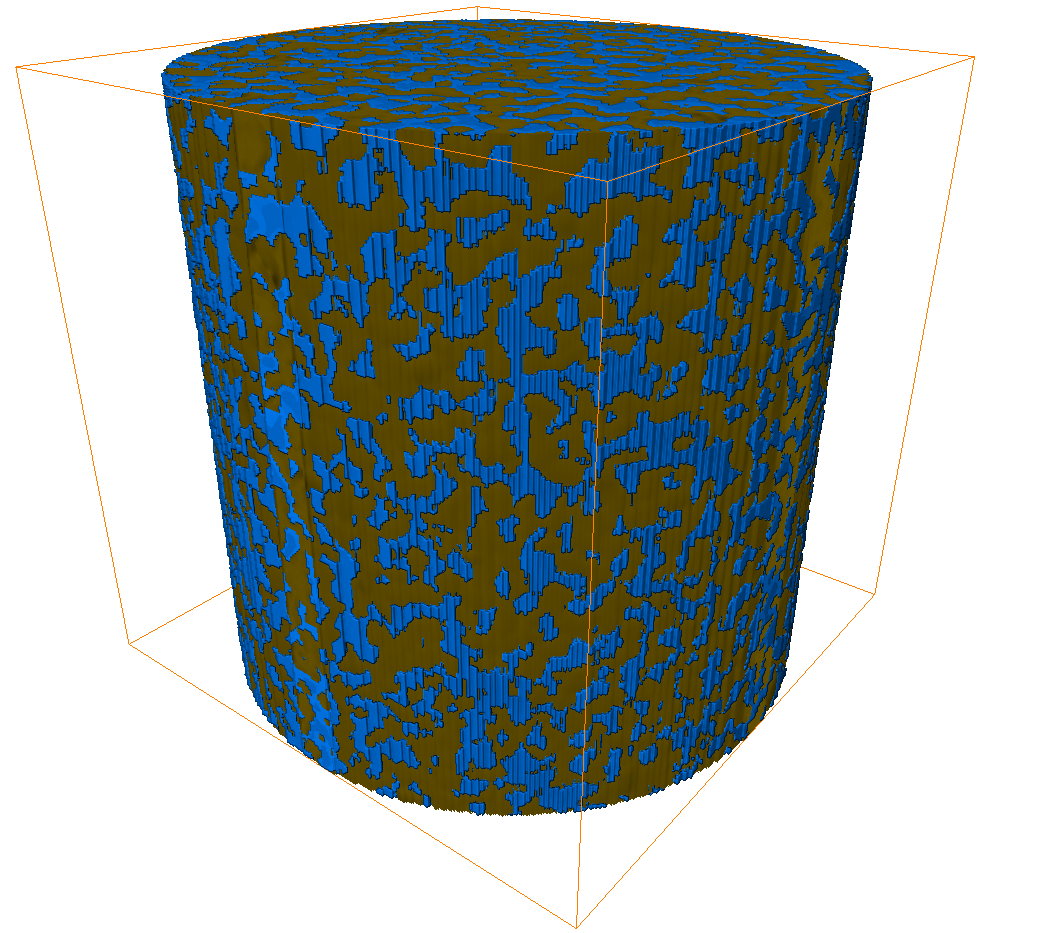

Supplement: S1 Data — (ZIP) [file pone.0296437.s001.zip › SI-Data/Data aggregation/unbiomineralization sample/3D unbiomineralization sample/17-3.png]

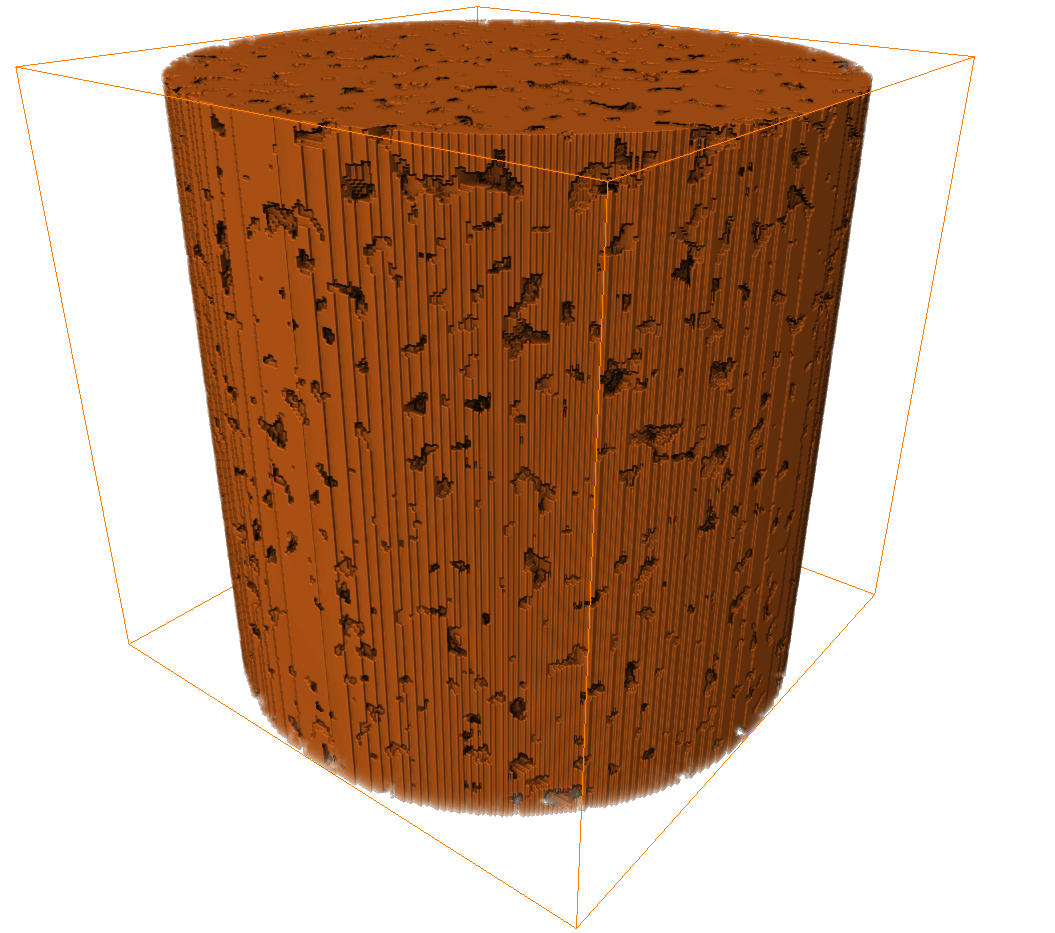

Supplement: S1 Data — (ZIP) [file pone.0296437.s001.zip › SI-Data/Data aggregation/unbiomineralization sample/3D unbiomineralization sample/18.png]

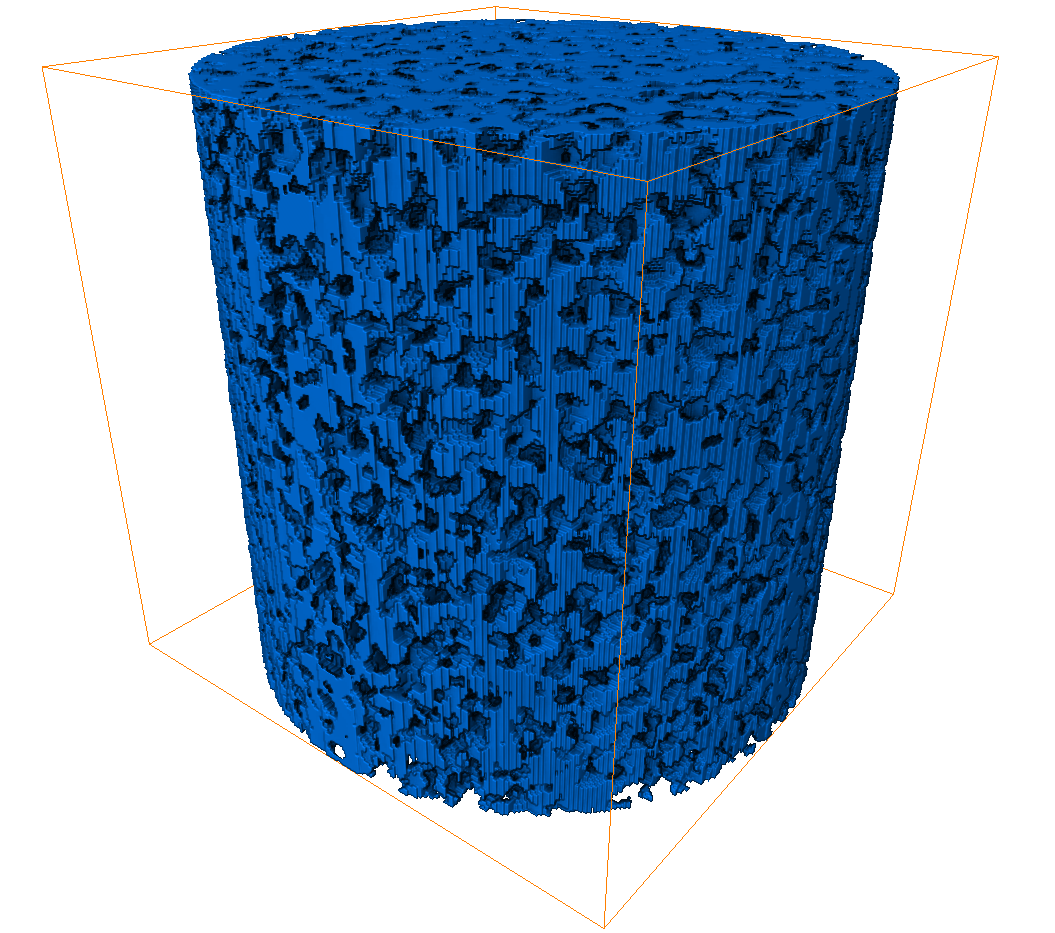

Supplement: S1 Data — (ZIP) [file pone.0296437.s001.zip › SI-Data/Data aggregation/unbiomineralization sample/3D unbiomineralization sample/20-hanyou gulikongxi .png]

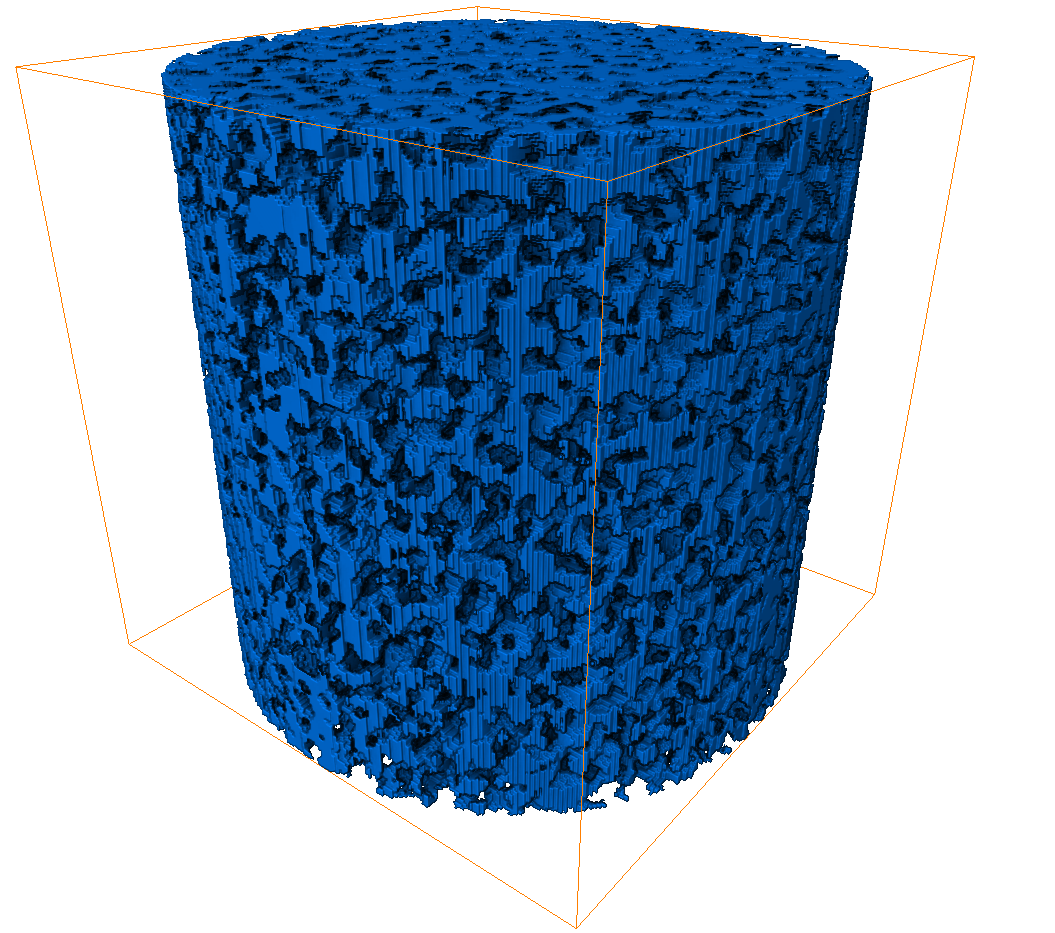

Supplement: S1 Data — (ZIP) [file pone.0296437.s001.zip › SI-Data/Data aggregation/unbiomineralization sample/3D unbiomineralization sample/20.png]

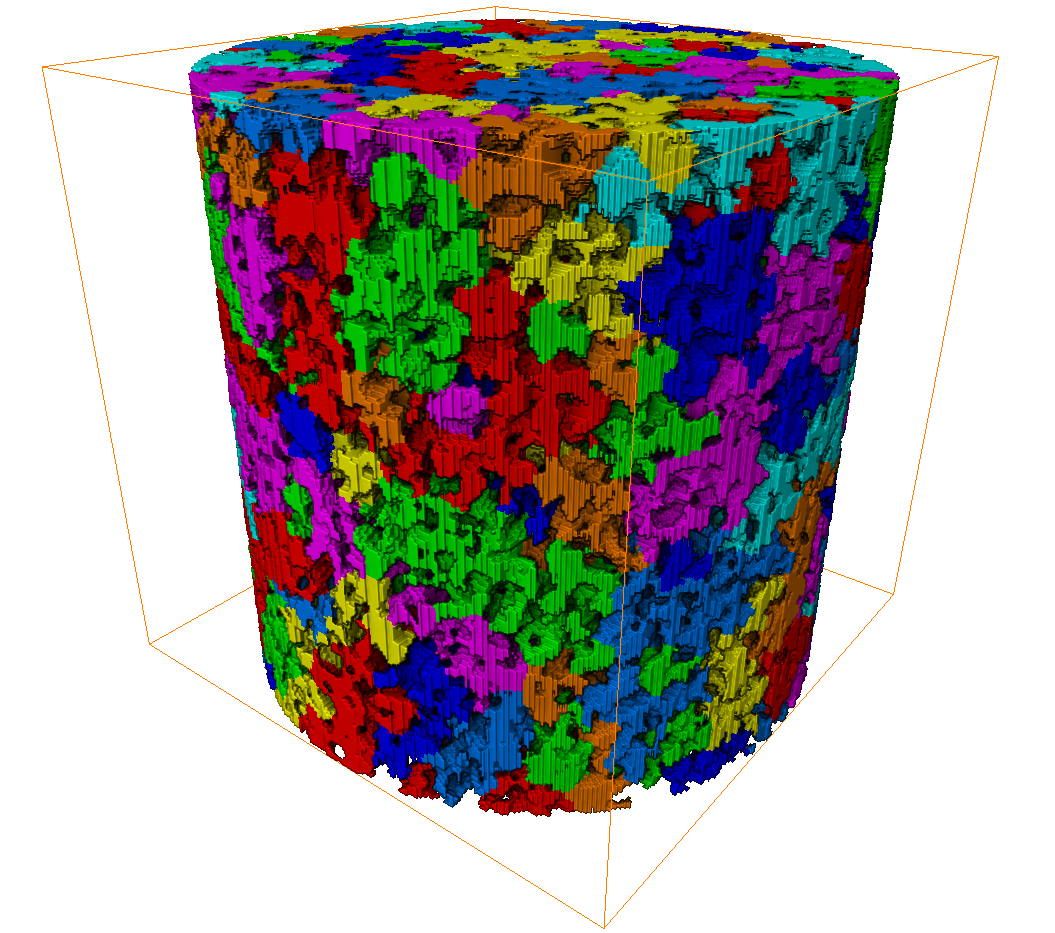

Supplement: S1 Data — (ZIP) [file pone.0296437.s001.zip › SI-Data/Data aggregation/unbiomineralization sample/3D unbiomineralization sample/22-gulikongxi fenge .png]

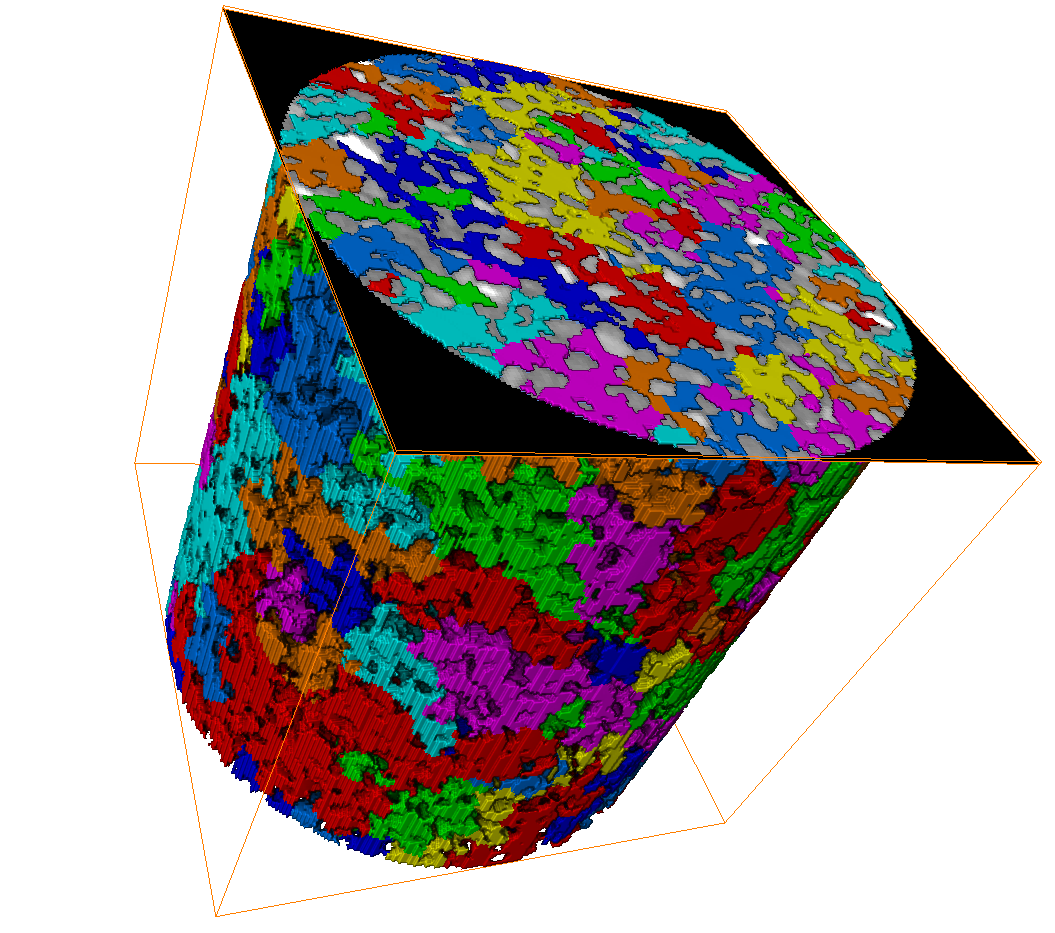

Supplement: S1 Data — (ZIP) [file pone.0296437.s001.zip › SI-Data/Data aggregation/unbiomineralization sample/3D unbiomineralization sample/22-gulikongxi fenge 1 .png]

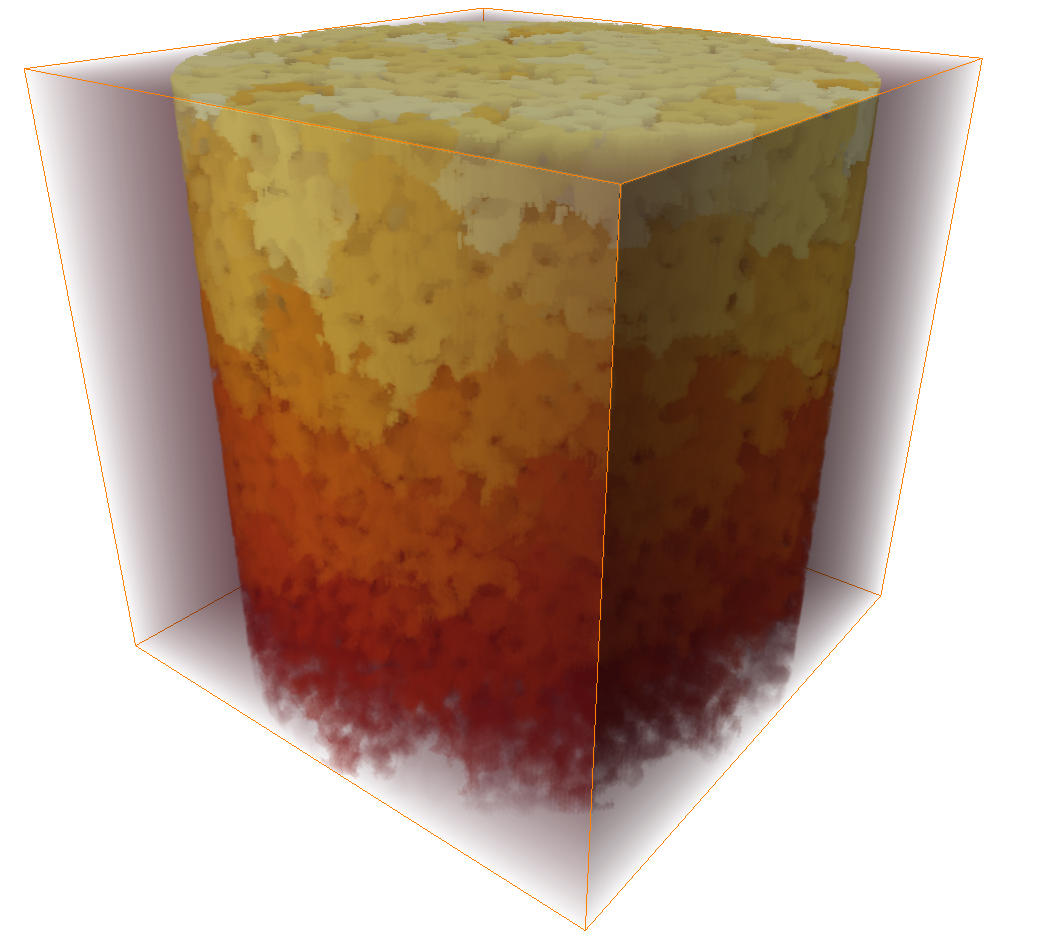

Supplement: S1 Data — (ZIP) [file pone.0296437.s001.zip › SI-Data/Data aggregation/unbiomineralization sample/3D unbiomineralization sample/22-gulikongxi fenge 1 .png]

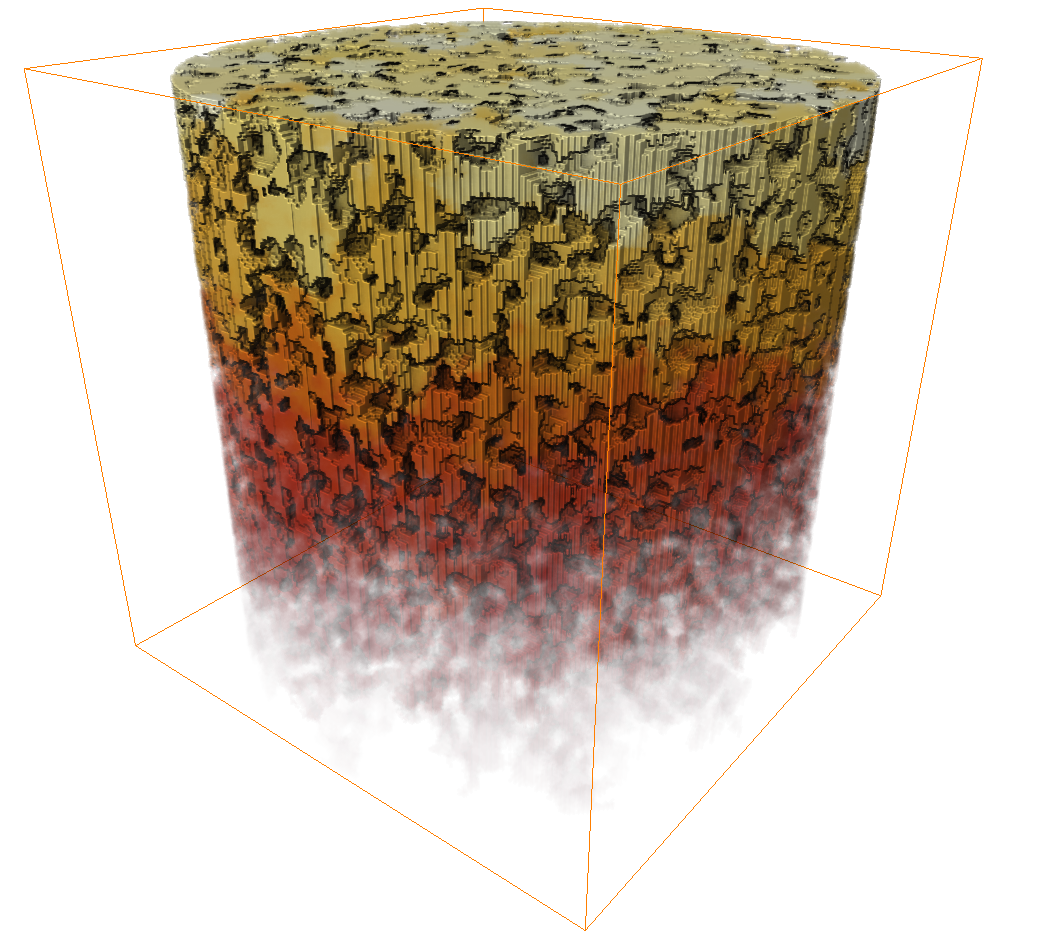

Supplement: S1 Data — (ZIP) [file pone.0296437.s001.zip › SI-Data/Data aggregation/unbiomineralization sample/3D unbiomineralization sample/22-gulikongxi fenge 2 .png]

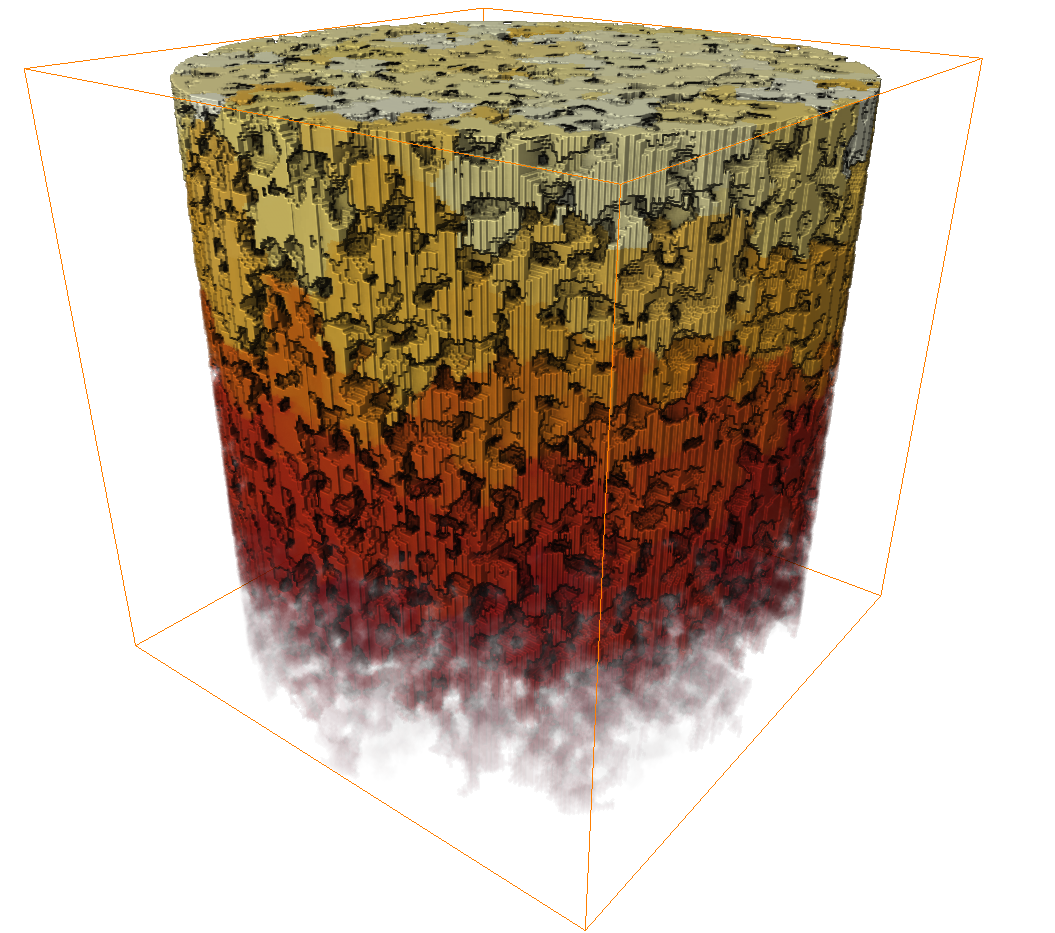

Supplement: S1 Data — (ZIP) [file pone.0296437.s001.zip › SI-Data/Data aggregation/unbiomineralization sample/3D unbiomineralization sample/22-gulikongxi fenge 3 .png]

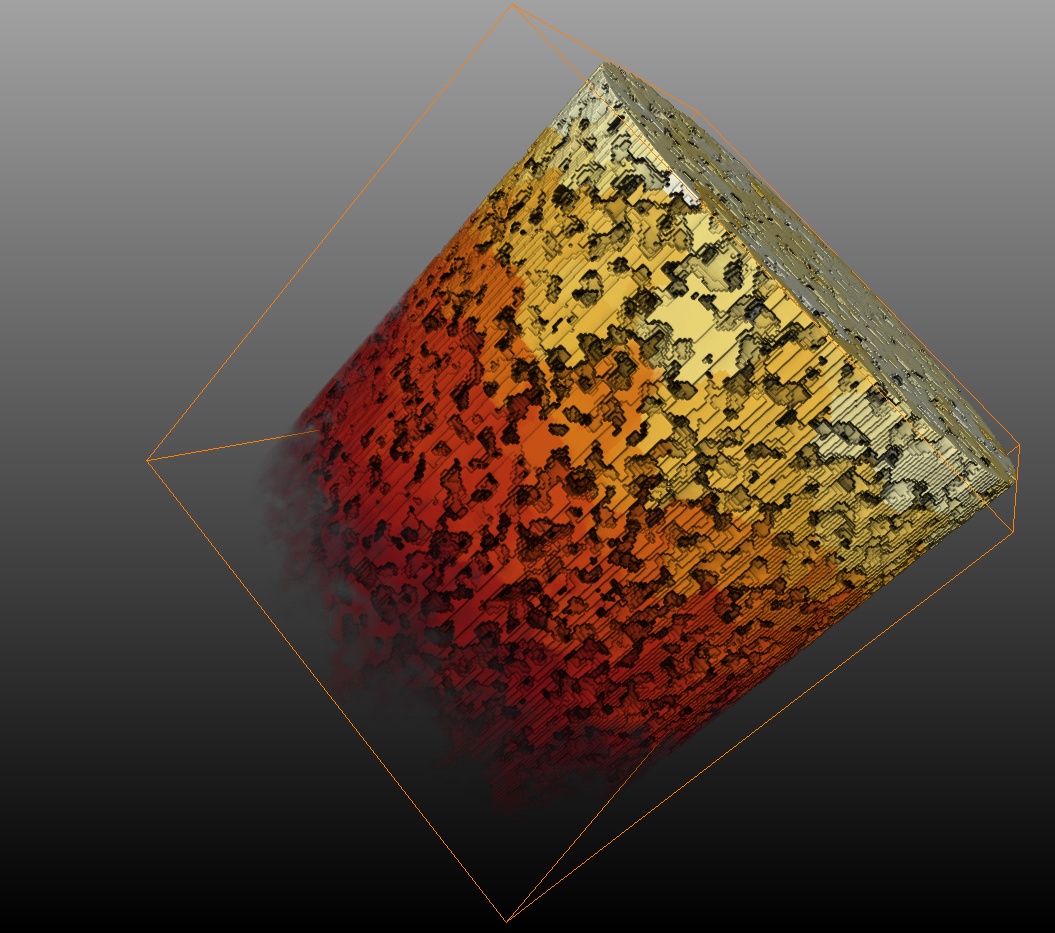

Supplement: S1 Data — (ZIP) [file pone.0296437.s001.zip › SI-Data/Data aggregation/unbiomineralization sample/3D unbiomineralization sample/22-gulikongxi fenge 5 .png]

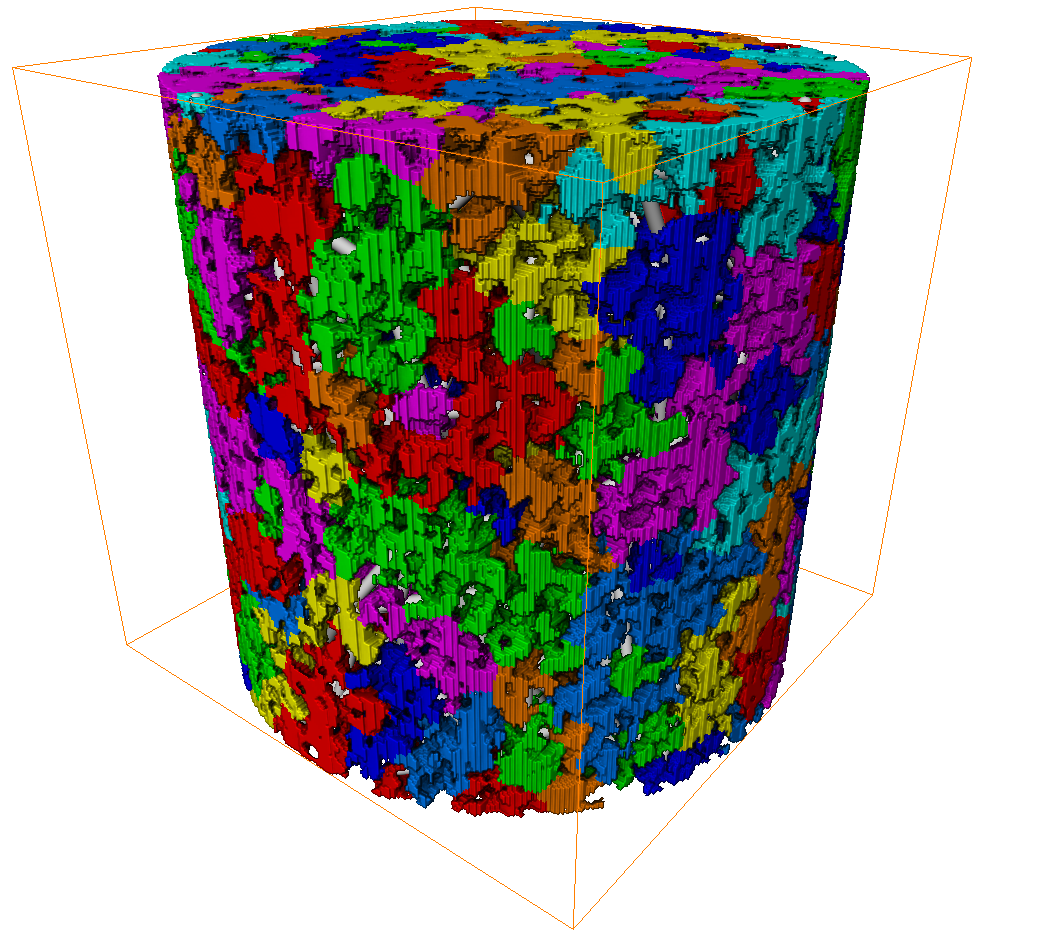

Supplement: S1 Data — (ZIP) [file pone.0296437.s001.zip › SI-Data/Data aggregation/unbiomineralization sample/3D unbiomineralization sample/23-gulikongxi fenge kongxiwangluomoxing .png]

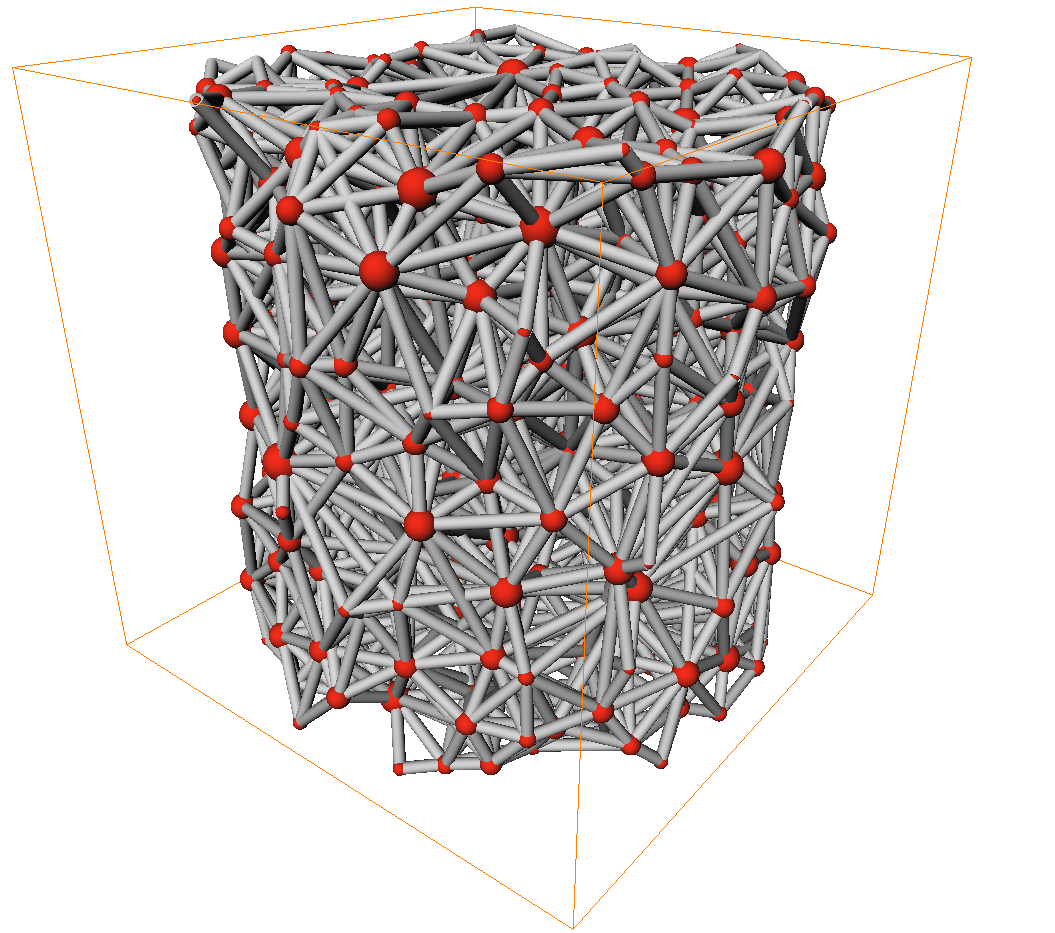

Supplement: S1 Data — (ZIP) [file pone.0296437.s001.zip › SI-Data/Data aggregation/unbiomineralization sample/3D unbiomineralization sample/24-kongxiwangluomoxing 2 .png]

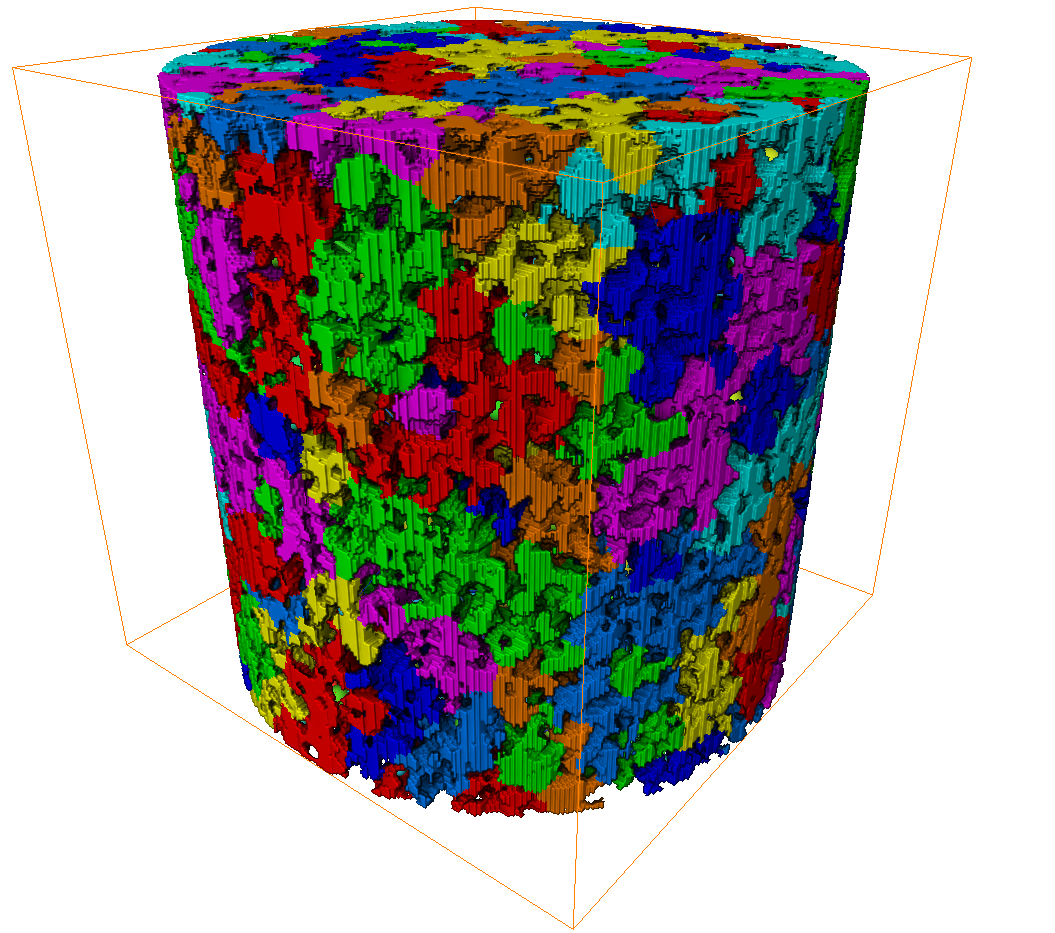

Supplement: S1 Data — (ZIP) [file pone.0296437.s001.zip › SI-Data/Data aggregation/unbiomineralization sample/3D unbiomineralization sample/24-kongxiwangluomoxing jia fenge .png]

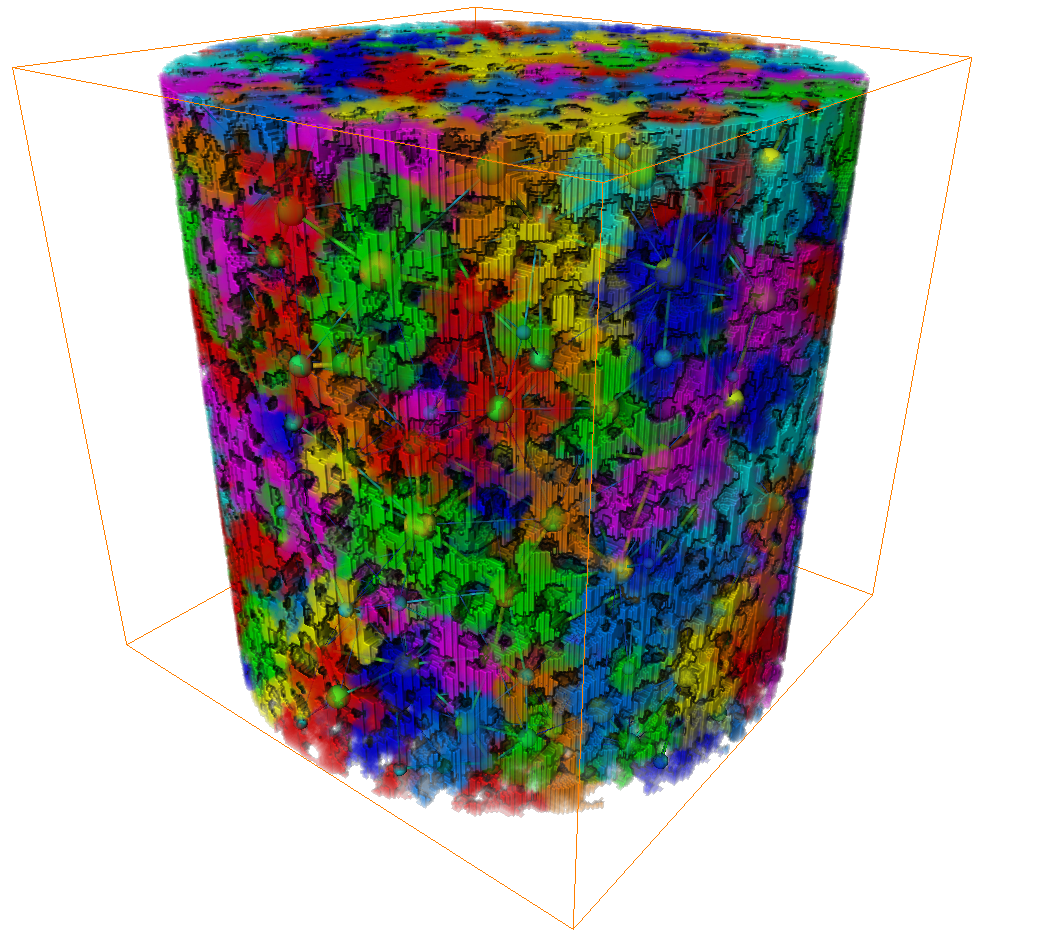

Supplement: S1 Data — (ZIP) [file pone.0296437.s001.zip › SI-Data/Data aggregation/unbiomineralization sample/3D unbiomineralization sample/24-kongxiwangluomoxing jia fenge 2 .png]

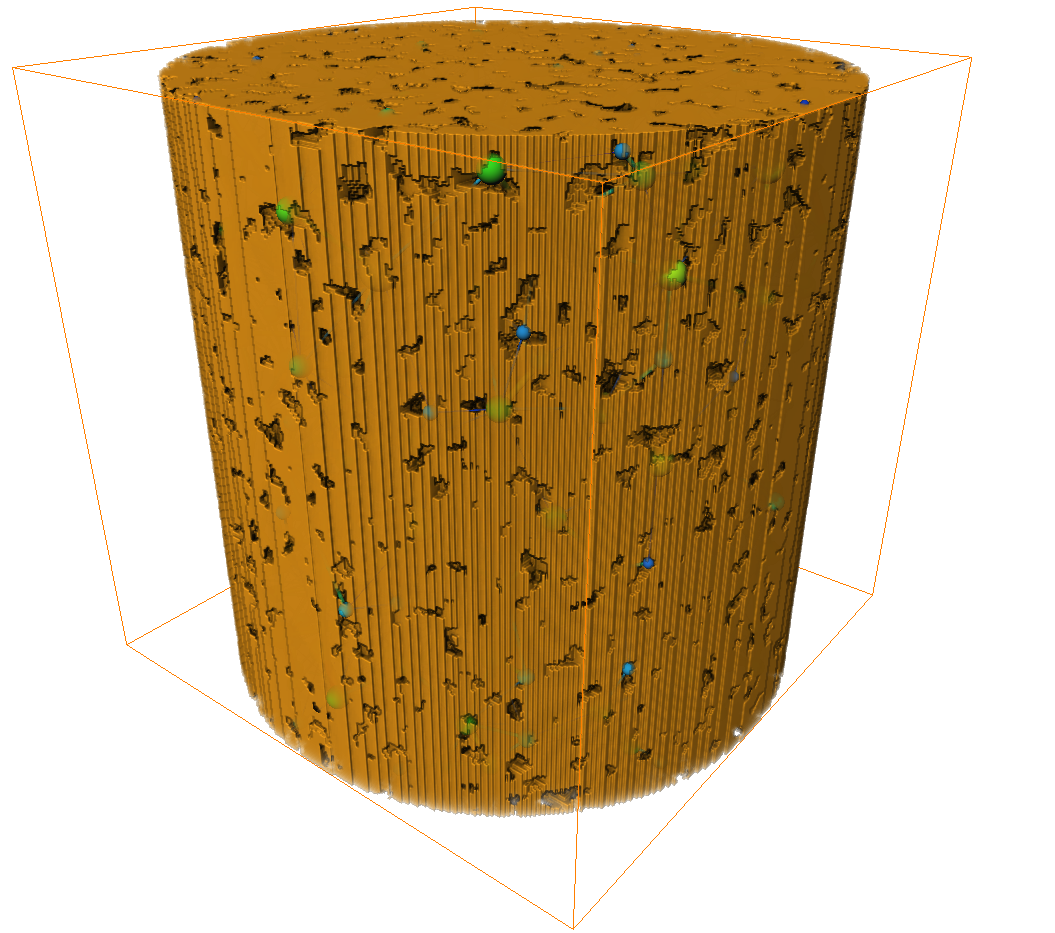

Supplement: S1 Data — (ZIP) [file pone.0296437.s001.zip › SI-Data/Data aggregation/unbiomineralization sample/3D unbiomineralization sample/24-kongxiwangluomoxing jia keli shiti .png]

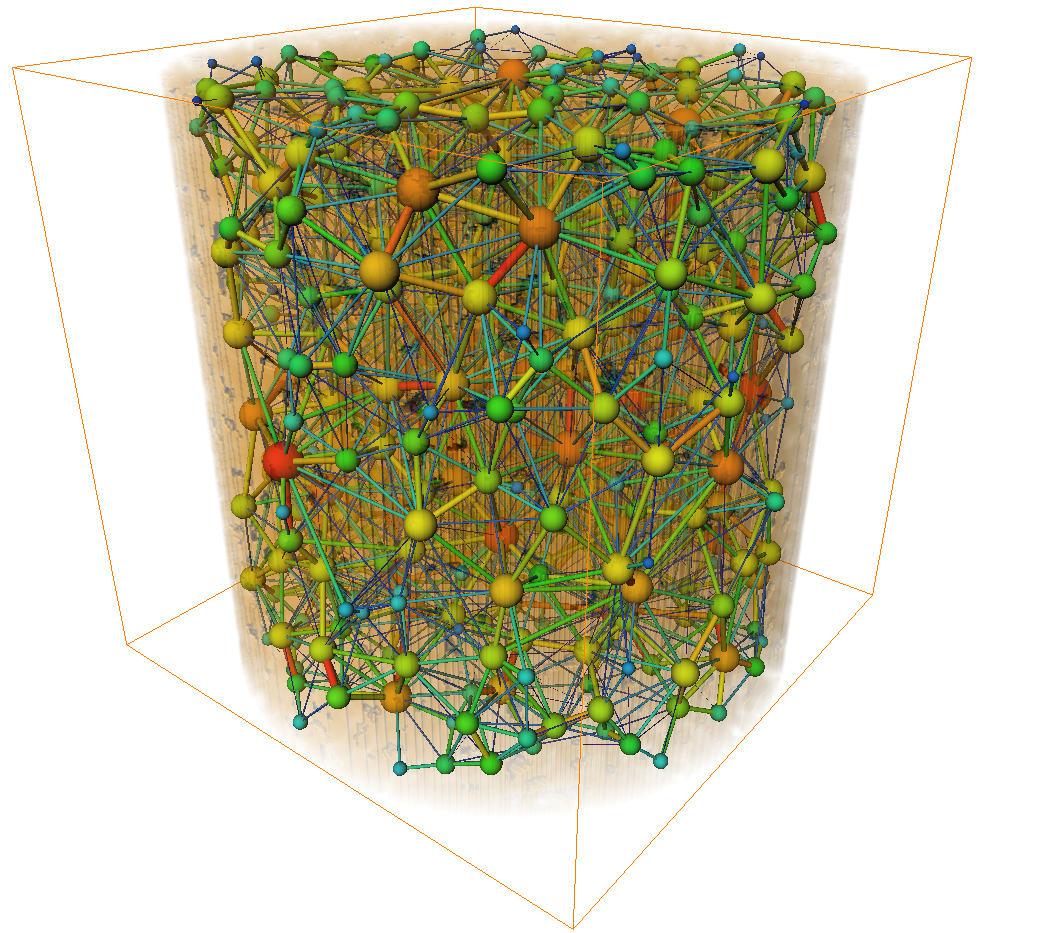

Supplement: S1 Data — (ZIP) [file pone.0296437.s001.zip › SI-Data/Data aggregation/unbiomineralization sample/3D unbiomineralization sample/24-kongxiwangluomoxing jia keli shiti 2 .png]

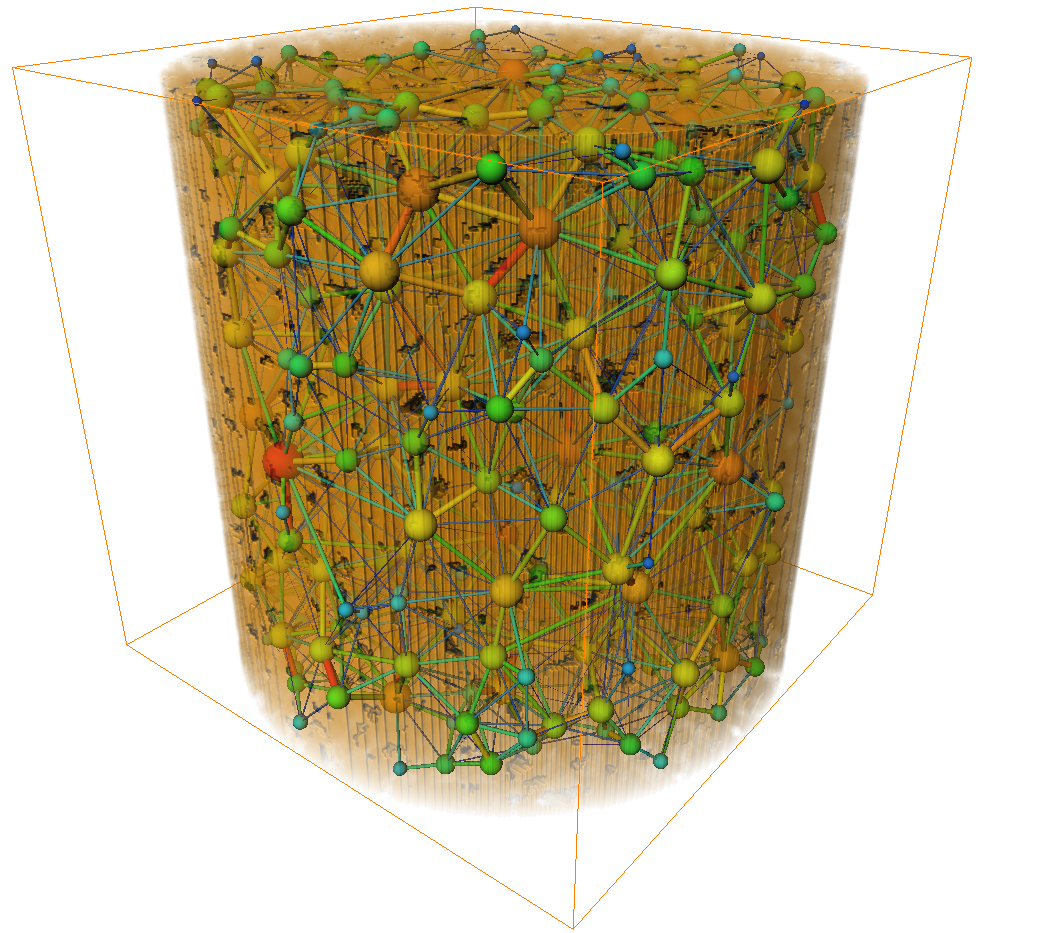

Supplement: S1 Data — (ZIP) [file pone.0296437.s001.zip › SI-Data/Data aggregation/unbiomineralization sample/3D unbiomineralization sample/24-kongxiwangluomoxing jia keli shiti 3 .png]

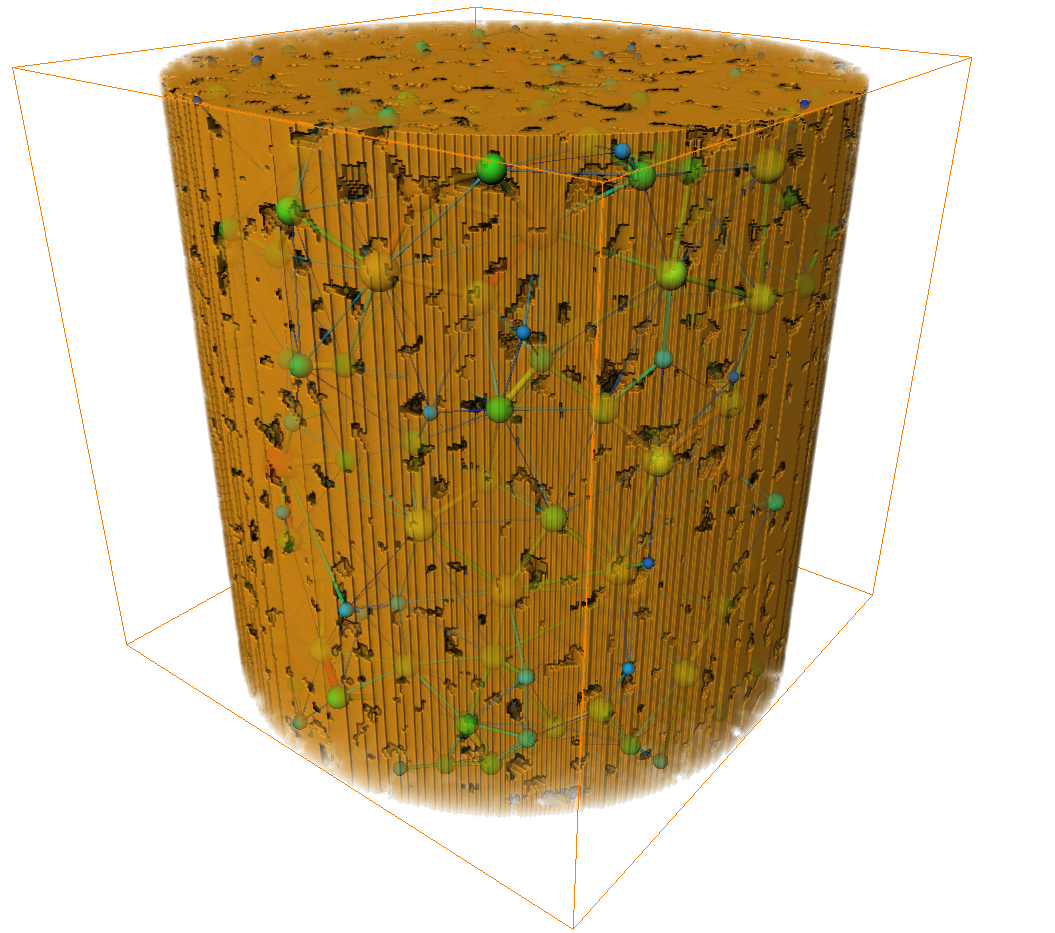

Supplement: S1 Data — (ZIP) [file pone.0296437.s001.zip › SI-Data/Data aggregation/unbiomineralization sample/3D unbiomineralization sample/24-kongxiwangluomoxing jia keli shiti 4 .png]

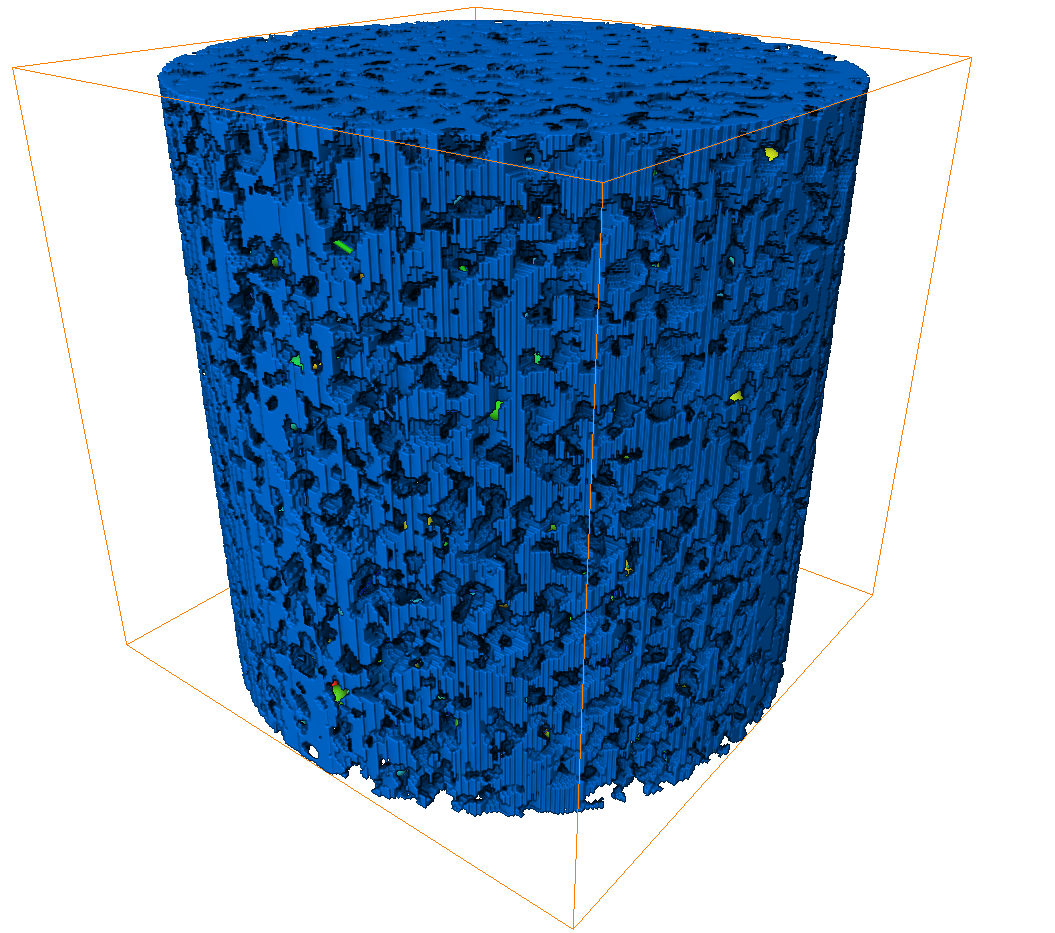

Supplement: S1 Data — (ZIP) [file pone.0296437.s001.zip › SI-Data/Data aggregation/unbiomineralization sample/3D unbiomineralization sample/24-kongxiwangluomoxing jia kongxi shiti .png]

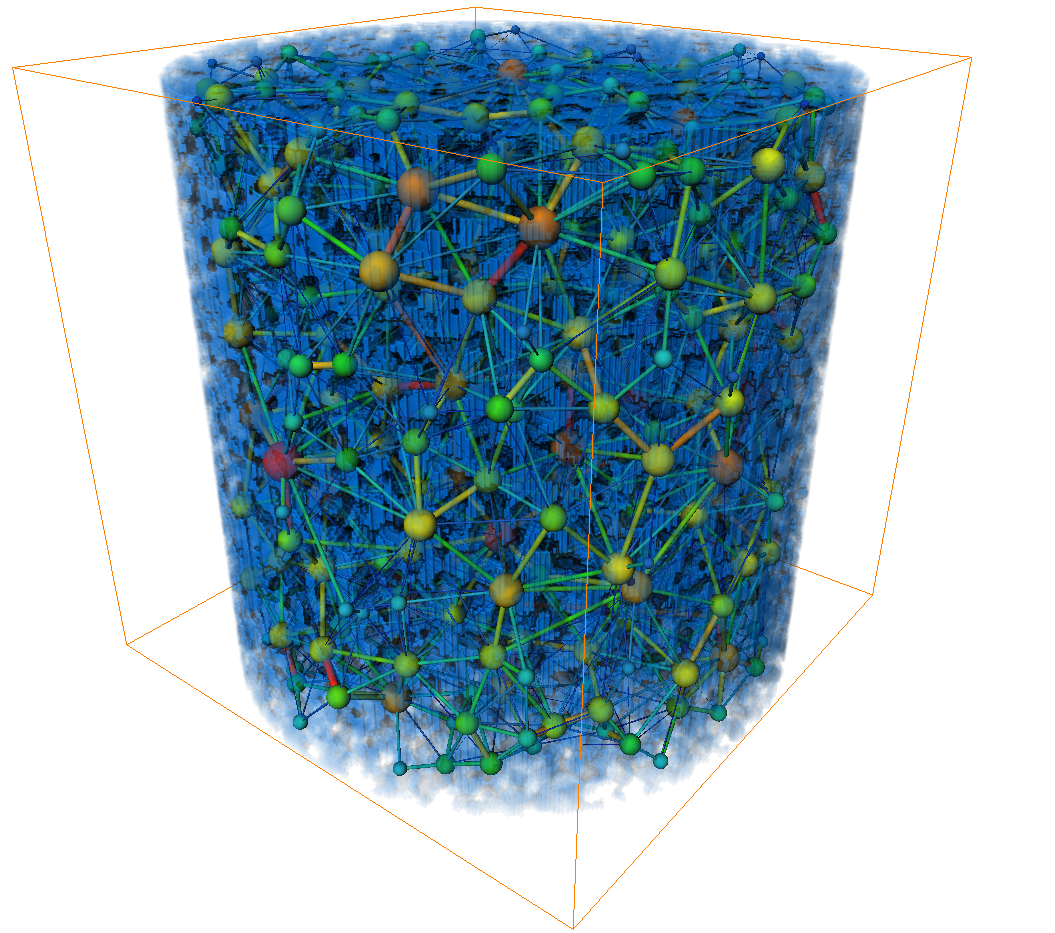

Supplement: S1 Data — (ZIP) [file pone.0296437.s001.zip › SI-Data/Data aggregation/unbiomineralization sample/3D unbiomineralization sample/24-kongxiwangluomoxing jia kongxi shiti 2 .png]

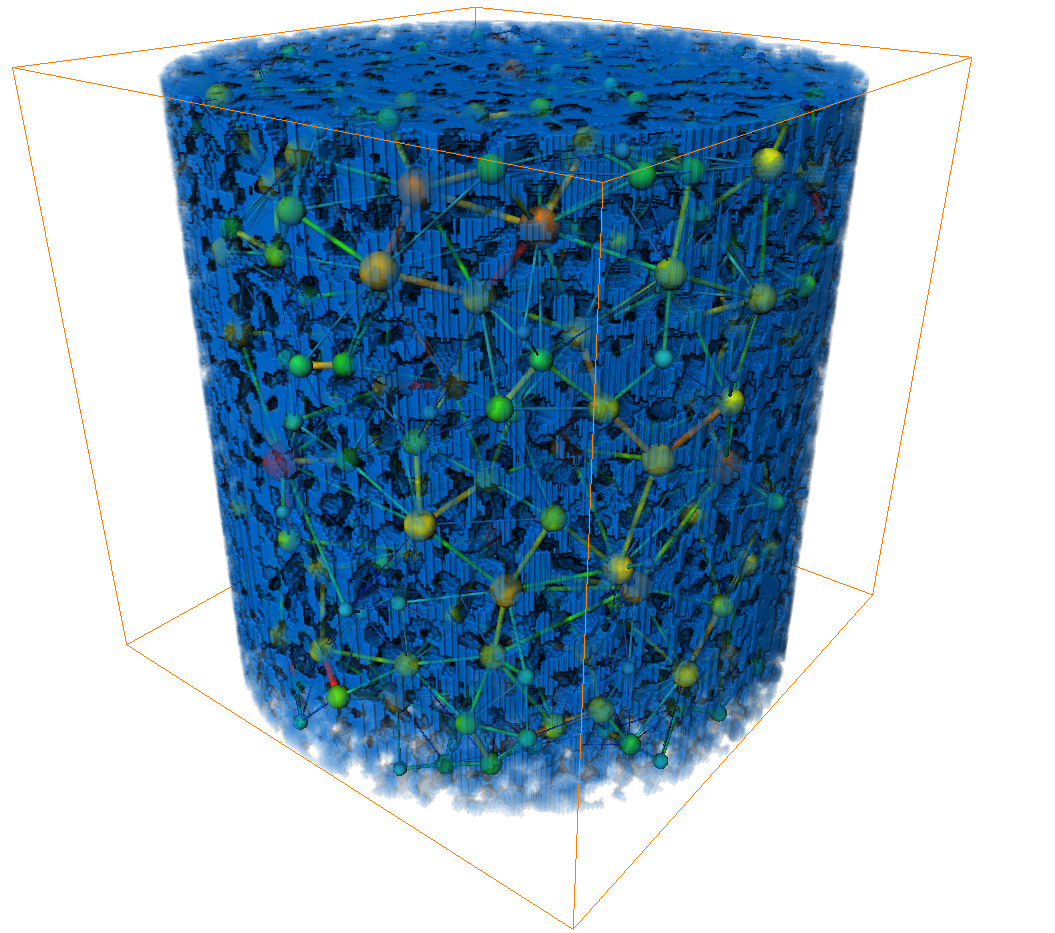

Supplement: S1 Data — (ZIP) [file pone.0296437.s001.zip › SI-Data/Data aggregation/unbiomineralization sample/3D unbiomineralization sample/24-kongxiwangluomoxing jia kongxi shiti 3 .png]

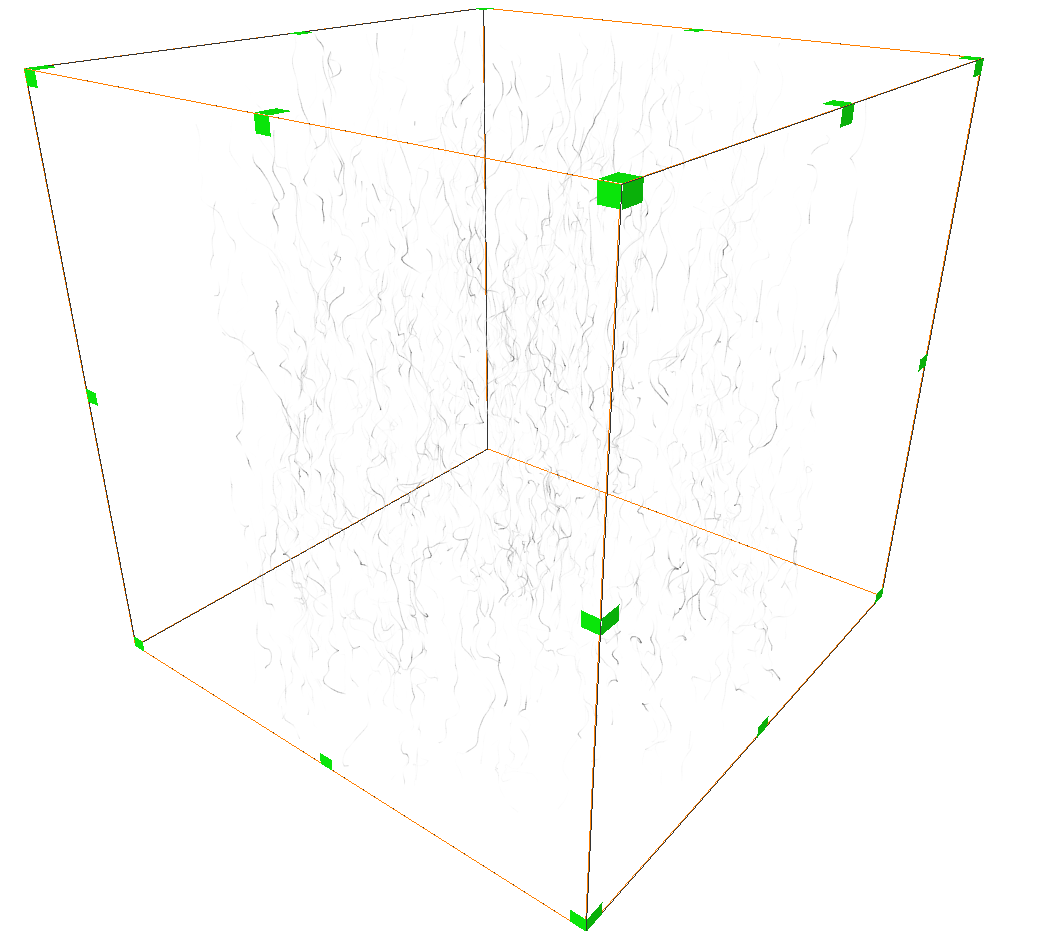

Supplement: S1 Data — (ZIP) [file pone.0296437.s001.zip › SI-Data/Data aggregation/unbiomineralization sample/3D unbiomineralization sample/25-liuxian .png]

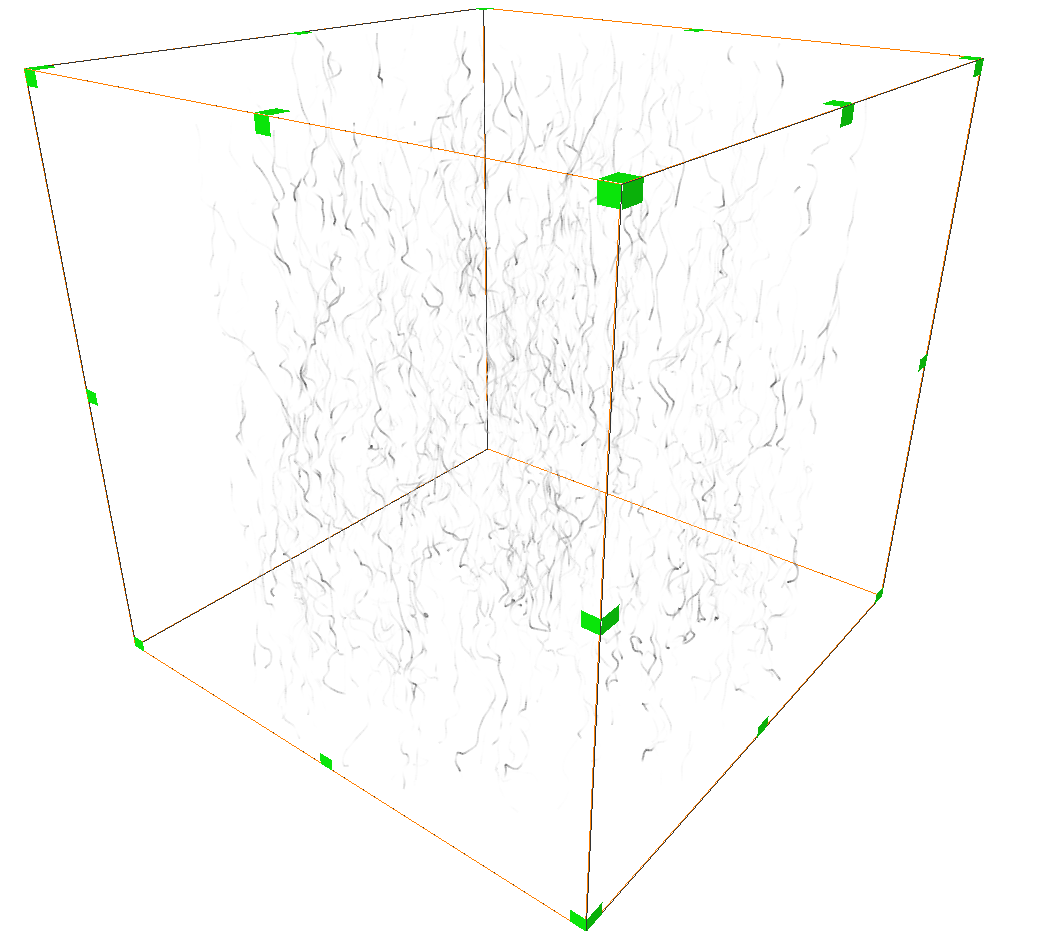

Supplement: S1 Data — (ZIP) [file pone.0296437.s001.zip › SI-Data/Data aggregation/unbiomineralization sample/3D unbiomineralization sample/25-liuxian 1 .png]

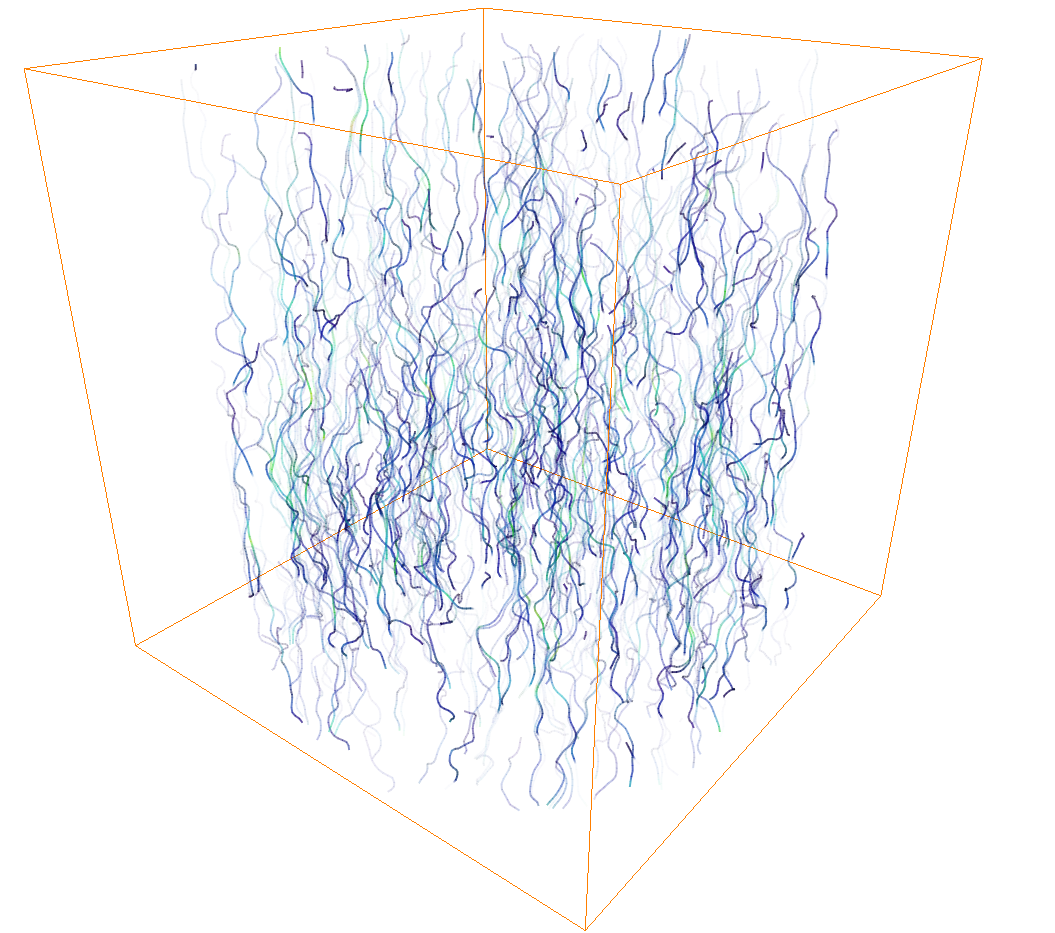

Supplement: S1 Data — (ZIP) [file pone.0296437.s001.zip › SI-Data/Data aggregation/unbiomineralization sample/3D unbiomineralization sample/25-liuxian 2 .png]

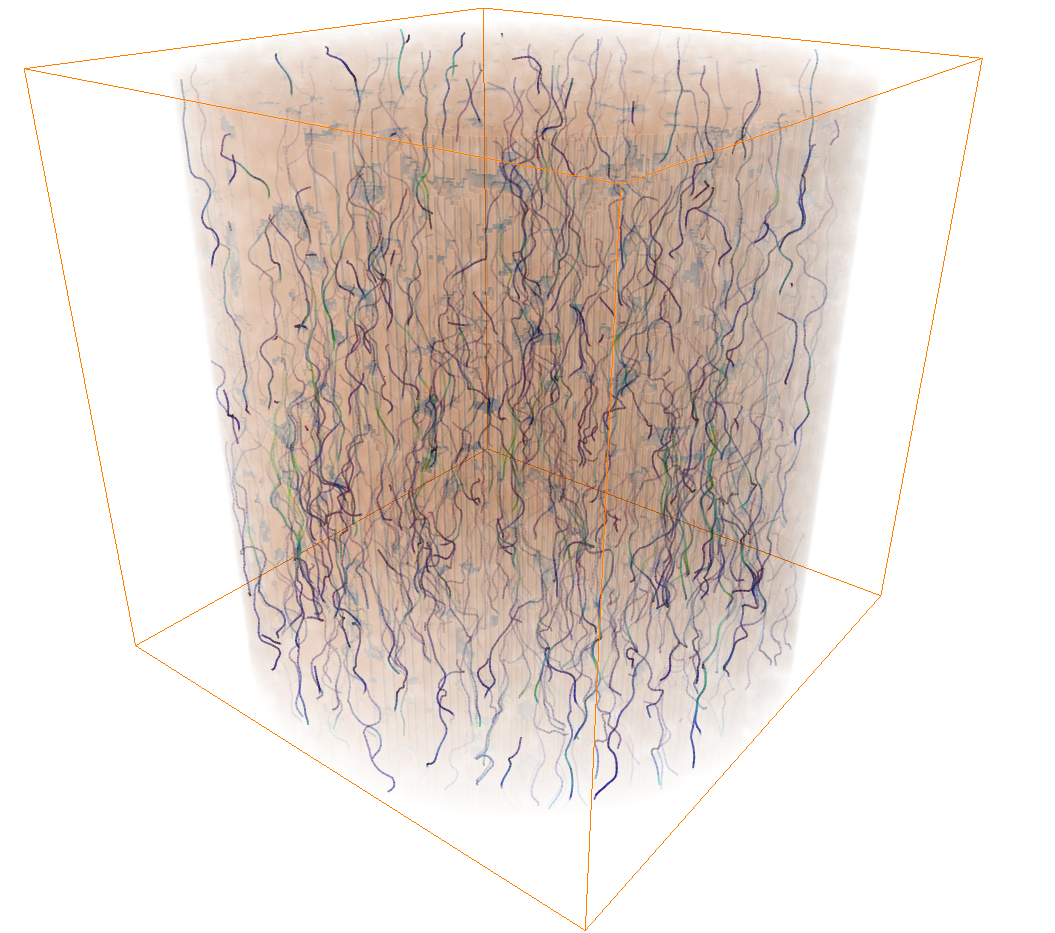

Supplement: S1 Data — (ZIP) [file pone.0296437.s001.zip › SI-Data/Data aggregation/unbiomineralization sample/3D unbiomineralization sample/25-liuxian jia keli shiti .png]

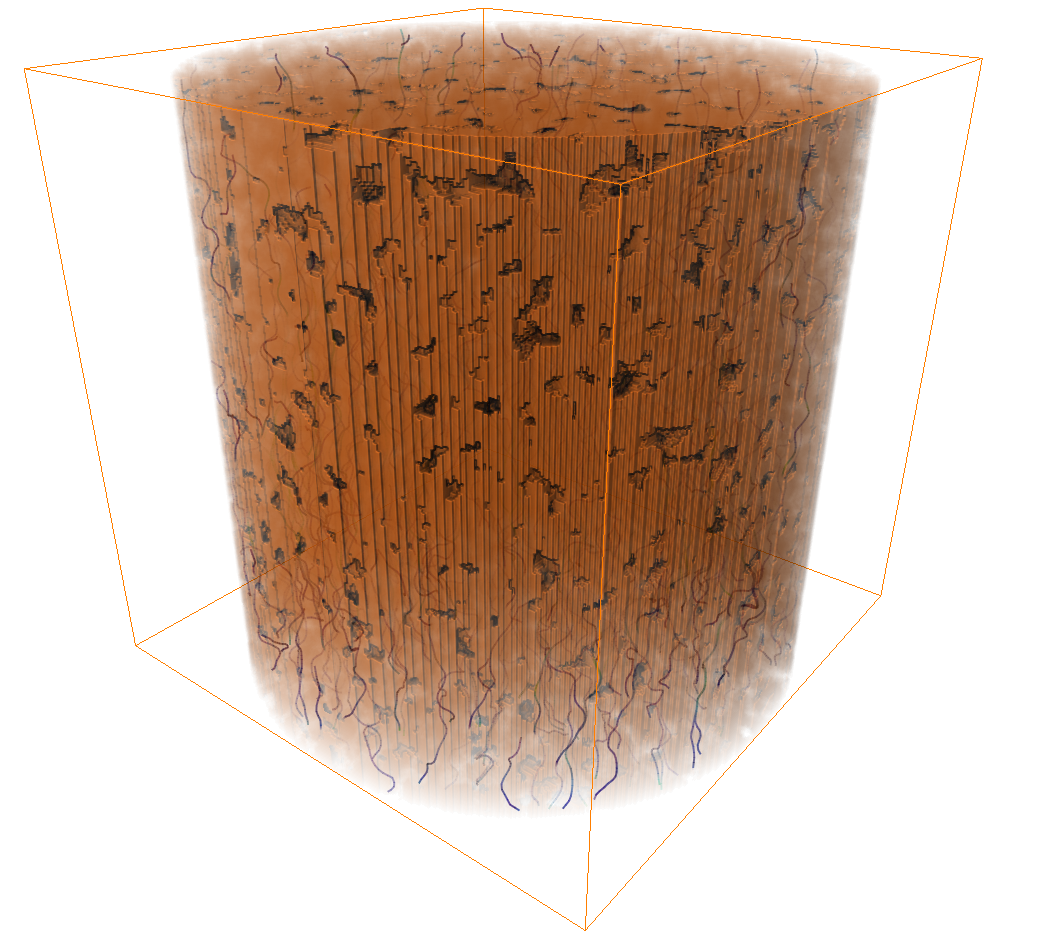

Supplement: S1 Data — (ZIP) [file pone.0296437.s001.zip › SI-Data/Data aggregation/unbiomineralization sample/3D unbiomineralization sample/25-liuxian jia keli shiti 1 .png]

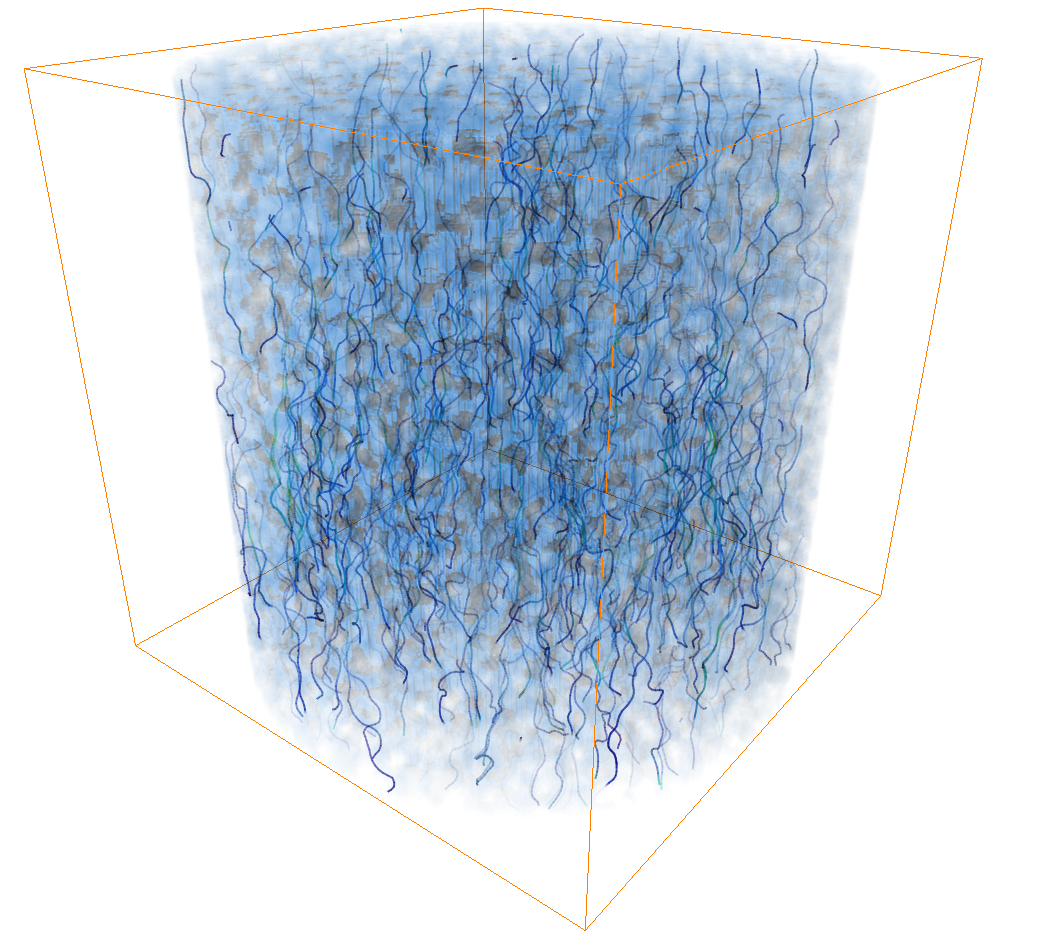

Supplement: S1 Data — (ZIP) [file pone.0296437.s001.zip › SI-Data/Data aggregation/unbiomineralization sample/3D unbiomineralization sample/25-liuxian jia kongxi shiti .png]

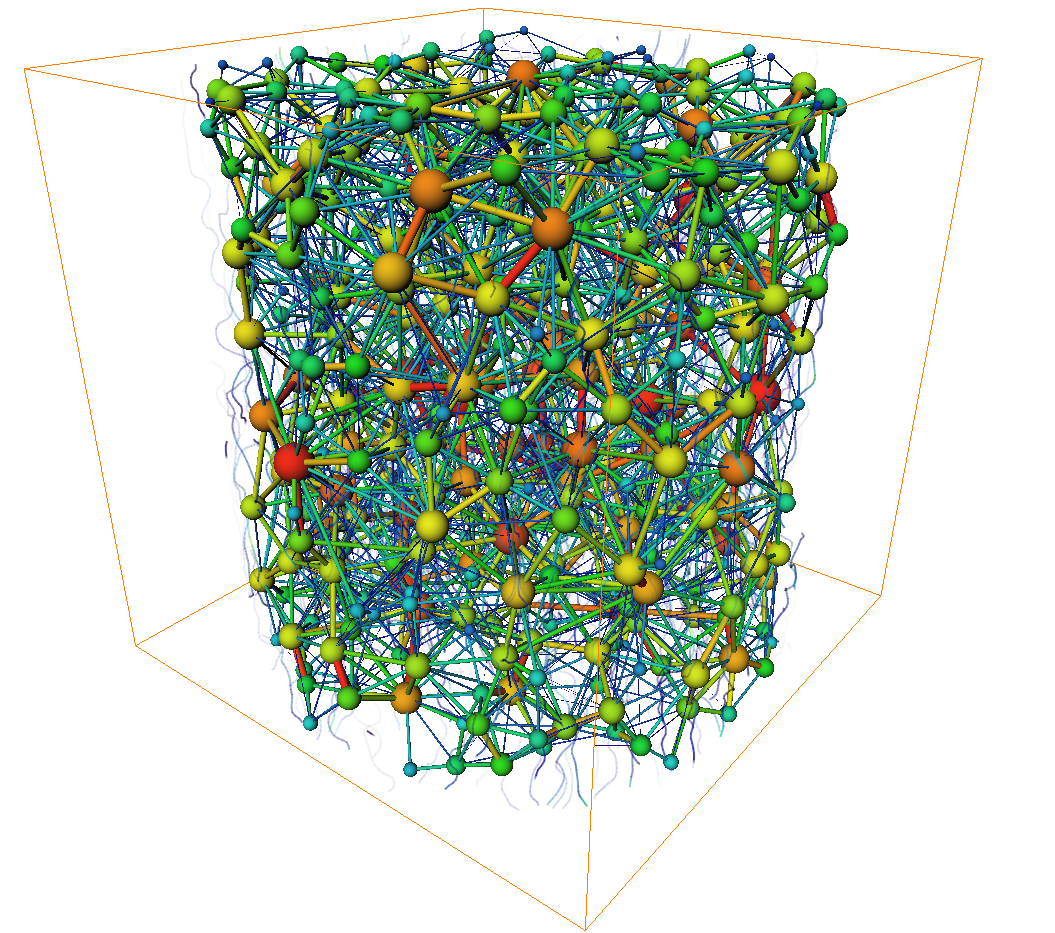

Supplement: S1 Data — (ZIP) [file pone.0296437.s001.zip › SI-Data/Data aggregation/unbiomineralization sample/3D unbiomineralization sample/25-liuxian jia kongxiwangluo moxing .png]

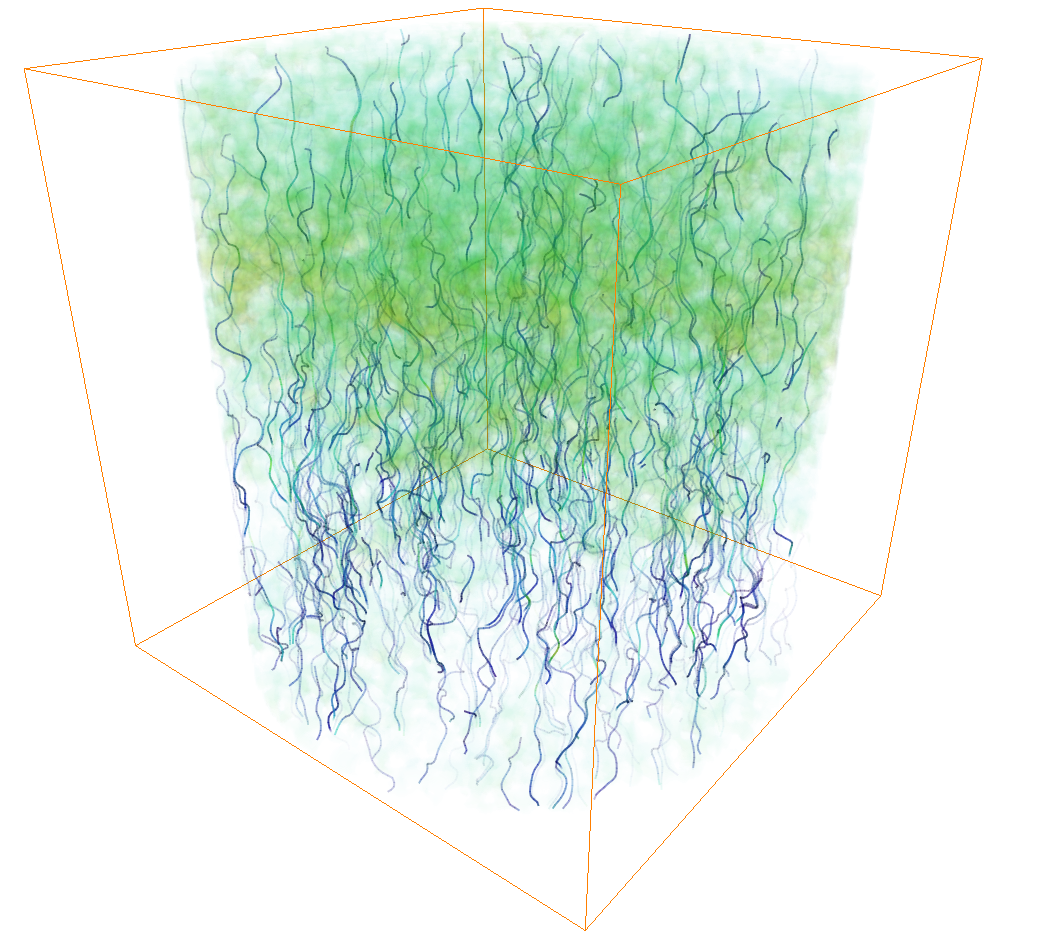

Supplement: S1 Data — (ZIP) [file pone.0296437.s001.zip › SI-Data/Data aggregation/unbiomineralization sample/3D unbiomineralization sample/26-yali jia liuxian 1 .png]

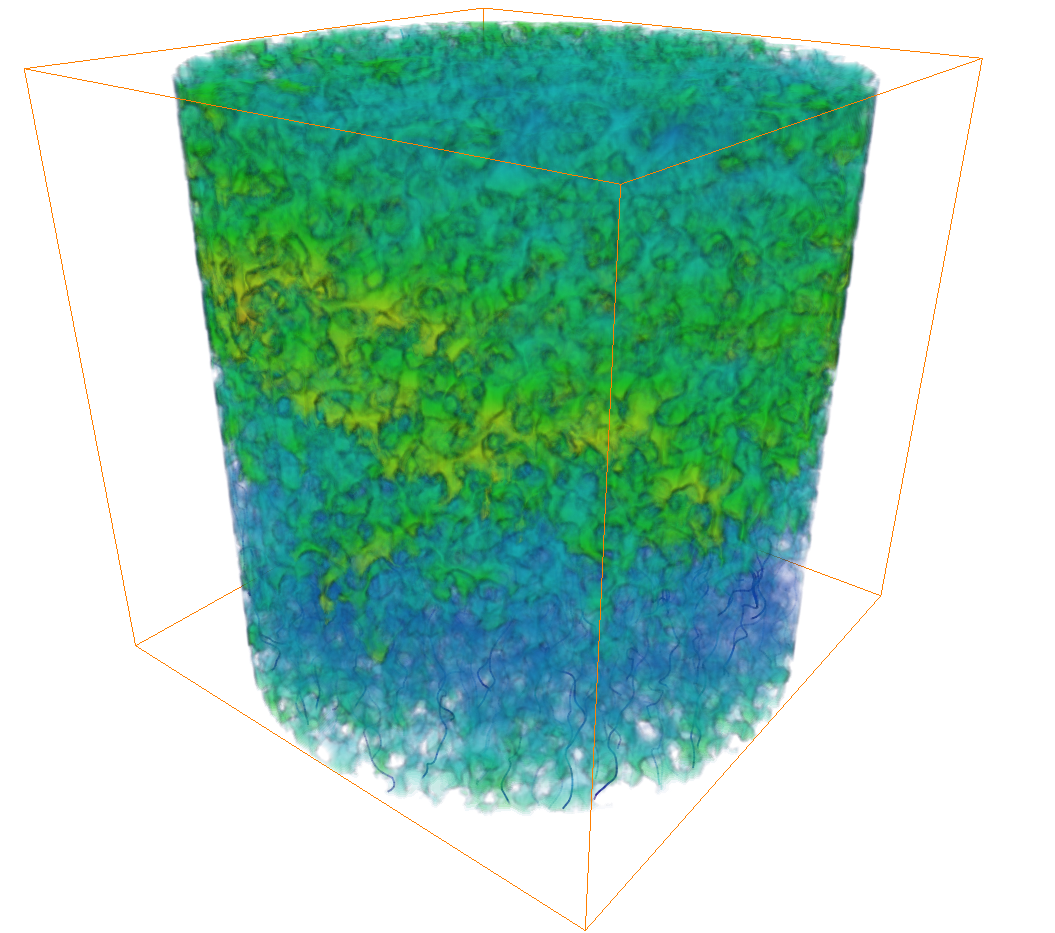

Supplement: S1 Data — (ZIP) [file pone.0296437.s001.zip › SI-Data/Data aggregation/unbiomineralization sample/3D unbiomineralization sample/26-yali jia liuxian 4 .png]
